# Supplementary material for: Short-term particulate matter contamination severely compromises insect antennal olfactory perception
Source: Nat Commun. 2023 Jul 11;14:4112. doi: 10.1038/s41467-023-39469-3 (PMC10336072; doi:10.1038/s41467-023-39469-3)
Supplement: Supplementary file 1 — Supplementary Information [file 41467_2023_39469_MOESM1_ESM.pdf]

Supplementary Information for Short-term particulate matter contamination severely compromises insect antennal olfactory perception

Wang, Liu, et al.

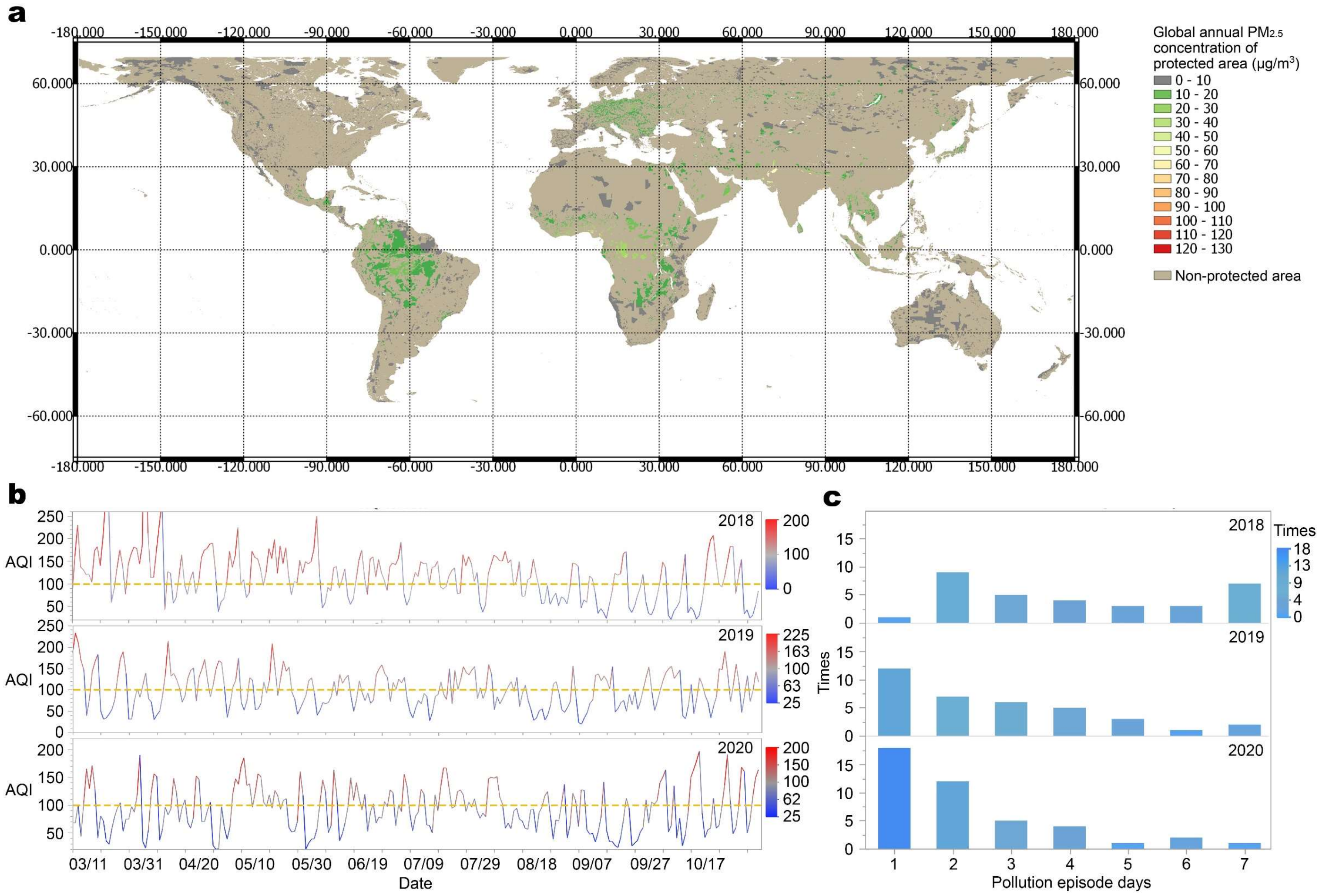

Figure S1. Pattern of PM pollution in Beijing and the world. a. Annual average $PM_{2.5}$ concentration in global land protected areas (except for Antarctica) from 2015 to 2019: about 40% of the total global protected areas are exposed to annual PM concentration that exceeds WHO recommendation of annual average < 10 μg/m^3 [14] (Data source: https://sites.wustl.edu/acag/datasets/surface-pm2-5/#V4.GL.03). **b.** Daily average Air Quality Index (AQI) of Beijing between March and October, when insects are active, from 2018 to 2020, showing intermittent pollution episodes. Yellow line indicates the threshold of light pollution level (AQI > 100). **c.** Frequency of different pollution episodes duration from 2018 to 2020, with the majority shorter than 2 days. The duration of pollution episodes is defined as the number of days with average AQI > 100.

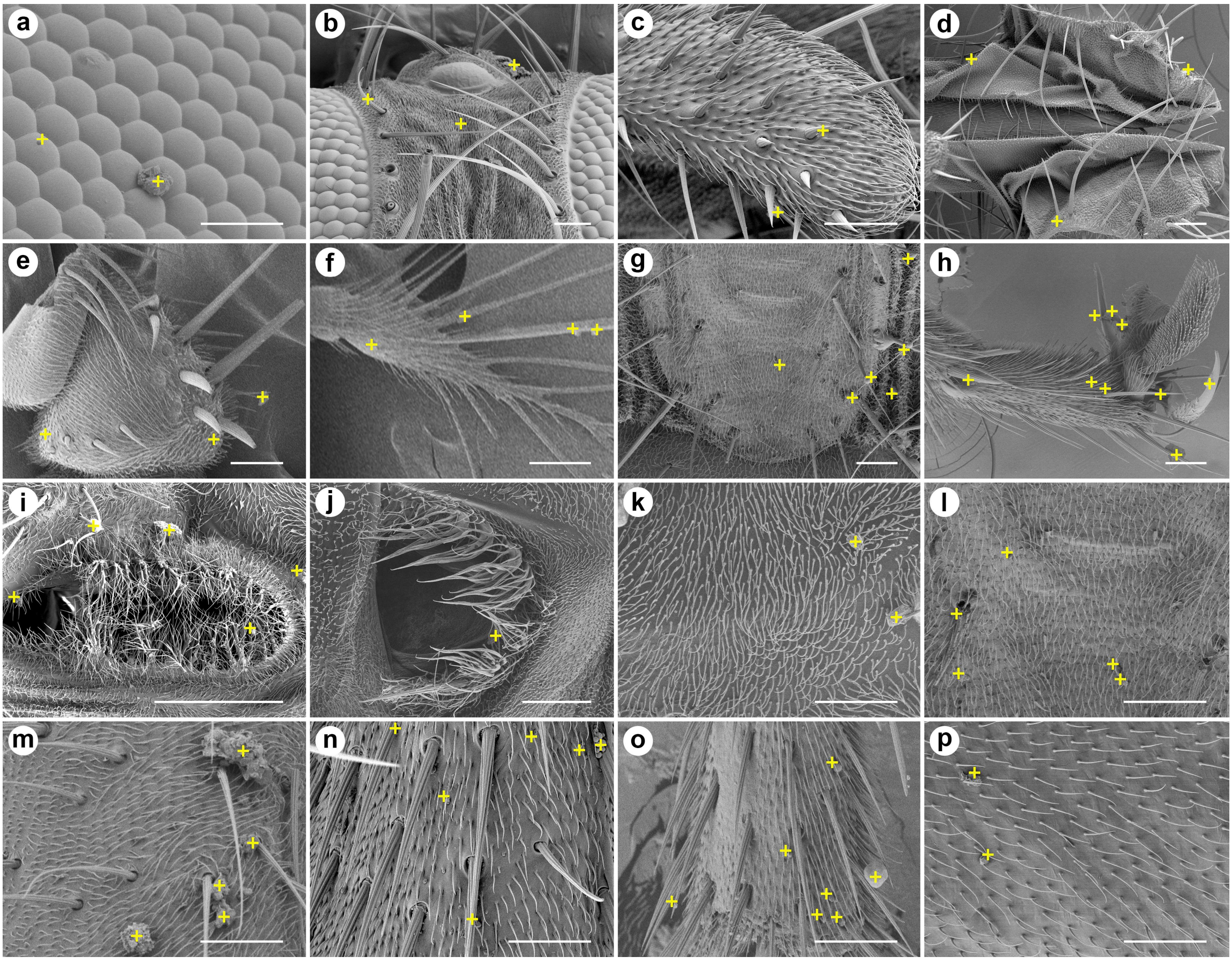

Figure S2. PM can contaminate various body parts of houseflies. SEM micrograph of contaminated housefly, **a.** compound eye, **b.** vertex, **c.** maxillary palp, **d.** mouthpart, **e.** antennal scape and pedicel, **f.** antennal arista, **g.** abdominal segment near ovipositor, **h.** pretarsus, **i.** mesothoracic spiracle, **j.** metathoracic spiracle, **k.** thorax prescutum, **l.** abdomen tergite 5, **m.** fore femur, **n.** fore tibia, and **o.** tarsus of fore legs, and **p.** wing. Yellow crosses mark the position of PM, similar results were observed in 10 individuals (n = 10). Scale bars: a-p = 50 µm.

Supplementary Note 1: Chemical content and classification of PM

The main elemental composition (w/w%) of each type of PM are documented using EDX, with characteristic elements highlighted for each type. The exact elemental composition between the PM on fly antennae and on glass fibre filter may differ, as it is inevitable that the analysis takes up contents of the substrate each particle is on. Silicates are irregular flaky particles (Figs.S3b, g), characterised with Si, Al, and Ca (Fig.S3k). Sulfurates are more regular rectangles or polygons (Figs.S3c, 3h), with a high proportion of Ca and S components (Fig.S3k). Fly ashes are spherical particles (Figs.S3d, i) with various elemental composition, mainly characterised with C and Si (Fig.S3k). Metal particles are often amorphous (Figs.S3e, j), characterised by a high concentration of metal elements, like Fe (Fig.S3k). The determination of characteristic elements and the types of the PM followed Gao 2018[1]. These images confirm that PM can contaminate the antenna and body of houseflies and suggest that this may apply to other flying insects exposed in the similar environment.

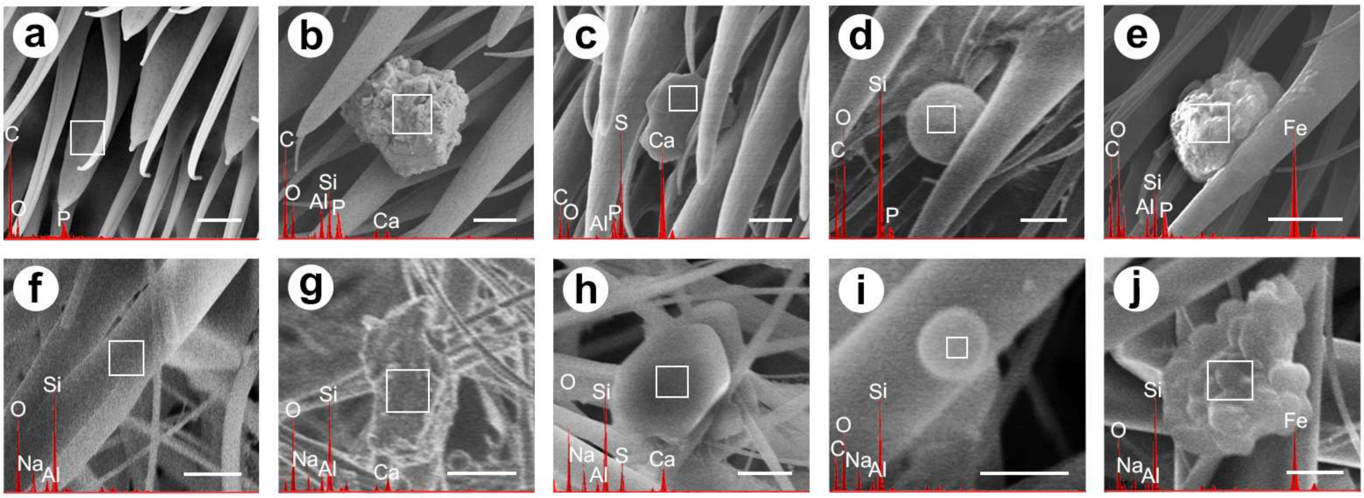

k

Elements Mean% (SE)	C	N	Na	Mg	Al	Si	P	S	Cl	K	Ca	Ti	Cr	Fe	Cu	Zn
**Antennae**																
Surface (n = 10)	74.89 (0.94)	13.15 (1.84)	1.16 (0.10)	0.31 (0.04)	0.86 (0.19)	0.25 (0.04)	4.61 (0.56)	1.79 (0.21)	0.80 (0.17)	1.17 (0.14)	0.14 (0.05)	0.14 (0.03)	0.08 (0.01)	0.13 (0.04)	0.22 (0.07)	0.32 (0.08)
Silicate (n = 21)	47.58 (2.17)	2.58 (0.51)	0.58 (0.16)	1.31 (0.48)	7.66 (1.04)	26.72 (3.07)	3.19 (0.27)	0.50 (0.08)	0.40 (0.20)	3.35 (0.64)	1.57 (0.54)	0.37 (0.06)	0.14 (0.02)	3.21 (0.64)	0.40 (0.08)	0.44 (0.07)
Sulphate (n = 9)	45.05 (4.12)	5.30 (1.10)	0.75 (0.28)	3.86 (1.92)	2.23 (0.42)	2.46 (1.20)	4.48 (0.43)	3.50 (2.32)	0.22 (0.05)	0.94 (0.36)	29.29 (3.22)	0.16 (0.04)	0.09 (0.04)	0.77 (0.30)	0.50 (0.10)	0.39 (0.10)
Fly ash (n = 7)	49.83 (3.10)	8.56 (5.05)	0.71 (0.13)	0.76 (0.34)	0.80 (0.25)	31.02 (8.49)	2.88 (0.94)	0.59 (0.07)	0.57 (0.17)	2.62 (1.77)	0.30 (0.14)	0.16 (0.08)	0.12 (0.05)	0.18 (0.05)	0.35 (0.11)	0.53 (0.22)
Metal particle (n = 7)	35.20 (2.67)	1.05 (0.33)	0.30 (0.11)	1.74 (0.98)	4.67 (1.28)	7.65 (2.46)	3.20 (0.29)	0.54 (0.13)	0.32 (0.06)	1.52 (0.49)	2.72 (2.15)	0.42 (0.23)	0.20 (0.05)	39.41 (4.55)	0.64 (0.50)	0.45 (0.20)
**Glass fibre filter**																
Surface (n = 10)	0.23 (0.16)	0.57 (0.23)	13.47 (0.88)	0.20 (0.06)	6.71 (0.15)	58.36 (1.05)	0.12 (0.03)	1.11 (0.31)	0.00 (0.00)	4.39 (0.19)	4.43 (0.25)	2.97 (0.27)	0.10 (0.03)	0.44 (0.06)	0.90 (0.46)	6.01 (0.52)
Silicate (n = 9)	5.07 (4.59)	1.12 (0.59)	14.83 (1.78)	0.24 (0.04)	5.75 (0.45)	46.05 (3.71)	0.25 (0.16)	6.74 (2.10)	0.00 (0.00)	4.16 (0.33)	5.64 (0.47)	2.82 (0.30)	0.12 (0.03)	0.60 (0.12)	0.46 (0.09)	6.17 (0.77)
Sulphate (n = 6)	0.00 (0.00)	0.32 (0.23)	13.78 (2.90)	0.62 (0.29)	5.74 (0.56)	39.42 (3.06)	0.04 (0.02)	10.6 (2.95)	0.01 (0.01)	4.39 (0.77)	13.09 (2.03)	3.73 (0.86)	0.47 (0.22)	0.87 (0.19)	0.79 (0.36)	6.11 (2.33)
Fly ash (n = 8)	18.07 (7.23)	1.42 (0.69)	7.02 (2.01)	0.38 (0.09)	13.17 (4.38)	37.69 (4.47)	0.30 (0.18)	1.12 (0.23)	0.09 (0.05)	3.64 (0.67)	4.62 (1.02)	3.04 (1.03)	0.48 (0.17)	2.41 (1.01)	1.27 (0.48)	5.28 (1.61)
Metal particle (n = 7)	1.88 (0.44)	0.11 (0.08)	3.64 (0.65)	0.22 (0.06)	2.38 (0.36)	16.19 (4.27)	0.11 (0.05)	0.54 (0.09)	0.03 (0.02)	1.34 (0.39)	1.73 (0.38)	1.03 (0.33)	0.33 (0.15)	66.61 (7.26)	0.88 (0.20)	2.96 (1.26)

Characteristic elements
Low High

Figure S3. SEM micrograph and EDX analysis of particulates on antennal surface and filter surface. The particles on the surface of **a.** antenna and **f.** glass fibre filter. **b-e, g-j.** The different types of particulates on antennal surface and filter surface.

Silicate on **b.** antennal surface and **g.** filter surface. Sulfurate on **c.** antennal surface and **h.** filter surface. Fly ash on **d.** antennal surface and **i.** filter surface. Metal particle on **e.** antennal surface and **j.** filter surface. Chromatographs show the elemental composition of each PM type. **k.** Elemental composition of particulates on antennal surface and filter surface, colours indicate the relative concentration of characteristic elements of each PM types. Scale bars: a-j = 2 μm.

Supplementary Note 2: PM pollution influence gene expression of houseflies

Transcriptome analysis shows that PM impacts the olfactory function more severely for female than male houseflies, and that the impact on male and female antennae are largely different, indicating that different mechanisms by which olfactory function is compromised by PM. The top twenty influenced pathways in antennae (Fig. S4) were highlighted. One of the most enriched pathways in female antennae is the circadian clock genes, which regulates various behaviours and the expression of multiple odorant binding proteins and olfactory receptors in the antennae of dipterans[2,3]. The presence of other genes that are not directly linked to chemoreception suggests that PM affects other antennal functions such as glucose (glycolysis and citrate cycle), amino acid, nucleotide, fatty acid metabolism. As these metabolic pathways provide energetic support and building molecules for antennal cells, this suggests that the PM pollution may affect sensory perception through altering basic cell function.

We also compared transcriptomic analysis results between the bodies of contaminated and uncontaminated houseflies, 151 and 248 genes are expressed differently in the bodies of females and males respectively, with 43 genes overlapping (Fig. S5b). The bodies were collected from the same individuals as the antennal transcriptome analysis, including all the body parts except for antennae. This comparison shows that several important pathways that are closely related to the fitness of flies may be compromised, which corresponds with previous research. For example, the enriched hormone biosynthesis and steroids biosynthesis pathways, suggest hormone regulated physiological processes in adult male flies, such as reproduction, mating, and longevity[4]. Similar to the response in their antennae, the circadian rhythm is among the most enriched pathways in female bodies, which may regulate functions ranging from immunity to flight, foraging and mating behaviour[5,6]. In addition, we subsequently discovered four DEGs that may be involved in the chemical signal transduction on female bodies of houseflies, two of them are olfactory receptors. Also, eight DEGs that may be involved in the chemical signal transduction on male bodies of houseflies, three of which are olfactory receptors, and three are odorant binding proteins (Supplementary Data 5, 6). The expression level change of these genes suggests that the chemical perception organs across the body of houseflies, such as the ones on the maxillary palp, mouth parts, tarsi, and tip of their abdomens are compromised, although further experimental evidence are needed to verify these possible impacts.

We found that there are several DEGs expressed related to odorant or gustatory receptors in both spring and summer antenna samples (23 in summer female sample, 24 in summer male sample, 4 in spring female sample, 1 in spring male sample), but only one DEG overlapped in both of the samples from Spring and Summer, which means that PM contamination in different seasons have different effects on olfactory related gene expression of houseflies, probably as the results of different types of pollution or temperature. This DEG, Odorant receptor 63a, encodes a multi-transmembrane chemoreceptor that mediates response to volatile chemicals[7].

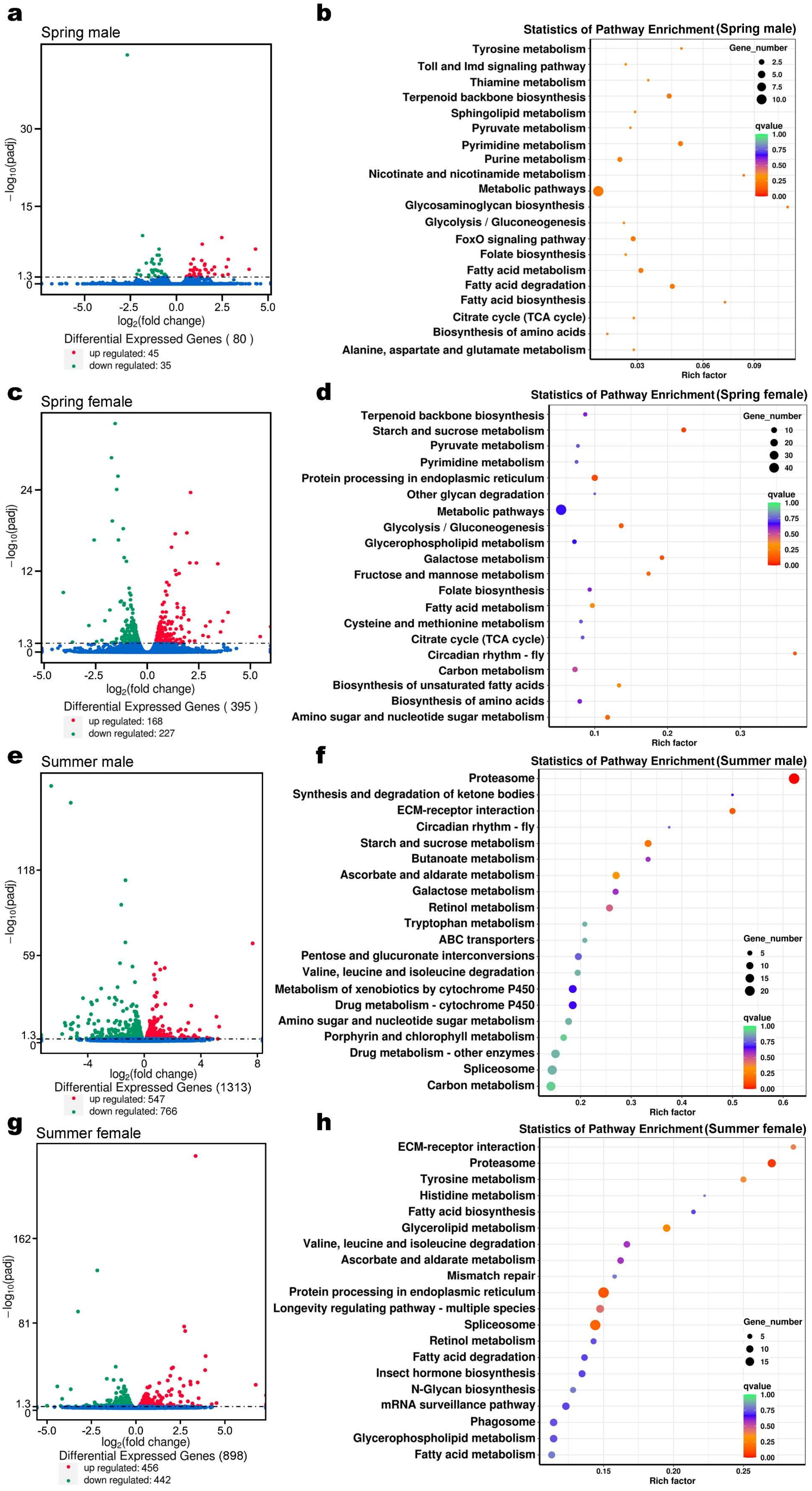
a
Spring male
−log10(padj)
log2(fold change)
Differential Expressed Genes (80)
up regulated: 45
down regulated: 35
b
Statistics of Pathway Enrichment (Spring male)
Tyrosine metabolism
Toll and Imd signaling pathway
Thiamine metabolism
Terpenoid backbone biosynthesis
Sphingolipid metabolism
Pyruvate metabolism
Pyrimidine metabolism
Purine metabolism
Nicotinate and nicotinamide metabolism
Metabolic pathways
Glycosaminoglycan biosynthesis
Glycolysis / Gluconeogenesis
FoxO signaling pathway
Folate biosynthesis
Fatty acid metabolism
Fatty acid degradation
Fatty acid biosynthesis
Citrate cycle (TCA cycle)
Biosynthesis of amino acids
Alanine, aspartate and glutamate metabolism
Gene_number
qvalue
Rich factor
c
Spring female
−log10(padj)
log2(fold change)
Differential Expressed Genes (395)
up regulated: 168
down regulated: 227
d
Statistics of Pathway Enrichment (Spring female)
Terpenoid backbone biosynthesis
Starch and sucrose metabolism
Pyruvate metabolism
Pyrimidine metabolism
Protein processing in endoplasmic reticulum
Other glycan degradation
Metabolic pathways
Glycolysis / Gluconeogenesis
Glycerophospholipid metabolism
Galactose metabolism
Fructose and mannose metabolism
Folate biosynthesis
Fatty acid metabolism
Cysteine and methionine metabolism
Citrate cycle (TCA cycle)
Circadian rhythm - fly
Carbon metabolism
Biosynthesis of unsaturated fatty acids
Biosynthesis of amino acids
Amino sugar and nucleotide sugar metabolism
Gene_number
qvalue
Rich factor
e
Summer male
−log10(padj)
log2(fold change)
Differential Expressed Genes (1313)
up regulated: 547
down regulated: 766
f
Statistics of Pathway Enrichment (Summer male)
Proteasome
Synthesis and degradation of ketone bodies
ECM-receptor interaction
Circadian rhythm - fly
Starch and sucrose metabolism
Butanoate metabolism
Ascorbate and aldarate metabolism
Galactose metabolism
Retinol metabolism
Tryptophan metabolism
ABC transporters
Pentose and glucuronate interconversions
Valine, leucine and isoleucine degradation
Metabolism of xenobiotics by cytochrome P450
Drug metabolism - cytochrome P450
Amino sugar and nucleotide sugar metabolism
Porphyrin and chlorophyll metabolism
Drug metabolism - other enzymes
Spliceosome
Carbon metabolism
Gene_number
qvalue
Rich factor
g
Summer female
−log10(padj)
log2(fold change)
Differential Expressed Genes (898)
up regulated: 456
down regulated: 442
h
Statistics of Pathway Enrichment (Summer female)
ECM-receptor interaction
Proteasome
Tyrosine metabolism
Histidine metabolism
Fatty acid biosynthesis
Glycerolipid metabolism
Valine, leucine and isoleucine degradation
Ascorbate and aldarate metabolism
Mismatch repair
Protein processing in endoplasmic reticulum
Longevity regulating pathway - multiple species
Spliceosome
Retinol metabolism
Fatty acid degradation
Insect hormone biosynthesis
N-Glycan biosynthesis
mRNA surveillance pathway
Phagosome
Glycerophospholipid metabolism
Fatty acid metabolism
Gene_number
qvalue
Rich factor

Figure S4. Transcriptome analysis shows that PM can influence gene expression level and affect metabolic pathways in antennae of houseflies. a, c, e, g. Volcano diagram of the differential expressed genes in antennae of **a.** spring male, **c.** spring female, **e.** summer male, and **g.** summer female houseflies between the uncontaminated and contaminated treatment. **b, d, f, h.** Top twenty most enriched pathways in KEGG analysis in **b.** spring male antennae, **d.** spring female antennae, **f.** summer male antennae, and **h.** summer female antennae. All *p*-values are based on two-sided tests.

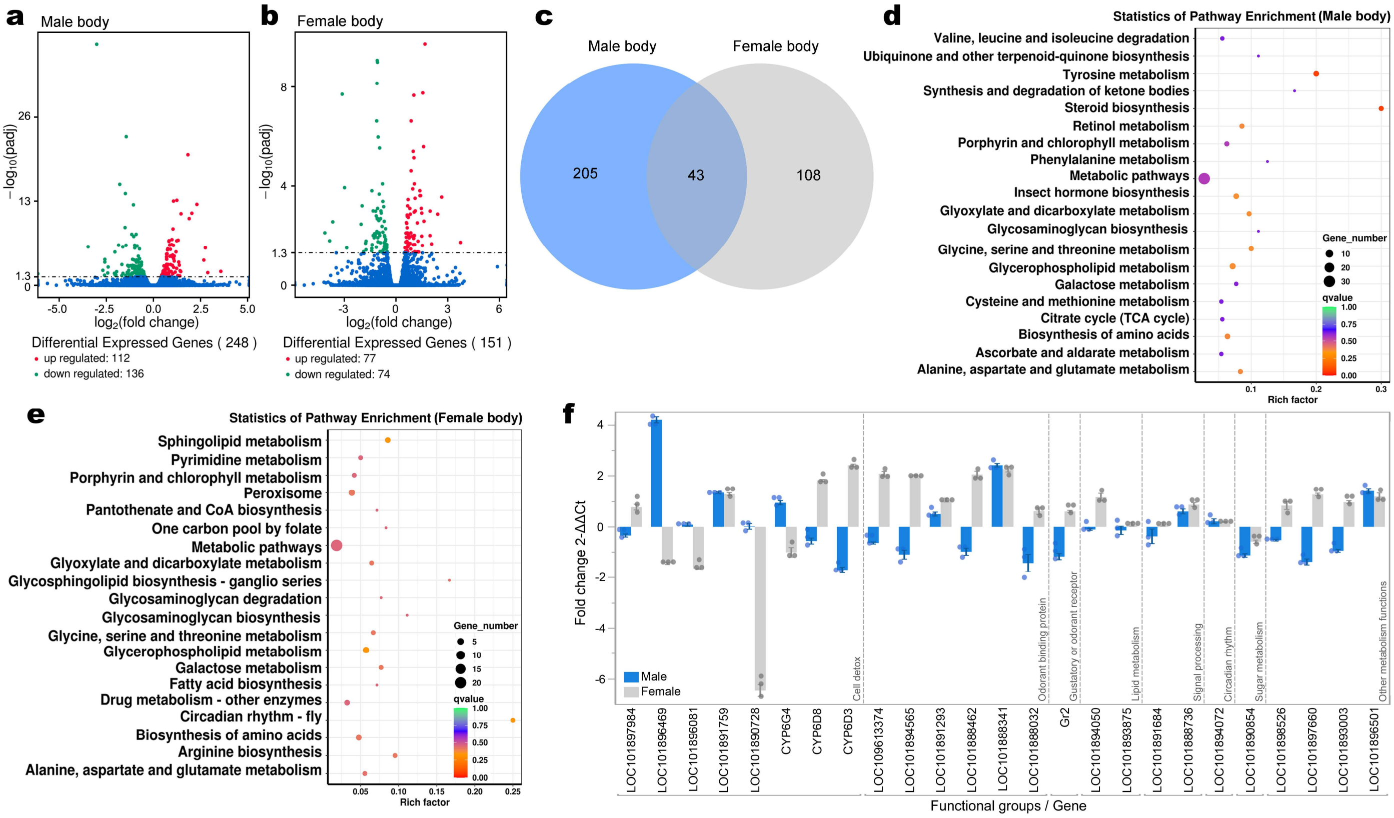

Figure S5. Transcriptome analysis shows that PM influence gene expression level in bodies of houseflies. a-b. Volcano diagram of the differential expressed genes in bodies (all body parts except for antennae) of **a.** male and **b.** female houseflies between the uncontaminated and contaminated treatment. More genes are expressed differently in male bodies. All *p*-values are based on two-sided tests. **c.** Venn diagram of numbers of DEGs in bodies of female and male houseflies. **d-e.** Top twenty most enriched pathways of KEGG analysis in **d.** contaminated vs. uncontaminated male bodies and **e.** contaminated vs. uncontaminated female bodies. **f.** Results of RT-qPCR analysis for DEGs in selected genes from body samples (n = 3). Centre: mean, error bars: SE.

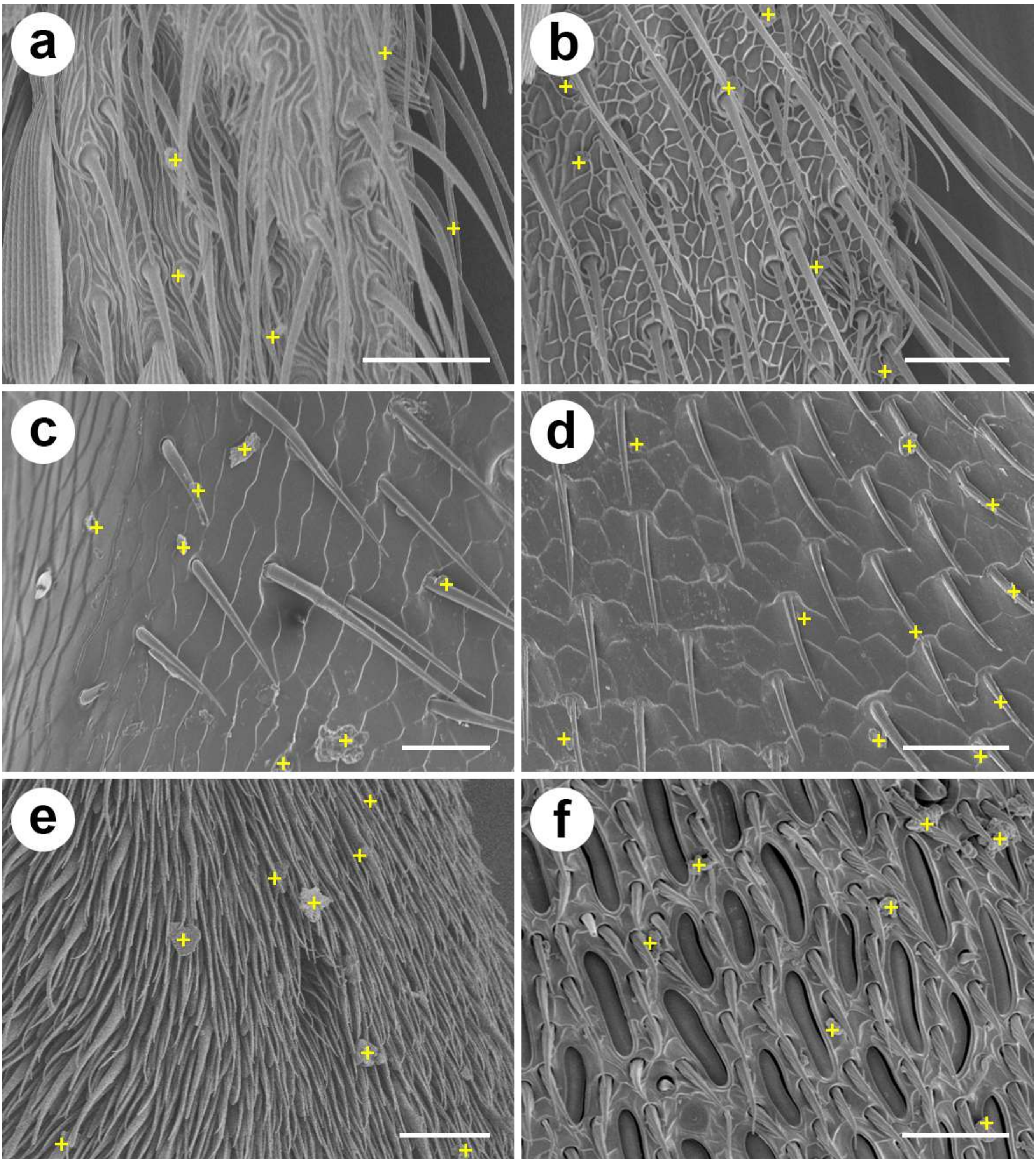

Figure S6. SEM micrograph of antennae of different insects after exposure to air pollution in Beijing, China and in Melbourne, Australia. a, **c**, **e**. Antennal surface of wild insects following exposing to urban PM pollution in Beijing between September and October 2020, **a.** diamondback moth (*Plutella xylostella*), **c.** honeybee (*Apis mellifera*), and **e.** blow fly (Calliphoridae). **b**, **d**, **f**. Antennal surface of wild insects following exposure to PM caused by a nearby bushfire in regional Victoria, Australia in March 2019, **b.** an unidentified moth, **d.** honeybee (*A. mellifera*), and **f.** an unidentified wasp (Vespidae). Yellow crosses mark the position of particulate matter. Scale bars: a-f = 20 µm. Similar results were observed in at least 20 individuals (n = 20).

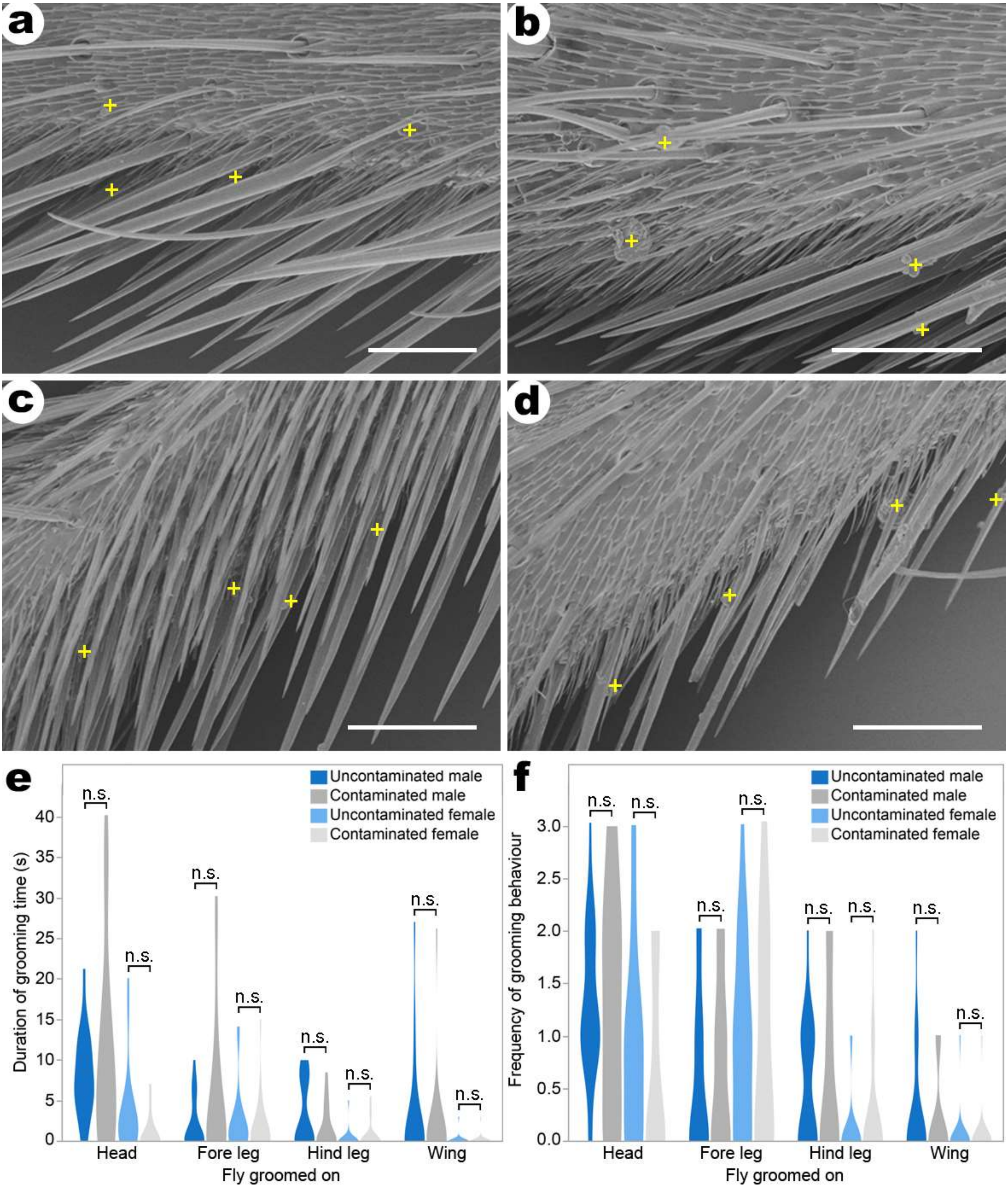

Figure S7. Grooming behaviour of housefly cannot defend antennal surface from PM contamination. a-d. SEM micrograph of the tibia of the fore-leg of **a-b.** uncontaminated houseflies and **c-d.** contaminated houseflies. PM can be found on both treatments, suggesting a small amount of PM can be removed by grooming behaviour. Yellow crosses mark the position of PM particles, Similar

results were observed in at least 20 individuals (n = 20). **e-f.** Violin plot of uncontaminated and contaminated houseflies show no differences in their **e.** duration (total grooming time) and **f.** frequency (times of grooming per minute) of grooming behaviour after exposure treatments (Duration of male fly: Full model: $F_{7,159} = 4.64$, $P < 0.001$; Treatment: $F_{1,159} = 0.89$, $P = 0.35$; Body parts: $F_{3,159} = 9.28$, $P < 0.001$; Treatment * Body parts: $F_{3,159} = 1.25$, $P = 0.29$, n = 40. Frequency of male fly: Full model: $F_{7,159} = 5.29$, $P < 0.001$; Treatment: $F_{1,159} = 0.51$, $P = 0.48$; Body parts: $F_{3,159} = 12.12$, $P < 0.001$; Treatment * Body parts: $F_{3,159} = 0.07$, $P = 0.98$, n = 40. Duration of female fly: Full model: $F_{7,159} = 2.30$, $P = 0.03$; Treatment: $F_{1,159} = 0.001$, $P = 0.97$; Body parts: $F_{3,159} = 3.99$, $P = 0.009$; Treatment * Body parts: $F_{3,159} = 1.36$, $P = 0.26$, n = 40. Frequency of female fly: Full model: $F_{7,159} = 6.81$, $P < 0.001$; Treatment: $F_{1,159} = 0.25$, $P = 0.62$; Body parts: $F_{3,159} = 15.31$, $P < 0.001$; Treatment * Body parts: $F_{3,159} = 0.49$, $P = 0.68$, n = 40). Assay duration: 2 min, centre: mean, error bars: SE, scale bars: a-d = 50 μm. All *p*-values are based on two-sided tests.

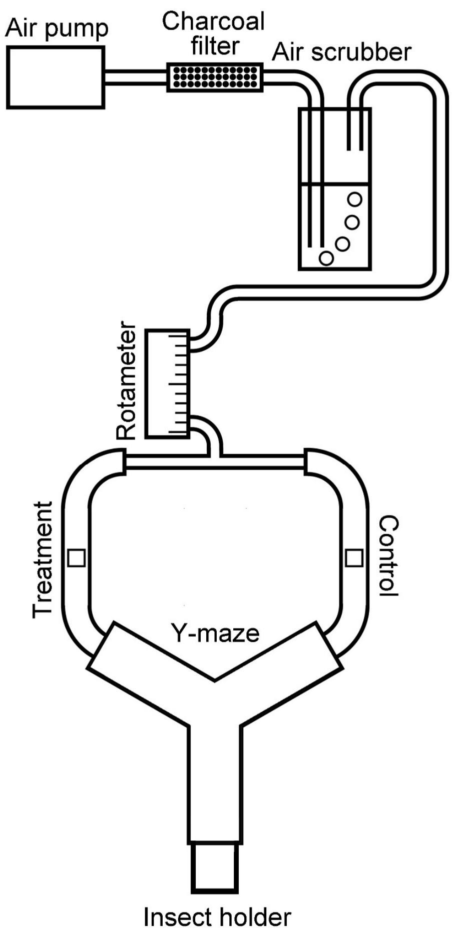

Figure S8. Diagram of Y-maze olfactometer used in the behavioural assays.

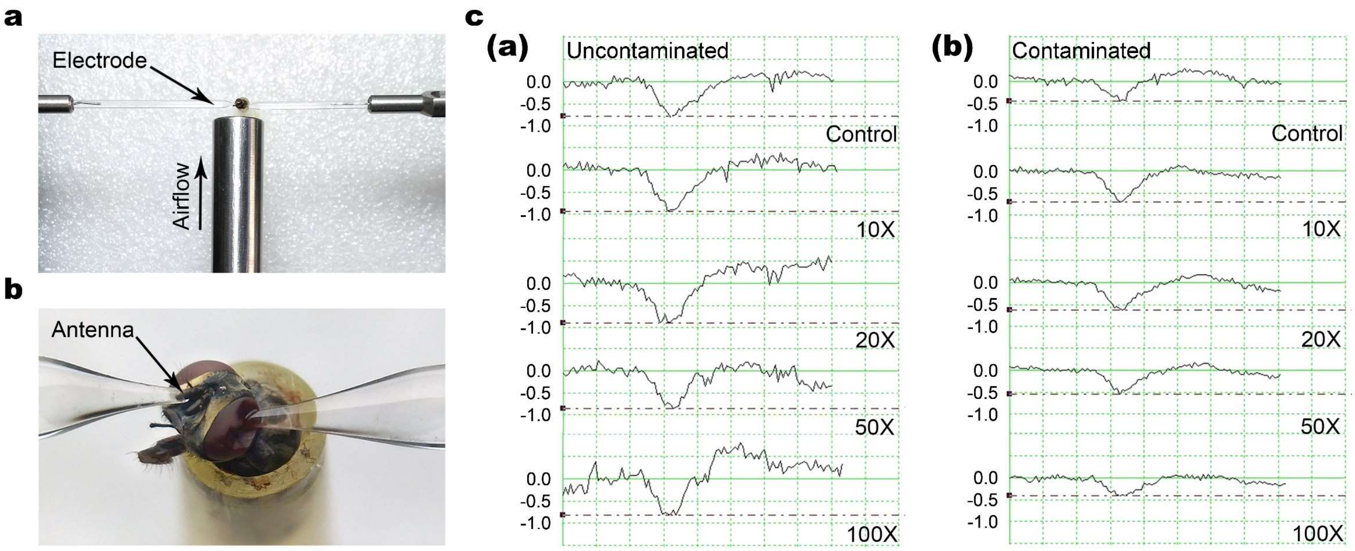

Figure S9. Electroantennogram (EAG) assays show PM pollution influences antennal function of houseflies. a. Equipment of EAG assays, arrows showing the glass electrode and the direction of airflow. **b.** The distal of one antenna and compound eyes of housefly were connected to two glass electrodes to complete the circuit, arrow points to the antenna. **c.** Results of EAG, showing records of voltage changes in uncontaminated **(i)** and contaminated **(ii)** houseflies. Dash lines indicate the maximum voltage changes.

Table S1. The location of different treatments and the number of individuals used in each sample batch in each experiment.

Experiments	Batches	Location of contamination	Control group		Contamination group	
			Room	Number of individuals	Room	Number of individuals
PM densities lab housflies	21/09/2020	Animal Non-Injury Research Laboratory, School of Ecology and Nature Conservation, Beijing Forestry University	Floor 10, Room 1	10	Floor 10, Room 2	10
PM densities lab housflies	29/09/2020		Floor 10, Room 2	20	Floor 10, Room 1	20
PM densities lab housflies	9/10/2020		Floor 10, Room 1	10	Floor 10, Room 2	10
PM densities lab housflies	27/10/2020		Floor 10, Room 2	10	Floor 10, Room 1	6
EAG- aged houseflies-female	3/03/2021	Animal Non-Injury Research Laboratory, School of Ecology and Nature Conservation, Beijing Forestry University	Floor 11, Room 1	30	Floor 11, Room 2	28
EAG- aged houseflies-female	4/03/2021		Floor 11, Room 2	72	Floor 11, Room 1	55
EAG- aged houseflies-female	5/03/2021		Floor 11, Room 1	35	Floor 11, Room 2	49
EAG- aged houseflies-male	3/03/2021	Animal Non-Injury Research Laboratory, School of Ecology and Nature Conservation, Beijing Forestry University	Floor 11, Room 1	18	Floor 11, Room 2	29
EAG- aged houseflies-male	4/03/2021		Floor 11, Room 2	63	Floor 11, Room 1	41
EAG- aged houseflies-male	5/03/2021		Floor 11, Room 1	28	Floor 11, Room 2	28
EAG-sex odour-male	28/03/2021	State Key Laboratory of Infectious Disease Prevention and Control, National Institute for Communicable Disease Control and Prevention, Chinese Center for Disease Control and Prevention	Room 2	30	Room 1	30
EAG-sex odour-male	29/03/2021		Room 1	30	Room 2	30
EAG-sex odour-male	30/03/2021		Room 2	30	Room 1	30
EAG-sex odour-female	28/03/2021	State Key Laboratory of Infectious Disease Prevention and Control, National Institute for Communicable Disease Control and Prevention, Chinese Center for Disease Control and Prevention	Room 2	30	Room 1	30
EAG-sex odour-female	29/03/2021		Room 1	30	Room 2	30
EAG-sex odour-female	30/03/2021		Room 2	30	Room 1	30
EAG-food odour-male	7/04/2021	State Key Laboratory of Infectious Disease Prevention and Control, National Institute for Communicable Disease Control and Prevention, Chinese Center for Disease Control and Prevention	Room 1	35	Room 2	35
EAG-food odour-male	8/04/2021		Room 2	35	Room 1	35
EAG-food odour-male	9/04/2021		Room 1	35	Room 2	35
EAG-food odour-female	7/04/2021	State Key Laboratory of Infectious Disease	Room 1	35	Room 2	35

EAG-food odour-female	8/04/2021	Prevention and Control, National Institute for Communicable Disease Control and Prevention, Chinese Center for Disease Control and Prevention	Room 2	35	Room 1	35
EAG-food odour-female	9/04/2021		Room 1	35	Room 2	35
Behaviour-food odour-male	7/05/2021	State Key Laboratory of Infectious Disease Prevention and Control, National Institute for Communicable Disease Control and Prevention, Chinese Center for Disease Control and Prevention	Room 2	100	Room 1	100
Behaviour-food odour-male	8/05/2021		Room 1	100	Room 2	100
Behaviour-food odour-male	9/05/2021		Room 2	100	Room 1	100
Behaviour-food odour-female	7/05/2021	State Key Laboratory of Infectious Disease Prevention and Control, National Institute for Communicable Disease Control and Prevention, Chinese Center for Disease Control and Prevention	Room 2	100	Room 1	100
Behaviour-food odour-female	8/05/2021		Room 1	100	Room 2	100
Behaviour-food odour-female	9/05/2021		Room 2	100	Room 1	100
Behaviour-sex odour	28/06/2021	State Key Laboratory of Infectious Disease Prevention and Control, National Institute for Communicable Disease Control and Prevention, Chinese Center for Disease Control and Prevention	Room 2	100	Room 1	100
Behaviour-sex odour	29/06/2021		Room 1	100	Room 2	100
Behaviour-sex odour	30/06/2021		Room 2	100	Room 1	100
Transcriptome analysis-male	28/03/2021	Animal Non-Injury Research Laboratory, School of Ecology and Nature Conservation, Beijing Forestry University	Floor 10, Room 2	60	Floor 10, Room 1	60
Transcriptome analysis-female	28/03/2021		Floor 11, Room 1	60	Floor 11, Room 2	60
Transcriptome analysis-male	14/08/2022	Animal Non-Injury Research Laboratory, School of Ecology and Nature Conservation, Beijing Forestry University	Floor 10, Room 1	60	Floor 10, Room 2	60
Transcriptome analysis-female	14/08/2022		Floor 11, Room 2	60	Floor 11, Room 1	60

Table S2. Primers used for qRT-PCR analysis on differentially expressed genes (DEGs) between antennae of uncontaminated and contaminated houseflies.

Number	Primer name	Upstream sequence	Downstream sequence	Product size
1	CYP6G4	ACCTACCGTTATTGGCAGCGTAATG	GACAGGCTCATCGCGTACTTTCG	150
2	Gr2	TCACTTCGTTAAGCGGTCCATTCG	TGCGTGTCTCCCACCACATAATTG	105
3	LOC101887499	AAGGGTCTGTTTCATCGTGCCATC	GCACAGGACACTTCAGCAGTCTG	118
4	LOC101888736	ACGGTGATGTCGCTGTTGAGAAAG	TGCTTCATCCTTTGACGCTGTTCC	128
5	LOC101890728	GGATCTAACTGGCACCAAGAAGGAC	AGCGGATAGTGAAGCAACCATGTG	99
6	LOC101890854	TGGTCATGGATGTGCCGATCTTTG	CTTGGAGGGTTGGGTGCGATAAC	83
7	LOC101891684	TTCCAGTATTTGTGCCCGCTATTCC	TGTTGTTGGCATTTCGGGTTCTTTG	148
8	LOC101893003	GCAGTTGACCGATGGACCTCAG	TGGCATAACAATCTGGGCTGTGAAG	136
9	LOC101893875	TCTTGGCATGAAATTGGCACGATTG	GCCGACTGGAAAGCAACACAAAC	136
10	LOC101894050	TCGCTGCTATATTTTGTGGGTGGAG	TGGAGGACGGTGCTGGATGTG	94
11	LOC101894072	TGCCATACAGTGACGATGACGATTC	TGCGGACAATTCCAGCTCCAATG	116
12	LOC101896081	GCCAAAGTCTATGAGGAGCAGCAG	CTGGGATACAGCCGTAGGGTCTC	128
13	LOC101896469	ATTTGCGGCTATGGATCAGTGTGG	TTTCGCCCTGGCATTGTGACC	121
14	LOC101896501	CTTCCCTGCTTTCATCTGGCTTCTC	TCAGCGGCACTTTGGGTTTCTTC	136
15	LOC101897660	GTTATCGTCGCCGCTGTCTTACC	AGCAGCACCTTGATTTCCTTGTCC	96
16	LOC101897984	TCACGGTCATAGTCACAACACTTCG	CTCTCCTCTCTGCTGATGCCATTG	125
17	LOC101898526	AAGATTTCGGGCACCACAACCAG	GCAGTCCTCGGAGCCCATTATTATC	121
18	LOC101899135	CTATTGATGGCGTGGGCGATGG	TTACTGTGCCTATGATGTCGGTTGC	126
19	CYP6A24	GGGTGGTGCTGCTGGACAAAC	GCTCCGCCATTTATCGCCATCC	146
20	CYP6D3	CGTGATGAACACCAAAGTTGCCTTG	AGACCAACACGAAGACCATTGACTG	135
21	CYP6D8	GCCGCCGCCTATATGCCATTC	CGCCGAGGGATCAACTTTGACTTC	129
22	LOC101888032	AAAGAAGAATACAGGGCTGGCTTGG	ATGGTGTAGGCTGCTTCACAGTTG	83
23	LOC101888341	TGCGTTGGTAAGACTGGTGTAACTG	ACCGTCGTCATGCAACACATCC	132
24	LOC101888462	TGATGCGTTTGGAGAGCTTGACAC	TCGCCATCTTTCGTGCCACATG	94
25	LOC101889928	CAGTAGTGCGGTCCTTGGTGTTC	AAAAGCCCCTGGGTGTTGTGTG	90
26	LOC101891293	GAGGCCAAGGAATCGATGAAGGATC	AAAGGCTCCATCACAGTCGTGTTC	99
27	LOC101894565	CGGCTGCTGTTGTTCGTGGAG	GCCCCAACTTCCGTTTTACATTCAG	91
28	LOC109613374	TAGAGACCCTGGATGCCTTGCG	CGGTGGCCTTTGGTATACTGAAGTC	144
29	LOC101891759	AATGGCTTTCCTTGATGTGCTGTTG	ACTTGTCGTGGTGTCATGTCCTTC	118
30	GAPDH	CTGCTTCTACCGGTGCCGCC	TGGCTGGCTTGCCAAGACGG	138

Table S3. Generalised linear mixed effect models with fixed effect tests used in comparing average density of PM detected on the antennal surface of uncontaminated, experimentally contaminated, and wild houseflies captured in good, moderate, and heavy air quality conditions. All *p*-values are based on two-sided tests.

	df	F	P
Full model: *RSquare* = 0.627, n = 25			
REML Variance Component Estimates			
Batch		3.316	0.7997
Fixed Effects Tests			
Treatment [Contaminated, Uncontaminated, Good, Moderate, Heavy]	4	43.2507	<.0001

Table S4. Generalised linear mixed effect models with fixed effect tests used in comparing average density of PM detected on different body parts of contaminated housefly. All p-values are based on two-sided tests.

	df	F	P
Full model: *RSquare* = 0.466, n = 10			
REML Variance Component Estimates			
Batch		<.0001	0.3442
Fixed Effects Tests			
Treatment [Contaminated, Uncontaminated, Good, Moderate, Heavy]	6	18.4098	<.0001

Table S5. Wilcoxon Test used to compare PM captured on antennal surface and fibre glass filters; p.adj is adjusted from the original p-value (p). All p-values are based on two-sided tests.

Size	y	group 1|	group 2	p	p.adj	Significant
$>PM_{10}$	Percentage	Antennae	Glass fibre filter	0.835	0.84	ns
PM_{10}	Percentage	Antennae	Glass fibre filter	0.0000697	0.00021	****
$PM_{2.5}$	Percentage	Antennae	Glass fibre filter	0.000286	0.00057	***

Table S6. Details of generalised linear models, with binomial distribution and logit link function, explaining the variation in Y-maze olfactometer choices for food odour by (a) female and (b) male houseflies, and for female body odour by (c) male houseflies. All p-values are based on two-sided tests.

	Estimate	SE	df	χ^2	P
**(a) Female response to food odour** (Full model: χ^2 = 20.44, *df* = 9, p = 0.015, n = 60)					
REML Variance Component Estimates					
Batch					0.42
Fixed Effects Tests					
Treatment [Contaminated, Uncontaminated]			1	14.67	< 0.001
Dilution [1×, 2×, 5×, 8×, 10×]			4	0.65	0.6
Treatment * Dilution			4	0.58	0.7
**(b) Male response to food odour** (Full model: χ^2 = 17.38, *df* = 9, p = 0.043, n = 60)					
REML Variance Component Estimates					
Batch					0.97
Fixed Effects Tests					
Treatment [Contaminated, Uncontaminated]			1	10.46	0.001
Dilution [1×, 2×, 5×, 8×, 10×]			4	1.20	0.3
Treatment * Dilution			4	0.45	0.8
**(c) Male response to female odour** (Full model: χ^2 = 22.61, *df* = 9, p = 0.007, n = 60)					
REML Variance Component Estimates					
Batch					0.17
Fixed Effects Tests					
Treatment [Contaminated, Uncontaminated]			1	12.74	< 0.001
Dilution [2×, 10×, 40×, 70×, 100×]			4	0.90	0.5
Treatment * Dilution			4	0.04	0.9

Table S7. Details of generalised linear mixed effect models, explaining the response of EAG for (Z)-9-Tricosene by (a) female and (b) male houseflies, for food odour by (c) female and (d) male houseflies, and for food odour by aged (e) female and (f) male houseflies. All *p*-values are based on two-sided tests.

	Estimate	SE	df	t	P
**(a) Female response to (Z)-9-Tricosene** (*RSquare* = 0.672, Observations = 180, n = 15)					
REML Variance Component Estimates					
Individual				30.479	0.0126
Contamination batch				0.001	0.332
Fixed Effects Tests					
Treatment [Contaminated, Uncontaminated]			1	11.992	0.001
Dilution [10×, 20×, 30×, 50×, 70×, 100×]			5	31.384	< 0.001
Treatment * Dilution			5	2.616	0.027
**(b) Male response to (Z)-9-Tricosene** (*RSquare* = 0.728, Observations = 180, n = 15)					
REML Variance Component Estimates					
Individual				18.650	0.048
Contamination batch				0.001	0.811
Fixed Effects Tests					
Treatment [Contaminated, Uncontaminated]			1	35.882	< 0.001
Dilution [10×, 20×, 30×, 50×, 70×, 100×]			5	43.172	< 0.001
Treatment * Dilution			5	10.900	< 0.001
**(c) Female response to food odour** (*RSquare* = 0.829, Observations = 210, n = 15)					
REML Variance Component Estimates					
Individual				17.843	0.061
Contamination batch				0.001	0.607
Fixed Effects Tests					
Treatment [Contaminated, Uncontaminated]			1	63.187	< 0.001
Dilution [2×, 5×, 8×, 10×, 20×, 50×, 100×]			6	119.173	< 0.001
Treatment * Dilution			6	6.030	< 0.001
**(d) Male response to food odour** (*RSquare* = 0.868, Observations = 210, n = 15)					
REML Variance Component Estimates					
Individual				14.137	0.086
Contamination batch				2.201	0.732
Fixed Effects Tests					
Treatment [Contaminated, Uncontaminated]			1	47.894	< 0.001
Dilution [2×, 5×, 8×, 10×, 20×, 50×, 100×]			6	173.822	< 0.001
Treatment * Dilution			6	4.652	< 0.001
**(e) Aged female response to food odour** (*RSquare* = 0.545, Observations = 269, n = 20)					
REML Variance Component Estimates					
Individual				31.103	0.001
Contamination batch				5.828	0.539
Fixed Effects Tests					
Treatment [Contaminated, Uncontaminated]			1	4.815	0.029
Dilution [2×, 5×, 8×, 10×, 20×, 50×, 100×]			6	11.374	< 0.001
Treatment * Dilution			6	1.417	0.209
**(f) Aged male response to food odour** (*RSquare* = 0.561, Observations = 207, n = 20)					
REML Variance Component Estimates					
Individual				26.498	0.010

Contamination batch		13.467	0.435
Fixed Effects Tests			
Treatment [Contaminated, Uncontaminated]	1	0.229	0.648
Dilution [2×, 5×, 8×, 10×, 20×, 50×, 100×]	6	10.626	< 0.001
Treatment * Dilution	6	1.506	0.179

Data availability: The SEM, behaviour, EAG, and RT-QPCR data generated in this study have been deposited in the Open Science Framework database under accession code https://osf.io/v92xe/?view_only=0744a389866a4cdcb602ae967fe61960. The transcriptome data generated in this study have been deposited in the NCBI database under accession code PRJNA909937 https://www.ncbi.nlm.nih.gov/bioproject/PRJNA909937.

Supplementary References

1 Gao, Y. Pollution characteristics of atmospheric particulates in northern urban Beijing during haze-fog and non haze-fog periods. (University of Science and Technology, Beijing, 2018).

2 Gadenne, C., Barrozo, R. B. & Anton, S. Plasticity in Insect Olfaction: To Smell or Not to Smell? *Annual review of entomology* **61**, 317-333 (2016).

3 Rund, S. S. C. *et al.* Daily rhythms in antennal protein and olfactory sensitivity in the malaria mosquito *Anopheles gambiae*. *Scientific Reports* **3**, 2494 (2013).

4 Wijesekera, T. P., Saurabh, S. & Dauwalder, B. Juvenile Hormone Is Required in Adult Males for *Drosophila* Courtship. *Plos One* **11**, e0151912 (2016).

5 Shirasu-Hiza, M. M., Dionne, M. S., Pham, L. N., Ayres, J. S. & Schneider, D. S. Interactions between circadian rhythm and immunity in *Drosophila melanogaster*. *Current biology : CB* **17**, R353-355 (2007).

6 Wager-Smith, K. & Kay, S. A. Circadian rhythm genetics: from flies to mice to humans. *Nature Genetics* **26**, 23-27 (2000).

7 Kreher, S. A., Kwon, J. Y. & Carlson, J. R. The molecular basis of odor coding in the *Drosophila* larva. *Neuron* **46**, 445-456 (2005).
