# Supplementary material for: Short-term particulate matter contamination severely compromises insect antennal olfactory perception
Source: Nat Commun. 2023 Jul 11;14:4112. doi: 10.1038/s41467-023-39469-3 (PMC10336072; doi:10.1038/s41467-023-39469-3)
Supplement: Supplementary file 3 — Description of Additional Supplementary Files [file 41467_2023_39469_MOESM3_ESM.pdf]

Description of Additional Supplementary Files

File Name: Supplementary Data 1
Description: Details of differential expressed genes (DEGs) between antennae of uncontaminated and contaminated female houseflies collected in spring.

File Name: Supplementary Data 2
Description: Details of differential expressed genes (DEGs) between antennae of uncontaminated and contaminated male houseflies collected in spring.

File Name: Supplementary Data 3
Description: Details of differential expressed genes (DEGs) between antennae of uncontaminated and contaminated female houseflies collected in summer.

File Name: Supplementary Data 4
Description: Details of differential expressed genes (DEGs) between antennae of uncontaminated and contaminated male houseflies collected in summer.

File Name: Supplementary Data 5
Description: Details of differential expressed genes (DEGs) between the bodies of uncontaminated and contaminated female houseflies in spring.

File Name: Supplementary Data 6
Description: Details of differential expressed genes (DEGs) between the bodies of uncontaminated and contaminated male houseflies in spring.
