# Supplementary material for: Short-term particulate matter contamination severely compromises insect antennal olfactory perception
Source: Nat Commun. 2023 Jul 11;14:4112. doi: 10.1038/s41467-023-39469-3 (PMC10336072; doi:10.1038/s41467-023-39469-3)
Supplement: Supplementary file 4 — Supplementary Data 1 [file 41467_2023_39469_MOESM4_ESM.pdf]

**Supplementary Data 1. Details of differential expressed genes (DEGs) between antennae of uncontaminated and contaminated female houseflies collected in spring.** Readcount is the average read count of each gen, log2FoldChange is calculated by formular log2 (readcount_CFA/readcount_UFA), pval is the *p* value, padj is the fdr corrected *p* value using Benjamini/Hochberg method. All *p*-values are based on two-sided tests. CFA: contaminated female antennae, UFA: uncontaminated female antennae.

| **Gene_id** | **readcount_CFA** | **readcount_UFA** | **log2FoldChange** | **pval** | **padj** | **gene_name** | **description** |
|---|---|---|---|---|---|---|---|
| Novel00014 | 7537.245 | 4769.258 | 0.66027 | 1.94E-08 | 6.13E-06 | - | - |
| Novel00064 | 210.219 | 343.5999 | -0.70884 | 6.26E-05 | 0.005288 | - | - |
| Novel00120 | 84.47008 | 38.82137 | 1.1216 | 0.000242 | 0.015504 | - | - |
| Novel00123 | 43.26445 | 99.21784 | -1.1974 | 7.38E-05 | 0.005952 | - | - |
| Novel00124 | 1693.019 | 2551.585 | -0.5918 | 3.32E-06 | 0.000439 | - | - |
| Novel00191 | 715.562 | 1079.466 | -0.59317 | 1.01E-05 | 0.001142 | - | - |
| Novel00414 | 390.315 | 224.1678 | 0.80006 | 2.25E-06 | 0.000324 | - | - |
| Novel00526 | 38.79558 | 11.96498 | 1.6971 | 0.000608 | 0.031147 | - | - |
| Novel00590 | 356.6062 | 541.6666 | -0.60307 | 0.000112 | 0.008572 | - | - |
| Novel00703 | 442.1205 | 795.0391 | -0.84659 | 8.24E-05 | 0.006547 | - | - |
| Novel00963 | 37.27293 | 12.54994 | 1.5704 | 0.001131 | 0.047385 | - | - |
| Novel00983 | 292.9741 | 528.807 | -0.85197 | 0.000671 | 0.032795 | - | - |
| Novel01056 | 150.749 | 239.6613 | -0.66885 | 0.000558 | 0.02915 | - | - |
| Novel01163 | 62.62001 | 27.09577 | 1.2086 | 0.000916 | 0.041098 | - | - |
| Novel01302 | 124.211 | 206.6684 | -0.73453 | 0.000903 | 0.041098 | - | - |
| Novel01333 | 119.1503 | 221.7139 | -0.89592 | 0.000723 | 0.0345 | - | - |
| Novel01520 | 278.175 | 141.1361 | 0.97891 | 3.84E-07 | 7.65E-05 | - | - |
| Novel01536 | 1280.825 | 1811.356 | -0.5 | 0.000127 | 0.009388 | - | - |
| Novel01619 | 52.90038 | 112.7257 | -1.0915 | 7.13E-05 | 0.005868 | - | - |
| Novel01700 | 251.6967 | 449.7512 | -0.83744 | 0.000114 | 0.008595 | - | - |
| Novel01713 | 221.93 | 343.6948 | -0.63102 | 0.000284 | 0.017643 | - | - |
| Novel01728 | 65.76915 | 149.6639 | -1.1862 | 3.00E-06 | 0.000406 | - | - |
| Novel01775 | 56.19449 | 20.72571 | 1.439 | 0.000221 | 0.01458 | - | - |
| Novel01777 | 97.28813 | 44.03301 | 1.1437 | 0.00018 | 0.012371 | - | - |

| | | | | | | | | |
|---|---|---|---|---|---|---|---|---|
| Novel01799 | 103.7958 | 49.80543 | 1.0594 | 0.000225 | 0.014751 | - | - |
| gene-CYP6G4 | 112931.7 | 167468.3 | -0.56844 | 6.91E-07 | 0.000122 | CYP6G4 | cytochrome P450 6g1-like |
| gene-Gr2 | 3107.626 | 4132.36 | -0.41115 | 0.000675 | 0.032895 | Gr2 | gustatory and odorant receptor 63a-like |
| gene-LOC101887389 | 710.1956 | 1045.074 | -0.55732 | 3.12E-05 | 0.003051 | LOC101887389 | acyl-CoA desaturase |
| gene-LOC101887437 | 380.0691 | 557.031 | -0.5515 | 0.001196 | 0.048974 | LOC101887437 | serine/arginine repetitive matrix protein 2 |
| gene-LOC101887456 | 430.2276 | 296.0369 | 0.53932 | 0.00091 | 0.041098 | LOC101887456 | saccharopine dehydrogenase-like oxidoreductase |
| gene-LOC101887458 | 9089.886 | 15796.14 | -0.79724 | 6.40E-12 | 3.69E-09 | LOC101887458 | heat shock 70 kDa protein cognate 3%2C transcript variant X2 |
| gene-LOC101887475 | 219.3885 | 54.02036 | 2.0219 | 1.03E-05 | 0.001158 | LOC101887475 | farnesyl pyrophosphate synthase-like |
| gene-LOC101887499 | 241.7068 | 107.8003 | 1.1649 | 0.001142 | 0.0476 | LOC101887499 | venom carboxylesterase-6 |
| gene-LOC101887559 | 1971.519 | 1444.666 | 0.44857 | 0.000367 | 0.02162 | LOC101887559 | uncharacterized protein LOC101887559\|\|uncharacterized LOC101887559%2C transcript variant X2 |
| gene-LOC101887573 | 107.1639 | 56.00358 | 0.93623 | 0.000653 | 0.032234 | LOC101887573 | formin-like protein 3 |
| gene-LOC101887634 | 114.7579 | 57.69821 | 0.99199 | 0.000286 | 0.017652 | LOC101887634 | saccharopine dehydrogenase-like oxidoreductase%2C transcript variant X2\|\|saccharopine dehydrogenase-like oxidoreductase isoform X2 |
| gene-LOC101887665 | 1075.914 | 1595.97 | -0.56887 | 1.54E-05 | 0.001654 | LOC101887665 | calcium-binding mitochondrial carrier protein SCaMC-2 isoform X3\|\|calcium-binding mitochondrial carrier protein SCaMC-2%2C transcript variant X3 |
| gene-LOC101887677 | 45.27347 | 13.00262 | 1.7999 | 7.95E-05 | 0.006346 | LOC101887677 | uncharacterized protein LOC101887677\|\|uncharacterized LOC101887677 |
| gene-LOC101887709 | 359.4128 | 86.62324 | 2.0528 | 0.000403 | 0.023294 | LOC101887709 | phormicin |
| gene-LOC101887828 | 327.1154 | 564.0233 | -0.78595 | 4.35E-07 | 8.45E-05 | LOC101887828 | F-box only protein 28%2C transcript variant X3\|\|F-box only protein 28 isoform X3 |
| gene-LOC101887974 | 570.8843 | 841.9188 | -0.56048 | 0.000104 | 0.00797 | LOC101887974 | rhophilin-2-A |
| gene-LOC101887978 | 124.4807 | 298.581 | -1.2622 | 5.15E-11 | 2.77E-08 | LOC101887978 | protein OPI10 homolog |
| gene-LOC101888002 | 36.19956 | 10.92336 | 1.7286 | 0.000573 | 0.02963 | LOC101888002 | lipoprotein lipase |

| | | | | | | | |
|---|---|---|---|---|---|---|---|
| gene-LOC101888004 | 11070.34 | 16621.7 | -0.58637 | 5.30E-07 | 0.000101 | LOC101888004 | pyruvate carboxylase%2C mitochondrial isoform X2\|\|pyruvate carboxylase%2C mitochondrial%2C transcript variant X2 |
| gene-LOC101888064 | 20.333 | 56.9739 | -1.4865 | 0.000154 | 0.010978 | LOC101888064 | tyrosine aminotransferase isoform X1\|\|tyrosine aminotransferase%2C transcript variant X1 |
| gene-LOC101888130 | 156.9747 | 249.8967 | -0.6708 | 0.000717 | 0.034309 | LOC101888130 | proteoglycan 4%2C transcript variant X2\|\|proteoglycan 4 |
| gene-LOC101888166 | 73.43389 | 131.2942 | -0.83829 | 0.001024 | 0.043928 | LOC101888166 | H/ACA ribonucleoprotein complex subunit 2-like protein |
| gene-LOC101888171 | 1707.94 | 2402.02 | -0.49199 | 7.31E-05 | 0.005936 | LOC101888171 | tubulin beta chain |
| gene-LOC101888337 | 198.602 | 328.9904 | -0.72817 | 0.000714 | 0.034255 | LOC101888337 | 10 kDa heat shock protein%2C mitochondrial |
| gene-LOC101888348 | 1411.717 | 2184 | -0.62952 | 3.67E-07 | 7.40E-05 | LOC101888348 | hypothetical protein%2C transcript variant X2\|\|uncharacterized protein LOC101888348 isoform X2 |
| gene-LOC101888428 | 264.9164 | 144.4425 | 0.87504 | 2.75E-05 | 0.002742 | LOC101888428 | aquaporin AQPcic |
| gene-LOC101888470 | 432.6642 | 639.2775 | -0.56319 | 0.000242 | 0.015504 | LOC101888470 | protein CREBRF homolog%2C transcript variant X2 |
| gene-LOC101888515 | 15.68618 | 45.13604 | -1.5248 | 0.000622 | 0.031392 | LOC101888515 | uncharacterized LOC101888515\|\|uncharacterized protein LOC101888515 |
| gene-LOC101888550 | 327.1 | 198.1683 | 0.72301 | 5.17E-05 | 0.004533 | LOC101888550 | protein roadkill |
| gene-LOC101888609 | 150.2448 | 69.30597 | 1.1163 | 2.67E-06 | 0.000374 | LOC101888609 | uncharacterized LOC101888609\|\|uncharacterized protein LOC101888609 |
| gene-LOC101888662 | 3746.433 | 2440.994 | 0.61805 | 3.54E-07 | 7.22E-05 | LOC101888662 | uncharacterized protein LOC101888662\|\|uncharacterized LOC101888662 |
| gene-LOC101888673 | 21.85006 | 2.808729 | 2.9596 | 6.47E-05 | 0.005407 | LOC101888673 | ctenidin-1-like |
| gene-LOC101888696 | 683.5997 | 1085.877 | -0.66764 | 0.000735 | 0.034767 | LOC101888696 | probable trehalose-phosphate phosphatase C |
| gene-LOC101888736 | 10207.88 | 7218.924 | 0.49983 | 1.30E-05 | 0.001431 | LOC101888736 | bacchus |
| gene-LOC101888799 | 88.86402 | 193.1538 | -1.1201 | 7.28E-05 | 0.005936 | LOC101888799 | phosphoenolpyruvate carboxykinase [GTP] |
| gene-LOC101888842 | 1207.782 | 837.6626 | 0.52792 | 8.96E-05 | 0.007 | LOC101888842 | protein takeout |

| | | | | | | | | |
|---|---|---|---|---|---|---|---|---|
| gene-LOC101888926 | 146.2219 | 59.61412 | 1.2944 | 1.60E-05 | 0.001708 | LOC101888926 | sodium/potassium/calcium exchanger 4 |
| gene-LOC101889000 | 213.9776 | 102.243 | 1.0655 | 4.75E-05 | 0.004237 | LOC101889000 | Membrane-associated protein\|\|protein snakeskin |
| gene-LOC101889021 | 1498.105 | 2045.987 | -0.44966 | 0.000418 | 0.023751 | LOC101889021 | dnaJ homolog shv |
| gene-LOC101889074 | 1902.606 | 2751.688 | -0.53234 | 2.59E-05 | 0.002599 | LOC101889074 | putative uncharacterized protein DDB_G0271606%2C transcript variant X2\|\|putative uncharacterized protein DDB_G0271606 |
| gene-LOC101889145 | 1503.629 | 2145.207 | -0.51267 | 3.63E-05 | 0.003449 | LOC101889145 | uncharacterized LOC101889145%2C transcript variant X2 |
| gene-LOC101889157 | 383.12 | 558.8548 | -0.54468 | 0.000418 | 0.023751 | LOC101889157 | putative inorganic phosphate cotransporter |
| gene-LOC101889191 | 749.1975 | 539.404 | 0.47398 | 0.000929 | 0.041531 | LOC101889191 | serine/threonine-protein kinase nekl-2 |
| gene-LOC101889260 | 184.4605 | 107.6882 | 0.77645 | 0.000543 | 0.028599 | LOC101889260 | protein valois |
| gene-LOC101889269 | 48.1086 | 15.92787 | 1.5947 | 0.000185 | 0.012616 | LOC101889269 | cytochrome P450 6g1 |
| gene-LOC101889292 | 184.2648 | 79.68313 | 1.2094 | 1.49E-07 | 3.42E-05 | LOC101889292 | probable G-protein coupled receptor Mth-like 10 |
| gene-LOC101889378 | 572.3521 | 813.0534 | -0.50645 | 0.000292 | 0.018002 | LOC101889378 | glycogenin-1 isoform X1\|\|glycogenin-1%2C transcript variant X1 |
| gene-LOC101889471 | 89.17651 | 155.1207 | -0.79866 | 0.000453 | 0.025418 | LOC101889471 | facilitated trehalose transporter Tret1-2 homolog |
| gene-LOC101889476 | 853.4075 | 1197.179 | -0.48833 | 0.000348 | 0.020616 | LOC101889476 | putative uncharacterized protein DDB_G0277255%2C transcript variant X8 |
| gene-LOC101889495 | 116.9633 | 192.0257 | -0.71524 | 0.000967 | 0.042739 | LOC101889495 | elongation of very long chain fatty acids protein AAEL008004 |
| gene-LOC101889504 | 1399.512 | 1884.86 | -0.42953 | 0.000731 | 0.034767 | LOC101889504 | dnaJ homolog subfamily C member 3 |
| gene-LOC101889542 | 168.2186 | 88.98506 | 0.9187 | 6.91E-05 | 0.005747 | LOC101889542 | EGF domain-specific O-linked N-acetylglucosamine transferase |
| gene-LOC101889565 | 192.5205 | 46.03433 | 2.0642 | 5.99E-17 | 6.44E-14 | LOC101889565 | alpha-tocopherol transfer protein-like |
| gene-LOC101889696 | 90.49382 | 43.23474 | 1.0656 | 0.000467 | 0.025998 | LOC101889696 | uncharacterized protein LOC101889696\|\|uncharacterized LOC101889696 |
| gene-LOC101889724 | 83.30894 | 39.0282 | 1.094 | 0.000472 | 0.026149 | LOC101889724 | peptide chain release factor 1-like%2C mitochondrial |
| gene-LOC101889838 | 3924.783 | 2521.955 | 0.63807 | 2.87E-07 | 6.01E-05 | LOC101889838 | murinoglobulin-2%2C transcript variant |

| | | | | | | | |
|---|---|---|---|---|---|---|---|
| | | | | | | | X3\|\|murinoglobulin-2 isoform X3 |
| gene-LOC101889957 | 105.3506 | 41.60814 | 1.3403 | 0.000901 | 0.041098 | LOC101889957 | attacin-A-like |
| gene-LOC101889961 | 11.9847 | 37.78462 | -1.6566 | 0.000766 | 0.036031 | LOC101889961 | chaoptin isoform X1\|\|chaoptin%2C transcript variant X1 |
| gene-LOC101890035 | 131.0036 | 219.4716 | -0.74443 | 0.000417 | 0.023751 | LOC101890035 | whirlin%2C transcript variant X7 |
| gene-LOC101890125 | 2210.114 | 3978.217 | -0.848 | 3.03E-12 | 1.88E-09 | LOC101890125 | elongation of very long chain fatty acids protein 7 |
| gene-LOC101890139 | 461.5995 | 657.2489 | -0.5098 | 0.000627 | 0.03141 | LOC101890139 | dual oxidase maturation factor 1 |
| gene-LOC101890180 | 1700.71 | 1231.451 | 0.46578 | 0.000235 | 0.015183 | LOC101890180 | period circadian protein%2C transcript variant X1 |
| gene-LOC101890185 | 1304.674 | 2094.421 | -0.68286 | 8.04E-08 | 2.20E-05 | LOC101890185 | uncharacterized protein LOC101890185 isoform X1\|\|uncharacterized LOC101890185%2C transcript variant X1 |
| gene-LOC101890283 | 1599.993 | 2419.52 | -0.59666 | 2.18E-06 | 0.000317 | LOC101890283 | protein EFR3 homolog cmp44E |
| gene-LOC101890440 | 73.76893 | 18.34609 | 2.0075 | 6.13E-08 | 1.77E-05 | LOC101890440 | lipase 3-like |
| gene-LOC101890446 | 859.6634 | 619.4085 | 0.47288 | 0.000945 | 0.041985 | LOC101890446 | probable galactose-1-phosphate uridylyltransferase |
| gene-LOC101890474 | 13.62086 | 0.306549 | 5.4736 | 6.02E-05 | 0.005137 | LOC101890474 | uncharacterized LOC101890474\|\|uncharacterized protein LOC101890474 |
| gene-LOC101890546 | 307.6552 | 192.3775 | 0.67737 | 0.000207 | 0.013828 | LOC101890546 | rho GTPase-activating protein 28%2C transcript variant X2\|\|rho GTPase-activating protein 28 isoform X2 |
| gene-LOC101890607 | 1123.705 | 501.6322 | 1.1636 | 3.23E-07 | 6.67E-05 | LOC101890607 | lipase 3-like |
| gene-LOC101890610 | 72.08193 | 149.7501 | -1.0548 | 0.000272 | 0.017049 | LOC101890610 | SH3 domain-containing protein C23A1.17 |
| gene-LOC101890630 | 1808.812 | 1321.752 | 0.45259 | 0.000317 | 0.019225 | LOC101890630 | ATP-binding cassette sub-family G member 4%2C transcript variant X2 |
| gene-LOC101890655 | 1074.412 | 1434.823 | -0.41733 | 0.001173 | 0.048378 | LOC101890655 | uncharacterized LOC101890655 |
| gene-LOC101890677 | 872.1874 | 1405.461 | -0.68833 | 1.86E-07 | 4.23E-05 | LOC101890677 | non-lysosomal glucosylceramidase%2C transcript variant X1\|\|non-lysosomal glucosylceramidase isoform X1 |
| gene-LOC101890728 | 653.5245 | 1822.599 | -1.4797 | 2.22E-28 | 8.95E-25 | LOC101890728 | probable cytochrome P450 313a4 |

| | | | | | | | |
|---|---|---|---|---|---|---|---|
| gene-LOC101890854 | 2305.01 | 1309.478 | 0.81578 | 8.57E-11 | 4.32E-08 | LOC101890854 | glucose dehydrogenase [FAD%2C quinone] |
| gene-LOC101890896 | 101.8514 | 8.688401 | 3.5512 | 9.42E-06 | 0.001092 | LOC101890896 | peptidoglycan-recognition protein SA-like |
| gene-LOC101890942 | 702.5819 | 1087.242 | -0.62993 | 3.84E-06 | 0.000499 | LOC101890942 | uncharacterized oxidoreductase YoxD isoform X2\|\|estradiol 17-beta-dehydrogenase 11%2C transcript variant X2 |
| gene-LOC101890961 | 1243.858 | 644.2372 | 0.94916 | 6.12E-12 | 3.66E-09 | LOC101890961 | putative nuclease HARBI1%2C transcript variant X2\|\|putative nuclease HARBI1 isoform X2 |
| gene-LOC101890997 | 157.7735 | 286.4865 | -0.86061 | 7.56E-06 | 0.00091 | LOC101890997 | Kv channel-interacting protein 1 |
| gene-LOC101891012 | 60.90093 | 140.0928 | -1.2018 | 0.000211 | 0.014049 | LOC101891012 | uncharacterized LOC101891012 |
| gene-LOC101891023 | 345.7416 | 505.7248 | -0.54866 | 0.000363 | 0.021454 | LOC101891023 | serine/threonine-protein phosphatase 2B catalytic subunit 3 |
| gene-LOC101891055 | 252.2193 | 446.3724 | -0.82357 | 5.99E-07 | 0.00011 | LOC101891055 | tRNA-splicing ligase RtcB homolog |
| gene-LOC101891295 | 1633.855 | 894.0816 | 0.8698 | 1.92E-08 | 6.13E-06 | LOC101891295 | putative inorganic phosphate cotransporter |
| gene-LOC101891392 | 3306.771 | 4316.788 | -0.38454 | 0.001107 | 0.046607 | LOC101891392 | ATP-dependent 6-phosphofructokinase%2C transcript variant X3\|\|ATP-dependent 6-phosphofructokinase isoform X3 |
| gene-LOC101891408 | 398.4515 | 254.6489 | 0.64589 | 0.000526 | 0.028001 | LOC101891408 | probable cytochrome P450 6a21 |
| gene-LOC101891418 | 1487.113 | 2033.027 | -0.45112 | 0.000406 | 0.023365 | LOC101891418 | protein transport protein Sec23A isoform X2\|\|protein transport protein Sec23A%2C transcript variant X2 |
| gene-LOC101891430 | 10.44192 | 34.66978 | -1.7313 | 0.000917 | 0.041098 | LOC101891430 | heat shock protein 70 |
| gene-LOC101891440 | 1036.63 | 1658.151 | -0.67767 | 2.48E-07 | 5.40E-05 | LOC101891440 | solute carrier family 26 member 6 |
| gene-LOC101891486 | 1431.115 | 3826.005 | -1.4187 | 1.81E-30 | 9.76E-27 | LOC101891486 | uncharacterized protein LOC101891486\|\|uncharacterized LOC101891486 |
| gene-LOC101891523 | 270.1301 | 408.5681 | -0.59692 | 0.000333 | 0.01999 | LOC101891523 | organic cation transporter protein |
| gene-LOC101891572 | 762.684 | 1079.505 | -0.50121 | 0.000232 | 0.015019 | LOC101891572 | membrane alanyl aminopeptidase |
| gene-LOC101891646 | 2399.222 | 3296.501 | -0.45837 | 0.000232 | 0.015019 | LOC101891646 | transcription factor Sp9 |
| gene-LOC101891654 | 82.20984 | 160.8504 | -0.96834 | 3.69E-05 | 0.003456 | LOC101891654 | probable H/ACA ribonucleoprotein complex subunit 1 |
| gene-LOC101891657 | 29.19914 | 173.583 | -2.5716 | 1.83E-20 | 2.69E-17 | LOC101891657 | basic-leucine zipper transcription factor A |

| | | | | | | | |
|---|---|---|---|---|---|---|---|
| gene-LOC101891680 | 38.00248 | 4.592047 | 3.0489 | 1.32E-07 | 3.14E-05 | LOC101891680 | lactase-like protein |
| gene-LOC101891684 | 4151.497 | 12206.19 | -1.5559 | 8.65E-39 | 1.40E-34 | LOC101891684 | proton-coupled amino acid transporter 2 |
| gene-LOC101891702 | 1026.724 | 622.3026 | 0.72236 | 3.99E-05 | 0.003675 | LOC101891702 | estradiol 17-beta-dehydrogenase 11 |
| gene-LOC101891715 | 808.2839 | 1165.58 | -0.52811 | 0.000519 | 0.027754 | LOC101891715 | apoptosis-stimulating of p53 protein 1 isoform X1\|\|apoptosis-stimulating of p53 protein 1%2C transcript variant X1 |
| gene-LOC101891733 | 434.0621 | 666.4553 | -0.61861 | 2.94E-05 | 0.002908 | LOC101891733 | facilitated trehalose transporter Tret1%2C transcript variant X3\|\|facilitated trehalose transporter Tret1 isoform X2 |
| gene-LOC101891759 | 1015.691 | 395.8768 | 1.3593 | 1.84E-21 | 3.29E-18 | LOC101891759 | probable cytochrome P450 4d14 |
| gene-LOC101891842 | 1582.732 | 2114.581 | -0.41795 | 0.000744 | 0.035092 | LOC101891842 | cyclin-dependent kinase-like 1 |
| gene-LOC101891912 | 2733.064 | 1419.475 | 0.94516 | 6.31E-14 | 4.85E-11 | LOC101891912 | chymotrypsin inhibitor |
| gene-LOC101891996 | 113.6828 | 280.685 | -1.3039 | 7.32E-11 | 3.81E-08 | LOC101891996 | venom acid phosphatase Acph-1 |
| gene-LOC101891997 | 977.3662 | 471.4439 | 1.0518 | 1.68E-13 | 1.23E-10 | LOC101891997 | general odorant-binding protein 56h |
| gene-LOC101892036 | 102.3069 | 38.78425 | 1.3994 | 2.80E-06 | 0.000387 | LOC101892036 | uncharacterized protein LOC101892036\|\|uncharacterized LOC101892036 |
| gene-LOC101892041 | 449.0468 | 293.7768 | 0.61215 | 0.000188 | 0.012763 | LOC101892041 | vanin-like protein 2 |
| gene-LOC101892046 | 366.5215 | 534.9617 | -0.54554 | 0.000454 | 0.025418 | LOC101892046 | SRSF protein kinase 1 isoform X4\|\|SRSF protein kinase 1%2C transcript variant X5 |
| gene-LOC101892072 | 6006.194 | 4024.318 | 0.57771 | 7.33E-07 | 0.000129 | LOC101892072 | cytochrome P450 6A1-like |
| gene-LOC101892091 | 6533.359 | 8686.865 | -0.41101 | 0.000569 | 0.029513 | LOC101892091 | gamma-glutamyltranspeptidase 1 |
| gene-LOC101892168 | 672.6099 | 924.6824 | -0.45919 | 0.001058 | 0.045014 | LOC101892168 | eukaryotic translation initiation factor 2-alpha kinase |
| gene-LOC101892221 | 1294.522 | 910.1054 | 0.50831 | 0.00015 | 0.010788 | LOC101892221 | dynein light chain Tctex-type |
| gene-LOC101892248 | 317.7541 | 460.0499 | -0.53388 | 0.000916 | 0.041098 | LOC101892248 | brain-specific angiogenesis inhibitor 1-associated protein 2 isoform X2\|\|brain-specific angiogenesis inhibitor 1-associated protein 2%2C transcript variant X2 |
| gene-LOC101892265 | 142.7452 | 278.4476 | -0.96396 | 6.46E-07 | 0.000117 | LOC101892265 | transcription factor Adf-1 |

| | | | | | | | |
|---|---|---|---|---|---|---|---|
| gene-LOC101892292 | 354.272 | 526.5669 | -0.57176 | 0.000994 | 0.04312 | LOC101892292 | uncharacterized LOC101892292\|\|uncharacterized protein LOC101892292 |
| gene-LOC101892311 | 2746.36 | 4470.892 | -0.70304 | 5.26E-09 | 1.77E-06 | LOC101892311 | trehalase |
| gene-LOC101892323 | 113.7063 | 201.8008 | -0.82762 | 5.53E-05 | 0.004745 | LOC101892323 | nucleoside diphosphate kinase 7 |
| gene-LOC101892399 | 4443.94 | 6575.538 | -0.56527 | 1.52E-06 | 0.000236 | LOC101892399 | facilitated trehalose transporter Tret1 |
| gene-LOC101892443 | 130.5512 | 236.2617 | -0.85577 | 2.50E-05 | 0.002524 | LOC101892443 | gamma-glutamyl hydrolase%2C transcript variant X1\|\|gamma-glutamyl hydrolase isoform X1 |
| gene-LOC101892510 | 2794.449 | 2025.627 | 0.4642 | 0.00015 | 0.010788 | LOC101892510 | GTP cyclohydrolase 1 isoform X1\|\|GTP cyclohydrolase 1%2C transcript variant X1 |
| gene-LOC101892606 | 7667.792 | 4599.486 | 0.73734 | 0.000513 | 0.027648 | LOC101892606 | uncharacterized LOC101892606\|\|uncharacterized protein LOC101892606 |
| gene-LOC101892627 | 251.5728 | 156.6974 | 0.68299 | 0.000476 | 0.026293 | LOC101892627 | uncharacterized protein LOC101892627\|\|uncharacterized LOC101892627 |
| gene-LOC101892719 | 1565.983 | 2355.535 | -0.58899 | 2.16E-05 | 0.002208 | LOC101892719 | uncharacterized LOC101892719%2C transcript variant X1 |
| gene-LOC101892728 | 558.5919 | 1804.695 | -1.6919 | 1.53E-23 | 4.10E-20 | LOC101892728 | phytanoyl-CoA dioxygenase domain-containing protein 1 |
| gene-LOC101892729 | 892.2125 | 1220.049 | -0.45148 | 0.000822 | 0.038221 | LOC101892729 | phosphoserine phosphatase |
| gene-LOC101892804 | 2546.118 | 1675.751 | 0.60349 | 1.30E-06 | 0.000206 | LOC101892804 | SEC14-like protein 2 |
| gene-LOC101892808 | 3495.964 | 2568.233 | 0.44491 | 0.000204 | 0.013725 | LOC101892808 | organic cation transporter protein |
| gene-LOC101892812 | 455.1261 | 653.673 | -0.5223 | 0.000554 | 0.029111 | LOC101892812 | mannose-1-phosphate guanyltransferase beta%2C transcript variant X2 |
| gene-LOC101892829 | 414.1331 | 156.0446 | 1.4081 | 4.30E-15 | 3.47E-12 | LOC101892829 | peptide methionine sulfoxide reductase\|\|LOW QUALITY PROTEIN: peptide methionine sulfoxide reductase |
| gene-LOC101893003 | 2019.156 | 892.4939 | 1.1778 | 2.33E-19 | 3.13E-16 | LOC101893003 | protein takeout\|\|Hemolymph juvenile hormone binding protein (JHBP) |
| gene-LOC101893008 | 508.7614 | 809.1696 | -0.66945 | 3.22E-06 | 0.000429 | LOC101893008 | protein grindelwald |

| | | | | | | | |
|---|---|---|---|---|---|---|---|
| gene-LOC101893009 | 31.93605 | 72.13679 | -1.1755 | 0.000668 | 0.032767 | LOC101893009 | uncharacterized LOC101893009 |
| gene-LOC101893013 | 179.3659 | 89.99281 | 0.99502 | 1.90E-05 | 0.001997 | LOC101893013 | uncharacterized LOC101893013%2C transcript variant X2 |
| gene-LOC101893017 | 2192.853 | 3847.379 | -0.81107 | 3.35E-11 | 1.86E-08 | LOC101893017 | glutamyl aminopeptidase isoform X2\|\|glutamyl aminopeptidase%2C transcript variant X2 |
| gene-LOC101893080 | 798.6332 | 556.9961 | 0.51987 | 0.000258 | 0.016369 | LOC101893080 | ubiquitin-conjugating enzyme E2-22 kDa |
| gene-LOC101893156 | 409.5759 | 247.0709 | 0.72921 | 1.52E-05 | 0.001647 | LOC101893156 | probable serine hydrolase |
| gene-LOC101893236 | 66.19271 | 145.5771 | -1.137 | 7.17E-06 | 0.000876 | LOC101893236 | odorant receptor 67d-like |
| gene-LOC101893282 | 1553.848 | 2472.842 | -0.67033 | 1.16E-07 | 2.93E-05 | LOC101893282 | integral membrane protein GPR155 |
| gene-LOC101893379 | 137.4445 | 227.6914 | -0.72823 | 0.000408 | 0.023423 | LOC101893379 | ribosome biogenesis regulatory protein homolog |
| gene-LOC101893387 | 1702.668 | 2575.597 | -0.59711 | 3.55E-06 | 0.000465 | LOC101893387 | pancreatic triacylglycerol lipase |
| gene-LOC101893442 | 102.4785 | 305.4611 | -1.5757 | 0.001094 | 0.046211 | LOC101893442 | cuticle protein 1 |
| gene-LOC101893496 | 78.47295 | 164.0974 | -1.0643 | 1.11E-05 | 0.001248 | LOC101893496 | uncharacterized LOC101893496\|\|uncharacterized protein LOC101893496 |
| gene-LOC101893618 | 6611.321 | 4754.896 | 0.47552 | 4.30E-05 | 0.003898 | LOC101893618 | nose resistant to fluoxetine protein 6 |
| gene-LOC101893628 | 105.9087 | 31.03539 | 1.7708 | 8.80E-09 | 2.90E-06 | LOC101893628 | pancreatic lipase-related protein 2 |
| gene-LOC101893655 | 941.5058 | 663.5723 | 0.50472 | 0.000231 | 0.015019 | LOC101893655 | Major Facilitator Superfamily protein\|\|uncharacterized protein LOC101893655 |
| gene-LOC101893676 | 938.1976 | 1404.715 | -0.58231 | 1.52E-05 | 0.001647 | LOC101893676 | (11Z)-hexadec-11-enoyl-CoA conjugase |
| gene-LOC101893715 | 55.72991 | 21.6788 | 1.3622 | 0.00052 | 0.027754 | LOC101893715 | nuclear RNA export factor 2 |
| gene-LOC101893720 | 2927.805 | 4313.645 | -0.55909 | 0.000638 | 0.031662 | LOC101893720 | twitchin isoform X2\|\|twitchin%2C transcript variant X2 |
| gene-LOC101893723 | 807.92 | 1251.749 | -0.63166 | 0.000615 | 0.031215 | LOC101893723 | titin%2C transcript variant X2 |
| gene-LOC101893737 | 6915.048 | 4802.908 | 0.52583 | 9.48E-06 | 0.001092 | LOC101893737 | RNA-binding protein Rsf1 |
| gene-LOC101893815 | 4523.301 | 6037.611 | -0.4166 | 0.000529 | 0.028053 | LOC101893815 | organic cation transporter-like protein |
| gene-LOC101893835 | 158.6143 | 249.722 | -0.6548 | 0.000911 | 0.041098 | LOC101893835 | zinc finger protein 704 |
| gene-LOC101893875 | 108.4337 | 34.63664 | 1.6464 | 2.92E-08 | 8.72E-06 | LOC101893875 | lipase 3-like |
| gene-LOC101893978 | 658.6186 | 1006.375 | -0.61165 | 1.29E-05 | 0.001431 | LOC101893978 | uncharacterized LOC101893978 |

| | | | | | | | |
|---|---|---|---|---|---|---|---|
| gene-LOC101894001 | 805.4858 | 294.5292 | 1.4514 | 2.17E-08 | 6.74E-06 | LOC101894001 | probable asparagine synthetase [glutamine-hydrolyzing] |
| gene-LOC101894016 | 324.9383 | 145.1134 | 1.163 | 3.28E-05 | 0.003188 | LOC101894016 | chymotrypsin-elastase inhibitor ixodidin |
| gene-LOC101894050 | 2259.007 | 1649.711 | 0.45348 | 0.000625 | 0.03141 | LOC101894050 | lipase 3-like |
| gene-LOC101894072 | 8544.43 | 5121.904 | 0.7383 | 2.01E-10 | 9.24E-08 | LOC101894072 | protein timeless |
| gene-LOC101894109 | 68.1639 | 142.6342 | -1.0652 | 1.76E-05 | 0.001869 | LOC101894109 | uncharacterized protein LOC101894109 isoform X1\|\|uncharacterized LOC101894109%2C transcript variant X1 |
| gene-LOC101894137 | 801.7999 | 494.9585 | 0.69593 | 1.17E-06 | 0.000189 | LOC101894137 | translocating chain-associated membrane protein 1 |
| gene-LOC101894154 | 3413.757 | 4485.101 | -0.39378 | 0.001037 | 0.044357 | LOC101894154 | actin-binding LIM protein 2 isoform X3\|\|actin-binding LIM protein 2%2C transcript variant X3 |
| gene-LOC101894182 | 335.6416 | 556.2589 | -0.72884 | 4.13E-06 | 0.000529 | LOC101894182 | band 7 protein AGAP004871%2C transcript variant X2 |
| gene-LOC101894192 | 371.3254 | 604.569 | -0.70322 | 5.45E-06 | 0.000682 | LOC101894192 | tetratricopeptide repeat protein 28 |
| gene-LOC101894235 | 31.54638 | 110.5915 | -1.8097 | 1.63E-09 | 6.10E-07 | LOC101894235 | uncharacterized protein LOC101894235\|\|Chitin binding protein |
| gene-LOC101894362 | 469.7294 | 657.9193 | -0.48608 | 0.001078 | 0.045639 | LOC101894362 | pescadillo homolog |
| gene-LOC101894368 | 173.1312 | 345.8232 | -0.99817 | 6.91E-08 | 1.95E-05 | LOC101894368 | uncharacterized protein LOC101894368 isoform X3\|\|serine-rich adhesin for platelets%2C transcript variant X3 |
| gene-LOC101894398 | 13175.57 | 17911.88 | -0.44305 | 0.000153 | 0.010932 | LOC101894398 | sialin\|\|LOW QUALITY PROTEIN: sialin |
| gene-LOC101894425 | 564.9513 | 391.1608 | 0.53036 | 0.000376 | 0.022044 | LOC101894425 | cytochrome P450 4d8%2C transcript variant X2\|\|cytochrome P450 4d8 isoform X2 |
| gene-LOC101894436 | 1087.526 | 718.3025 | 0.59839 | 9.99E-06 | 0.001142 | LOC101894436 | small ubiquitin-related modifier 3 |
| gene-LOC101894440 | 279.1889 | 176.892 | 0.65837 | 0.000486 | 0.026685 | LOC101894440 | wee1-like protein kinase |
| gene-LOC101894472 | 347.0333 | 600.8109 | -0.79184 | 8.62E-05 | 0.006813 | LOC101894472 | tyrosine-protein kinase Btk29A |
| gene-LOC101894477 | 731.5177 | 503.972 | 0.53755 | 0.000193 | 0.013054 | LOC101894477 | protein NASP homolog |
| gene-LOC101894480 | 1934.794 | 2557.517 | -0.40256 | 0.001072 | 0.045494 | LOC101894480 | protein ABHD4 |

| | | | | | | | |
|---|---|---|---|---|---|---|---|
| gene-LOC101894546 | 1242.272 | 839.1659 | 0.56595 | 3.36E-05 | 0.003246 | LOC101894546 | uncharacterized LOC101894546\|\|uncharacterized protein LOC101894546 |
| gene-LOC101894606 | 2097.241 | 3351.17 | -0.67617 | 8.61E-07 | 0.000146 | LOC101894606 | uncharacterized protein LOC101894606\|\|uncharacterized LOC101894606 |
| gene-LOC101894736 | 1884.427 | 1359.743 | 0.47079 | 0.00017 | 0.011789 | LOC101894736 | acidic leucine-rich nuclear phosphoprotein 32 family member A%2C transcript variant X1 |
| gene-LOC101894790 | 193.2051 | 315.6082 | -0.708 | 0.000391 | 0.022747 | LOC101894790 | putative mediator of RNA polymerase II transcription subunit 26 |
| gene-LOC101894797 | 1501.072 | 888.4737 | 0.75659 | 0.000713 | 0.034255 | LOC101894797 | uncharacterized LOC101894797\|\|uncharacterized protein LOC101894797 |
| gene-LOC101895135 | 96.34431 | 40.36561 | 1.2551 | 0.00014 | 0.010265 | LOC101895135 | uncharacterized LOC101895135\|\|uncharacterized protein LOC101895135 |
| gene-LOC101895287 | 147.5345 | 85.83015 | 0.7815 | 0.001021 | 0.043928 | LOC101895287 | putative folylpolyglutamate synthase |
| gene-LOC101895322 | 616.6082 | 327.1276 | 0.9145 | 3.44E-09 | 1.21E-06 | LOC101895322 | protein cutoff |
| gene-LOC101895356 | 6937.518 | 9315.819 | -0.42526 | 0.000311 | 0.019014 | LOC101895356 | UTP--glucose-1-phosphate uridylyltransferase isoform X2\|\|UTP--glucose-1-phosphate uridylyltransferase%2C transcript variant X2 |
| gene-LOC101895417 | 1247.1 | 803.3974 | 0.63439 | 2.27E-05 | 0.002305 | LOC101895417 | uncharacterized protein LOC101895417 isoform X1\|\|uncharacterized LOC101895417%2C transcript variant X1 |
| gene-LOC101895580 | 184.9411 | 296.2455 | -0.67973 | 0.000222 | 0.014636 | LOC101895580 | WD repeat-containing protein 43 |
| gene-LOC101895609 | 406.6086 | 256.6605 | 0.66378 | 6.23E-05 | 0.005287 | LOC101895609 | twinkle protein%2C mitochondrial |
| gene-LOC101895657 | 501.8876 | 846.7654 | -0.7546 | 1.35E-07 | 3.16E-05 | LOC101895657 | clusterin-associated protein 1 homolog |
| gene-LOC101895658 | 1434.347 | 2168.862 | -0.59654 | 4.21E-05 | 0.003856 | LOC101895658 | muscle M-line assembly protein unc-89%2C transcript variant X2 |
| gene-LOC101895698 | 1700.881 | 1025.946 | 0.72933 | 0.000679 | 0.033002 | LOC101895698 | acyl-CoA-binding protein homolog |
| gene-LOC101895722 | 453.567 | 308.6443 | 0.55537 | 0.000514 | 0.027648 | LOC101895722 | probable peroxisomal acyl-coenzyme A oxidase 1 |
| gene-LOC101895857 | 202.1227 | 113.6904 | 0.83012 | 0.000989 | 0.043013 | LOC101895857 | protein PFC0760c-like |

| | | | | | | | |
|---|---|---|---|---|---|---|---|
| gene-LOC101895876 | 111.3519 | 217.1926 | -0.96385 | 3.14E-06 | 0.000422 | LOC101895876 | phospholipase B1%2C membrane-associated |
| gene-LOC101895892 | 514.7954 | 797.3144 | -0.63115 | 8.91E-06 | 0.001057 | LOC101895892 | uncharacterized LOC101895892 |
| gene-LOC101895908 | 4.17256 | 22.81558 | -2.451 | 0.000514 | 0.027648 | LOC101895908 | pupal cuticle protein Edg-78E |
| gene-LOC101895911 | 3488.092 | 5171.415 | -0.56812 | 2.34E-06 | 0.000334 | LOC101895911 | zinc finger matrin-type protein CG9776 |
| gene-LOC101895918 | 16468.58 | 11158.34 | 0.56159 | 1.21E-06 | 0.000194 | LOC101895918 | circadian clock-controlled protein-like |
| gene-LOC101895958 | 842.3405 | 1411.906 | -0.74517 | 2.73E-08 | 8.31E-06 | LOC101895958 | dual 3'%2C5'-cyclic-AMP and -GMP phosphodiesterase 11%2C transcript variant X2\|\|dual 3'%2C5'-cyclic-AMP and -GMP phosphodiesterase 11 isoform X2 |
| gene-LOC101895965 | 436.0345 | 1448.162 | -1.7317 | 2.26E-33 | 1.82E-29 | LOC101895965 | partner of bursicon |
| gene-LOC101895976 | 1014.724 | 747.8871 | 0.44019 | 0.00111 | 0.046629 | LOC101895976 | uncharacterized protein LOC101895976\|\|uncharacterized LOC101895976 |
| gene-LOC101895994 | 115.5437 | 188.2736 | -0.70439 | 0.001192 | 0.04891 | LOC101895994 | ribosomal RNA small subunit methyltransferase NEP1 |
| gene-LOC101895996 | 5003.797 | 3719.615 | 0.42787 | 0.000314 | 0.019118 | LOC101895996 | protein scylla |
| gene-LOC101896013 | 276.0219 | 159.6516 | 0.78986 | 5.20E-05 | 0.004538 | LOC101896013 | LIRP isoform X1\|\|LIRP%2C transcript variant X1 |
| gene-LOC101896067 | 962.2358 | 1700.394 | -0.82141 | 0.000166 | 0.011694 | LOC101896067 | neurogenic locus notch homolog protein 1%2C transcript variant X1\|\|neurogenic locus notch homolog protein 1 isoform X1 |
| gene-LOC101896081 | 1201.041 | 717.8651 | 0.7425 | 3.54E-08 | 1.04E-05 | LOC101896081 | cytochrome P450 4e2 |
| gene-LOC101896165 | 498.2901 | 799.9541 | -0.68293 | 2.18E-06 | 0.000317 | LOC101896165 | Sporozoite P67 surface antigen |
| gene-LOC101896233 | 4132.301 | 2932.437 | 0.49485 | 3.71E-05 | 0.003456 | LOC101896233 | glucose-6-phosphate 1-dehydrogenase%2C transcript variant X2\|\|glucose-6-phosphate 1-dehydrogenase isoform X2 |
| gene-LOC101896294 | 850.6662 | 1242.793 | -0.54692 | 3.63E-05 | 0.003449 | LOC101896294 | uncharacterized LOC101896294%2C transcript variant X2\|\|uncharacterized protein LOC101896294 isoform X2 |
| gene-LOC101896306 | 155.2637 | 260.6507 | -0.7474 | 0.00014 | 0.010265 | LOC101896306 | LON peptidase N-terminal domain and RING finger protein 2 |

| | | | | | | | |
|---|---|---|---|---|---|---|---|
| gene-LOC101896362 | 256.6881 | 387.8166 | -0.59536 | 0.000489 | 0.026746 | LOC101896362 | neurochondrin homolog |
| gene-LOC101896384 | 936.8249 | 2031.612 | -1.1168 | 8.56E-18 | 1.06E-14 | LOC101896384 | histone-lysine N-methyltransferase%2C H3 lysine-79 specific%2C transcript variant X4\|\|histone-lysine N-methyltransferase%2C H3 lysine-79 specific isoform X3 |
| gene-LOC101896415 | 439.7547 | 284.0998 | 0.6303 | 0.000121 | 0.009042 | LOC101896415 | lysoplasmalogenase-like protein TMEM86A |
| gene-LOC101896442 | 23643.72 | 31234.83 | -0.4017 | 0.000973 | 0.04287 | LOC101896442 | farnesol dehydrogenase |
| gene-LOC101896469 | 7946.924 | 3867.156 | 1.0391 | 1.09E-07 | 2.79E-05 | LOC101896469 | probable cytochrome P450 313a4%2C transcript variant X2\|\|probable cytochrome P450 313a4 isoform X2 |
| gene-LOC101896501 | 10261.42 | 16717.92 | -0.70416 | 1.29E-09 | 4.97E-07 | LOC101896501 | heat shock protein 83 |
| gene-LOC101896517 | 349.0468 | 208.5273 | 0.74318 | 3.66E-05 | 0.003452 | LOC101896517 | chitooligosaccharidolytic beta-N-acetylglucosaminidase%2C transcript variant X5\|\|chitooligosaccharidolytic beta-N-acetylglucosaminidase |
| gene-LOC101896544 | 1594.423 | 2094.768 | -0.39376 | 0.001204 | 0.049167 | LOC101896544 | pyruvate dehydrogenase (acetyl-transferring) kinase%2C mitochondrial |
| gene-LOC101896559 | 295.7255 | 574.3182 | -0.95759 | 1.07E-09 | 4.22E-07 | LOC101896559 | phospholipase D2 |
| gene-LOC101896673 | 6263.983 | 8199.088 | -0.38838 | 0.00087 | 0.040077 | LOC101896673 | 2-oxoglutarate dehydrogenase-like%2C mitochondrial |
| gene-LOC101896690 | 240.8474 | 114.677 | 1.0705 | 1.32E-07 | 3.14E-05 | LOC101896690 | alkaline phosphatase 4%2C transcript variant X1\|\|alkaline phosphatase 4 isoform X1 |
| gene-LOC101896715 | 4188.998 | 5499.509 | -0.3927 | 0.000988 | 0.043013 | LOC101896715 | adenylosuccinate synthetase |
| gene-LOC101896755 | 258.3671 | 398.0083 | -0.62338 | 0.000115 | 0.008659 | LOC101896755 | glutamic acid-rich protein%2C transcript variant X7\|\|glutamic acid-rich protein isoform X1 |
| gene-LOC101896765 | 195.1811 | 423.5162 | -1.1176 | 1.26E-10 | 6.15E-08 | LOC101896765 | zinc finger protein jing isoform X2\|\|zinc finger protein jing%2C transcript variant X3 |
| gene-LOC101896814 | 387.9107 | 234.0699 | 0.72879 | 1.91E-05 | 0.001997 | LOC101896814 | frizzled-3 |
| gene-LOC101896822 | 1404.103 | 2243.453 | -0.67607 | 8.43E-08 | 2.27E-05 | LOC101896822 | calcium-binding mitochondrial carrier protein |

| | | | | | | | |
|---|---|---|---|---|---|---|---|
| | | | | | | | Aralar1%2C transcript variant X3\|\|calcium-binding mitochondrial carrier protein Aralar1 isoform X3 |
| gene-LOC101896861 | 227.7644 | 130.3204 | 0.80548 | 4.88E-05 | 0.004299 | LOC101896861 | T-cell activation inhibitor%2C mitochondrial |
| gene-LOC101896936 | 402.8271 | 613.4745 | -0.60684 | 4.86E-05 | 0.004299 | LOC101896936 | mesencephalic astrocyte-derived neurotrophic factor homolog |
| gene-LOC101896986 | 1492.827 | 1047.373 | 0.51127 | 9.10E-05 | 0.007061 | LOC101896986 | RNA-binding protein lark |
| gene-LOC101897025 | 1266.988 | 1948.11 | -0.62067 | 1.00E-06 | 0.000167 | LOC101897025 | GTP-binding protein 1 |
| gene-LOC101897046 | 4526.298 | 5983.471 | -0.40265 | 0.000664 | 0.032644 | LOC101897046 | protein ref(2)P |
| gene-LOC101897148 | 620.4747 | 866.0529 | -0.48108 | 0.00059 | 0.030429 | LOC101897148 | sulfide:quinone oxidoreductase%2C mitochondrial |
| gene-LOC101897194 | 180.9764 | 58.47305 | 1.63 | 8.49E-07 | 0.000146 | LOC101897194 | double-headed protease inhibitor%2C submandibular gland |
| gene-LOC101897216 | 684.2629 | 374.237 | 0.8706 | 0.000273 | 0.017049 | LOC101897216 | uncharacterized protein LOC101897216\|\|uncharacterized LOC101897216 |
| gene-LOC101897223 | 188.5153 | 55.30769 | 1.7691 | 7.48E-10 | 3.02E-07 | LOC101897223 | uncharacterized LOC101897223\|\|uncharacterized protein LOC101897223 |
| gene-LOC101897275 | 722.7501 | 452.9094 | 0.67427 | 4.43E-06 | 0.000562 | LOC101897275 | uncharacterized family 31 glucosidase KIAA1161 |
| gene-LOC101897317 | 347.4204 | 191.864 | 0.8566 | 1.57E-06 | 0.000241 | LOC101897317 | uncharacterized LOC101897317%2C transcript variant X1 |
| gene-LOC101897343 | 608.8687 | 848.2875 | -0.47842 | 0.000632 | 0.031571 | LOC101897343 | intraflagellar transport protein 56 |
| gene-LOC101897347 | 1361.795 | 2339.616 | -0.78076 | 4.98E-10 | 2.11E-07 | LOC101897347 | dnaJ protein homolog 1%2C transcript variant X2\|\|dnaJ protein homolog 1 |
| gene-LOC101897406 | 552.9218 | 313.4962 | 0.81863 | 1.89E-07 | 4.24E-05 | LOC101897406 | cytochrome b561 domain-containing protein 2-like\|\|cytochrome b561 domain-containing protein 2-like%2C transcript variant X2 |
| gene-LOC101897428 | 328.6245 | 177.6572 | 0.88734 | 3.89E-06 | 0.000502 | LOC101897428 | phosphatidylserine decarboxylase proenzyme%2C mitochondrial%2C transcript variant X2 |
| gene-LOC101897522 | 3288.92 | 4578.692 | -0.47732 | 7.32E-05 | 0.005936 | LOC101897522 | scavenger receptor class B member 1 |
| gene-LOC101897587 | 923.4515 | 608.3073 | 0.60224 | 0.000883 | 0.040591 | LOC101897587 | cytochrome P450 4e2-like |

| | | | | | | | |
|---|---|---|---|---|---|---|---|
| gene-LOC101897649 | 308.6232 | 444.9637 | -0.52784 | 0.001042 | 0.044453 | LOC101897649 | uncharacterized LOC101897649\|\|uncharacterized protein LOC101897649 |
| gene-LOC101897660 | 612.2523 | 86.03284 | 2.8312 | 8.26E-07 | 0.000143 | LOC101897660 | vasotab |
| gene-LOC101897674 | 130.7774 | 256.6116 | -0.97247 | 5.96E-07 | 0.00011 | LOC101897674 | zinc metalloproteinase nas-14 |
| gene-LOC101897717 | 215.3297 | 344.8227 | -0.67931 | 0.000114 | 0.008595 | LOC101897717 | uncharacterized protein LOC101897717\|\|uncharacterized LOC101897717 |
| gene-LOC101897719 | 540.7451 | 744.368 | -0.46107 | 0.001162 | 0.048291 | LOC101897719 | band 7 protein AGAP004871-like |
| gene-LOC101897739 | 619.6305 | 426.4899 | 0.5389 | 0.000339 | 0.020246 | LOC101897739 | FACT complex subunit Ssrp1 |
| gene-LOC101897767 | 919.0306 | 1438.865 | -0.64675 | 9.47E-07 | 0.000159 | LOC101897767 | protein disulfide-isomerase A6 homolog%2C transcript variant X2 |
| gene-LOC101897863 | 505.8201 | 842.0786 | -0.73533 | 2.59E-07 | 5.58E-05 | LOC101897863 | microsomal glutathione S-transferase 1 |
| gene-LOC101897926 | 4251.663 | 5745.101 | -0.43431 | 0.000219 | 0.014539 | LOC101897926 | multiple inositol polyphosphate phosphatase 1 |
| gene-LOC101897978 | 1678.138 | 2259.535 | -0.42916 | 0.000697 | 0.033775 | LOC101897978 | serine/threonine-protein kinase PITSLRE%2C transcript variant X2\|\|serine/threonine-protein kinase PITSLRE isoform X1 |
| gene-LOC101897984 | 558.7945 | 829.1539 | -0.56932 | 5.27E-05 | 0.004569 | LOC101897984 | zinc transporter 2%2C transcript variant X4 |
| gene-LOC101898011 | 932.2134 | 1269.526 | -0.44556 | 0.00101 | 0.043665 | LOC101898011 | probable cytochrome P450 28c1 |
| gene-LOC101898055 | 59.03929 | 126.4071 | -1.0983 | 3.05E-05 | 0.003003 | LOC101898055 | adenylate cyclase type 8%2C transcript variant X1 |
| gene-LOC101898132 | 1433.244 | 2486.682 | -0.79494 | 2.55E-10 | 1.11E-07 | LOC101898132 | fatty acyl-CoA reductase wat |
| gene-LOC101898153 | 1974.975 | 2904.973 | -0.55669 | 6.87E-06 | 0.000846 | LOC101898153 | carboxypeptidase B |
| gene-LOC101898193 | 968.8011 | 670.1101 | 0.5318 | 0.000146 | 0.010622 | LOC101898193 | selenocysteine methyltransferase%2C transcript variant X1\|\|selenocysteine methyltransferase isoform X1 |
| gene-LOC101898279 | 127.7306 | 264.382 | -1.0495 | 1.32E-07 | 3.14E-05 | LOC101898279 | putative inorganic phosphate cotransporter |
| gene-LOC101898280 | 7.644622 | 54.24338 | -2.8269 | 6.67E-07 | 0.000119 | LOC101898280 | major heat shock 70 kDa protein Ba-like |
| gene-LOC101898324 | 9346.033 | 6422.666 | 0.54118 | 2.82E-06 | 0.000387 | LOC101898324 | very long-chain-fatty-acid--CoA ligase bubblegum |
| gene-LOC101898389 | 1149.976 | 766.2601 | 0.5857 | 1.48E-05 | 0.001625 | LOC101898389 | L-galactose dehydrogenase |
| gene-LOC101898439 | 270.1745 | 411.7971 | -0.60804 | 0.000278 | 0.017306 | LOC101898439 | probable ATP-dependent RNA helicase pitchoune |

| | | | | | | | |
|---|---|---|---|---|---|---|---|
| gene-LOC101898458 | 2368.101 | 3199.916 | -0.4343 | 0.000449 | 0.025298 | LOC101898458 | ethanolamine kinase |
| gene-LOC101898467 | 1251.693 | 1751.689 | -0.48487 | 0.000173 | 0.01195 | LOC101898467 | mesocentin\|\|mesocentin%2C transcript variant X3 |
| gene-LOC101898520 | 4023.918 | 5813.822 | -0.53089 | 7.53E-06 | 0.00091 | LOC101898520 | putative phospholipase B-like lamina ancestor |
| gene-LOC101898526 | 3217.972 | 6471.198 | -1.0079 | 3.35E-17 | 3.87E-14 | LOC101898526 | esterase B1%2C transcript variant X2\|\|esterase B1 |
| gene-LOC101898545 | 169.4513 | 100.872 | 0.74835 | 0.000947 | 0.041985 | LOC101898545 | double-strand break repair protein MRE11 |
| gene-LOC101898640 | 2703.83 | 1614.437 | 0.74397 | 3.32E-09 | 1.19E-06 | LOC101898640 | ecdysteroid-regulated 16 kDa protein |
| gene-LOC101898644 | 323.9984 | 852.3196 | -1.3954 | 1.63E-20 | 2.63E-17 | LOC101898644 | purine nucleoside phosphorylase%2C transcript variant X3\|\|purine nucleoside phosphorylase isoform X3 |
| gene-LOC101898663 | 3511.534 | 7855.968 | -1.1617 | 2.46E-22 | 5.67E-19 | LOC101898663 | carnitine O-palmitoyltransferase 1%2C liver isoform%2C transcript variant X2 |
| gene-LOC101898711 | 1539.98 | 2230.798 | -0.53465 | 0.000457 | 0.025489 | LOC101898711 | elongation of very long chain fatty acids protein AAEL008004%2C transcript variant X1 |
| gene-LOC101898731 | 16.91496 | 70.03648 | -2.0498 | 8.80E-08 | 2.33E-05 | LOC101898731 | uncharacterized protein LOC101898731 isoform X5\|\|uncharacterized LOC101898731%2C transcript variant X5 |
| gene-LOC101898770 | 2.922895 | 48.98139 | -4.0668 | 2.38E-12 | 1.54E-09 | LOC101898770 | venom allergen 5 |
| gene-LOC101899102 | 173.4493 | 94.68767 | 0.87327 | 0.00016 | 0.011326 | LOC101899102 | parkin coregulated gene protein homolog |
| gene-LOC101899135 | 212.703 | 78.00159 | 1.4473 | 1.46E-10 | 6.94E-08 | LOC101899135 | cytochrome P450 6d1-like |
| gene-LOC101899158 | 298.5451 | 499.6956 | -0.7431 | 2.60E-06 | 0.000368 | LOC101899158 | ATP-binding cassette sub-family G member 5 |
| gene-LOC101899169 | 2859.425 | 4074.931 | -0.51105 | 2.13E-05 | 0.002207 | LOC101899169 | endoplasmin homolog |
| gene-LOC101899230 | 8898.635 | 6410.903 | 0.47306 | 0.00089 | 0.0408 | LOC101899230 | ATP-binding cassette sub-family A member 12%2C transcript variant X2\|\|ATP-binding cassette sub-family A member 12 |
| gene-LOC101899240 | 333.3438 | 483.5544 | -0.53667 | 0.000623 | 0.031392 | LOC101899240 | carbohydrate sulfotransferase 13 isoform X1\|\|carbohydrate sulfotransferase 13%2C transcript variant X1 |
| gene-LOC101899314 | 487.6764 | 294.3821 | 0.72823 | 8.18E-06 | 0.000977 | LOC101899314 | integumentary mucin A.1 |

| | | | | | | | |
|---|---|---|---|---|---|---|---|
| gene-LOC101899329 | 923.4752 | 571.5986 | 0.69207 | 9.33E-06 | 0.001091 | LOC101899329 | tissue factor pathway inhibitor%2C transcript variant X2\|\|tissue factor pathway inhibitor |
| gene-LOC101899370 | 688.8 | 1029.809 | -0.58022 | 2.16E-05 | 0.002208 | LOC101899370 | stress-induced-phosphoprotein 1 |
| gene-LOC101899381 | 1042.704 | 1590.407 | -0.60907 | 8.87E-05 | 0.006983 | LOC101899381 | uncharacterized protein LOC101899381\|\|uncharacterized LOC101899381 |
| gene-LOC101899427 | 198.7078 | 309.0061 | -0.63699 | 0.000614 | 0.031215 | LOC101899427 | uncharacterized protein LOC101899427\|\|Zona pellucida-like domain protein |
| gene-LOC101899434 | 273.8865 | 439.5219 | -0.68236 | 4.27E-05 | 0.003893 | LOC101899434 | probable cytochrome P450 6t3 |
| gene-LOC101899573 | 215.5315 | 349.188 | -0.69611 | 0.000272 | 0.017049 | LOC101899573 | ionotropic receptor 21a |
| gene-LOC101899613 | 296.2084 | 448.1939 | -0.59751 | 0.000331 | 0.019938 | LOC101899613 | GTP-binding protein 1\|\|GTP-binding protein 1%2C transcript variant X2 |
| gene-LOC101899617 | 3107.041 | 4740.197 | -0.60941 | 4.09E-07 | 8.05E-05 | LOC101899617 | choline/ethanolamine kinase isoform X2\|\|choline/ethanolamine kinase%2C transcript variant X2 |
| gene-LOC101899620 | 4762.629 | 6346.698 | -0.41425 | 0.00053 | 0.028053 | LOC101899620 | protein krasavietz%2C transcript variant X1\|\|protein krasavietz |
| gene-LOC101899666 | 179.8418 | 65.14333 | 1.465 | 5.95E-10 | 2.46E-07 | LOC101899666 | maltase A3-like |
| gene-LOC101899684 | 597.4224 | 377.374 | 0.66276 | 4.64E-05 | 0.004154 | LOC101899684 | vitellogenin-1-like |
| gene-LOC101899700 | 440.4461 | 652.0529 | -0.56602 | 0.00017 | 0.011789 | LOC101899700 | L-lactate dehydrogenase |
| gene-LOC101899715 | 215.1249 | 109.9778 | 0.96796 | 6.33E-05 | 0.005314 | LOC101899715 | ribosome-recycling factor%2C mitochondrial |
| gene-LOC101899769 | 115.4302 | 200.9915 | -0.80011 | 0.00119 | 0.04891 | LOC101899769 | protocadherin-15%2C transcript variant X2 |
| gene-LOC101899804 | 1787.886 | 2909.623 | -0.70258 | 0.000568 | 0.029513 | LOC101899804 | farnesol dehydrogenase |
| gene-LOC101899870 | 56.00684 | 23.20093 | 1.2714 | 0.001169 | 0.048342 | LOC101899870 | uncharacterized protein LOC101899870\|\|pseudouridine kinase |
| gene-LOC101899878 | 1790.085 | 3045.876 | -0.76683 | 1.06E-06 | 0.000174 | LOC101899878 | titin%2C transcript variant X1 |
| gene-LOC101899903 | 5231.045 | 3428.189 | 0.60965 | 2.34E-07 | 5.17E-05 | LOC101899903 | flagellar radial spoke protein 2 |
| gene-LOC101899963 | 601.4574 | 929.8041 | -0.62846 | 6.38E-06 | 0.000791 | LOC101899963 | protein late bloomer |
| gene-LOC101899983 | 13.87206 | 39.54599 | -1.5113 | 0.000863 | 0.039872 | LOC101899983 | pancreatic triacylglycerol lipase%2C transcript variant |

| | | | | | | | |
|---|---|---|---|---|---|---|---|
| | | | | | | | X1\|\|pancreatic triacylglycerol lipase isoform X1 |
| gene-LOC101900065 | 124.7529 | 39.10539 | 1.6736 | 2.83E-06 | 0.000387 | LOC101900065 | probable cytochrome P450 6a14\|\|LOW QUALITY PROTEIN: probable cytochrome P450 6a14 |
| gene-LOC101900080 | 838.2519 | 566.7877 | 0.56458 | 5.41E-05 | 0.00467 | LOC101900080 | hyccin |
| gene-LOC101900132 | 101.4236 | 53.34525 | 0.92696 | 0.001014 | 0.043732 | LOC101900132 | uncharacterized protein LOC101900132\|\|uncharacterized LOC101900132 |
| gene-LOC101900224 | 3891.822 | 5300.357 | -0.44564 | 0.000167 | 0.011694 | LOC101900224 | uncharacterized LOC101900224%2C transcript variant X5 |
| gene-LOC101900234 | 207.7389 | 317.0643 | -0.61 | 0.000814 | 0.037928 | LOC101900234 | kinesin-like protein Nod |
| gene-LOC101900252 | 9099.015 | 13593.66 | -0.57915 | 5.33E-07 | 0.000101 | LOC101900252 | tensin |
| gene-LOC101900253 | 395.9838 | 255.3459 | 0.63299 | 0.000184 | 0.012573 | LOC101900253 | UNC93-like protein%2C transcript variant X2 |
| gene-LOC101900256 | 1404.424 | 2017.976 | -0.52293 | 0.000913 | 0.041098 | LOC101900256 | protein tyrosine phosphatase type IVA 1 |
| gene-LOC101900259 | 994.9222 | 1360.153 | -0.45111 | 0.00098 | 0.043013 | LOC101900259 | UPF0389 protein CG9231 |
| gene-LOC101900280 | 70.81532 | 167.0299 | -1.238 | 0.000168 | 0.011749 | LOC101900280 | adult cuticle protein 1 |
| gene-LOC101900422 | 109.6964 | 224.183 | -1.0312 | 9.22E-06 | 0.001086 | LOC101900422 | serine/threonine-protein phosphatase 2B catalytic subunit 1 isoform X2\|\|serine/threonine-protein phosphatase 2B catalytic subunit 1%2C transcript variant X2 |
| gene-LOC101900442 | 114.786 | 56.8232 | 1.0144 | 0.000634 | 0.031584 | LOC101900442 | probable proline--tRNA ligase%2C mitochondrial%2C transcript variant X1 |
| gene-LOC101900472 | 304.7244 | 515.8309 | -0.75939 | 2.09E-06 | 0.00031 | LOC101900472 | chloride channel protein 2 isoform X4\|\|chloride channel protein 2%2C transcript variant X5 |
| gene-LOC101900502 | 1147.3 | 805.8862 | 0.50959 | 0.000125 | 0.009263 | LOC101900502 | uncharacterized protein LOC101900502\|\|uncharacterized LOC101900502 |
| gene-LOC101900674 | 349.6545 | 230.5631 | 0.60077 | 0.000597 | 0.030677 | LOC101900674 | neuferricin homolog |
| gene-LOC101900692 | 357.8147 | 238.4199 | 0.58571 | 0.000947 | 0.041985 | LOC101900692 | alpha-tocopherol transfer protein-like |
| gene-LOC101900724 | 27.5026 | 4.230256 | 2.7008 | 3.75E-05 | 0.003479 | LOC101900724 | cytochrome P450 4d1-like |
| gene-LOC101900736 | 193.7904 | 302.1371 | -0.64071 | 0.000479 | 0.026369 | LOC101900736 | serine palmitoyltransferase 2 isoform X1\|\|serine |

| | | | | | | | |
|---|---|---|---|---|---|---|---|
| | | | | | | | palmitoyltransferase 2%2C transcript variant X1 |
| gene-LOC101900747 | 237.9504 | 137.2756 | 0.79359 | 0.000798 | 0.037302 | LOC101900747 | alpha-tocopherol transfer protein |
| gene-LOC101900815 | 173.1071 | 378.4447 | -1.1284 | 2.53E-10 | 1.11E-07 | LOC101900815 | F-box only protein 32 |
| gene-LOC101900874 | 156.9321 | 294.8715 | -0.90995 | 1.47E-06 | 0.000231 | LOC101900874 | uncharacterized protein LOC101900874\|\|uncharacterized LOC101900874 |
| gene-LOC101901071 | 835.4228 | 1193.124 | -0.51417 | 0.000143 | 0.010463 | LOC101901071 | ELMO domain-containing protein 2%2C transcript variant X4 |
| gene-LOC101901086 | 2347.498 | 1688.215 | 0.47563 | 0.000113 | 0.008572 | LOC101901086 | uncharacterized LOC101901086\|\|uncharacterized protein LOC101901086 |
| gene-LOC101901112 | 394.0875 | 561.3483 | -0.51038 | 0.000984 | 0.043013 | LOC101901112 | lipase member H-B |
| gene-LOC101901151 | 35.64721 | 2.362974 | 3.9151 | 3.96E-09 | 1.36E-06 | LOC101901151 | nose resistant to fluoxetine protein 6 |
| gene-LOC101901231 | 805.163 | 1554.768 | -0.94935 | 0.000149 | 0.010788 | LOC101901231 | hypothetical protein\|\|uncharacterized protein LOC101901231 |
| gene-LOC101901237 | 1626.186 | 2209.662 | -0.44233 | 0.0005 | 0.027224 | LOC101901237 | heat shock factor protein isoform X1\|\|heat shock factor protein%2C transcript variant X5 |
| gene-LOC101901243 | 939.7371 | 1357.942 | -0.53109 | 7.00E-05 | 0.005792 | LOC101901243 | ubiquitin-conjugating enzyme E2 Q2%2C transcript variant X3 |
| gene-LOC101901262 | 1611.998 | 2214.844 | -0.45836 | 0.000257 | 0.016369 | LOC101901262 | sialin |
| gene-LOC101901296 | 3199.036 | 4495.367 | -0.4908 | 4.44E-05 | 0.004001 | LOC101901296 | aminopeptidase N-like |
| gene-LOC101901311 | 2489.964 | 4578.449 | -0.87873 | 5.04E-13 | 3.53E-10 | LOC101901311 | proline dehydrogenase 1%2C mitochondrial |
| gene-LOC101901393 | 494.7502 | 192.4486 | 1.3622 | 9.43E-16 | 8.45E-13 | LOC101901393 | uncharacterized LOC101901393%2C transcript variant X1 |
| gene-LOC101901407 | 1998.122 | 3163.346 | -0.66281 | 7.19E-08 | 2.00E-05 | LOC101901407 | hypoxia up-regulated protein 1 |
| gene-LOC101901572 | 1996.433 | 1228.128 | 0.70096 | 0.00039 | 0.022747 | LOC101901572 | uncharacterized threonine-rich GPI-anchored glycoprotein PJ4664.02 |
| gene-LOC101901600 | 3898.504 | 2459.915 | 0.66431 | 1.32E-07 | 3.14E-05 | LOC101901600 | dynein light chain 2%2C cytoplasmic%2C transcript variant X1 |
| gene-LOC101901705 | 1642.443 | 2659.691 | -0.69541 | 2.03E-06 | 0.000307 | LOC101901705 | excitatory amino acid transporter isoform |

| | | | | | | | |
|---|---|---|---|---|---|---|---|
| | | | | | | | X1\|\|excitatory amino acid transporter%2C transcript variant X1 |
| gene-LOC101901767 | 37.45815 | 87.70893 | -1.2274 | 7.92E-05 | 0.006346 | LOC101901767 | proton-associated sugar transporter A |
| gene-LOC101901806 | 220.2785 | 352.6367 | -0.67885 | 8.98E-05 | 0.007 | LOC101901806 | putative sodium-dependent multivitamin transporter |
| gene-LOC101901815 | 127.3102 | 68.81574 | 0.88754 | 0.000796 | 0.037302 | LOC101901815 | uncharacterized LOC101901815\|\|uncharacterized protein LOC101901815 |
| gene-LOC101901820 | 1.234381 | 15.19148 | -3.6214 | 0.000703 | 0.033952 | LOC101901820 | larval cuticle protein 5-like isoform X1\|\|larval cuticle protein 5-like%2C transcript variant X1 |
| gene-LOC101901826 | 1296.319 | 1981.833 | -0.61241 | 1.94E-06 | 0.000295 | LOC101901826 | uncharacterized LOC101901826\|\|uncharacterized protein LOC101901826 |
| gene-LOC105261419 | 3661.995 | 2385.893 | 0.6181 | 2.63E-07 | 5.59E-05 | LOC105261419 | high mobility group protein D%2C transcript variant X1 |
| gene-LOC105261447 | 1071.04 | 1487.058 | -0.47345 | 0.000326 | 0.019667 | LOC105261447 | helicase domino |
| gene-LOC105261583 | 1255.848 | 331.7024 | 1.9207 | 1.17E-21 | 2.37E-18 | LOC105261583 | uncharacterized LOC105261583%2C transcript variant X2\|\|uncharacterized protein LOC105261583 isoform X2 |
| gene-LOC105261589 | 245.5875 | 155.0461 | 0.66354 | 0.00065 | 0.032165 | LOC105261589 | uncharacterized protein LOC105261589\|\|uncharacterized LOC105261589 |
| gene-LOC105261744 | 3342.229 | 4415.892 | -0.40189 | 0.000734 | 0.034767 | LOC105261744 | hexokinase type 2 |
| gene-LOC105261803 | 11206.99 | 8095.03 | 0.46929 | 0.000989 | 0.043013 | LOC105261803 | uncharacterized LOC105261803\|\|uncharacterized protein LOC105261803 |
| gene-LOC105261829 | 2317.701 | 1254.226 | 0.8859 | 1.50E-12 | 1.01E-09 | LOC105261829 | cytochrome P450 6A1-like |
| gene-LOC105261914 | 322.0876 | 110.603 | 1.5421 | 2.81E-15 | 2.39E-12 | LOC105261914 | general odorant-binding protein 56h-like |
| gene-LOC105261927 | 404.0688 | 94.30716 | 2.0992 | 7.75E-28 | 2.50E-24 | LOC105261927 | farnesyl pyrophosphate synthase |
| gene-LOC105262082 | 16.08477 | 0 | Inf | 1.10E-06 | 0.000179 | LOC105262082 | LOW QUALITY PROTEIN: uncharacterized protein LOC105262082\|\|uncharacterized LOC105262082 |
| gene-LOC105262153 | 48.78821 | 19.38903 | 1.3313 | 0.001134 | 0.047385 | LOC105262153 | putative protein tag-52 |
| gene-LOC105262272 | 85.27771 | 7.975278 | 3.4186 | 9.29E-17 | 8.82E-14 | LOC105262272 | lectin subunit alpha-like |

| | | | | | | | |
|---|---|---|---|---|---|---|---|
| gene-LOC105262305 | 3188.717 | 5146.448 | -0.6906 | 2.08E-06 | 0.00031 | LOC105262305 | uncharacterized protein LOC105262305 isoform X2\|\|uncharacterized LOC105262305%2C transcript variant X2 |
| gene-LOC105262391 | 221.5931 | 42.48375 | 2.3829 | 6.61E-17 | 6.66E-14 | LOC105262391 | turripeptide Pal9.2-like |
| gene-LOC105262393 | 32.35719 | 2.56361 | 3.6578 | 1.04E-07 | 2.70E-05 | LOC105262393 | uncharacterized protein LOC105262393\|\|uncharacterized LOC105262393 |
| gene-LOC105262450 | 240.9986 | 383.0837 | -0.66863 | 9.18E-05 | 0.007088 | LOC105262450 | uncharacterized protein LOC105262450\|\|uncharacterized LOC105262450%2C transcript variant X1 |
| gene-LOC105262478 | 1906.695 | 2762.892 | -0.53511 | 0.000557 | 0.02915 | LOC105262478 | nucleic-acid-binding protein from mobile element jockey\|\|uncharacterized protein LOC105262478 |
| gene-LOC109611735 | 6.184764 | 28.31136 | -2.1946 | 0.000297 | 0.0182 | LOC109611735 | uncharacterized LOC109611735\|\|uncharacterized protein LOC109611735 |
| gene-LOC109611747 | 123.7585 | 56.67854 | 1.1267 | 2.07E-05 | 0.002153 | LOC109611747 | protein spaetzle 5-like\|\|protein spaetzle 5-like%2C transcript variant X2 |
| gene-LOC109612301 | 213.1578 | 330.0458 | -0.63074 | 0.000611 | 0.031205 | LOC109612301 | max-binding protein MNT-like |
| gene-LOC109613297 | 218.2967 | 121.2905 | 0.84782 | 3.45E-05 | 0.00331 | LOC109613297 | hexokinase type 2-like |
| gene-LOC109613447 | 397.5786 | 227.839 | 0.80323 | 0.00042 | 0.023789 | LOC109613447 | uncharacterized protein LOC109613447\|\|uncharacterized LOC109613447 |
| gene-LOC109613802 | 572.3346 | 798.067 | -0.47965 | 0.000842 | 0.03905 | LOC109613802 | uncharacterized LOC109613802 |
| gene-LOC109613852 | 140.0823 | 276.9291 | -0.98324 | 5.43E-07 | 0.000102 | LOC109613852 | von Willebrand factor D and EGF domain-containing protein |
| gene-LOC109613860 | 126.5936 | 227.7347 | -0.84715 | 0.00027 | 0.017049 | LOC109613860 | uncharacterized LOC109613860\|\|uncharacterized protein LOC109613860 |
| gene-LOC109613986 | 1105.57 | 791.2175 | 0.48264 | 0.000347 | 0.020616 | LOC109613986 | uncharacterized LOC109613986 |
| gene-LOC109614129 | 41.32639 | 86.20918 | -1.0608 | 0.000509 | 0.027648 | LOC109614129 | uncharacterized LOC109614129 |
| gene-LOC109614175 | 521.8618 | 153.9478 | 1.7612 | 2.59E-09 | 9.51E-07 | LOC109614175 | uncharacterized LOC109614175\|\|uncharacterized protein LOC109614175 |

| | | | | | | | |
|---|---|---|---|---|---|---|---|
| gene-LOC109614213 | 44.56404 | 16.892 | 1.3995 | 0.001168 | 0.048342 | LOC109614213 | uncharacterized LOC109614213 |
| gene-LOC109614258 | 53.33515 | 125.1758 | -1.2308 | 5.28E-06 | 0.000665 | LOC109614258 | uncharacterized LOC109614258 |
| gene-defensin-1 | 590.9002 | 120.1658 | 2.2979 | 0.000393 | 0.022784 | defensin-1 | phormicin-like\|\|uncharacterized protein LOC101887540 |
