# Supplementary material for: Short-term particulate matter contamination severely compromises insect antennal olfactory perception
Source: Nat Commun. 2023 Jul 11;14:4112. doi: 10.1038/s41467-023-39469-3 (PMC10336072; doi:10.1038/s41467-023-39469-3)
Supplement: Supplementary file 5 — Supplementary Data 2 [file 41467_2023_39469_MOESM5_ESM.pdf]

Supplementary Data 2. Details of differential expressed genes (DEGs) between antennae of uncontaminated and contaminated male houseflies collected in spring. Readcount is the average read count of each gen, log2FoldChange is calculated by formular log2(readcount_CMA/readcount_UMA), pval is the *p* value, padj is the fdr corrected *p* value using Benjamini/Hochberg method. All *p*-values are based on two-sided tests. CMA: contaminated male antennae, UMA: uncontaminated male antennae.

**Gene_id**	**readcount_CMA**	**readcount_UMA**	**log2FoldChange**	**pval**	**padj**	**gene_name**	**description**
Novel00983	133.5351	340.7551	-1.3515	4.71E-06	0.002175	-	-
Novel01663	75.60713	271.1517	-1.8425	5.87E-14	4.74E-10	-	-
Novel01969	24.89299	70.30523	-1.4979	0.000219	0.04591	-	-
gene-CYP6D8	1433.515	3470.511	-1.2756	1.83E-05	0.006899	CYP6D8	probable cytochrome P450 6d5-like
gene-LOC101887475	71.12814	21.21828	1.7451	1.91E-05	0.007019	LOC101887475	farnesyl pyrophosphate synthase-like
gene-LOC101887522	146.9218	60.38857	1.2827	6.47E-06	0.002751	LOC101887522	nuclear receptor corepressor 2
gene-LOC101887828	286.0006	503.4456	-0.81582	2.00E-05	0.007203	LOC101887828	F-box only protein 28%2C transcript variant X3||F-box only protein 28 isoform X3
gene-LOC101887978	119.4878	296.3497	-1.3104	1.04E-08	1.68E-05	LOC101887978	protein OPI10 homolog
gene-LOC101888348	1309.703	2031.85	-0.63355	4.44E-05	0.014081	LOC101888348	hypothetical protein%2C transcript variant X2||uncharacterized protein LOC101888348 isoform X2
gene-LOC101888467	15.3547	63.42879	-2.0465	5.98E-06	0.002613	LOC101888467	alpha-methyldopa hypersensitive protein
gene-LOC101888799	38.00021	167.7738	-2.1424	4.06E-05	0.01366	LOC101888799	phosphoenolpyruvate carboxykinase [GTP]
gene-LOC101888919	77.99366	20.41282	1.9339	3.57E-06	0.001988	LOC101888919	larval cuticle protein 8
gene-LOC101889374	3457.835	2002.089	0.78836	1.52E-07	0.000143	LOC101889374	dynein beta chain%2C ciliary
gene-LOC101889838	2713.452	1844.567	0.55685	0.000227	0.047079	LOC101889838	murinoglobulin-2%2C transcript variant X3||murinoglobulin-2 isoform X3
gene-LOC101889844	61.88433	139.1976	-1.1695	9.59E-05	0.025827	LOC101889844	elongation of very long chain fatty acids protein 7%2C transcript variant X1||elongation of very long chain fatty acids protein 7
gene-LOC101890440	98.40305	32.08187	1.6169	3.86E-06	0.001988	LOC101890440	lipase 3-like
gene-LOC101890607	1417.933	745.3969	0.92771	1.02E-08	1.68E-05	LOC101890607	lipase 3-like
gene-LOC101890854	2262.856	1510.283	0.58333	0.00016	0.036036	LOC101890854	glucose dehydrogenase [FAD%2C quinone]

gene-LOC101890896	61.05515	8.5828	2.8306	1.40E-08	1.89E-05	LOC101890896	peptidoglycan-recognition protein SA-like
gene-LOC101891486	1229.98	2356.631	-0.93809	1.45E-09	2.93E-06	LOC101891486	uncharacterized protein LOC101891486||uncharacterized LOC101891486
gene-LOC101891572	614.5047	959.1787	-0.64238	0.000162	0.036036	LOC101891572	membrane alanyl aminopeptidase
gene-LOC101891684	5730.264	10207.23	-0.83292	1.40E-08	1.89E-05	LOC101891684	proton-coupled amino acid transporter 2
gene-LOC101891759	1719.219	908.4507	0.92027	8.47E-06	0.003422	LOC101891759	probable cytochrome P450 4d14
gene-LOC101891996	108.2609	235.1102	-1.1188	3.87E-06	0.001988	LOC101891996	venom acid phosphatase Acph-1
gene-LOC101891997	762.7584	327.3872	1.2202	0.000154	0.036036	LOC101891997	general odorant-binding protein 56h
gene-LOC101892159	302.4076	157.2431	0.9435	7.64E-05	0.021307	LOC101892159	probable RNA helicase armi
gene-LOC101892265	114.6319	268.3705	-1.2272	1.80E-07	0.000145	LOC101892265	transcription factor Adf-1
gene-LOC101892323	92.2972	235.3462	-1.3504	5.76E-08	6.20E-05	LOC101892323	nucleoside diphosphate kinase 7
gene-LOC101892630	12.71143	49.00098	-1.9467	7.16E-05	0.020319	LOC101892630	uncharacterized LOC101892630%2C transcript variant X2||uncharacterized protein LOC101892630 isoform X2
gene-LOC101892684	111.6239	48.39453	1.2057	0.000203	0.043188	LOC101892684	EF-hand calcium-binding domain-containing protein 1
gene-LOC101892760	2325.13	1332.415	0.80327	1.90E-07	0.000147	LOC101892760	protein FAM188B2
gene-LOC101892829	566.5641	298.2531	0.9257	4.06E-06	0.001988	LOC101892829	peptide methionine sulfoxide reductase||LOW QUALITY PROTEIN: peptide methionine sulfoxide reductase
gene-LOC101893184	127.5855	54.11361	1.2374	3.39E-05	0.011672	LOC101893184	peptidoglycan-recognition protein SB1 precursor||peptidoglycan-recognition protein SB1
gene-LOC101893361	90.25747	175.6611	-0.96068	0.0002	0.043022	LOC101893361	acyl-CoA synthetase family member 2%2C mitochondrial
gene-LOC101893628	234.8054	108.9126	1.1083	3.10E-06	0.001791	LOC101893628	pancreatic lipase-related protein 2
gene-LOC101894001	536.7598	201.773	1.4115	5.36E-12	2.17E-08	LOC101894001	probable asparagine synthetase [glutamine-hydrolyzing]
gene-LOC101894147	427.2666	881.2854	-1.0445	1.24E-09	2.86E-06	LOC101894147	transcription factor Adf-1
gene-LOC101894235	30.89586	87.63281	-1.5041	7.00E-05	0.020199	LOC101894235	uncharacterized protein LOC101894235||Chitin

							binding protein
gene-LOC101894425	735.6978	444.281	0.72764	5.10E-05	0.015305	LOC101894425	cytochrome P450 4d8%2C transcript variant X2||cytochrome P450 4d8 isoform X2
gene-LOC101894603	90.49587	35.27809	1.3591	0.000186	0.040612	LOC101894603	protein still life%2C isoforms C/SIF type 2
gene-LOC101895121	1036.962	640.9333	0.69412	0.000143	0.034849	LOC101895121	esterase B1-like
gene-LOC101895965	191.3794	1222.151	-2.6749	3.29E-49	5.32E-45	LOC101895965	partner of bursicon
gene-LOC101896469	391.9876	231.1796	0.76179	0.000116	0.030275	LOC101896469	probable cytochrome P450 313a4%2C transcript variant X2||probable cytochrome P450 313a4 isoform X2
gene-LOC101896501	10589.15	20373.66	-0.94412	7.25E-11	1.95E-07	LOC101896501	heat shock protein 83
gene-LOC101896676	1304.443	767.8418	0.76455	4.60E-06	0.002175	LOC101896676	glycine-rich cell wall structural protein
gene-LOC101896690	238.3936	91.58144	1.3802	2.57E-08	3.10E-05	LOC101896690	alkaline phosphatase 4%2C transcript variant X1||alkaline phosphatase 4 isoform X1
gene-LOC101896765	149.0686	296.574	-0.99242	7.60E-06	0.00315	LOC101896765	zinc finger protein jing isoform X2||zinc finger protein jing%2C transcript variant X3
gene-LOC101896897	29.713	4.199663	2.8227	5.11E-05	0.015305	LOC101896897	diptericin-D-like
gene-LOC101897064	228.3544	123.3979	0.88796	0.000163	0.036036	LOC101897064	growth arrest and DNA damage-inducible protein GADD45 alpha
gene-LOC101897095	869.977	435.4729	0.99839	1.15E-06	0.000743	LOC101897095	-
gene-LOC101897194	145.9097	49.60792	1.5564	1.77E-07	0.000145	LOC101897194	double-headed protease inhibitor%2C submandibular gland
gene-LOC101897223	132.657	45.73079	1.5365	3.56E-07	0.000262	LOC101897223	uncharacterized LOC101897223||uncharacterized protein LOC101897223
gene-LOC101897347	1371.473	2123.041	-0.63041	4.58E-05	0.01423	LOC101897347	dnaJ protein homolog 1%2C transcript variant X2||dnaJ protein homolog 1
gene-LOC101897373	47.13483	11.0446	2.0935	4.41E-05	0.014081	LOC101897373	double-headed protease inhibitor%2C submandibular gland-like
gene-LOC101897406	483.869	290.1412	0.73786	0.00016	0.036036	LOC101897406	cytochrome b561 domain-containing protein 2-

							like‖cytochrome b561 domain-containing protein 2-like%2C transcript variant X2
gene-LOC101897453	76.65491	18.22299	2.0726	8.35E-07	0.000563	LOC101897453	arginine/serine-rich coiled-coil protein 2-like
gene-LOC101897488	103.1226	17.96004	2.5215	8.14E-05	0.022295	LOC101897488	vasotab-like
gene-LOC101897542	468.3662	284.1276	0.7211	0.00023	0.047079	LOC101897542	sodium-independent sulfate anion transporter isoform X1‖sodium-independent sulfate anion transporter%2C transcript variant X1
gene-LOC101897560	880.2383	1357.674	-0.62517	0.000119	0.030447	LOC101897560	protein boule%2C transcript variant X1
gene-LOC101897660	527.9993	94.91478	2.4758	2.13E-13	1.15E-09	LOC101897660	vasotab
gene-LOC101897678	778.6336	1236.346	-0.66707	5.49E-05	0.016142	LOC101897678	solute carrier family 25 member 36 isoform X2‖solute carrier family 25 member 36%2C transcript variant X4
gene-LOC101897731	167.2045	82.5747	1.0178	0.000158	0.036036	LOC101897731	phospholipase B1%2C membrane-associated-like
gene-LOC101898324	7441.769	5041.689	0.56174	0.000144	0.034849	LOC101898324	very long-chain-fatty-acid--CoA ligase bubblegum
gene-LOC101898526	30658.08	44900.06	-0.55045	0.000107	0.028254	LOC101898526	esterase B1%2C transcript variant X2‖esterase B1
gene-LOC101898644	351.661	618.5677	-0.81475	9.78E-06	0.003854	LOC101898644	purine nucleoside phosphorylase%2C transcript variant X3‖purine nucleoside phosphorylase isoform X3
gene-LOC101898663	3220.613	5760.809	-0.83894	2.69E-08	3.10E-05	LOC101898663	carnitine O-palmitoyltransferase 1%2C liver isoform%2C transcript variant X2
gene-LOC101899240	427.1694	813.9368	-0.93011	1.59E-07	0.000143	LOC101899240	carbohydrate sulfotransferase 13 isoform X1‖carbohydrate sulfotransferase 13%2C transcript variant X1
gene-LOC101899684	319.0514	162.4386	0.97389	3.00E-05	0.010556	LOC101899684	vitellogenin-1-like
gene-LOC101900622	1708.167	2575.088	-0.59217	0.000137	0.033956	LOC101900622	lipid storage droplets surface-binding protein 2%2C transcript variant X4‖lipid storage droplets surface-binding protein 2 isoform X4
gene-LOC101900736	121.8936	256.4569	-1.0731	5.93E-06	0.002613	LOC101900736	serine palmitoyltransferase 2 isoform X1‖serine palmitoyltransferase 2%2C transcript variant X1
gene-LOC101901151	82.64153	12.47847	2.7274	7.90E-07	0.000555	LOC101901151	nose resistant to fluoxetine protein 6

gene-LOC101901643	2432.947	1606.99	0.59834	0.000135	0.033956	LOC101901643	probable cytochrome P450 4ac1%2C transcript variant X2||probable cytochrome P450 4ac1 isoform X2
gene-LOC105261927	114.1245	38.37932	1.5722	2.52E-06	0.001569	LOC105261927	farnesyl pyrophosphate synthase
gene-LOC105262272	49.1274	2.467303	4.3155	6.23E-11	1.95E-07	LOC105262272	lectin subunit alpha-like
gene-LOC105262391	92.9014	28.5317	1.7031	0.000242	0.048806	LOC105262391	turripeptide Pal9.2-like
gene-LOC105262393	27.14866	1.73886	3.9647	2.66E-06	0.001592	LOC105262393	uncharacterized protein LOC105262393||uncharacterized LOC105262393
gene-LOC109613852	95.55221	217.5883	-1.1872	3.97E-06	0.001988	LOC109613852	von Willebrand factor D and EGF domain-containing protein
gene-LOC109613860	54.41317	134.565	-1.3063	1.43E-05	0.005516	LOC109613860	uncharacterized LOC109613860||uncharacterized protein LOC109613860
gene-LOC109614246	786.8491	2450.648	-1.639	9.88E-08	9.98E-05	LOC109614246	-
gene-defensin-1	114.2811	45.94871	1.3145	4.20E-05	0.013862	defensin-1	phormicin-like||uncharacterized protein LOC101887540
