# Supplementary material for: Short-term particulate matter contamination severely compromises insect antennal olfactory perception
Source: Nat Commun. 2023 Jul 11;14:4112. doi: 10.1038/s41467-023-39469-3 (PMC10336072; doi:10.1038/s41467-023-39469-3)
Supplement: Supplementary file 6 — Supplementary Data 3 [file 41467_2023_39469_MOESM6_ESM.pdf]

Supplementary Data 3. Details of differential expressed genes (DEGs) between antennae of uncontaminated and contaminated female houseflies collected in summer. Readcount is the average read count of each gen, log2FoldChange is calculated by formular log2(readcount_CFA/readcount_UFA), pval is the *p* value, padj is the fdr corrected *p* value using Benjamini/Hochberg method. All *p*-values are based on two-sided tests. CFA: contaminated female antennae, UFA: uncontaminated female antennae.

Gene_id	readcount_CFA	readcount_UFA	log2FoldChange	pval	padj	Description
AQP	6133.97	7547.464	-0.29917	1.43E-06	0.000118	aquaporin
CPR	29789.14	25584.32	0.21953	0.000156	0.005899	NADPH--cytochrome P450 reductase-like, transcript variant X1
CYP6D3	19041.78	25119.47	-0.39964	1.03E-10	2.11E-08	cytochrome P450 6d3-like, transcript variant X1
CYP6D8	2143.309	1771.187	0.27512	0.000216	0.007638	probable cytochrome P450 6d5-like
CYP6G4	57390.1	74056.76	-0.36783	1.24E-10	2.48E-08	cytochrome P450 6g1-like
Gr2	8254.64	6048.197	0.4487	1.66E-08	2.13E-06	gustatory and odorant receptor 63a-like
HSP70	56848.76	46968.72	0.27543	1.50E-06	0.000123	heat shock 70 kDa protein cognate 4
IR25a	4840.003	4134.598	0.22726	0.001622	0.035787	glutamate receptor ionotropic, kainate 4-like
LOC101887198	310.015	178.4075	0.79716	1.21E-07	1.33E-05	atrial natriuretic peptide receptor 1
LOC101887207	754.2882	566.3257	0.41348	0.00015	0.005717	protein MTO1 homolog, mitochondrial
LOC101887230	389.8682	244.067	0.67571	4.77E-07	4.43E-05	cleft lip and palate transmembrane protein 1-like protein, transcript variant X1
LOC101887269	18657.24	16308.78	0.19409	0.000943	0.023696	uncharacterized LOC101887269
LOC101887288	33.11105	11.63365	1.509	0.001927	0.040615	glutamate-gated chloride channel, transcript variant X5
LOC101887292	817.0186	1015.218	-0.31335	0.000656	0.017672	histone-lysine N-methyltransferase E(z), transcript variant X1
LOC101887315	5191.33	4521.466	0.19931	0.002081	0.042924	mannose-6-phosphate isomerase
LOC101887318	129.1161	81.68721	0.66049	0.00228	0.045512	lectin subunit alpha-like
LOC101887332	112600.4	99770.72	0.17452	0.002197	0.044644	venom carboxylesterase-6
LOC101887345	64.90674	136.1261	-1.0685	3.01E-06	0.000214	uncharacterized LOC101887345
LOC101887354	1613.048	1901.651	-0.23746	0.002029	0.042096	protein transport protein Sec61 subunit beta
LOC101887356	503.1148	643.744	-0.3556	0.000759	0.019866	mediator of RNA polymerase II transcription subunit 10
LOC101887379	17921.54	13862.29	0.37053	3.40E-10	6.32E-08	uncharacterized LOC101887379, transcript variant X1
LOC101887402	352.698	222.9784	0.66153	2.37E-06	0.000179	rootletin, transcript variant X1
LOC101887413	674.5008	480.5942	0.489	0.001132	0.027483	elongation of very long chain fatty acids protein 6

LOC101887417	49.18491	92.60628	-0.91289	0.000695	0.018481	sodium-dependent nutrient amino acid transporter 1
LOC101887443	1953.975	190.4422	3.359	6.28E-246	1.09E-241	actin-87E
LOC101887458	15607.07	13097.42	0.25292	1.79E-05	0.000978	heat shock 70 kDa protein cognate 3, transcript variant X2
LOC101887496	1093.141	1340.504	-0.2943	0.000907	0.023057	thioredoxin-like protein 1
LOC101887499	2305.946	2922.705	-0.34195	1.20E-06	0.000101	venom carboxylesterase-6
LOC101887521	629.1533	777.0141	-0.30453	0.001947	0.040896	probable prefoldin subunit 6
LOC101887528	51.73482	9.076814	2.5109	1.43E-08	1.83E-06	uncharacterized transmembrane protein DDB_G0289901-like
LOC101887539	1743.384	2359.452	-0.43656	3.39E-09	4.99E-07	PAX3- and PAX7-binding protein 1, transcript variant X3
LOC101887559	7215.947	5738.669	0.33047	1.31E-07	1.40E-05	uncharacterized LOC101887559, transcript variant X1
LOC101887571	490.3611	345.4525	0.50536	2.46E-05	0.001285	transmembrane emp24 domain-containing protein 6
LOC101887604	1021.111	770.7809	0.40575	8.66E-06	0.000526	transmembrane protein 104 homolog, transcript variant X2
LOC101887647	225.4575	125.8769	0.84084	1.64E-06	0.000129	transcription factor grauzone-like, transcript variant X2
LOC101887696	1510.245	1953.887	-0.37156	1.41E-06	0.000117	succinate dehydrogenase assembly factor 4, mitochondrial
LOC101887728	6679.347	5518.907	0.27532	1.26E-05	0.000731	gamma-glutamyltranspeptidase 1
LOC101887765	71.77808	132.2639	-0.8818	8.23E-05	0.003519	serine protease nudel
LOC101887774	166.3935	235.5989	-0.50173	0.002002	0.041635	insulin-like growth factor 2 mRNA-binding protein 1, transcript variant X1
LOC101887777	30.59834	74.69842	-1.2876	3.92E-05	0.001889	attacin-A-like
LOC101887788	273.8588	477.0439	-0.80069	2.23E-10	4.31E-08	uncharacterized LOC101887788
LOC101887803	8996.504	7682.131	0.22786	0.000199	0.007151	acyl-CoA Delta(11) desaturase, transcript variant X1
LOC101887889	127.1037	205.4568	-0.69283	0.000159	0.005968	putative uncharacterized protein DDB_G0267716
LOC101887892	447.2539	331.3999	0.43252	0.000494	0.014382	probable isoaspartyl peptidase/L-asparaginase GA20639
LOC101887902	3495.696	4211.55	-0.26877	4.41E-05	0.002087	kinesin-related protein 4, transcript variant X1
LOC101887904	1048.017	260.0049	2.0111	9.95E-42	1.92E-38	cell division protein ZipA
LOC101887963	10126.73	8295.345	0.2878	1.88E-05	0.001025	4-coumarate--CoA ligase-like 7
LOC101887991	1095.339	888.8846	0.30131	0.000772	0.020125	uncharacterized LOC101887991
LOC101888004	9320.828	8051.4	0.21122	0.000545	0.01552	pyruvate carboxylase, mitochondrial, transcript variant X1
LOC101888011	286.4635	499.6461	-0.80256	1.80E-08	2.26E-06	dynein intermediate chain 2, ciliary

LOC101888016	5.53141	24.82115	-2.1659	0.000373	0.011675	uncharacterized LOC101888016
LOC101888025	1582.218	1897.999	-0.26253	0.000583	0.01626	charged multivesicular body protein 1b
LOC101888066	10183.47	7473.322	0.44641	2.50E-13	6.99E-11	adenylate kinase isoenzyme 5, transcript variant X2
LOC101888074	141.3654	85.1853	0.73075	0.000616	0.016889	ficolin-1
LOC101888087	302.9397	448.9242	-0.56744	5.87E-06	0.000368	uncharacterized LOC101888087
LOC101888113	141.4212	86.6201	0.70723	0.001424	0.032496	pancreas transcription factor 1 subunit alpha
LOC101888123	1343.888	1633.047	-0.28115	0.000375	0.011701	cold shock domain-containing protein CG9705
LOC101888148	2567.763	1811.456	0.50336	6.08E-12	1.41E-09	hemocytin, transcript variant X2
LOC101888165	1345.207	1097.723	0.29331	0.000462	0.01354	aldehyde dehydrogenase, mitochondrial
LOC101888190	194.2743	280.6926	-0.5309	0.000404	0.012293	uncharacterized LOC101888190
LOC101888218	14107.01	16118.34	-0.19229	0.001161	0.027965	female-specific protein transformer, transcript variant X1
LOC101888226	18253.6	22034.28	-0.27157	7.02E-05	0.0031	peroxiredoxin-6
LOC101888253	1471.663	2928.966	-0.99294	0.000368	0.011543	MoaC family
LOC101888268	5776.6	4365.937	0.40393	3.34E-06	0.000232	alcohol dehydrogenase class-3
LOC101888287	65.43604	148.8847	-1.186	7.78E-08	8.83E-06	tektin-3
LOC101888299	2462.812	2850.805	-0.21106	0.002541	0.049164	proteasome subunit alpha type-3
LOC101888337	322.2982	201.1484	0.68014	2.67E-06	0.000193	10 kDa heat shock protein, mitochondrial
LOC101888348	1717.237	2113.466	-0.29952	6.94E-05	0.003074	hypothetical protein, transcript variant X2
LOC101888350	115.0094	53.15869	1.1134	6.31E-06	0.000394	peritrophin-48
LOC101888355	1554.41	1251.415	0.31281	0.000128	0.005022	long-chain-fatty-acid--CoA ligase 4, transcript variant X1
LOC101888379	164.4148	254.0564	-0.62781	8.57E-05	0.003606	LEM domain-containing protein Bocksbeutel, transcript variant X3
LOC101888396	1086.338	1446.56	-0.41315	5.09E-07	4.70E-05	U4/U6.U5 tri-snRNP-associated protein 1
LOC101888402	139.233	86.11144	0.69322	0.002441	0.047666	serine-rich adhesin for platelets
LOC101888428	341.1602	810.7232	-1.2488	1.84E-30	2.13E-27	aquaporin AQPcic
LOC101888446	579.0175	717.83	-0.31004	0.002297	0.045762	zinc finger protein BRUTUS-like At1g74770, transcript variant X1
LOC101888462	30104.88	38376.81	-0.35024	1.58E-06	0.000126	general odorant-binding protein 69a
LOC101888467	45.3073	11.45349	1.984	0.000281	0.009424	alpha-methyldopa hypersensitive protein
LOC101888470	647.9447	957.2438	-0.56302	3.00E-09	4.49E-07	protein CREBRF homolog, transcript variant X2

LOC101888499	237.965	161.6525	0.55785	0.000658	0.017683	lambda-crystallin-like
LOC101888535	3133.693	3828.569	-0.28894	1.71E-05	0.000939	fatty-acid amide hydrolase 2-A
LOC101888560	1916.541	2265.464	-0.2413	0.001158	0.027938	26S proteasome non-ATPase regulatory subunit 7
LOC101888566	481.4136	643.9957	-0.41977	8.52E-05	0.003599	RNA-binding protein 8A
LOC101888567	737.2865	473.0827	0.64013	9.92E-10	1.67E-07	uncharacterized LOC101888567
LOC101888589	2160.672	1796.974	0.26591	0.000316	0.010286	dolichyl-diphosphooligosaccharide--protein glycosyltransferase 48 kDa subunit
LOC101888593	602.9604	872.094	-0.53242	3.93E-08	4.78E-06	heat shock protein 27
LOC101888611	227.5326	27.24321	3.0621	6.94E-29	7.09E-26	uncharacterized transmembrane protein DDB_G0289901
LOC101888614	16950.74	13801.19	0.29656	5.69E-07	5.17E-05	fatty acid synthase
LOC101888615	624.3029	95.12815	2.7143	4.94E-82	2.14E-78	uncharacterized LOC101888615
LOC101888623	123.1653	198.6794	-0.68985	0.000143	0.005521	probable tubulin polyglutamylase TTLL1
LOC101888639	105.6452	173.7872	-0.71809	0.000184	0.006754	transient receptor potential cation channel subfamily V member 5
LOC101888651	865.2143	655.7499	0.39991	7.03E-05	0.0031	UNC93-like protein MFSD11, transcript variant X1
LOC101888660	898.8552	1121.99	-0.3199	0.000311	0.010149	peptidyl-prolyl cis-trans isomerase G
LOC101888662	4536.948	3882.218	0.22484	0.000628	0.017157	uncharacterized LOC101888662
LOC101888665	748.7858	559.6692	0.41998	3.86E-05	0.001877	erythroid differentiation-related factor 1
LOC101888706	495.4671	325.9988	0.60392	6.87E-07	6.11E-05	uncharacterized LOC101888706, transcript variant X1
LOC101888715	239.2551	161.4017	0.56789	0.001452	0.033047	mediator of RNA polymerase II transcription subunit 4
LOC101888725	549.5414	431.7685	0.34797	0.002146	0.043996	uncharacterized LOC101888725
LOC101888736	5850.124	7912.919	-0.43574	0.001097	0.026814	bacchus
LOC101888795	1318.749	887.3089	0.57166	3.51E-11	7.62E-09	uncharacterized LOC101888795, transcript variant X2
LOC101888805	2764.963	2203.567	0.32742	4.35E-06	0.000289	dolichyl-diphosphooligosaccharide--protein glycosyltransferase subunit 2, transcript variant X2
LOC101888854	1266.444	1041.179	0.28256	0.00137	0.031631	uncharacterized LOC101888854, transcript variant X1
LOC101888899	305.7231	429.8199	-0.49151	0.000106	0.004272	mitogen-activated protein kinase 15
LOC101888907	1509.482	1843.818	-0.28864	0.00068	0.018183	glycerol-3-phosphate dehydrogenase [NAD(+)], cytoplasmic, transcript variant X3

LOC101888941	859.8083	663.7126	0.37346	0.000203	0.007256	serine/threonine-protein kinase S6KL
LOC101888949	2753.647	2354.433	0.22596	0.001317	0.030527	calcium-transporting ATPase sarcoplasmic/endoplasmic reticulum type
LOC101888958	346.1232	213.1915	0.69914	7.86E-07	6.86E-05	sodium- and chloride-dependent GABA transporter ine
LOC101888968	16103.8	22167.94	-0.46107	4.02E-15	1.34E-12	actin-5C, transcript variant X2
LOC101888969	1846.617	1465.936	0.33306	1.99E-05	0.001069	elongation factor G, mitochondrial
LOC101888987	178.7996	120.4717	0.56965	0.002325	0.046029	ATP-dependent DNA helicase Q-like 5
LOC101888991	39.6797	120.6815	-1.6047	4.40E-10	7.95E-08	glycerophosphocholine phosphodiesterase GPCPD1
LOC101889001	510.8938	744.3955	-0.54305	1.80E-07	1.85E-05	uncharacterized LOC101889001, transcript variant X1
LOC101889018	259.1513	350.629	-0.43615	0.00132	0.030527	uncharacterized LOC101889018
LOC101889023	142.8727	84.88019	0.75123	0.000472	0.01382	mucin-2
LOC101889026	336.383	241.9915	0.47515	0.000662	0.017759	aldehyde dehydrogenase X, mitochondrial
LOC101889033	2358.841	2009.899	0.23096	0.001551	0.034829	eukaryotic translation initiation factor 3 subunit L
LOC101889119	4640.755	4028.946	0.20396	0.002273	0.045512	L-dopachrome tautomerase yellow-f2
LOC101889163	1735.377	2416.083	-0.47742	1.03E-10	2.11E-08	uncharacterized LOC101889163
LOC101889164	87.12087	35.27638	1.3043	7.06E-06	0.000434	uncharacterized LOC101889164
LOC101889182	1169.137	900.749	0.37625	1.77E-05	0.000968	CD2 antigen cytoplasmic tail-binding protein 2 homolog
LOC101889193	5626.59	4719.802	0.25353	7.44E-05	0.003239	UDP-glucuronosyltransferase 2A2-like, transcript variant X2
LOC101889224	3652.711	2923.737	0.32115	2.44E-06	0.000182	lysophospholipid acyltransferase 1, transcript variant X5
LOC101889227	3607.855	2821.685	0.35458	2.04E-07	2.06E-05	phosphatidate phosphatase LPIN1, transcript variant X5
LOC101889238	482.7255	632.5645	-0.39001	0.000275	0.009304	uncharacterized protein YJR142W
LOC101889275	806.8575	1013.079	-0.32836	0.000272	0.009217	esterase B1
LOC101889281	207.3496	320.4885	-0.62821	1.55E-05	0.000858	proton-coupled amino acid transporter 4, transcript variant X2
LOC101889283	54.03484	94.22321	-0.80219	0.002308	0.045832	filamin-B
LOC101889303	559.1251	702.9333	-0.33022	0.001389	0.0319	putative sulfiredoxin, transcript variant X3
LOC101889365	12267.37	10503.31	0.22398	0.000269	0.009142	cytochrome P450 6A1-like
LOC101889374	2750.626	4359.389	-0.66437	1.30E-22	7.55E-20	dynein beta chain, ciliary
LOC101889402	1666.625	2045.68	-0.29565	8.22E-05	0.003519	CD109 antigen, transcript variant X3

LOC101889413	1954.544	2378.598	-0.28328	0.001272	0.029776	solute carrier family 41 member 2, transcript variant X4
LOC101889421	377.5335	490.596	-0.37793	0.001296	0.03017	carbonic anhydrase
LOC101889452	1264.632	1553.822	-0.2971	0.000264	0.008998	glycine-rich cell wall structural protein 1, transcript variant X4
LOC101889462	3271.56	3810.968	-0.22018	0.001046	0.025839	uncharacterized protein CG3556
LOC101889463	342.1117	615.3334	-0.8469	1.47E-13	4.26E-11	uncharacterized LOC101889463
LOC101889502	5429.445	4572.848	0.24771	0.000115	0.004571	RING finger protein 10
LOC101889504	2083.193	1773.637	0.23209	0.001948	0.040896	dnaJ homolog subfamily C member 3
LOC101889554	9.88898	48.39002	-2.2908	3.69E-07	3.52E-05	phospholipase A1 2
LOC101889623	6252.09	5224.4	0.25907	7.46E-05	0.003239	eukaryotic translation initiation factor 3 subunit C
LOC101889651	714.4613	434.8332	0.71639	1.24E-09	2.04E-07	uncharacterized LOC101889651
LOC101889653	2.363483	41.79637	-4.1444	1.01E-11	2.28E-09	circadian clock-controlled protein
LOC101889672	306.5305	414.2209	-0.43437	0.000646	0.017499	cytochrome P450 CYP12A2-like
LOC101889704	174.0367	109.4619	0.66896	0.000452	0.013355	probable cytochrome P450 6a21
LOC101889705	807.8015	1048.995	-0.37693	3.26E-05	0.001611	lamin Dm0, transcript variant X1
LOC101889715	2716.054	2315.841	0.22998	0.001114	0.027117	Malate/L-lactate dehydrogenase, transcript variant X1
LOC101889717	1735.327	1287.757	0.43035	4.94E-08	5.83E-06	chitinase-like protein Idgf4
LOC101889749	727.2686	926.4801	-0.34927	0.000201	0.007196	ADP-ribosylation factor-like protein 1
LOC101889761	1377.745	1721.774	-0.32159	3.99E-05	0.001908	IST1 homolog, transcript variant X2
LOC101889768	194.9915	272.2073	-0.48129	0.001805	0.038693	gonadotropin-releasing hormone receptor
LOC101889773	6391.832	7519.94	-0.23449	0.000888	0.022676	UDP-glucuronosyltransferase 2B9-like
LOC101889802	1136.785	1696.095	-0.57726	2.14E-06	0.000162	ubiquitin-conjugating enzyme E2 W
LOC101889806	4279.876	3560.389	0.26553	5.42E-05	0.002508	active breakpoint cluster region-related protein, transcript variant X4
LOC101889808	1546.965	1131.089	0.45173	0.000978	0.024393	proton-coupled amino acid transporter-like protein pathetic, transcript variant X1
LOC101889813	23.26442	116.0907	-2.3191	4.04E-08	4.87E-06	vitellogenin-3-like
LOC101889827	1413.993	1138.448	0.31271	0.000146	0.005627	uncharacterized LOC101889827, transcript variant X3
LOC101889832	2262.96	531.6234	2.0897	1.17E-42	2.53E-39	general odorant-binding protein 57c-like
LOC101889836	2017.902	1580.865	0.35214	0.000194	0.007016	60S ribosomal protein L10

LOC101889913	142.8948	83.6827	0.77195	0.00034	0.010817	N-acetylglucosaminyl-phosphatidylinositol de-N-acetylase
LOC101889943	1913.35	2357.228	-0.30099	3.60E-05	0.001756	tight junction protein ZO-1, transcript variant X4
LOC101889953	694.5212	850.2902	-0.29194	0.002252	0.045418	uncharacterized LOC101889953, transcript variant X4
LOC101889980	2726.665	2191.987	0.3149	2.13E-05	0.001136	endoplasmic reticulum metallopeptidase 1, transcript variant X1
LOC101890000	3786.692	2903.419	0.38319	2.84E-07	2.78E-05	fatty acyl-CoA reductase wat
LOC101890005	318.9238	463.9344	-0.54071	1.12E-05	0.000659	C-type lectin 37Db, transcript variant X2
LOC101890032	383.905	290.955	0.39995	0.002314	0.045874	dnaJ protein homolog 1, transcript variant X2
LOC101890039	1716.021	1422.17	0.27097	0.000536	0.015298	short neuropeptide F, transcript variant X1
LOC101890053	2186.176	1821.023	0.26366	0.000382	0.011833	DNA-directed RNA polymerase II subunit RPB2
LOC101890094	36.43095	0	Inf	3.82E-15	1.30E-12	vitelline membrane protein Vm26Ab-like
LOC101890109	335.9811	462.4184	-0.46082	0.000158	0.005946	hypothetical protein
LOC101890117	1198.838	1001.631	0.25929	0.002419	0.047353	rabankyrin-5
LOC101890140	543.8644	420.0185	0.37279	0.001077	0.026408	putative odorant receptor 92a
LOC101890159	706.024	540.8745	0.38442	0.00157	0.034996	odorant receptor 2a-like
LOC101890173	29.4632	9.775648	1.5916	0.00219	0.044575	plancitoxin-1
LOC101890191	1661.078	1955.831	-0.23566	0.001892	0.040156	26S proteasome non-ATPase regulatory subunit 8
LOC101890217	2853.439	1734.301	0.71835	4.01E-23	2.58E-20	general odorant-binding protein 19d
LOC101890222	1530.7	1244.95	0.2981	0.000248	0.008564	uncharacterized LOC101890222, transcript variant X2
LOC101890230	704.1326	551.4906	0.35251	0.000559	0.015776	furin-like protease 2
LOC101890259	37.0306	0	Inf	1.80E-15	6.39E-13	cuticle protein 16.5
LOC101890271	7744.856	11473.61	-0.56701	1.08E-20	5.51E-18	UDP-glucuronosyltransferase 2B15, transcript variant X1
LOC101890297	1362.57	989.2969	0.46185	6.36E-05	0.002849	short/branched chain specific acyl-CoA dehydrogenase, mitochondrial
LOC101890313	51.50973	94.60809	-0.87712	0.000787	0.02042	uncharacterized LOC101890313
LOC101890318	437.0839	552.5944	-0.33831	0.002463	0.048002	rho GTPase-activating protein 18, transcript variant X1
LOC101890335	838.316	1054.218	-0.33061	0.000656	0.017672	probable cytochrome P450 28d1
LOC101890341	1942.229	1620.002	0.26172	0.000594	0.016502	presenilin homolog, transcript variant X3
LOC101890356	60.391	25.80958	1.2264	0.000349	0.011044	uncharacterized LOC101890356

LOC101890413	636.276	929.6672	-0.54706	9.92E-09	1.32E-06	KH domain-containing, RNA-binding, signal transduction-associated protein 2, transcript variant X1
LOC101890414	562.9594	82.40098	2.7723	1.28E-77	4.46E-74	actin-57B
LOC101890447	1014.703	748.063	0.43983	2.11E-06	0.00016	protein spaetzle 3, transcript variant X1
LOC101890474	3.267321	20.213	-2.6291	0.000378	0.011764	uncharacterized LOC101890474
LOC101890500	719.0488	522.0847	0.46181	8.36E-05	0.003563	GRIP and coiled-coil domain-containing protein 1
LOC101890508	175.8132	75.19237	1.2254	2.95E-09	4.45E-07	atrial natriuretic peptide receptor 1
LOC101890516	634.059	820.424	-0.37175	0.00016	0.005987	integrin alpha-PS3
LOC101890539	178.4422	62.81822	1.5062	1.13E-12	2.84E-10	uncharacterized LOC101890539
LOC101890549	2065.589	1618.728	0.35169	2.91E-06	0.000208	transcription factor mef2A, transcript variant X9
LOC101890572	3375.333	2714.636	0.31427	4.96E-06	0.000322	alpha-mannosidase 2
LOC101890586	929.3361	1139.984	-0.29474	0.000815	0.021096	uncharacterized LOC101890586
LOC101890619	680.461	452.5612	0.5884	4.25E-08	5.09E-06	laminin subunit gamma-1, transcript variant X1
LOC101890621	184.1102	267.4709	-0.53881	0.000488	0.014206	uncharacterized LOC101890621
LOC101890623	3805.32	3139.03	0.2777	0.001579	0.035036	cysteine and histidine-rich protein 1 homolog, transcript variant X2
LOC101890631	745.2735	602.3504	0.30717	0.002149	0.043996	DNA-directed RNA polymerase III subunit RPC2
LOC101890668	1071.519	869.3351	0.30167	0.000759	0.019866	protein hemingway
LOC101890706	3287.425	3875.016	-0.23724	0.000449	0.013335	double-stranded RNA-specific editase B2
LOC101890714	3129.483	4714.964	-0.59132	5.42E-19	2.62E-16	probable cytochrome P450 28a5
LOC101890720	227.4151	375.953	-0.72522	1.44E-07	1.54E-05	glycine-rich cell wall structural protein 1, transcript variant X3
LOC101890724	5301.093	6806.412	-0.3606	1.04E-08	1.36E-06	chitinase-like protein Idgf4
LOC101890728	2897.733	3501.587	-0.27308	5.34E-05	0.002477	probable cytochrome P450 313a4
LOC101890734	479.7301	2155.641	-2.1678	2.74E-136	2.38E-132	uncharacterized LOC101890734, transcript variant X2
LOC101890765	191.6079	304.4288	-0.66795	8.87E-06	0.000537	4-aminobutyrate aminotransferase, mitochondrial
LOC101890768	987.67	1198.416	-0.27903	0.001227	0.029017	dorsal-related immunity factor Dif, transcript variant X2
LOC101890854	7716.925	5898.9	0.38758	3.45E-06	0.000238	glucose dehydrogenase [FAD, quinone]
LOC101890875	12.23216	0.979587	3.6424	0.000904	0.02301	juvenile hormone acid O-methyltransferase-like
LOC101890909	207.9135	107.4595	0.95219	1.75E-07	1.82E-05	transcription factor grauzone

LOC101890912	1474.721	1101.456	0.42103	3.10E-07	3.01E-05	zinc finger CCHC domain-containing protein 8 homolog
LOC101890927	693.9741	480.7282	0.52966	1.43E-06	0.000118	uncharacterized LOC101890927
LOC101890934	7898.871	6487.71	0.28394	4.97E-06	0.000322	low-density lipoprotein receptor-related protein 2
LOC101890936	437.2151	737.5321	-0.75436	1.46E-12	3.61E-10	heat shock protein 23
LOC101890938	3325.325	2773.107	0.26199	0.000141	0.005472	transmembrane emp24 domain-containing protein eca
LOC101890978	237.7288	363.0509	-0.61086	7.67E-06	0.000469	uncharacterized LOC101890978, transcript variant X2
LOC101890993	69.49242	137.2446	-0.98182	6.30E-05	0.002844	uncharacterized oxidoreductase SSP0419-like
LOC101891012	69.66621	133.6232	-0.93964	2.83E-05	0.001434	uncharacterized LOC101891012
LOC101891022	902.7098	1115.225	-0.305	0.000608	0.016765	protein pangolin, isoforms A/H/I/S, transcript variant X2
LOC101891064	85.7418	187.9443	-1.1322	9.98E-09	1.32E-06	sodium-independent sulfate anion transporter
LOC101891141	1194.856	1455.608	-0.28479	0.000485	0.01414	saccharopine dehydrogenase-like oxidoreductase
LOC101891188	758.937	1108.008	-0.54592	1.74E-09	2.77E-07	serine-rich adhesin for platelets-like
LOC101891189	237.561	151.7902	0.64622	9.62E-05	0.003911	uncharacterized LOC101891189
LOC101891230	2329.367	1896.586	0.29653	0.001578	0.035036	glutamate receptor ionotropic, kainate 1, transcript variant X2
LOC101891245	336.7104	245.55	0.45549	0.001066	0.026252	GSK3-beta interaction protein
LOC101891251	58.10338	15.08107	1.9459	4.51E-07	4.26E-05	vitellogenin-1-like
LOC101891258	393.8406	294.341	0.42012	0.001231	0.029036	DExH-box ATP-dependent RNA helicase DExH3
LOC101891268	1175.067	833.6272	0.49527	2.71E-08	3.36E-06	CD151 antigen
LOC101891286	19.59455	55.34185	-1.4979	0.000243	0.008437	carnitine O-acetyltransferase-like
LOC101891339	1028.052	1270.29	-0.30524	0.00033	0.010598	uncharacterized LOC101891339
LOC101891393	872.55	695.7216	0.32673	0.000561	0.015776	U2 snRNP-associated SURP motif-containing protein
LOC101891411	944.7073	1284.709	-0.4435	0.000848	0.021812	nucleoplasmin-like protein
LOC101891414	212.9291	336.3564	-0.65962	3.92E-06	0.000268	cuticle protein 38
LOC101891419	208.1848	57.33698	1.8603	2.39E-19	1.19E-16	uncharacterized lipoprotein TP_0136
LOC101891420	584.3319	458.4064	0.35016	0.001379	0.031758	ATP-dependent RNA helicase Ddx1
LOC101891455	239.1745	341.5446	-0.51401	0.000335	0.010712	molybdopterin synthase catalytic subunit
LOC101891457	4711.503	3786.213	0.31543	0.000524	0.015041	glycine-rich protein 5-like
LOC101891473	388.1636	288.525	0.42797	0.001083	0.026527	UPF0587 protein CG4646

LOC101891504	550.6458	777.7765	-0.49823	0.000924	0.023347	uncharacterized LOC101891504
LOC101891510	152.3628	325.8528	-1.0967	7.56E-13	2.02E-10	serine proteinase stubble
LOC101891519	1508.303	1266.751	0.25179	0.001785	0.038361	uncharacterized LOC101891519, transcript variant X2
LOC101891525	652.305	507.8064	0.36127	0.000715	0.018917	uncharacterized LOC101891525
LOC101891527	412.3653	107.7807	1.9358	1.43E-34	2.25E-31	protein no-on-transient A
LOC101891532	6104.154	5162.871	0.24162	0.001043	0.02583	odorant receptor coreceptor
LOC101891535	551.9242	433.0999	0.34977	0.001916	0.040517	chitinase-like protein Idgf5
LOC101891572	172.3783	261.7309	-0.60251	0.000133	0.005214	membrane alanyl aminopeptidase
LOC101891623	3130.28	2592.959	0.27169	9.41E-05	0.003864	tyrosine-protein kinase Src42A, transcript variant X5
LOC101891652	15.01955	40.79851	-1.4417	0.000746	0.019638	uncharacterized LOC101891652
LOC101891662	199.0756	114.8779	0.79322	1.36E-05	0.000777	uncharacterized LOC101891662
LOC101891684	1020.7	1443.925	-0.50044	2.59E-09	3.98E-07	proton-coupled amino acid transporter 2
LOC101891711	733.7025	512.2061	0.51847	4.67E-07	4.38E-05	WD repeat and HMG-box DNA-binding protein 1
LOC101891785	453.9638	198.606	1.1927	8.95E-19	4.09E-16	uncharacterized LOC101891785
LOC101891808	3941.862	2041.397	0.94932	1.20E-10	2.43E-08	uncharacterized LOC101891808
LOC101891810	1988.576	2367.351	-0.25154	0.001459	0.033123	activating transcription factor 7-interacting protein 1, transcript variant X1
LOC101891811	54.40915	97.97691	-0.84859	0.001169	0.02808	dynein assembly factor 1, axonemal homolog
LOC101891823	882.1065	1103.237	-0.32272	0.000336	0.010716	transmembrane protein 164, transcript variant X2
LOC101891848	95.12458	46.04027	1.0469	9.48E-05	0.003874	serine/threonine-protein kinase grp, transcript variant X1
LOC101891870	7017.396	8005.441	-0.19005	0.002048	0.042433	serine/arginine repetitive matrix protein 2, transcript variant X5
LOC101891896	11.96044	0.970159	3.6239	0.000977	0.024393	uncharacterized protein DDB_G0283357-like
LOC101891926	232.4629	164.7111	0.49706	0.00237	0.04667	TBC1 domain family member 5 homolog A
LOC101891951	1348.372	1654.969	-0.29559	0.000197	0.007106	uncharacterized LOC101891951, transcript variant X2
LOC101891960	870.0715	1092.815	-0.32884	0.000324	0.01047	uncharacterized LOC101891960
LOC101891965	418.1653	530.2575	-0.34262	0.002492	0.048451	density-regulated protein homolog
LOC101892031	27924.09	23636.79	0.24048	0.001225	0.029014	protein D2-like
LOC101892036	39.26047	75.35011	-0.94053	0.001585	0.035105	uncharacterized LOC101892036

LOC101892041	462.2455	354.4794	0.38296	0.001476	0.033422	vanin-like protein 2
LOC101892046	638.7782	954.7653	-0.57983	1.43E-09	2.33E-07	SRSF protein kinase 1, transcript variant X4
LOC101892069	1918.126	2298.176	-0.26079	0.000405	0.012328	protein DDI1 homolog 2, transcript variant X1
LOC101892105	344.4321	465.9352	-0.43591	0.000291	0.009641	protein argonaute-2, transcript variant X1
LOC101892126	4906.62	5760.257	-0.2314	0.000282	0.009431	2-hydroxyacylsphingosine 1-beta-galactosyltransferase
LOC101892132	1578.781	1150.317	0.45678	1.92E-08	2.40E-06	protein abnormal spindle
LOC101892133	2110.994	3726.449	-0.81988	1.67E-30	2.08E-27	golgin subfamily A member 6-like protein 22
LOC101892192	934.0289	1216.049	-0.38066	1.11E-05	0.000653	uncharacterized LOC101892192
LOC101892211	4097.496	4942.488	-0.27049	3.19E-05	0.001585	uncharacterized LOC101892211
LOC101892230	1963.96	1421.469	0.46638	1.97E-09	3.08E-07	glutamine--fructose-6-phosphate aminotransferase [isomerizing] 2
LOC101892255	310.7027	470.2712	-0.59796	1.31E-06	0.000109	cathepsin L1
LOC101892279	932.7586	1210.413	-0.37592	1.42E-05	0.0008	ubiquitin carboxyl-terminal hydrolase 5
LOC101892289	204.6661	131.6556	0.6365	0.000336	0.010712	translation factor waclaw, mitochondrial
LOC101892311	3512.758	4447.922	-0.34053	2.18E-07	2.19E-05	trehalase
LOC101892380	4538.483	3702.582	0.29368	0.000412	0.01252	nucleolar GTP-binding protein 1
LOC101892386	1194.836	1447.881	-0.27713	0.000637	0.017276	scavenger receptor class B member 1
LOC101892393	13.85813	0	Inf	4.04E-06	0.000274	cytochrome c1, transcript variant X1
LOC101892399	5249.157	6260.614	-0.25422	5.83E-05	0.002669	facilitated trehalose transporter Tret1
LOC101892423	18.81812	48.37058	-1.362	0.000546	0.015526	uncharacterized LOC101892423
LOC101892436	280.8359	201.5929	0.47828	0.00142	0.032452	uncharacterized LOC101892436
LOC101892510	3012.17	3924.896	-0.38185	1.34E-08	1.73E-06	GTP cyclohydrolase 1, transcript variant X2
LOC101892551	147.4633	37.64441	1.9698	1.37E-15	5.05E-13	CD109 antigen
LOC101892556	1203.19	959.9025	0.3259	0.000147	0.005651	glucose-6-phosphate exchanger SLC37A2, transcript variant X1
LOC101892570	105.7647	169.223	-0.67807	0.000391	0.012001	facilitated trehalose transporter Tret1
LOC101892589	1641.668	1271.393	0.36875	6.99E-06	0.000432	uncharacterized LOC101892589, transcript variant X3
LOC101892594	1152.374	1423.175	-0.3045	0.000583	0.01626	chromosomal protein D1
LOC101892606	17206.4	12123.57	0.50513	1.27E-14	3.80E-12	uncharacterized LOC101892606
LOC101892703	503.199	365.0543	0.46302	8.50E-05	0.003599	protein spindle-F

LOC101892720	16.35671	50.57494	-1.6285	4.87E-05	0.00228	putative leucine-rich repeat-containing protein DDB_G0290503
LOC101892733	2127.743	2570.096	-0.2725	0.000163	0.006085	proteasome subunit beta type-1
LOC101892734	301.6402	416.3057	-0.46481	0.000281	0.009431	proton-coupled amino acid transporter-like protein CG1139, transcript variant X2
LOC101892760	1623.338	2424.264	-0.57858	6.75E-15	2.17E-12	protein FAM188B2
LOC101892787	101.6711	13.38535	2.9252	3.83E-09	5.55E-07	uncharacterized LOC101892787
LOC101892856	147.664	85.96665	0.78047	0.000185	0.006762	uncharacterized LOC101892856, transcript variant X2
LOC101892950	549.6182	409.2481	0.42545	0.000188	0.006868	cleavage and polyadenylation specificity factor subunit 1
LOC101892958	1727.642	1335.948	0.37094	2.71E-06	0.000195	hsp90 co-chaperone Cdc37
LOC101892961	320.8736	224.7264	0.51384	0.00035	0.011048	sodium- and chloride-dependent GABA transporter ine
LOC101892992	882.5126	1285.32	-0.54244	4.93E-10	8.65E-08	hydroxymethylglutaryl-CoA synthase 1, transcript variant X2
LOC101893003	880.0635	1522.343	-0.79061	9.03E-10	1.54E-07	Hemolymph juvenile hormone binding protein (JHBP)
LOC101893008	626.1971	827.0045	-0.40128	0.000289	0.009594	protein grindelwald
LOC101893056	756.6734	609.8825	0.31114	0.002176	0.044389	cap-specific mRNA (nucleoside-2'-O-)-methyltransferase 1
LOC101893073	1106.937	1370.3	-0.30792	0.00022	0.007781	1-aminocyclopropane-1-carboxylate oxidase, transcript variant X1
LOC101893093	2808.492	2369.508	0.24521	0.000502	0.014567	protein lifeguard 1, transcript variant X3
LOC101893098	1902.381	1573.189	0.27411	0.000324	0.01047	nucleoprotein TPR
LOC101893128	195.8054	100.2556	0.96574	1.39E-05	0.000789	lectin subunit alpha
LOC101893129	100.8171	154.8766	-0.61938	0.001873	0.039852	apolipoprotein D
LOC101893170	387.3927	516.6681	-0.41544	0.000384	0.011843	uncharacterized LOC101893170
LOC101893183	227.0589	487.6238	-1.1027	1.34E-17	5.67E-15	circadian clock-controlled protein
LOC101893184	520.8705	766.4493	-0.55727	1.22E-07	1.34E-05	peptidoglycan-recognition protein SB1
LOC101893192	22.76096	5.531551	2.0408	0.001243	0.029196	acidic mammalian chitinase
LOC101893216	19.51522	3.600857	2.4382	0.001437	0.032735	monocarboxylate transporter 7
LOC101893225	214.7682	129.9032	0.72534	2.98E-05	0.001509	20-hydroxyecdysone protein
LOC101893230	1479.847	1805.508	-0.28696	0.000211	0.0075	nucleoplasmin-like protein
LOC101893252	390.393	296.3719	0.39752	0.002272	0.045512	menin
LOC101893254	2881.814	2386.043	0.27236	0.000125	0.004914	tyrosine-protein phosphatase 10D

LOC101893257	2645.159	3066.66	-0.21331	0.002198	0.044644	ribose-phosphate pyrophosphokinase 1, transcript variant X3
LOC101893298	657.0221	390.9743	0.74887	9.62E-12	2.20E-09	uncharacterized LOC101893298
LOC101893323	479.1538	659.1705	-0.46016	1.41E-05	0.000797	monocarboxylate transporter 10, transcript variant X1
LOC101893329	1042.847	1818.294	-0.80206	1.03E-12	2.64E-10	dynein beta chain, ciliary
LOC101893377	789.4186	1006.648	-0.3507	0.000817	0.021108	uncharacterized LOC101893377, transcript variant X1
LOC101893387	4837.236	4123.92	0.23017	0.00039	0.012001	pancreatic triacylglycerol lipase
LOC101893397	393.2969	250.5726	0.65039	1.07E-06	9.15E-05	uncharacterized LOC101893397
LOC101893459	54.05814	121.1794	-1.1646	3.16E-06	0.000222	venom peptide Pc
LOC101893476	638.9163	494.3595	0.37007	0.000514	0.014883	glutamyl aminopeptidase
LOC101893481	195.8487	327.3378	-0.74104	4.23E-07	4.01E-05	helicase POLQ-like
LOC101893482	2418.485	2024.861	0.25628	0.000413	0.012526	uncharacterized LOC101893482, transcript variant X2
LOC101893502	2856.728	3877.008	-0.44058	3.42E-10	6.32E-08	failed axon connections, transcript variant X1
LOC101893508	1610.511	624.7488	1.3662	2.38E-14	7.01E-12	myosin heavy chain, muscle, transcript variant X22
LOC101893528	151.0258	221.6259	-0.55333	0.001171	0.028086	serine/threonine-protein phosphatase 6 regulatory ankyrin repeat subunit A, transcript variant X4
LOC101893532	3611.585	5707.765	-0.66029	3.25E-24	2.45E-21	sodium-independent sulfate anion transporter
LOC101893552	0.987634	18.43791	-4.2226	0.000384	0.011843	transient receptor potential cation channel subfamily A member 1
LOC101893577	9.127942	0	Inf	0.00032	0.01038	juvenile hormone acid O-methyltransferase
LOC101893582	1757.919	1323.402	0.40962	1.71E-07	1.79E-05	far upstream element-binding protein 1, transcript variant X1
LOC101893598	584.1889	452.5414	0.36839	0.000959	0.024071	nuclear receptor coactivator 2
LOC101893605	1085.087	1329.193	-0.29274	0.000445	0.013286	Ecdysteroid kinase
LOC101893624	187.3448	272.5366	-0.54075	0.002151	0.043996	TBC1 domain family member 31
LOC101893626	1736.234	1428.001	0.28196	0.000317	0.0103	BAG family molecular chaperone regulator 2, transcript variant X3
LOC101893634	649.4509	477.7247	0.44304	3.93E-05	0.00189	ETS-like protein pointed
LOC101893650	24.94103	53.42943	-1.0991	0.001834	0.039223	larval cuticle protein A3A
LOC101893670	478.1326	362.7083	0.3986	0.00076	0.019866	N-acetyltransferase 9-like protein
LOC101893702	3000.996	2401.975	0.32122	4.65E-06	0.000307	adenosylhomocysteinase
LOC101893720	818.8447	594.2707	0.46247	3.06E-06	0.000217	twitchin, transcript variant X2

LOC101893731	2751.468	1807.465	0.60623	7.69E-17	3.18E-14	venom carboxylesterase-6
LOC101893737	6688.076	9606.463	-0.52241	2.50E-06	0.000184	RNA-binding protein Rsf1
LOC101893770	2431.927	2865.685	-0.23678	0.000693	0.018481	protein transport protein Sec61 gamma-2 subunit, transcript variant X1
LOC101893793	4272.637	3720.551	0.19961	0.002352	0.046455	importin-5
LOC101893801	503.5486	657.4567	-0.38476	0.000307	0.010061	neuropeptide SIFamide receptor
LOC101893834	522.568	682.4865	-0.38518	0.000241	0.008392	microprocessor complex subunit DGCR8, transcript variant X1
LOC101893836	210.8142	348.2726	-0.72424	2.96E-07	2.89E-05	uncharacterized LOC101893836, transcript variant X1
LOC101893866	404.855	259.0784	0.64402	9.49E-07	8.24E-05	probable methyltransferase BMT2 homolog
LOC101893875	2730.286	2320.594	0.23456	0.002436	0.04764	lipase 3-like
LOC101893891	3546.201	3010.723	0.23616	0.000515	0.014883	uncharacterized LOC101893891
LOC101893904	975.3254	728.6971	0.42056	5.79E-06	0.000366	adenylate cyclase type 2
LOC101893921	2022.333	2507.569	-0.31027	0.001222	0.02899	HIG1 domain family member 1A, mitochondrial
LOC101893931	641.874	909.1778	-0.50227	1.91E-07	1.95E-05	SPRY domain-containing SOCS box protein 3
LOC101893959	1421.134	1142.477	0.31488	0.000141	0.005465	2-hydroxyacylsphingosine 1-beta-galactosyltransferase
LOC101893969	78.84074	41.20755	0.93603	0.001145	0.027774	uncharacterized LOC101893969
LOC101893992	312.0217	171.3256	0.86491	9.84E-09	1.32E-06	probable serine/threonine-protein kinase kinX
LOC101894014	5304.537	6282.342	-0.24408	0.000108	0.004325	serine protease 7
LOC101894043	49.16632	92.65895	-0.91426	0.002219	0.045007	dynein heavy chain 6, axonemal
LOC101894050	4661.913	5710.073	-0.29259	4.64E-06	0.000307	lipase 3-like
LOC101894065	183.7782	65.23322	1.4943	9.85E-13	2.55E-10	uncharacterized protein CG45076
LOC101894072	80175.11	66966.57	0.25971	5.66E-06	0.00036	protein timeless
LOC101894106	4322.454	5164.983	-0.25691	6.93E-05	0.003074	apoptotic chromatin condensation inducer in the nucleus
LOC101894128	684.1308	518.026	0.40125	0.000608	0.016765	protein seele
LOC101894143	4520.478	3676.76	0.29804	5.83E-06	0.000367	vacuolar protein sorting-associated protein 13C, transcript variant X3
LOC101894145	3820.062	4611.146	-0.27153	3.13E-05	0.001558	glutamic acid-rich protein
LOC101894157	1585.19	1875.734	-0.2428	0.001727	0.037626	run domain Beclin-1-interacting and cysteine-rich domain-containing protein

LOC101894184	1185.649	934.6907	0.34312	8.48E-05	0.003599	uncharacterized LOC101894184
LOC101894200	10127.78	8648.347	0.22782	0.000173	0.006414	lysosomal aspartic protease-like, transcript variant X1
LOC101894205	663.4823	910.9576	-0.45733	0.000246	0.008537	uncharacterized LOC101894205
LOC101894209	5705.576	4708.868	0.27699	1.54E-05	0.000855	ATP-binding cassette sub-family E member 1, transcript variant X1
LOC101894230	1177.323	893.2945	0.3983	5.06E-06	0.000324	cGMP-dependent protein kinase, isozyme 2 forms cD4/T1/T3A/T3B
LOC101894316	2335.195	2734.612	-0.22779	0.001377	0.031749	26S protease regulatory subunit 6B
LOC101894317	855.8693	680.547	0.3307	0.000538	0.015327	cAMP-dependent protein kinase type I regulatory subunit, transcript variant X1
LOC101894319	581.8643	799.5607	-0.45852	4.23E-06	0.000282	probable serine/threonine-protein kinase yakA
LOC101894328	2678.823	2146.534	0.31959	0.000872	0.022401	DNA topoisomerase 1, transcript variant X2
LOC101894337	354.684	263.6207	0.42807	0.001608	0.035516	zinc finger protein 28 homolog
LOC101894344	4979.081	3696.553	0.4297	5.33E-11	1.13E-08	basic-leucine zipper transcription factor A-like
LOC101894367	578.5031	389.3733	0.57117	4.70E-07	4.39E-05	inositol oxygenase
LOC101894390	380.2918	264.2382	0.52527	7.37E-05	0.003217	protein strawberry notch, transcript variant X1
LOC101894395	1090.422	827.8632	0.39742	1.08E-05	0.000638	protein real-time
LOC101894402	1536.262	1161.924	0.40291	6.91E-07	6.12E-05	acanthoscurrin-1-like
LOC101894425	1265.241	1046.096	0.2744	0.001202	0.028682	cytochrome P450 4d8, transcript variant X1
LOC101894451	1152.583	953.9958	0.27282	0.001563	0.034971	transcription factor GAGA, transcript variant X1
LOC101894479	482.2183	357.3337	0.43241	0.000277	0.009362	carabin
LOC101894492	1131.545	916.8443	0.30354	0.000516	0.014883	mannosyl-oligosaccharide glucosidase
LOC101894507	643.306	864.8639	-0.42697	9.05E-06	0.000546	ankyrin-2, transcript variant X1
LOC101894528	8889.53	10278.55	-0.20946	0.000571	0.015993	protein suppressor of white apricot
LOC101894540	554.2645	700.3673	-0.33754	0.00107	0.02632	uncharacterized LOC101894540
LOC101894546	3535.748	4278.866	-0.27521	9.20E-05	0.003812	uncharacterized LOC101894546
LOC101894565	1525.497	986.68	0.62862	1.01E-09	1.69E-07	general odorant-binding protein 28a
LOC101894567	252.2729	337.5342	-0.42005	0.002186	0.04455	protein yellow
LOC101894579	1338.968	1091.685	0.29456	0.000403	0.012293	putative U5 small nuclear ribonucleoprotein 200 kDa helicase
LOC101894587	210.193	131.8869	0.67241	0.000137	0.005352	FK506-binding protein 5, transcript variant X5

LOC101894605	527.871	159.451	1.7271	5.92E-15	1.94E-12	troponin T, skeletal muscle, transcript variant X2
LOC101894607	3830.593	3195.264	0.26163	0.000103	0.004162	hamartin
LOC101894655	2402.05	1900.476	0.33791	4.08E-06	0.000274	hillarin, transcript variant X2
LOC101894723	229.6552	15.14453	3.9226	1.25E-53	3.61E-50	general odorant-binding protein 28a
LOC101894727	5986.818	7379.397	-0.30171	7.22E-05	0.003165	retinaldehyde-binding protein 1
LOC101894734	363.5725	250.7065	0.53624	8.13E-05	0.003493	tubulin--tyrosine ligase-like protein 12
LOC101894735	2914.71	2243.614	0.37753	1.11E-07	1.23E-05	papilin, transcript variant X1
LOC101894813	689.0641	521.9948	0.4006	0.00011	0.004419	sarcocystatin-A
LOC101894839	647.0153	975.3653	-0.59214	4.59E-10	8.22E-08	G-protein coupled receptor Mth2, transcript variant X3
LOC101894841	1271.994	1052.057	0.27388	0.001212	0.028829	diacylglycerol O-acyltransferase 1, transcript variant X1
LOC101894876	15.34877	2.275885	2.7536	0.001487	0.033577	maltase A1-like
LOC101894885	128.0135	23.34352	2.4552	1.57E-18	6.83E-16	general odorant-binding protein 28a-like
LOC101894912	4.45527	21.17549	-2.2488	0.001235	0.029059	pickpocket protein 28
LOC101894960	2076.638	1683.484	0.3028	0.000259	0.008875	tryptophan--tRNA ligase, cytoplasmic
LOC101894970	168.7973	247.1339	-0.55	0.000635	0.01725	protein LLP homolog
LOC101894989	1388.568	2061.084	-0.56981	1.54E-13	4.38E-11	tiggrin
LOC101895011	599.5671	462.5375	0.37435	0.000635	0.01725	apyrase
LOC101895046	2766.074	2181.67	0.34241	1.70E-06	0.000133	serine/threonine-protein kinase MARK2, transcript variant X3
LOC101895072	390.2815	570.1881	-0.54692	1.58E-06	0.000126	myb-like protein A, transcript variant X3
LOC101895074	1365.978	1883.393	-0.4634	2.80E-09	4.27E-07	peptidoglycan-recognition protein LB
LOC101895088	2677.671	2302.896	0.21753	0.002308	0.045832	probable cyclin-dependent serine/threonine-protein kinase DDB_G0292550
LOC101895094	1148.324	913.3908	0.33023	0.00122	0.028975	glycerol kinase
LOC101895146	736.2781	913.0041	-0.31037	0.001155	0.027902	RB1-inducible coiled-coil protein 1
LOC101895152	2597.805	3230.707	-0.31456	5.11E-06	0.000326	uncharacterized LOC101895152
LOC101895175	1492.143	1862.396	-0.31977	0.000285	0.009466	TGF-beta receptor type-1, transcript variant X2
LOC101895193	359.9125	577.8765	-0.68312	1.93E-07	1.96E-05	pro-resilin
LOC101895219	29336.23	25583.27	0.19748	0.001547	0.034794	putative odorant-binding protein A5

LOC101895236	489.4793	615.7264	-0.33104	0.002233	0.045244	centrosomal protein of 135 kDa, transcript variant X2
LOC101895252	12701.08	15354.88	-0.27375	0.000139	0.00542	ATP-dependent RNA helicase dbp2
LOC101895253	105.6779	187.5258	-0.82741	1.30E-05	0.000745	uncharacterized LOC101895253
LOC101895296	3029.795	2614.044	0.21294	0.001919	0.040538	pre-mRNA-splicing factor ATP-dependent RNA helicase PRP16
LOC101895318	384.492	286.1766	0.42605	0.001194	0.028552	transcription factor Ouib
LOC101895334	3055.155	2161.992	0.49888	2.25E-09	3.49E-07	gustatory and odorant receptor 21a-like
LOC101895351	1921.206	1587.02	0.27569	0.000327	0.010522	uncharacterized LOC101895351, transcript variant X1
LOC101895361	169.013	11.35289	3.896	5.13E-40	8.91E-37	uncharacterized LOC101895361
LOC101895385	4184.935	4843.71	-0.21091	0.001098	0.026814	organic cation transporter protein, transcript variant X1
LOC101895448	1633.825	2334.455	-0.51483	5.93E-12	1.39E-09	ABC transporter G family member 20
LOC101895519	1370.35	1069.997	0.35694	0.001266	0.029696	odorant receptor 45a-like
LOC101895544	4205.933	5358.015	-0.34927	5.83E-08	6.74E-06	vitellogenin-1
LOC101895548	2804.264	3310.983	-0.23964	0.000452	0.013355	arginine/serine-rich protein PNISR, transcript variant X2
LOC101895609	778.1859	628.4445	0.30833	0.00166	0.036437	twinkle protein, mitochondrial
LOC101895629	602.7923	829.9576	-0.46138	2.45E-06	0.000182	heat shock protein 70
LOC101895630	580.194	765.037	-0.39899	7.09E-05	0.003118	nuclear factor NF-kappa-B p110 subunit
LOC101895658	191.0717	82.56601	1.2105	6.20E-09	8.55E-07	muscle M-line assembly protein unc-89, transcript variant X1
LOC101895675	293.2609	154.5856	0.92378	3.13E-09	4.65E-07	proline-rich protein 2
LOC101895695	658.8187	491.3597	0.4231	6.37E-05	0.002849	odorant receptor 45a-like
LOC101895706	3384.765	2931.915	0.20721	0.002365	0.046668	sodium leak channel non-selective protein, transcript variant X3
LOC101895719	7135.957	5777.332	0.3047	1.11E-06	9.38E-05	band 3 anion transport protein, transcript variant X1
LOC101895735	433.6032	639.01	-0.55946	3.60E-07	3.45E-05	cuticle protein CP14.6
LOC101895776	2492.747	2069.578	0.2684	0.000203	0.007256	set1/Ash2 histone methyltransferase complex subunit ASH2, transcript variant X1
LOC101895800	865.4458	666.1772	0.37754	9.36E-05	0.003851	uncharacterized LOC101895800, transcript variant X2
LOC101895836	241.7818	338.1366	-0.4839	0.000453	0.013355	uncharacterized LOC101895836
LOC101895843	685.6138	524.1813	0.38733	0.000191	0.00694	proliferating cell nuclear antigen
LOC101895846	3588.995	4187.448	-0.22249	0.002509	0.048726	serine/threonine-protein phosphatase 2A 56 kDa regulatory subunit

						epsilon isoform, transcript variant X4
LOC101895860	301.8633	422.3928	-0.48469	0.000912	0.023104	locomotion-related protein Hikaru genki
LOC101895885	373.4262	107.7636	1.793	6.94E-30	7.53E-27	troponin I, transcript variant X5
LOC101895915	12064.21	14350.44	-0.25036	2.71E-05	0.001389	cytochrome P450 4e3-like
LOC101895985	1284.803	1031.192	0.31723	0.000205	0.007293	serine/arginine repetitive matrix protein 2
LOC101895989	631.3491	798.1015	-0.33813	0.000651	0.017604	serine protease inhibitor 77Ba
LOC101895991	1487.259	1827.541	-0.29725	0.000222	0.007819	probable proteasome subunit beta type-2
LOC101896002	86.63473	146.7601	-0.76044	0.0003	0.009863	ataxin-2 homolog
LOC101896011	6201.651	7838.896	-0.338	5.00E-08	5.87E-06	uncharacterized LOC101896011
LOC101896015	1391.315	1719.114	-0.30522	8.96E-05	0.003731	sphingosine kinase 2
LOC101896017	125.625	67.24229	0.90168	8.65E-05	0.00362	DNA replication licensing factor Mcm6
LOC101896043	3424.99	4520.481	-0.40038	2.23E-07	2.22E-05	zinc-type alcohol dehydrogenase-like protein C1773.06c, transcript variant X3
LOC101896070	62.70787	115.4339	-0.88035	0.000264	0.008998	uncharacterized LOC101896070, transcript variant X1
LOC101896081	2071.707	2656.312	-0.3586	5.47E-07	5.00E-05	cytochrome P450 4e2
LOC101896085	288.4435	403.6523	-0.48482	0.001756	0.037972	protein disconnected
LOC101896105	447.1161	320.4766	0.48043	0.000622	0.017001	GTP-binding protein Di-Ras2
LOC101896112	2640.712	2241.521	0.23645	0.000913	0.023104	uncharacterized LOC101896112
LOC101896119	165.0873	25.70073	2.6833	1.10E-24	8.69E-22	uncharacterized LOC101896119
LOC101896155	2774.226	3745.69	-0.43315	1.94E-05	0.001049	protein scylla
LOC101896172	493.4219	724.8245	-0.55481	1.05E-07	1.17E-05	facilitated trehalose transporter Tret1, transcript variant X4
LOC101896174	1810.908	1387.472	0.38425	1.44E-05	0.000807	endoplasmic reticulum resident protein 44, transcript variant X1
LOC101896175	754.4042	1182.414	-0.64833	1.00E-05	0.000596	fatty acyl-CoA reductase wat
LOC101896184	2241.445	1875.357	0.25726	0.000549	0.015572	venom serine carboxypeptidase, transcript variant X1
LOC101896204	1100.124	1558.809	-0.50278	7.34E-10	1.27E-07	probable cytochrome P450 6v1
LOC101896218	1470.001	1878.867	-0.35405	4.98E-06	0.000322	tyrosine 3-monooxygenase
LOC101896220	223.3521	134.7186	0.72937	2.68E-05	0.001379	probable dolichyl pyrophosphate Glc1Man9GlcNAc2 alpha-1,3-glucosyltransferase

LOC101896232	1323.643	1568.882	-0.24522	0.002278	0.045512	bleomycin hydrolase
LOC101896243	202.1129	136.9141	0.56189	0.001383	0.031802	glutamyl-tRNA(Gln) amidotransferase subunit B, mitochondrial
LOC101896258	3695.823	3061.819	0.27151	0.001572	0.034996	troponin C, skeletal muscle-like, transcript variant X2
LOC101896301	96.47113	162.0632	-0.74839	0.000235	0.008223	uncharacterized LOC101896301
LOC101896347	380.6064	618.2471	-0.69988	1.53E-09	2.46E-07	vesicular inhibitory amino acid transporter, transcript variant X1
LOC101896443	2132.616	1698.961	0.32797	1.31E-05	0.000749	nucleolar GTP-binding protein 2
LOC101896469	3672.62	5793.697	-0.65767	4.21E-24	2.92E-21	probable cytochrome P450 313a4, transcript variant X1
LOC101896481	280.2258	185.6299	0.59416	9.47E-05	0.003874	cation-independent mannose-6-phosphate receptor
LOC101896485	2721.549	3178.591	-0.22396	0.001152	0.027865	uncharacterized LOC101896485, transcript variant X2
LOC101896501	11824.11	9118.872	0.3748	4.89E-10	8.65E-08	heat shock protein 83
LOC101896512	2240.617	1884.992	0.24934	0.000728	0.019222	phosphatidylinositol 4-phosphate 3-kinase C2 domain-containing subunit beta, transcript variant X1
LOC101896513	1839.854	1463.461	0.33021	2.06E-05	0.001104	acyl-CoA synthetase family member 4 homolog, transcript variant X7
LOC101896533	1423.147	1133.038	0.32889	6.19E-05	0.002806	uncharacterized LOC101896533
LOC101896535	13.30232	64.82198	-2.2848	1.57E-06	0.000126	glycine, alanine and asparagine-rich protein-like
LOC101896546	476.2647	939.8725	-0.9807	1.02E-22	6.10E-20	uncharacterized LOC101896546
LOC101896549	2902.442	3493.506	-0.26741	7.27E-05	0.003181	epsin-2, transcript variant X5
LOC101896555	66.26848	128.42	-0.95447	0.000258	0.008875	segmentation protein Runt, transcript variant X1
LOC101896594	2986.866	4510.384	-0.59462	0.000597	0.016523	eukaryotic translation initiation factor 4E-binding protein 3
LOC101896601	7977.151	6493.96	0.29677	1.55E-06	0.000125	uncharacterized LOC101896601
LOC101896604	1448.854	1204.746	0.26618	0.001046	0.025839	CTL-like protein 2
LOC101896617	2277.706	1789.318	0.34817	2.06E-06	0.000157	uncharacterized LOC101896617
LOC101896620	2452.304	3167.946	-0.36941	9.04E-08	1.01E-05	neural/ectodermal development factor IMP-L2, transcript variant X1
LOC101896659	297.6959	491.1194	-0.72223	4.74E-09	6.74E-07	mucin-5AC, transcript variant X2
LOC101896714	2684.258	3210.539	-0.25829	0.000208	0.007396	beta-parvin
LOC101896716	3.344919	17.3483	-2.3748	0.002061	0.042543	ctenidin-1
LOC101896718	61.5616	111.3965	-0.8556	0.000429	0.012859	inner centromere protein, transcript variant X1
LOC101896744	1634.704	1990.259	-0.28393	0.00017	0.006318	mucin-17, transcript variant X2

LOC101896797	1585.792	1218.256	0.38039	2.63E-06	0.000191	poly [ADP-ribose] polymerase
LOC101896810	4161.278	3134.393	0.40884	0.000158	0.005959	60 kDa heat shock protein, mitochondrial
LOC101896817	52.01296	4.506772	3.5287	2.28E-12	5.50E-10	uncharacterized LOC101896817
LOC101896845	69.74529	0.653058	6.7387	1.07E-25	8.82E-23	uncharacterized LOC101896845
LOC101896879	1361.191	1042.399	0.38496	4.21E-06	0.000282	sodium-independent sulfate anion transporter, transcript variant X1
LOC101896887	399.8599	512.5676	-0.35825	0.001741	0.037783	asparagine synthetase domain-containing protein CG17486
LOC101896895	5959.977	7679.208	-0.36565	3.50E-09	5.10E-07	heterogeneous nuclear ribonucleoprotein 87F
LOC101896929	4428.745	5388.578	-0.28301	2.65E-05	0.001366	cofilin/actin-depolymerizing factor homolog
LOC101896936	633.435	501.2641	0.33763	0.001679	0.036727	mesencephalic astrocyte-derived neurotrophic factor homolog
LOC101896960	614.393	454.0809	0.43621	5.96E-05	0.002713	uncharacterized LOC101896960
LOC101896983	1195.83	906.1262	0.40023	4.71E-06	0.00031	trithorax group protein osa
LOC101896989	8.735018	0	Inf	0.000448	0.013335	uncharacterized LOC101896989
LOC101897022	228.8057	160.9822	0.50722	0.002443	0.047666	probable cytochrome P450 6a21
LOC101897026	320.7796	217.2677	0.56211	8.37E-05	0.003563	nuclear pore complex protein Nup133
LOC101897046	6534.355	7596.936	-0.21737	0.000419	0.01264	protein ref(2)P
LOC101897075	823.0686	1030.752	-0.32461	0.000775	0.020156	CD109 antigen
LOC101897084	588.5567	855.8908	-0.54025	4.34E-08	5.16E-06	alpha-tocopherol transfer protein
LOC101897093	203.5775	133.9191	0.60422	0.000691	0.018454	angiotensin-converting enzyme, transcript variant X4
LOC101897104	2036.727	1700.177	0.26057	0.000595	0.016503	peptidyl-alpha-hydroxyglycine alpha-amidating lyase 1
LOC101897125	989.6673	746.9289	0.40597	1.11E-05	0.000653	serine-rich adhesin for platelets
LOC101897143	224.4872	118.2149	0.92522	1.77E-07	1.83E-05	uncharacterized LOC101897143
LOC101897154	8567.995	9794.837	-0.19306	0.001525	0.034396	esterase B1
LOC101897160	2148.467	2571.758	-0.25945	0.000284	0.009461	26S protease regulatory subunit 4
LOC101897191	223.2076	476.0664	-1.0928	0.000371	0.011636	uncharacterized LOC101897191
LOC101897200	233.8001	41.83233	2.4826	9.11E-32	1.32E-28	leucine-rich repeat extensin-like protein 1
LOC101897244	1181.592	861.5005	0.45581	2.65E-07	2.61E-05	proline-rich extensin-like protein EPR1
LOC101897289	235.3561	437.0204	-0.89286	1.34E-11	2.99E-09	cytosolic carboxypeptidase 6
LOC101897307	1204.898	899.4605	0.42178	2.59E-06	0.000189	sodium-dependent nutrient amino acid transporter 1, transcript

						variant X2
LOC101897314	192.28	313.9392	-0.70728	1.79E-06	0.00014	heat shock protein 23
LOC101897332	2005.767	2449.571	-0.28838	7.69E-05	0.003323	proteasome subunit alpha type-7-1
LOC101897335	1739.645	2190.345	-0.33237	7.73E-06	0.000471	mucin-5AC, transcript variant X1
LOC101897352	2581.734	1962.24	0.39584	1.52E-06	0.000124	cystathionine beta-synthase
LOC101897386	1672.941	2061.352	-0.3012	5.84E-05	0.002669	ATP-binding cassette sub-family G member 1, transcript variant X2
LOC101897392	710.31	540.8653	0.39318	0.000156	0.005899	protein PTCD3 homolog, mitochondrial
LOC101897406	1348.722	1596.156	-0.24301	0.002142	0.043958	cytochrome b561 domain-containing protein 2-like, transcript variant X1
LOC101897422	963.6405	762.057	0.3386	0.000251	0.008649	G-protein coupled receptor Mth2, transcript variant X2
LOC101897428	573.2043	410.3805	0.48209	1.48E-05	0.000825	phosphatidylserine decarboxylase proenzyme, mitochondrial, transcript variant X2
LOC101897439	22.47985	0.970354	4.534	2.63E-07	2.61E-05	uncharacterized LOC101897439
LOC101897451	130.6145	65.48155	0.99615	1.47E-05	0.000822	angiotensin-converting enzyme
LOC101897456	416.9796	562.0239	-0.43065	0.002243	0.04539	apolipoprotein D
LOC101897501	1434.817	1731.25	-0.27095	0.000515	0.014883	esterase B1
LOC101897516	1768.923	1496.148	0.24162	0.001771	0.038253	lysozyme
LOC101897522	4405.704	5334.796	-0.27606	1.95E-05	0.001049	scavenger receptor class B member 1
LOC101897526	3653.295	4406.946	-0.27058	3.97E-05	0.001906	probable serine/threonine-protein kinase DDB_G0282963
LOC101897551	17736.09	20819.9	-0.23127	0.000181	0.00668	ATP-binding cassette sub-family G member 1
LOC101897586	884.9886	691.2144	0.35653	0.000221	0.00781	dual specificity tyrosine-phosphorylation-regulated kinase 2
LOC101897587	154.4161	479.3669	-1.6343	1.17E-31	1.56E-28	cytochrome P450 4e2-like
LOC101897607	6.107525	24.1675	-1.9844	0.001101	0.026843	sperm-associated antigen 1
LOC101897655	45.97661	133.039	-1.5329	2.83E-10	5.39E-08	heat shock protein 27
LOC101897660	1648.107	2071.564	-0.32991	1.00E-05	0.000596	vasotab
LOC101897669	1144.433	808.3517	0.50158	1.78E-08	2.26E-06	cytochrome P450 4d2
LOC101897673	755.5291	557.4982	0.43852	1.42E-05	0.0008	laminin subunit alpha
LOC101897678	1488.174	1818.972	-0.28958	0.000184	0.006754	solute carrier family 25 member 36, transcript variant X2

LOC101897689	38.26813	90.12117	-1.2357	1.39E-05	0.000789	open rectifier potassium channel protein 1
LOC101897708	1095.298	903.7088	0.27739	0.001979	0.041337	eukaryotic peptide chain release factor subunit 1
LOC101897719	471.5506	604.3346	-0.35793	0.001014	0.025193	band 7 protein AGAP004871-like
LOC101897741	274.5065	160.6417	0.77299	4.83E-06	0.000316	tropomyosin-2, transcript variant X1
LOC101897764	2581.763	2077.528	0.31349	1.20E-05	0.000698	probable phospholipid-transporting ATPase IIB, transcript variant X1
LOC101897767	1906.812	1228.97	0.63371	7.25E-16	2.74E-13	protein disulfide-isomerase A6 homolog, transcript variant X2
LOC101897774	20027.47	24763.98	-0.30626	1.52E-07	1.61E-05	latrophilin Cirl
LOC101897776	703.3455	564.3854	0.31755	0.002114	0.043499	protein FRA10AC1 homolog
LOC101897795	12349.9	14509.9	-0.23254	9.27E-05	0.003832	NADH-cytochrome b5 reductase 3, transcript variant X3
LOC101897803	3264.134	4273.139	-0.3886	5.44E-09	7.62E-07	ctenidin-1
LOC101897804	382.5488	518.1634	-0.43776	0.000151	0.005754	probable serine hydrolase, transcript variant X7
LOC101897810	3463.145	3986.943	-0.2032	0.002267	0.045512	E3 ubiquitin-protein ligase Nedd-4, transcript variant X6
LOC101897812	757.2953	568.5798	0.41349	4.25E-05	0.002029	ribonucleoside-diphosphate reductase subunit M2
LOC101897823	376.4543	255.8779	0.55702	3.27E-05	0.001613	sodium-dependent nutrient amino acid transporter 1
LOC101897841	1976.757	1584.344	0.31925	4.82E-05	0.002261	cytochrome P450 4d2
LOC101897852	2198.297	1647.749	0.41589	2.95E-08	3.61E-06	uncharacterized LOC101897852
LOC101897892	107.1844	172.2249	-0.6842	0.000428	0.012855	extensin, transcript variant X2
LOC101897901	900.4227	1095.459	-0.28286	0.001456	0.033086	uncharacterized LOC101897901, transcript variant X2
LOC101897924	114.8711	205.7024	-0.84054	3.41E-06	0.000236	Hemolymph juvenile hormone binding protein (JHBP), transcript variant X1
LOC101897955	173.4681	103.4352	0.74594	0.00014	0.005424	single-pass membrane and coiled-coil domain-containing protein 4 homolog
LOC101897984	785.5569	953.9563	-0.28021	0.00231	0.045832	zinc transporter 2, transcript variant X5
LOC101898013	141.4088	276.9565	-0.96979	1.91E-09	3.01E-07	uncharacterized LOC101898013
LOC101898023	53466.92	45215.41	0.24183	2.43E-05	0.001276	uncharacterized LOC101898023
LOC101898032	315.778	211.0141	0.58157	5.54E-05	0.00255	DNA fragmentation factor subunit beta, transcript variant X2
LOC101898033	1184.916	982.0769	0.27088	0.001749	0.037901	probable G-protein coupled receptor Mth-like 8
LOC101898060	4158.122	3366.097	0.30486	4.84E-06	0.000316	mucin-5AC

LOC101898074	1393.116	1653.546	-0.24725	0.002413	0.047282	annexin B10
LOC101898080	662.3409	528.8526	0.32471	0.001777	0.038274	DET1- and DDB1-associated protein 1
LOC101898107	871.0669	1911.086	-1.1335	1.93E-43	4.78E-40	cell division cycle 7-related protein kinase
LOC101898132	385.268	563.4383	-0.5484	1.58E-06	0.000126	fatty acyl-CoA reductase wat
LOC101898144	287.5508	436.9666	-0.60371	1.89E-06	0.000148	small nuclear ribonucleoprotein Sm D3, transcript variant X2
LOC101898179	804.0596	635.5287	0.33935	0.000561	0.015776	cysteine-rich with EGF-like domain protein 2, transcript variant X1
LOC101898188	2554.294	2012.442	0.34398	2.01E-06	0.000155	pre-rRNA-processing protein TSR1 homolog
LOC101898205	329.5768	175.1251	0.91223	8.32E-10	1.43E-07	paramyosin, long form
LOC101898207	586.6631	356.8315	0.71729	3.35E-10	6.32E-08	uncharacterized LOC101898207
LOC101898215	5163.899	4263.62	0.27638	1.60E-05	0.000882	periostin, transcript variant X2
LOC101898217	234.2103	387.4353	-0.72615	1.06E-06	9.04E-05	venom dipeptidyl peptidase 4
LOC101898222	741.2871	556.4161	0.41387	0.002385	0.046839	probable 3-hydroxyisobutyrate dehydrogenase, mitochondrial
LOC101898242	2952.808	2439.661	0.27541	8.58E-05	0.003606	LETM1 domain-containing protein 1
LOC101898268	816.7036	660.1306	0.30706	0.001664	0.03649	alpha-mannosidase 2
LOC101898280	215.098	303.4424	-0.49643	0.001738	0.037783	major heat shock 70 kDa protein Ba-like
LOC101898324	35233.27	30958.22	0.18662	0.001209	0.028796	very long-chain-fatty-acid--CoA ligase bubblegum
LOC101898381	971.9699	1204.583	-0.30955	0.00038	0.011797	uncharacterized LOC101898381
LOC101898399	828.3149	1044.26	-0.33423	0.000233	0.008172	probable serine/threonine-protein kinase tsuA
LOC101898412	1433.959	1147.256	0.32182	0.000106	0.004272	von Willebrand factor A domain-containing protein 8
LOC101898434	455.6012	348.041	0.38851	0.001471	0.03334	RUN domain-containing protein 1
LOC101898467	204.0064	141.038	0.53253	0.00225	0.045418	mesocentin, transcript variant X1
LOC101898506	626.0043	437.6564	0.51637	2.40E-06	0.00018	nuclear cap-binding protein subunit 1
LOC101898526	723.3242	1238.544	-0.77593	7.68E-13	2.02E-10	esterase B1, transcript variant X1
LOC101898530	8831.323	10041.58	-0.18529	0.002407	0.047217	ferritin subunit
LOC101898563	57.73227	153.5392	-1.4112	4.01E-10	7.32E-08	alpha-tocopherol transfer protein-like
LOC101898586	1446.06	1187.529	0.28416	0.000461	0.01354	pre-mRNA-processing factor 6
LOC101898592	1512.185	1176.701	0.36189	9.79E-06	0.000586	sodium/hydrogen exchanger 8
LOC101898640	9175.541	6594.875	0.47645	1.06E-14	3.22E-12	ecdysteroid-regulated 16 kDa protein

LOC101898659	215.2661	117.3176	0.8757	2.02E-06	0.000155	exonuclease 1
LOC101898672	2872.732	3698.247	-0.36442	7.16E-08	8.18E-06	ankyrin repeat domain-containing protein 39
LOC101898698	244.5086	412.0616	-0.75297	1.12E-08	1.46E-06	esterase B1-like
LOC101898708	2964.934	3749.465	-0.33868	5.75E-07	5.20E-05	probable pseudouridine-5'-phosphatase, transcript variant X3
LOC101898711	1292.814	1680.311	-0.37821	0.000278	0.009366	elongation of very long chain fatty acids protein AAEL008004, transcript variant X1
LOC101898732	14.31452	63.96204	-2.1597	3.40E-05	0.001672	coiled-coil domain-containing protein 42 like-2-like
LOC101898738	2820.018	1938.772	0.54056	2.51E-11	5.53E-09	major heat shock 70 kDa protein Ab-like
LOC101898756	130.6237	196.4652	-0.58886	0.000938	0.023644	dynein intermediate chain 2, axonemal
LOC101898779	1605.034	1340.176	0.26018	0.001075	0.026389	uncharacterized LOC101898779
LOC101898780	1351.185	1653.219	-0.29105	0.000241	0.008387	glycerol-3-phosphate acyltransferase 3, transcript variant X1
LOC101898798	707.7858	548.7004	0.36729	0.000335	0.010712	maltase 2, transcript variant X2
LOC101898830	4281.483	2913.442	0.55539	1.35E-16	5.44E-14	uncharacterized LOC101898830
LOC101898838	365.8496	271.0271	0.43281	0.001268	0.029701	MICOS complex subunit MIC27
LOC101898847	12.35337	83.93167	-2.7643	3.08E-05	0.001541	flightin
LOC101898875	1604.745	1897.137	-0.24148	0.001571	0.034996	type-1 angiotensin II receptor-associated protein
LOC101898908	30.18234	63.59546	-1.0752	0.001319	0.030527	protein dispatched
LOC101898939	3825.91	3313.545	0.20743	0.002059	0.042543	transmembrane protein 189, transcript variant X1
LOC101898969	4.582678	21.49142	-2.2295	0.001042	0.02583	uncharacterized LOC101898969
LOC101898975	517.5741	691.5354	-0.41804	5.52E-05	0.00255	probable multidrug resistance-associated protein lethal(2)03659
LOC101898982	2070.321	2466.457	-0.25259	0.000481	0.014074	uncharacterized LOC101898982
LOC101899021	1289.439	1017.293	0.34201	5.97E-05	0.002713	probable phospholipid-transporting ATPase VA, transcript variant X2
LOC101899057	620.3245	785.3565	-0.34032	0.000618	0.016924	uncharacterized LOC101899057
LOC101899080	2061.347	2458.904	-0.25443	0.000449	0.013335	proteasome subunit beta type-5
LOC101899101	108.9795	169.0085	-0.63304	0.001123	0.027313	protein yellow
LOC101899135	722.6882	978.2595	-0.43684	2.40E-06	0.00018	cytochrome P450 6d1-like
LOC101899169	5404.956	3454.057	0.64599	5.61E-23	3.48E-20	endoplasmin homolog
LOC101899237	163.6088	233.5128	-0.51325	0.001572	0.034996	neuropeptide-like 4

LOC101899245	94.5108	52.58853	0.84573	0.001317	0.030527	lectin subunit alpha-like
LOC101899286	75.11702	36.54654	1.0394	0.000529	0.015145	extensin
LOC101899302	20980.56	18336.97	0.1943	0.000879	0.022553	farnesol dehydrogenase
LOC101899456	1091.76	758.1275	0.52614	9.01E-05	0.003744	kinesin-associated protein 3, transcript variant X2
LOC101899458	33.29746	73.06343	-1.1337	0.000297	0.009793	acetylcholine receptor subunit alpha-L1, transcript variant X1
LOC101899460	1008.749	1248.703	-0.30786	0.000315	0.010258	uncharacterized LOC101899460
LOC101899490	62.26341	115.3392	-0.88943	0.000196	0.007098	hypothetical protein
LOC101899531	4.928995	20.85741	-2.0812	0.002114	0.043499	peptide transporter family 1, transcript variant X1
LOC101899539	475.5234	314.0512	0.59852	1.03E-06	8.89E-05	splicing factor U2AF 50 kDa subunit
LOC101899617	3534.984	4225.644	-0.25747	9.53E-05	0.003885	choline/ethanolamine kinase, transcript variant X1
LOC101899618	731.9158	591.3866	0.30758	0.002515	0.0488	serine-rich adhesin for platelets
LOC101899627	1252.534	1534.693	-0.2931	0.000308	0.010069	uncharacterized LOC101899627
LOC101899679	349.2041	482.7181	-0.46711	0.000112	0.004465	uncharacterized LOC101899679
LOC101899757	24065.11	29720.08	-0.3045	1.55E-07	1.63E-05	tubulin beta-1 chain
LOC101899767	47.39907	111.6147	-1.2356	1.13E-06	9.57E-05	myosin-9
LOC101899804	1693.875	2193.088	-0.37264	6.51E-06	0.000405	farnesol dehydrogenase
LOC101899813	2735.462	2223.159	0.29917	2.58E-05	0.001337	probable serine/threonine-protein kinase DDB_G0282963, transcript variant X3
LOC101899820	131.7697	82.66482	0.67267	0.002256	0.045436	beta-1,3-galactosyltransferase 6
LOC101899852	854.0292	648.7033	0.39673	4.41E-05	0.002087	cyclic nucleotide-gated cation channel beta-3
LOC101899866	8098.931	7101.219	0.18966	0.001871	0.039852	phospholipid-transporting ATPase ID
LOC101899878	221.1553	113.3985	0.96366	6.06E-08	6.97E-06	titin, transcript variant X1
LOC101899879	1558.712	1292.777	0.26988	0.000746	0.019638	uncharacterized LOC101899879
LOC101899907	171.344	260.9578	-0.60692	0.000121	0.004802	uncharacterized LOC101899907
LOC101899975	406.2804	305.7994	0.40989	0.001282	0.029908	dynein assembly factor 5, axonemal, transcript variant X2
LOC101899990	1760.587	2071.499	-0.23462	0.001678	0.036727	proteasome subunit alpha type-1
LOC101900012	1517.653	1805.559	-0.2506	0.00253	0.049029	mitochondrial folate transporter/carrier
LOC101900017	2671.02	3151.652	-0.23872	0.000557	0.015755	very-long-chain (3R)-3-hydroxyacyl-CoA dehydratase hpo-8

LOC101900064	44.78568	12.93053	1.7923	3.89E-05	0.00188	uncharacterized LOC101900064
LOC101900092	577.48	437.6969	0.39984	0.000342	0.010861	apoptosis-resistant E3 ubiquitin protein ligase 1
LOC101900118	657.6234	866.4287	-0.39782	9.30E-05	0.003835	uncharacterized LOC101900118
LOC101900136	963.3327	1336.031	-0.47185	2.75E-08	3.39E-06	aromatic-L-amino-acid decarboxylase, transcript variant X1
LOC101900142	31082.77	35128.2	-0.17651	0.002277	0.045512	circadian clock-controlled protein
LOC101900179	333.7547	242.0177	0.46368	0.000775	0.020156	serine/threonine-protein phosphatase 2A regulatory subunit B'' subunit gamma
LOC101900188	1300.826	1641.764	-0.33582	2.61E-05	0.001347	uncharacterized protein DDB_G0284459
LOC101900190	3806.951	3250.708	0.22788	0.000695	0.018481	protein kinase, transcript variant X3
LOC101900194	290.1316	395.9323	-0.44855	0.000615	0.016889	ras guanine nucleotide exchange factor Y
LOC101900224	2891.928	3433.386	-0.2476	0.000278	0.009366	uncharacterized LOC101900224, transcript variant X5
LOC101900236	1313.04	1013.656	0.37334	1.13E-05	0.00066	transmembrane protein 135, transcript variant X1
LOC101900255	17007.88	22629.05	-0.41197	1.98E-12	4.84E-10	alkaline phosphatase 4
LOC101900256	891.4191	1146.086	-0.36254	0.000963	0.024099	protein tyrosine phosphatase type IVA 1
LOC101900264	14749.14	12460.81	0.24323	0.000884	0.022604	putative ammonium transporter 3
LOC101900276	1868.01	2373.59	-0.34557	0.000771	0.020125	uncharacterized LOC101900276, transcript variant X2
LOC101900303	2124.326	3397.365	-0.67741	3.59E-22	2.01E-19	protein hairy
LOC101900306	1185.746	1417.849	-0.25791	0.001785	0.038361	potassium voltage-gated channel protein Shab, transcript variant X5
LOC101900313	229.5081	127.3158	0.85013	7.54E-07	6.61E-05	fatty acyl-CoA reductase wat
LOC101900324	425.9141	581.948	-0.45033	6.30E-05	0.002844	39S ribosomal protein L22, mitochondrial
LOC101900326	421.7498	612.1979	-0.53761	1.52E-06	0.000124	sperm-specific antigen 2
LOC101900334	2952.366	2417.914	0.28811	3.88E-05	0.00188	manganese-transporting ATPase 13A1
LOC101900336	138.2101	16.35968	3.0786	4.47E-26	3.88E-23	general odorant-binding protein 57c
LOC101900345	3194.954	4239.501	-0.4081	1.08E-09	1.79E-07	alpha-tocopherol transfer protein
LOC101900347	885.5106	1168.276	-0.3998	0.00056	0.015776	aldose reductase
LOC101900405	86.95625	45.09625	0.94728	0.000583	0.01626	eukaryotic translation initiation factor 5B
LOC101900415	237.8344	330.8958	-0.47642	0.000652	0.017617	DNA-binding protein D-ETS-6
LOC101900429	48.83072	96.38768	-0.98106	0.000525	0.015041	peptidyl-prolyl cis-trans isomerase-like

LOC101900430	515.8124	396.2455	0.38045	0.001606	0.035516	syndetin
LOC101900445	3101.194	3597.1	-0.21401	0.001557	0.034888	lipopolysaccharide-induced tumor necrosis factor-alpha factor homolog, transcript variant X1
LOC101900457	6487.843	7464.341	-0.20228	0.001169	0.02808	Na(+)/H(+) exchange regulatory cofactor NHE-RF1, transcript variant X1
LOC101900470	2296.736	2768.282	-0.26941	0.000148	0.005674	cold shock domain-containing protein E1
LOC101900474	504.9646	393.292	0.36058	0.001824	0.039054	probable cleavage and polyadenylation specificity factor subunit 2
LOC101900491	3421.777	2797.499	0.29061	2.26E-05	0.0012	juvenile hormone epoxide hydrolase 1
LOC101900498	5378.909	4612.955	0.22162	0.000554	0.015687	leukocyte receptor cluster member 8 homolog
LOC101900502	2320.12	1950.709	0.2502	0.0007	0.018575	uncharacterized LOC101900502
LOC101900504	345.9752	231.9309	0.57697	3.51E-05	0.001717	homeobox protein unc-4
LOC101900507	12.25075	1.278416	3.2604	0.001913	0.040508	general odorant-binding protein 57c-like
LOC101900593	12.01796	40.59221	-1.756	0.000122	0.004819	uncharacterized LOC101900593
LOC101900623	3565.608	4134.955	-0.21372	0.001298	0.03017	chromosome-associated kinesin KIF4A, transcript variant X1
LOC101900625	1171.616	915.9276	0.35519	4.63E-05	0.002183	zinc finger protein on ecdysone puffs, transcript variant X1
LOC101900631	330.1455	234.471	0.49369	0.000418	0.01264	putative DNA helicase Ino80
LOC101900655	236.2326	158.7153	0.57377	0.000598	0.016534	UPF0047 protein YjbQ
LOC101900656	46.99697	87.87571	-0.9029	0.000911	0.023104	mucin-5AC
LOC101900684	129.751	11.66135	3.4759	5.58E-28	5.38E-25	general odorant-binding protein 57c-like
LOC101900721	80072.47	103668	-0.37259	5.01E-11	1.07E-08	uncharacterized LOC101900721, transcript variant X2
LOC101900725	988.7632	1301.548	-0.39653	3.22E-06	0.000225	putative fatty acyl-CoA reductase CG5065
LOC101900759	1492.372	1201.917	0.31227	0.000113	0.004487	protein-L-isoaspartate(D-aspartate) O-methyltransferase
LOC101900767	190.2908	264.485	-0.47498	0.00213	0.043761	uncharacterized LOC101900767
LOC101900775	1954.523	2325.929	-0.25099	0.000565	0.015845	endothelin-converting enzyme 1, transcript variant X5
LOC101900777	518.2973	685.8457	-0.4041	0.000149	0.005705	protein borderless, transcript variant X2
LOC101900779	12331.11	10571.44	0.22213	0.000192	0.006971	secernin-2, transcript variant X2
LOC101900785	10593.27	8701.453	0.28382	2.89E-06	0.000207	sterol regulatory element-binding protein 1, transcript variant X1
LOC101900862	36.85016	8.469728	2.1213	2.31E-05	0.001224	general odorant-binding protein 57c-like

LOC101900866	1794.151	1518.522	0.24063	0.001751	0.037901	CCR4-NOT transcription complex subunit 3, transcript variant X2
LOC101900869	625.0929	478.7091	0.38492	0.000359	0.011317	probable multidrug resistance-associated protein lethal(2)03659
LOC101900906	22307.69	18610	0.26146	7.66E-06	0.000469	probable cytochrome P450 308a1
LOC101900914	21.10378	56.92341	-1.4315	7.91E-05	0.00341	EF-hand domain-containing family member C2, transcript variant X2
LOC101900934	16464.15	14140.8	0.21946	0.00042	0.012644	alcohol dehydrogenase
LOC101900945	3741.162	3234.902	0.20976	0.001923	0.040573	alpha-(1,3)-fucosyltransferase C
LOC101900973	1101.876	1879.761	-0.77059	8.48E-19	3.98E-16	NACHT and WD repeat domain-containing protein 2, transcript variant X2
LOC101901001	458.4725	627.0905	-0.45184	0.000995	0.02478	ubiquitin-conjugating enzyme E2 E1, transcript variant X1
LOC101901110	282.4371	385.0854	-0.44725	0.000675	0.018075	H/ACA ribonucleoprotein complex subunit 4
LOC101901113	593.393	1057.743	-0.83393	1.08E-18	4.79E-16	inositol-trisphosphate 3-kinase homolog
LOC101901144	1145.873	1450.579	-0.34018	3.44E-05	0.00169	protein pigeon, transcript variant X1
LOC101901154	548.3073	366.6529	0.58057	5.46E-07	5.00E-05	indole-3-acetaldehyde oxidase, transcript variant X2
LOC101901176	383.5527	536.0482	-0.48294	2.80E-05	0.001425	protein turtle
LOC101901189	642.7983	510.5842	0.33222	0.001866	0.039801	uncharacterized LOC101901189, transcript variant X2
LOC101901198	7413.645	6481.622	0.19383	0.001739	0.037783	multidrug resistance-associated protein 4
LOC101901237	2088.194	2628.908	-0.33221	3.24E-06	0.000226	heat shock factor protein, transcript variant X7
LOC101901242	4084.969	3518.19	0.21549	0.00119	0.028506	E3 ubiquitin-protein ligase MARCH6
LOC101901250	658.2419	489.016	0.42874	5.02E-05	0.002342	S-formylglutathione hydrolase
LOC101901267	432.8988	304.4687	0.50774	5.64E-05	0.00259	probable cysteine--tRNA ligase, mitochondrial
LOC101901286	3503.003	4124.317	-0.23556	0.000383	0.011843	sulfhydryl oxidase 2
LOC101901302	1515.299	2002.904	-0.40249	1.31E-07	1.40E-05	arginine/serine-rich coiled-coil protein 2, transcript variant X1
LOC101901327	678.7833	505.8213	0.42432	6.42E-05	0.002868	odorant receptor 82a
LOC101901338	1084.719	836.5593	0.37478	3.00E-05	0.001512	ATP-binding cassette sub-family F member 3
LOC101901341	3767.289	3083.062	0.28916	1.89E-05	0.001028	uncharacterized LOC101901341
LOC101901407	2538.475	2177.372	0.22137	0.001988	0.041386	hypoxia up-regulated protein 1
LOC101901418	4563.029	3842.799	0.24783	0.000164	0.006126	low density lipoprotein receptor adapter protein 1-A
LOC101901452	227.4084	373.6326	-0.71634	9.92E-07	8.57E-05	beta-1,3-glucosyltransferase

LOC101901505	2434.287	1915.414	0.34584	1.91E-06	0.000148	CAD protein
LOC101901557	120.8157	62.33473	0.9547	5.04E-05	0.002347	gustatory receptor for sugar taste 64f
LOC101901575	581.2463	793.5578	-0.44919	6.55E-06	0.000406	serine/threonine-protein kinase ATM
LOC101901606	966.0641	1178.396	-0.28663	0.000942	0.023696	histone deacetylase Rpd3
LOC101901620	1071.747	876.7523	0.28972	0.001228	0.029017	WD repeat-containing protein 7
LOC101901632	2239.107	2797.232	-0.32108	5.78E-06	0.000366	RING-variant domain protein, transcript variant X2
LOC101901642	1341.686	1085.877	0.30519	0.000263	0.008998	solute carrier family 35 member F6
LOC101901643	1640.865	1187.478	0.46656	5.81E-08	6.74E-06	probable cytochrome P450 4ac1, transcript variant X1
LOC101901661	738.9072	597.4892	0.30648	0.002246	0.045405	activator of 90 kDa heat shock protein ATPase homolog 1
LOC101901668	1603.677	1353.313	0.24489	0.001876	0.03987	hypothetical protein, transcript variant X1
LOC101901673	365.2669	495.2615	-0.43924	0.000185	0.006762	cartilage oligomeric matrix protein, transcript variant X2
LOC101901690	419.948	588.4413	-0.48669	1.28E-05	0.000736	retinol-binding protein pinta
LOC101901702	7564.724	6421.876	0.23629	0.000296	0.009787	general odorant-binding protein 84a
LOC101901731	301.9645	497.1236	-0.71923	4.02E-09	5.77E-07	thiamine transporter 1
LOC101901732	1.304449	13.50363	-3.3718	0.000903	0.02301	extensin-2
LOC101901744	1826.228	2142.066	-0.23014	0.001895	0.040174	sodium-dependent nutrient amino acid transporter 1
LOC101901753	136.197	52.29755	1.3809	8.86E-08	9.99E-06	luciferin 4-monooxygenase-like
LOC101901765	1207.08	994.9331	0.27885	0.001288	0.03002	kelch-like protein 5
LOC101901809	825.5472	603.33	0.4524	8.61E-05	0.003612	transmembrane protein 205, transcript variant X2
LOC105261445	547.9612	375.4734	0.54536	2.49E-06	0.000184	cyclic AMP-responsive element-binding protein 5
LOC105261580	573.8065	339.9498	0.75524	6.83E-11	1.43E-08	pickpocket protein 28-like
LOC105261583	921.4413	1489.635	-0.69299	1.67E-16	6.58E-14	uncharacterized LOC105261583, transcript variant X1
LOC105261589	600.7496	434.7697	0.46651	2.33E-05	0.001227	uncharacterized LOC105261589
LOC105261596	50.82352	96.88633	-0.9308	0.000431	0.012902	uncharacterized LOC105261596
LOC105261625	3136.911	3886.808	-0.30924	0.002277	0.045512	V-type proton ATPase subunit d 1
LOC105261647	441.0513	556.8153	-0.33625	0.002331	0.046108	WAS/WASL-interacting protein family member 1
LOC105261692	11.36722	44.12432	-1.9567	6.84E-05	0.003045	uncharacterized LOC105261692, transcript variant X9
LOC105261738	45.64418	19.26112	1.2447	0.001983	0.041337	uncharacterized LOC105261738

LOC105261794	202.5448	49.94132	2.0199	2.86E-21	1.50E-18	uncharacterized LOC105261794
LOC105261826	3275.69	2068.928	0.66292	4.45E-13	1.21E-10	putative ammonium transporter 3
LOC105261850	300.1233	220.6133	0.44404	0.002057	0.042543	uncharacterized LOC105261850
LOC105261855	124.9963	74.40785	0.74836	0.000808	0.02094	cytochrome P450 6a9-like
LOC105261919	364.2068	174.29	1.0633	2.65E-13	7.32E-11	uncharacterized LOC105261919
LOC105261934	301.8837	407.5597	-0.43302	0.000593	0.01649	transposable element Hobo transposase, transcript variant X1
LOC105262057	18.86001	0.979587	4.267	5.03E-06	0.000323	general odorant-binding protein 57c-like
LOC105262081	6.867074	27.81336	-2.018	0.000517	0.014883	turripeptide Lol9.1
LOC105262082	131.675	228.8824	-0.79763	3.07E-06	0.000217	uncharacterized LOC105262082
LOC105262120	14.62685	40.31657	-1.4628	0.000884	0.022604	uncharacterized LOC105262120
LOC105262123	5943.062	4766.312	0.31833	6.22E-07	5.60E-05	uncharacterized LOC105262123
LOC105262128	140.9244	217.9907	-0.62935	0.001638	0.035992	lipopolysaccharide-induced tumor necrosis factor-alpha factor homolog
LOC105262130	296.6663	120.2931	1.3023	1.55E-15	5.61E-13	uncharacterized LOC105262130
LOC105262200	3.733135	25.53669	-2.7741	2.48E-05	0.001291	uncharacterized LOC105262200
LOC105262206	62.25297	122.1981	-0.97301	4.36E-05	0.002073	uncharacterized LOC105262206
LOC105262305	2314.517	3358.575	-0.53714	7.59E-15	2.40E-12	uncharacterized LOC105262305, transcript variant X2
LOC105262475	71.79243	25.61999	1.4866	3.05E-05	0.001531	uncharacterized LOC105262475
LOC105262478	2157.42	3039.294	-0.49443	2.56E-12	6.08E-10	nucleic-acid-binding protein from mobile element jockey
LOC105262527	129.7718	75.11475	0.78881	0.002159	0.044098	uncharacterized LOC105262527, transcript variant X1
LOC109611626	182.565	30.17805	2.5968	1.02E-14	3.15E-12	glycine-rich protein 5-like
LOC109611756	187.9273	304.9937	-0.6986	2.51E-06	0.000184	uncharacterized LOC109611756
LOC109611821	649.0723	487.1223	0.4141	9.95E-05	0.004035	glutamate receptor 1-like
LOC109611874	29.28321	3.228162	3.1813	7.45E-07	6.57E-05	uncharacterized LOC109611874
LOC109612058	62.95455	107.4824	-0.77172	0.001937	0.040761	uncharacterized LOC109612058
LOC109612083	97.09365	53.11469	0.87027	0.000712	0.018878	uncharacterized LOC109612083
LOC109612265	0.358713	11.41675	-4.9922	0.000232	0.008156	cytochrome P450 4d1-like
LOC109612339	46.79665	17.70234	1.4025	0.000453	0.013355	uncharacterized LOC109612339

LOC109612357	607.4097	808.8234	-0.41315	3.05E-05	0.001531	uncharacterized protein K02A2.6-like
LOC109612490	198.6113	132.2332	0.58686	0.000961	0.024088	nuclear transcription factor Y subunit beta-like
LOC109612700	6080.291	7004.829	-0.20421	0.001975	0.04132	cytochrome P450 6d1
LOC109612803	16.31483	51.32781	-1.6536	2.78E-05	0.00142	uncharacterized LOC109612803
LOC109612900	1697.757	1234.786	0.45937	8.62E-09	1.18E-06	tryptophan--tRNA ligase, cytoplasmic-like
LOC109612950	102.2168	212.9994	-1.0592	9.56E-09	1.30E-06	uncharacterized LOC109612950, transcript variant X2
LOC109613077	1917.01	2345.124	-0.29081	7.57E-05	0.003278	uncharacterized LOC109613077, transcript variant X1
LOC109613080	185.7126	316.9666	-0.77126	0.000612	0.016834	uncharacterized LOC109613080
LOC109613422	889.0795	706.3434	0.33194	0.000456	0.013412	uncharacterized LOC109613422
LOC109613447	402.9191	285.0646	0.4992	0.000111	0.004438	uncharacterized LOC109613447
LOC109613501	30.74905	68.55764	-1.1568	0.000346	0.010979	putative uncharacterized protein DDB_G0291608
LOC109614140	143.5058	240.7216	-0.74626	9.58E-06	0.000576	uncharacterized LOC109614140
LOC109614162	159.4163	104.0698	0.61525	0.001983	0.041337	uncharacterized LOC109614162
LOC109614166	22.24683	5.85808	1.9251	0.002373	0.04667	uncharacterized LOC109614166
LOC109614175	131.7312	284.397	-1.1103	1.48E-10	2.89E-08	uncharacterized LOC109614175
LOC109614207	1322.606	1767.052	-0.41796	1.23E-07	1.34E-05	uncharacterized LOC109614207
Novel00023	639.0292	896.3516	-0.48818	0.002371	0.04667	-
Novel00034	613.4709	246.8685	1.3133	2.01E-27	1.83E-24	-
Novel00036	36.94808	73.71983	-0.99655	0.000937	0.023639	-
Novel00046	31.87009	10.70985	1.5733	0.001863	0.039779	-
Novel00138	337.2484	187.1224	0.84983	5.64E-09	7.83E-07	-
Novel00168	4.04995	86.35859	-4.4144	4.97E-24	3.32E-21	-
Novel00183	10.96341	31.96649	-1.5439	0.001629	0.035903	-
Novel00188	192.9756	122.7415	0.6528	0.000367	0.011543	-
Novel00311	189.0706	264.8608	-0.48631	0.001805	0.038693	-
Novel00340	47.7285	87.44586	-0.87354	0.001413	0.032331	-
Novel00376	12.94039	34.84042	-1.4289	0.002543	0.049164	-
Novel00483	159.5324	81.73296	0.96486	3.95E-06	0.000269	-

Novel00516	146.67	248.9287	-0.76316	3.86E-06	0.000265	-
Novel00593	794.724	1195.83	-0.58949	2.39E-05	0.001257	-
Novel00595	316.1298	231.0408	0.45237	0.001697	0.037055	-
Novel00628	350.4853	610.1831	-0.79989	0.001717	0.037451	-
Novel00634	29.4258	65.13202	-1.1463	0.00247	0.04809	-
Novel00705	571.4303	754.1737	-0.40032	6.36E-05	0.002849	-
Novel00728	14363.31	12367.27	0.21586	0.000282	0.009431	-
Novel00734	2.481491	17.89232	-2.8501	0.000295	0.009764	-
Novel00847	6.998967	26.80744	-1.9374	0.000823	0.021242	-
Novel00983	24.26893	60.4227	-1.316	0.000199	0.007151	-
Novel01002	211.1858	317.1975	-0.58687	0.000727	0.019218	-
Novel01006	7.750604	59.64782	-2.9441	0.001203	0.028682	-
Novel01217	103.1129	18.04714	2.5144	3.25E-15	1.13E-12	-
Novel01218	112.6835	27.23968	2.0485	2.44E-05	0.001277	-
Novel01231	158.5261	96.62285	0.71428	0.000448	0.013335	-
Novel01333	35.64855	72.21155	-1.0184	0.001064	0.026239	-
Novel01367	354.503	256.5891	0.46634	0.001955	0.040987	-
Novel01369	316.3498	231.3945	0.45117	0.001411	0.032322	-
Novel01394	9.427652	0	Inf	0.000238	0.008321	-
Novel01405	505.4485	345.6745	0.54815	4.07E-06	0.000274	-
Novel01443	11.56925	47.98295	-2.0522	0.001234	0.029059	-
Novel01461	12.99619	47.38468	-1.8663	0.000157	0.005923	-
Novel01548	92.51123	191.1696	-1.0472	0.001274	0.029781	-
Novel01677	123.2692	228.2062	-0.88853	0.000377	0.011751	-
Novel01764	328.8844	232.8458	0.49821	0.000385	0.011843	-
Novel01828	1501.523	934.4266	0.68427	4.12E-16	1.59E-13	-
Novel01878	511.407	644.6425	-0.33403	0.001483	0.033537	-
Novel01935	5367.988	6904.357	-0.36313	0.001401	0.032137	-

Novel02008	142.7539	207.1629	-0.53724	0.00226	0.045467	-
Novel02028	81.41995	150.5079	-0.88639	2.14E-05	0.001141	-
Novel02045	1665.768	2274.241	-0.4492	0.000165	0.00615	-
Novel02046	132.6661	205.023	-0.62799	0.000419	0.01264	-
Novel02055	134.7172	85.6134	0.65403	0.002298	0.045762	-
Novel02069	15.6611	46.22121	-1.5614	0.000171	0.006355	-
Novel02082	4.601272	29.78886	-2.6947	0.001553	0.034829	-
Novel02100	9.43385	119.3214	-3.6609	2.81E-21	1.50E-18	-
Novel02124	202.9565	287.3813	-0.5018	0.000835	0.021502	-
Novel02193	2363.634	2987.3	-0.33784	0.00033	0.010598	-
Novel02228	465.7217	334.7574	0.47635	8.78E-05	0.003664	-
Novel02242	894.6576	730.1479	0.29315	0.001967	0.041195	-
Novel02307	442.4583	332.9648	0.41017	0.001	0.024887	-
Novel02353	19.51373	3.899102	2.3233	0.001152	0.027865	-
Novel02355	84.31763	187.7541	-1.1549	4.81E-09	6.79E-07	-
Novel02365	135.9268	68.05723	0.99801	1.13E-05	0.00066	-
Novel02415	160.9161	60.13997	1.4199	1.43E-10	2.82E-08	-
Novel02421	2166.449	1258.613	0.7835	3.77E-24	2.73E-21	-
Novel02431	14.58496	1.305726	3.4816	0.000302	0.009904	-
Novel02438	174.1714	1657.986	-3.2509	1.84E-96	1.07E-92	-
Novel02463	64.20642	27.67681	1.214	0.000255	0.008773	-
Novel02615	3126.96	2662.694	0.23187	0.001529	0.034443	-
Novel02687	4665.506	5478.602	-0.23177	0.000268	0.00914	-
Novel02710	32.72583	91.42787	-1.4822	3.44E-07	3.32E-05	-
Novel02746	25.23454	56.84973	-1.1718	0.000758	0.019866	-
Novel02821	96.2986	168.8959	-0.81055	4.65E-05	0.002186	-
Novel02837	14.92956	46.58567	-1.6417	0.000179	0.006615	-
Novel02907	261.8939	358.4549	-0.45281	0.002007	0.041676	-

Novel02936	1371.911	1072.469	0.35525	0.000403	0.012293	-
Novel02956	337.7744	233.3028	0.53386	0.000127	0.005002	-
Novel02960	79.39956	128.1296	-0.6904	0.00158	0.035036	-
Novel02998	36.30354	70.40679	-0.9556	0.001635	0.035987	-
Novel03035	82.1983	138.4018	-0.75168	0.000522	0.015012	-
Novel03066	220.9326	321.6534	-0.5419	0.000127	0.005009	-
SOD	4276.435	5013.833	-0.22951	0.0004	0.012257	superoxide dismutase [Cu-Zn]
TPS	165.244	235.0777	-0.50854	0.001774	0.038255	alpha,alpha-trehalose-phosphate synthase [UDP-forming]
diptericin	168.5078	91.45615	0.88166	0.000635	0.01725	diptericin-D-like
nAChRbeta3	261.2553	164.5528	0.66691	3.10E-05	0.001549	neuronal acetylcholine receptor subunit alpha-4-like
yp3	1060.598	1415.287	-0.41622	6.41E-07	5.74E-05	vitellogenin-1-like
