# Supplementary material for: Short-term particulate matter contamination severely compromises insect antennal olfactory perception
Source: Nat Commun. 2023 Jul 11;14:4112. doi: 10.1038/s41467-023-39469-3 (PMC10336072; doi:10.1038/s41467-023-39469-3)
Supplement: Supplementary file 7 — Supplementary Data 4 [file 41467_2023_39469_MOESM7_ESM.pdf]

Supplementary Data 4. Details of differential expressed genes (DEGs) between antennae of uncontaminated and contaminated male houseflies collected in summer. Readcount is the average read count of each gen, log2FoldChange is calculated by formular log2(readcount_CMA/readcount_UMA), pval is the *p* value, padj is the fdr corrected *p* value using Benjamini/Hochberg method. All *p*-values are based on two-sided tests. CMA: contaminated male antennae, UMA: uncontaminated male antennae.

**Gene_id**	**readcount_CMA**	**readcount_UMA**	**log2FoldChange**	**pval**	**padj**	**Description**
AQP	6164.077	7918.665	-0.36137	1.02E-12	1.69E-10	aquaporin
CPI	0.348761	19.60651	-5.8129	3.43E-07	1.94E-05	sarcocystatin-A-like
CPR	34061.84	30704.93	0.14969	0.000854	0.015511	NADPH--cytochrome P450 reductase-like, transcript variant X1
CYP6A24	27426.97	36413.59	-0.40888	3.23E-11	4.33E-09	cytochrome P450 6A1-like
CYP6D3	23352.73	28823.53	-0.30366	8.13E-11	9.58E-09	cytochrome P450 6d3-like, transcript variant X1
CYP6D8	2221.964	1785.908	0.31518	1.32E-06	6.49E-05	probable cytochrome P450 6d5-like
CYP6G4	67334.75	78203.84	-0.21589	1.20E-06	6.01E-05	cytochrome P450 6g1-like
GST-6A	8925.877	9904.185	-0.15004	0.00286	0.040746	glutathione S-transferase 1-like
Gr2	7212.667	4277.255	0.75385	2.12E-46	2.86E-43	gustatory and odorant receptor 63a-like
LOC101887206	3882.256	4471.383	-0.20383	0.000798	0.014697	protein dopey-1 homolog
LOC101887230	344.4668	250.9031	0.45724	0.000754	0.014018	cleft lip and palate transmembrane protein 1-like protein, transcript variant X1
LOC101887237	179.5123	252.1949	-0.49046	0.002484	0.036599	uncharacterized LOC101887237, transcript variant X1
LOC101887273	2898.563	2252.462	0.36384	4.70E-09	4.00E-07	protein sickie, transcript variant X1
LOC101887291	3213.193	2753.079	0.22296	0.000161	0.004054	ATP-binding cassette sub-family G member 4, transcript variant X3
LOC101887315	4870.883	4343.577	0.1653	0.002489	0.036639	mannose-6-phosphate isomerase
LOC101887352	7191.865	8963.558	-0.31771	3.21E-08	2.28E-06	venom protease
LOC101887377	495.1053	631.582	-0.35123	0.000964	0.017216	PRKCA-binding protein, transcript variant X2
LOC101887379	12875.37	10893.71	0.24112	1.27E-06	6.29E-05	uncharacterized LOC101887379, transcript variant X1
LOC101887381	292.7973	118.5483	1.3044	2.70E-13	4.78E-11	Chitin binding Peritrophin-A, transcript variant X2
LOC101887389	883.3176	636.6068	0.47253	6.99E-07	3.67E-05	acyl-CoA desaturase
LOC101887394	1222.343	893.0752	0.4528	3.07E-08	2.21E-06	probable cytochrome P450 311a1, transcript variant X2
LOC101887397	120.427	466.9323	-1.9551	6.68E-32	5.10E-29	H/ACA ribonucleoprotein complex subunit 1

LOC101887443	1009.008	784.7384	0.36265	2.92E-05	0.000961	actin-87E
LOC101887449	344.2522	486.9019	-0.50017	2.70E-05	0.0009	ninjurin-1, transcript variant X2
LOC101887475	243.0643	141.9374	0.77608	9.70E-06	0.000365	farnesyl pyrophosphate synthase-like
LOC101887496	1379.887	1644.853	-0.25341	0.000318	0.007033	thioredoxin-like protein 1
LOC101887504	7.603639	25.61214	-1.7521	0.003425	0.046845	uncharacterized LOC101887504
LOC101887528	14.74386	40.26189	-1.4493	0.001435	0.023867	uncharacterized transmembrane protein DDB_G0289901-like
LOC101887539	2293.555	3258.866	-0.50678	2.52E-15	5.66E-13	PAX3- and PAX7-binding protein 1, transcript variant X3
LOC101887559	8728.261	7546.316	0.20992	3.91E-05	0.001248	uncharacterized LOC101887559, transcript variant X1
LOC101887561	152.8495	292.5076	-0.93636	1.69E-09	1.58E-07	stearoyl-CoA desaturase 5, transcript variant X2
LOC101887577	216.1234	148.8553	0.53794	0.00157	0.025656	D-3-phosphoglycerate dehydrogenase
LOC101887601	259.7684	183.3921	0.5023	0.00174	0.027833	peptidyl-prolyl cis-trans isomerase-like 1
LOC101887603	338.0989	237.6854	0.50839	0.001503	0.024855	uncharacterized LOC101887603
LOC101887604	852.5571	698.3323	0.28788	0.002003	0.031021	transmembrane protein 104 homolog, transcript variant X2
LOC101887632	3.831339	30.43774	-2.9899	3.86E-06	0.000164	uncharacterized LOC101887632, transcript variant X2
LOC101887665	1280.224	1041.028	0.29839	0.000167	0.004192	calcium-binding mitochondrial carrier protein SCaMC-2, transcript variant X1
LOC101887680	1165.44	967.0005	0.26929	0.00089	0.016057	transmembrane protein 185B
LOC101887688	921.5634	698.8865	0.39903	1.01E-05	0.000376	protein numb, transcript variant X3
LOC101887696	1479.464	1735.695	-0.23044	0.001471	0.02438	succinate dehydrogenase assembly factor 4, mitochondrial
LOC101887728	7928.811	6766.724	0.22865	2.69E-06	0.000119	gamma-glutamyltranspeptidase 1
LOC101887732	452.7126	357.2726	0.34157	0.002993	0.042247	transcription termination factor 3, mitochondrial
LOC101887745	6272.979	7227.101	-0.20427	4.58E-05	0.001429	secretory carrier-associated membrane protein 1
LOC101887747	266.4997	145.581	0.87231	4.15E-08	2.88E-06	synaptic vesicle glycoprotein 2B
LOC101887752	651.728	499.2592	0.38448	0.000211	0.005086	ATP-dependent RNA helicase vasa
LOC101887777	56.53916	108.3419	-0.93827	0.000263	0.006075	attacin-A-like
LOC101887788	985.3724	631.3035	0.64234	3.33E-12	5.17E-10	uncharacterized LOC101887788
LOC101887790	1364.483	1711.782	-0.32714	5.75E-06	0.000231	rhythmically expressed gene 2 protein
LOC101887791	390.3354	291.713	0.42016	0.001089	0.018843	uncharacterized LOC101887791, transcript variant X1

LOC101887803	9358.043	8013.47	0.22378	8.79E-06	0.000335	acyl-CoA Delta(11) desaturase, transcript variant X1
LOC101887813	260.8678	130.6439	0.99768	1.01E-08	8.40E-07	protein peste
LOC101887821	155.8192	103.2715	0.59343	0.003398	0.046581	N-sulphoglucosamine sulphohydrolase, transcript variant X2
LOC101887824	258.7432	610.0543	-1.2374	1.38E-25	6.75E-23	uncharacterized abhydrolase domain-containing protein DDB_G0269086
LOC101887833	14750.88	18152.11	-0.29934	4.34E-05	0.001367	ATP-dependent RNA helicase p62, transcript variant X2
LOC101887834	1139.984	1527.518	-0.42218	3.50E-06	0.00015	uncharacterized LOC101887834, transcript variant X1
LOC101887849	3085.3	3690.85	-0.25854	8.96E-06	0.00034	calpain-A, transcript variant X2
LOC101887866	844.0733	673.069	0.32661	0.000253	0.005923	mediator of RNA polymerase II transcription subunit 21
LOC101887872	458.3823	1098.563	-1.261	3.82E-12	5.83E-10	phormicin
LOC101887881	7539.101	8863.245	-0.23344	0.000257	0.005984	PI-PLC X domain-containing protein 1
LOC101887883	1503.579	922.112	0.70539	2.30E-19	8.22E-17	protein eiger, transcript variant X2
LOC101887889	192.4904	294.1058	-0.61155	4.84E-05	0.001499	putative uncharacterized protein DDB_G0267716
LOC101887902	3083.335	4526.212	-0.55381	1.62E-22	6.93E-20	kinesin-related protein 4, transcript variant X1
LOC101887904	622.6329	856.3477	-0.45981	6.93E-07	3.66E-05	cell division protein ZipA
LOC101887923	289.9566	469.5235	-0.69536	1.97E-08	1.48E-06	protein lin-37 homolog, transcript variant X1
LOC101887958	928.3354	725.6601	0.35535	7.50E-05	0.002165	1-acyl-sn-glycerol-3-phosphate acyltransferase delta, transcript variant X3
LOC101887959	3674.276	4202.999	-0.19396	0.000521	0.010394	ubiquitin carboxyl-terminal hydrolase 32
LOC101887961	9015.74	10227.52	-0.18194	0.000302	0.006753	cathepsin B
LOC101888007	1000.018	1266.839	-0.34121	1.94E-05	0.000665	tonsoku-like protein, transcript variant X1
LOC101888032	84375.19	104019.7	-0.30197	3.17E-12	4.97E-10	general odorant-binding protein 19a
LOC101888087	818.6553	555.6302	0.55913	1.08E-08	8.86E-07	uncharacterized LOC101888087
LOC101888148	685.9116	861.0241	-0.32803	0.000598	0.011575	hemocytin, transcript variant X2
LOC101888167	1078.212	1343.914	-0.3178	0.000845	0.015424	uncharacterized LOC101888167
LOC101888192	276.7859	392.2378	-0.50296	0.000171	0.004282	apyrase
LOC101888215	1258.065	1499.531	-0.2533	0.000863	0.015634	condensin complex subunit 3
LOC101888223	378.1155	250.203	0.59573	6.36E-06	0.000252	tumor necrosis factor receptor superfamily member wengen,

						transcript variant X1
LOC101888225	3.504777	38.16076	-3.4447	1.81E-08	1.39E-06	defensin-1-like
LOC101888226	20890.22	23484.54	-0.16888	0.000337	0.007353	peroxiredoxin-6
LOC101888255	14441.05	12394	0.22053	2.44E-06	0.00011	probable aconitate hydratase, mitochondrial
LOC101888291	61792.81	73761.94	-0.25544	1.07E-08	8.81E-07	sensory neuron membrane protein 1
LOC101888299	2804.361	3193.96	-0.18767	0.001782	0.028435	proteasome subunit alpha type-3
LOC101888312	1223.545	1471.419	-0.26614	0.001733	0.027746	F-actin-capping protein subunit beta, transcript variant X2
LOC101888320	908.0742	702.6386	0.37003	6.54E-05	0.001912	protein takeout
LOC101888341	333318.6	377567.1	-0.17983	3.28E-05	0.001063	general odorant-binding protein 83a
LOC101888352	4087.428	5201.476	-0.34773	2.47E-10	2.64E-08	ATP-binding cassette sub-family A member 3, transcript variant X5
LOC101888354	613.6878	494.386	0.31187	0.002948	0.041684	Gap junction alpha-1 protein
LOC101888357	1603.563	1339.37	0.25973	0.00034	0.007411	scavenger receptor class B member 1, transcript variant X3
LOC101888363	2239.488	1828.891	0.2922	5.06E-06	0.000208	succinyl-CoA:3-ketoacid coenzyme A transferase 1, mitochondrial, transcript variant X3
LOC101888383	187.0226	122.0001	0.61633	0.001128	0.019432	PDZ domain-containing protein 8
LOC101888396	1255.666	1591.838	-0.34224	5.61E-06	0.000228	U4/U6.U5 tri-snRNP-associated protein 1
LOC101888407	163.5876	285.2082	-0.80195	6.95E-07	3.66E-05	ejaculatory bulb-specific protein 3
LOC101888424	2954.73	2450.874	0.26973	8.75E-06	0.000334	sterile alpha and TIR motif-containing protein 1, transcript variant X1
LOC101888428	510.9183	742.33	-0.53897	3.15E-08	2.25E-06	aquaporin AQPcic
LOC101888432	310.5855	407.0511	-0.39022	0.002286	0.034339	odorant receptor 74a-like
LOC101888462	53731.85	66999.69	-0.31838	1.51E-10	1.69E-08	general odorant-binding protein 69a
LOC101888470	621.7704	918.5854	-0.56303	1.28E-09	1.23E-07	protein CREBRF homolog, transcript variant X2
LOC101888478	314.9627	198.2193	0.66808	0.00043	0.008875	uncharacterized LOC101888478
LOC101888488	1339.926	1552.635	-0.21257	0.002443	0.036113	putative tricarboxylate transport protein, mitochondrial, transcript variant X1
LOC101888493	183.6295	93.14137	0.9793	8.63E-07	4.42E-05	transforming growth factor beta-1-induced transcript 1 protein

LOC101888494	26023.18	23210.41	0.16503	0.000287	0.006496	protein takeout
LOC101888495	801.7687	975.5474	-0.28303	0.002573	0.037557	kinesin-related protein 10, transcript variant X4
LOC101888514	580517.9	673475.8	-0.21429	9.38E-07	4.77E-05	pheromone-binding protein-related protein 6, transcript variant X2
LOC101888515	39.63384	78.20842	-0.98059	0.001167	0.019991	uncharacterized LOC101888515
LOC101888530	4.785169	37.25509	-2.9608	3.31E-07	1.90E-05	uncharacterized LOC101888530, transcript variant X1
LOC101888535	2686.835	3764.269	-0.48646	1.15E-16	3.31E-14	fatty-acid amide hydrolase 2-A
LOC101888555	1434.6	1696.201	-0.24166	0.001316	0.022161	uncharacterized LOC101888555
LOC101888567	895.2234	591.4004	0.59811	1.12E-10	1.30E-08	uncharacterized LOC101888567
LOC101888576	24.60027	63.20709	-1.3614	0.000131	0.003415	uncharacterized family 31 glucosidase KIAA1161
LOC101888593	1845.961	1309.617	0.49523	3.45E-12	5.31E-10	heat shock protein 27
LOC101888602	522.0696	352.6891	0.56585	0.0006	0.011591	transcriptional regulator ATRX homolog, transcript variant X1
LOC101888615	116.152	288.8076	-1.3141	8.41E-15	1.78E-12	uncharacterized LOC101888615
LOC101888627	1160.681	935.277	0.31151	0.000422	0.008746	sodium/potassium/calcium exchanger 3, transcript variant X2
LOC101888630	1092.341	803.8312	0.44246	2.44E-07	1.45E-05	bumetanide-sensitive sodium-(potassium)-chloride cotransporter, transcript variant X1
LOC101888639	372.0702	226.4084	0.71665	4.39E-05	0.00138	transient receptor potential cation channel subfamily V member 5
LOC101888654	787.3666	943.591	-0.26113	0.003718	0.049734	calcium-binding protein E63-1
LOC101888656	232.2766	157.5645	0.5599	0.001003	0.017728	nucleolar complex protein 3 homolog
LOC101888659	1324.471	1067.033	0.31181	6.58E-05	0.00192	putative tricarboxylate transport protein, mitochondrial, transcript variant X1
LOC101888662	5241.427	4401.478	0.25197	3.93E-06	0.000166	uncharacterized LOC101888662
LOC101888676	1442.307	1163.984	0.30931	5.31E-05	0.001611	alpha-tocopherol transfer protein-like
LOC101888705	11414.46	10174.88	0.16585	0.000417	0.008669	restin homolog, transcript variant X5
LOC101888717	0.931631	25.14479	-4.7544	8.45E-08	5.51E-06	uncharacterized LOC101888717
LOC101888734	69.37877	136.8542	-0.98007	7.22E-05	0.002086	uncharacterized LOC101888734
LOC101888738	22957.96	26109.89	-0.1856	0.000574	0.01119	bromodomain-containing protein DDB_G0280777
LOC101888763	3618.464	2881.276	0.32867	1.42E-08	1.11E-06	protein bangles and beads
LOC101888778	81.83996	140.9765	-0.78458	0.00024	0.005679	protein psiN, transcript variant X2

LOC101888787	1815.703	2598.589	-0.5172	1.61E-09	1.51E-07	serine/arginine repetitive matrix protein 1
LOC101888835	1396.132	1757.973	-0.33248	2.17E-06	0.0001	8-oxo-dGDP phosphatase NUDT18, transcript variant X3
LOC101888850	10.25802	139.1815	-3.7621	3.44E-30	2.32E-27	uncharacterized LOC101888850
LOC101888852	18.34109	48.67985	-1.4082	0.000479	0.009664	protein PFC0760c, transcript variant X1
LOC101888854	749.7075	611.9086	0.29301	0.002543	0.037272	uncharacterized LOC101888854, transcript variant X1
LOC101888862	746.8081	956.2924	-0.35671	5.55E-05	0.001676	RNA-directed DNA polymerase from mobile element jockey, transcript variant X1
LOC101888870	206.9308	123.4493	0.74523	3.36E-05	0.001085	proline-rich extensin-like protein EPR1
LOC101888873	644.8353	831.0959	-0.36608	8.45E-05	0.002387	GA-binding protein subunit beta-1, transcript variant X1
LOC101888879	2215.828	2694.487	-0.28217	1.03E-05	0.00038	protein similar
LOC101888894	1382.829	1615.287	-0.22417	0.002116	0.032367	tyrosine-protein phosphatase vhp-1
LOC101888925	426.4173	332.8196	0.35753	0.003533	0.047728	uncharacterized LOC101888925
LOC101888926	256.6751	433.5001	-0.75609	3.61E-09	3.15E-07	sodium/potassium/calcium exchanger 4
LOC101888931	258.2081	373.6643	-0.53321	7.76E-05	0.002226	O-phosphoseryl-tRNA(Sec) selenium transferase
LOC101888941	652.8408	500.538	0.38325	0.000199	0.004866	serine/threonine-protein kinase S6KL
LOC101888955	3281.718	4224.418	-0.3643	1.63E-10	1.80E-08	glycerophosphocholine phosphodiesterase GPCPD1, transcript variant X2
LOC101888990	3371.424	2985.087	0.17559	0.002067	0.031731	matrix metalloproteinase-14, transcript variant X2
LOC101888994	37.72254	73.15507	-0.95553	0.002874	0.040864	glutamate receptor ionotropic, delta-1
LOC101889001	718.3755	1125.938	-0.64832	6.86E-14	1.31E-11	uncharacterized LOC101889001, transcript variant X1
LOC101889021	2173.184	1835.748	0.24344	0.000226	0.005376	dnaJ homolog shv
LOC101889038	1343.657	1146.659	0.22873	0.002016	0.031059	disheveled-associated activator of morphogenesis 1
LOC101889041	1132.098	1387.572	-0.29356	0.00012	0.003203	uncharacterized LOC101889041
LOC101889056	406.9409	304.9687	0.41616	0.001281	0.021672	ruvB-like helicase 2
LOC101889127	207.4709	292.0455	-0.49328	0.000853	0.015505	paramyosin
LOC101889163	2149.834	2558.843	-0.25127	4.65E-05	0.001444	uncharacterized LOC101889163
LOC101889170	959.2306	1143.924	-0.25404	0.001911	0.029996	uncharacterized LOC101889170
LOC101889198	3509.408	2352.714	0.5769	0.000813	0.014933	vasotab

LOC101889208	25.57379	149.3945	-2.5464	5.88E-17	1.75E-14	carboxypeptidase Y
LOC101889224	3052.733	2437.207	0.32487	9.05E-08	5.86E-06	lysophospholipid acyltransferase 1, transcript variant X5
LOC101889234	3248.192	3711.484	-0.19236	0.000917	0.016474	26S protease regulatory subunit 8
LOC101889248	44.59409	175.0259	-1.9726	3.68E-17	1.15E-14	uncharacterized LOC101889248
LOC101889274	786.684	977.9327	-0.31395	0.000248	0.005828	tetratricopeptide repeat protein 21B
LOC101889287	1838.855	2176.633	-0.24329	0.001505	0.024855	cationic amino acid transporter 2, transcript variant X1
LOC101889303	629.4648	810.7791	-0.36518	0.000153	0.003892	putative sulfiredoxin, transcript variant X3
LOC101889304	17113.73	15507.21	0.14222	0.003415	0.046743	sperm surface protein Sp17, transcript variant X1
LOC101889324	2994.295	3580.941	-0.25812	9.20E-06	0.000348	NF-kappa-B inhibitor cactus, transcript variant X4
LOC101889356	11004.08	12455.91	-0.17879	0.001506	0.024855	moesin/ezrin/radixin homolog 1
LOC101889358	2094.119	2453.625	-0.22857	0.000407	0.008533	uncharacterized LOC101889358
LOC101889360	176.7287	86.2853	1.0343	4.38E-07	2.42E-05	dynein heavy chain 5, axonemal
LOC101889365	15176.29	20003.17	-0.39841	5.73E-13	9.76E-11	cytochrome P450 6A1-like
LOC101889370	2234.389	2542.208	-0.1862	0.003646	0.048914	uncharacterized protein At4g17910, transcript variant X1
LOC101889374	10055.59	7247.555	0.47243	3.76E-15	8.14E-13	dynein beta chain, ciliary
LOC101889377	394.1752	275.5659	0.51644	5.17E-05	0.001577	titin
LOC101889400	1078.054	1428.255	-0.40582	0.000176	0.0044	mucin-5AC, transcript variant X3
LOC101889402	1877.623	2162.033	-0.20348	0.001847	0.029253	CD109 antigen, transcript variant X3
LOC101889403	1004.99	808.5689	0.31374	0.001049	0.018377	ets DNA-binding protein pokkuri, transcript variant X2
LOC101889413	2008.762	2404.431	-0.25939	4.48E-05	0.001405	solute carrier family 41 member 2, transcript variant X4
LOC101889429	826.5224	666.7321	0.30995	0.000899	0.016204	sn1-specific diacylglycerol lipase alpha, transcript variant X1
LOC101889442	790.9657	946.1954	-0.25852	0.003238	0.045098	uncharacterized LOC101889442
LOC101889452	1404.464	1679.428	-0.25795	0.000265	0.006112	glycine-rich cell wall structural protein 1, transcript variant X4
LOC101889455	56.3767	115.8251	-1.0388	7.55E-05	0.002176	uncharacterized LOC101889455
LOC101889457	2245.793	1869.48	0.26459	2.77E-05	0.000917	coatomer subunit gamma
LOC101889461	1884.065	2613.058	-0.47189	1.88E-08	1.42E-06	cationic amino acid transporter 3, transcript variant X1
LOC101889463	377.3492	621.9428	-0.72088	5.68E-11	7.18E-09	uncharacterized LOC101889463
LOC101889504	2260.248	1987.87	0.18526	0.003511	0.047673	dnaJ homolog subfamily C member 3

LOC101889527	1484.627	1838.09	-0.30811	1.17E-05	0.000429	lipase 3-like
LOC101889565	700.8816	378.0258	0.89069	8.18E-17	2.39E-14	alpha-tocopherol transfer protein-like
LOC101889570	1.968077	24.81894	-3.6566	2.16E-06	9.99E-05	uncharacterized LOC101889570
LOC101889596	221.0364	136.2661	0.69786	6.50E-05	0.001908	B-cell lymphoma/leukemia 11B
LOC101889618	0.97603	33.95634	-5.1206	1.03E-10	1.21E-08	uncharacterized LOC101889618
LOC101889623	5836.517	5144.885	0.18197	0.001666	0.026916	eukaryotic translation initiation factor 3 subunit C
LOC101889629	463.8396	599.5143	-0.37017	0.000346	0.0075	protein THEM6, transcript variant X2
LOC101889661	2001.841	1698.222	0.2373	0.000373	0.008005	probable enoyl-CoA hydratase, mitochondrial
LOC101889682	13.58796	54.10941	-1.9936	2.73E-06	0.000121	serendipity locus protein alpha, transcript variant X2
LOC101889696	121.8072	75.95977	0.68129	0.003331	0.046099	uncharacterized LOC101889696
LOC101889715	3054.818	2630.987	0.21548	0.000712	0.013325	Malate/L-lactate dehydrogenase, transcript variant X1
LOC101889718	4643.516	5267.456	-0.18189	0.000764	0.014163	suppressor of hairless protein
LOC101889732	772.6788	570.2407	0.4383	4.29E-06	0.00018	prostatic acid phosphatase
LOC101889761	1638.755	1952.436	-0.25267	0.000255	0.005941	IST1 homolog, transcript variant X2
LOC101889773	7365.766	8799.246	-0.25654	9.47E-05	0.002624	UDP-glucuronosyltransferase 2B9-like
LOC101889778	609.4313	406.7936	0.58317	1.18E-07	7.36E-06	uncharacterized LOC101889778
LOC101889793	472.7012	367.7495	0.36221	0.003478	0.047351	chromobox protein homolog 1
LOC101889812	1.574917	52.25606	-5.0523	1.60E-15	3.74E-13	uncharacterized LOC101889812
LOC101889815	109.3999	172.8845	-0.6602	0.000858	0.015566	fibrillin-1, transcript variant X1
LOC101889825	999.3791	1189.842	-0.25167	0.001905	0.029976	ubiquitin fusion degradation protein 1 homolog
LOC101889828	1361.162	1074.518	0.34115	1.72E-05	0.000597	protein Malvolio, transcript variant X4
LOC101889916	1310.252	1114.22	0.23381	0.002169	0.032946	AN1-type zinc finger protein 5, transcript variant X2
LOC101889928	955.8687	1279.264	-0.42043	9.94E-08	6.36E-06	odorant receptor 67d-like
LOC101889936	1180.773	1483.598	-0.32937	8.55E-05	0.002406	autophagy-related protein 2 homolog B
LOC101889943	2239.436	2702.929	-0.27139	0.000128	0.00337	tight junction protein ZO-1, transcript variant X4
LOC101889953	764.9614	954.5152	-0.31938	0.000282	0.006449	uncharacterized LOC101889953, transcript variant X4
LOC101889957	112.3117	270.3802	-1.2675	2.62E-13	4.69E-11	attacin-A-like
LOC101889976	1158.025	1418.094	-0.29229	0.000147	0.003774	tetracycline resistance protein, class A

LOC101889993	306.602	218.2281	0.49053	0.001064	0.018577	DPH3 homolog, transcript variant X2
LOC101890000	3335.383	2801.049	0.25189	0.003589	0.048256	fatty acyl-CoA reductase wat
LOC101890005	135.6119	502.843	-1.8906	7.32E-41	8.56E-38	C-type lectin 37Db, transcript variant X2
LOC101890015	598.3125	734.0321	-0.29494	0.002043	0.031412	spermatogenesis-associated protein 5
LOC101890023	952.6745	733.2964	0.37759	2.86E-05	0.000945	uncharacterized LOC101890023
LOC101890051	4433.231	5006.244	-0.17537	0.00153	0.025189	probable cation-transporting ATPase 13A3, transcript variant X1
LOC101890092	434.4482	572.6356	-0.39843	0.000461	0.009411	uncharacterized LOC101890092
LOC101890094	0.310544	11.36774	-5.194	0.000294	0.006627	vitelline membrane protein Vm26Ab-like
LOC101890101	13.68156	40.41617	-1.5627	0.000849	0.01547	uncharacterized LOC101890101
LOC101890109	512.88	714.4867	-0.47829	1.83E-06	8.63E-05	hypothetical protein
LOC101890125	1278.839	2020.595	-0.65995	1.69E-14	3.50E-12	elongation of very long chain fatty acids protein 7
LOC101890131	2539.453	2998.43	-0.23969	9.86E-05	0.002721	interferon regulatory factor 2-binding protein-like, transcript variant X1
LOC101890149	2704.585	2243.409	0.26971	9.34E-06	0.000352	uncharacterized LOC101890149
LOC101890180	14818.29	16828.15	-0.1835	0.00014	0.00363	period circadian protein, transcript variant X2
LOC101890187	4211.637	4914.451	-0.22265	6.40E-05	0.001882	ATP-dependent RNA helicase bel, transcript variant X2
LOC101890191	1901.832	2234.212	-0.23238	0.000289	0.006525	26S proteasome non-ATPase regulatory subunit 8
LOC101890200	699.2401	1093.474	-0.64506	8.98E-14	1.68E-11	protein MCM10 homolog
LOC101890254	2.196004	15.04729	-2.7766	0.003216	0.044825	phosphoenolpyruvate carboxykinase [GTP]
LOC101890259	2.817091	27.87638	-3.3068	4.28E-05	0.001354	cuticle protein 16.5
LOC101890271	7963.819	9697.2	-0.28411	1.13E-08	9.26E-07	UDP-glucuronosyltransferase 2B15, transcript variant X1
LOC101890297	1384.715	960.8008	0.52728	1.54E-11	2.20E-09	short/branched chain specific acyl-CoA dehydrogenase, mitochondrial
LOC101890371	962.743	736.0354	0.38738	1.73E-05	0.000599	fringe glycosyltransferase
LOC101890375	1477.694	2058.908	-0.47853	5.51E-11	7.05E-09	rab5 GDP/GTP exchange factor
LOC101890383	2268.321	2606.063	-0.20025	0.002429	0.035966	sodium/bile acid cotransporter, transcript variant X2
LOC101890405	601.7695	479.6277	0.3273	0.002338	0.034969	protein takeout
LOC101890413	702.1198	968.3464	-0.46381	1.94E-07	1.17E-05	KH domain-containing, RNA-binding, signal transduction-

						associated protein 2, transcript variant X1
LOC101890431	3641.337	4714.886	-0.37275	7.75E-11	9.38E-09	SAFB-like transcription modulator, transcript variant X1
LOC101890434	3561.353	2667.013	0.4172	6.97E-13	1.18E-10	beta-galactosidase, transcript variant X2
LOC101890440	1140.279	1402.457	-0.29857	9.27E-05	0.002578	lipase 3-like
LOC101890486	2757.201	2326.074	0.24531	0.002215	0.033532	uncharacterized LOC101890486, transcript variant X1
LOC101890498	1383.113	1609.06	-0.2183	0.001791	0.028542	regucalcin, transcript variant X3
LOC101890499	215.0776	141.3445	0.60564	0.000536	0.010651	uncharacterized LOC101890499
LOC101890500	626.1871	476.5504	0.39396	0.000126	0.003323	GRIP and coiled-coil domain-containing protein 1
LOC101890527	849.8239	696.3348	0.28738	0.001701	0.027463	neutral and basic amino acid transport protein rBAT
LOC101890532	10004.83	11838.11	-0.24274	0.003031	0.042687	V-type proton ATPase subunit G
LOC101890540	4208.638	4936.466	-0.23013	2.71E-05	0.000902	molybdenum cofactor sulfurase 3
LOC101890544	33.96511	66.98482	-0.97978	0.002195	0.033283	fibroin heavy chain, transcript variant X2
LOC101890547	5539.331	4907.402	0.17475	0.000781	0.01443	uncharacterized LOC101890547, transcript variant X3
LOC101890549	1422.8	1052.287	0.4352	9.04E-07	4.61E-05	transcription factor mef2A, transcript variant X9
LOC101890572	3049.335	2260.006	0.43217	4.94E-06	0.000204	alpha-mannosidase 2
LOC101890579	106.2463	189.6663	-0.83605	1.53E-05	0.000544	uncharacterized LOC101890579
LOC101890619	423.4229	288.8939	0.55156	2.37E-05	0.000801	laminin subunit gamma-1, transcript variant X1
LOC101890621	531.244	686.063	-0.36897	0.000431	0.008888	uncharacterized LOC101890621
LOC101890629	1790.733	2206.315	-0.30109	9.42E-06	0.000355	U1 small nuclear ribonucleoprotein 70 kDa, transcript variant X1
LOC101890635	0.665486	11.41471	-4.1003	0.001141	0.019612	glycine-rich cell wall structural protein 1.8-like, transcript variant X2
LOC101890687	2220.046	2677.366	-0.27022	2.26E-05	0.000769	proteasome subunit beta type-4
LOC101890714	3799.949	2829.183	0.42559	2.09E-11	2.93E-09	probable cytochrome P450 28a5
LOC101890715	1012.35	1441.839	-0.5102	1.96E-08	1.47E-06	probable cytochrome P450 6a14
LOC101890728	30824.01	23642.29	0.38268	5.26E-17	1.59E-14	probable cytochrome P450 313a4
LOC101890734	670.5071	2074.284	-1.6293	2.01E-98	8.83E-95	uncharacterized LOC101890734, transcript variant X2
LOC101890768	1486.929	1731.46	-0.21965	0.002567	0.037544	dorsal-related immunity factor Dif, transcript variant X2
LOC101890770	94.15436	199.2287	-1.0813	2.79E-08	2.06E-06	uncharacterized LOC101890770, transcript variant X3

LOC101890832	16.03296	45.57372	-1.5072	0.000426	0.008804	neprilysin-1
LOC101890839	4390.855	3783.502	0.21478	0.000101	0.00277	calcineurin B homologous protein 1
LOC101890854	9211.267	7583.295	0.28057	1.16E-08	9.38E-07	glucose dehydrogenase [FAD, quinone]
LOC101890875	9.113336	42.61353	-2.2253	0.001254	0.021254	juvenile hormone acid O-methyltransferase-like
LOC101890884	6.442702	55.76481	-3.1136	6.56E-08	4.34E-06	probable salivary secreted peptide
LOC101890896	155.3631	471.8756	-1.6028	1.19E-30	8.35E-28	peptidoglycan-recognition protein SA-like
LOC101890906	2.348875	19.07	-3.0213	0.000281	0.00643	trypsin-1
LOC101890923	161.9958	93.8891	0.78693	0.000115	0.003084	serine-enriched protein, transcript variant X3
LOC101890927	351.4865	238.8997	0.55706	4.33E-05	0.001367	uncharacterized LOC101890927
LOC101890938	3573.68	3146.906	0.18348	0.00133	0.022327	transmembrane emp24 domain-containing protein eca
LOC101890951	4022.981	4642.482	-0.20663	0.000246	0.005802	translocation protein SEC63 homolog
LOC101890987	574.6627	440.3373	0.38411	0.000544	0.010748	serine/arginine repetitive matrix protein 2, transcript variant X3
LOC101890999	685.5249	1027.245	-0.5835	4.01E-06	0.000168	DNA ligase 1
LOC101891025	2447.361	3272.351	-0.4191	2.34E-12	3.70E-10	general odorant-binding protein 56a-like
LOC101891027	256.7774	145.73	0.81722	6.77E-07	3.59E-05	glucose dehydrogenase [FAD, quinone]-like
LOC101891055	256.9511	350.4999	-0.44792	0.001055	0.018456	tRNA-splicing ligase RtcB homolog
LOC101891077	46.2983	259.2908	-2.4855	2.84E-33	2.38E-30	vitellogenin-1
LOC101891085	748.5618	979.281	-0.3876	0.000114	0.003082	zinc finger protein 208
LOC101891099	1721.321	2239.825	-0.37987	1.74E-08	1.35E-06	arylsulfatase B
LOC101891137	2487.4	3142.876	-0.33745	3.46E-08	2.41E-06	zinc finger CCCH domain-containing protein 18
LOC101891143	69.98661	120.1105	-0.77921	0.001037	0.018193	putative helicase mov-10-B.1
LOC101891147	1396.301	1182.336	0.23997	0.001536	0.025241	lysosomal Pro-X carboxypeptidase
LOC101891160	1305.531	1075.701	0.27936	0.000254	0.005923	uncharacterized LOC101891160, transcript variant X4
LOC101891167	1.695751	16.59453	-3.2907	0.000916	0.016463	cobra venom factor-like
LOC101891171	1334.284	1126.076	0.24476	0.001533	0.02521	ubiquitin-like modifier-activating enzyme ATG7
LOC101891181	309.1449	221.0976	0.4836	0.000833	0.015274	28S ribosomal protein S18b, mitochondrial
LOC101891255	766.3162	936.3641	-0.28913	0.001023	0.01804	serine/threonine-protein kinase pelle
LOC101891266	178.6319	99.4551	0.84487	2.72E-05	0.000903	nucleolar protein 14 homolog

LOC101891280	301.3557	415.0901	-0.46196	0.000311	0.006896	uncharacterized LOC101891280
LOC101891293	339334.6	300205.8	0.17676	4.47E-05	0.001403	general odorant-binding protein 56d
LOC101891303	1379.185	1131.544	0.28552	0.000179	0.004446	cap-specific mRNA (nucleoside-2'-O-)-methyltransferase 2, transcript variant X2
LOC101891334	468.3785	343.8534	0.44588	0.000121	0.003218	insulin receptor
LOC101891339	1157.397	1386.659	-0.26073	0.000971	0.017316	uncharacterized LOC101891339
LOC101891357	167.4597	236.0142	-0.49506	0.003266	0.045344	venom allergen 5
LOC101891401	88.82591	43.69388	1.0235	0.000331	0.007243	flocculation protein FLO11
LOC101891403	4.059265	63.78361	-3.9739	3.12E-15	6.83E-13	uncharacterized LOC101891403
LOC101891404	114.391	65.63423	0.80145	0.001196	0.020411	protein son of sevenless, transcript variant X3
LOC101891414	391.7813	614.4113	-0.64916	3.77E-09	3.28E-07	cuticle protein 38
LOC101891430	3610.149	6804.884	-0.91451	2.02E-27	1.08E-24	heat shock protein 70
LOC101891440	1017.824	1218.139	-0.25919	0.001552	0.025405	solute carrier family 26 member 6
LOC101891448	244.0112	157.8998	0.62794	0.000146	0.003752	alpha-(1,6)-fucosyltransferase, transcript variant X2
LOC101891456	304.006	406.5681	-0.4194	0.001443	0.02396	nuclear pore complex protein Nup160 homolog
LOC101891484	375.7167	638.5023	-0.76505	4.90E-12	7.28E-10	lipopolysaccharide-induced tumor necrosis factor-alpha factor homolog, transcript variant X2
LOC101891486	987.6	806.163	0.29286	0.000552	0.010868	uncharacterized LOC101891486
LOC101891504	930.9725	1304.759	-0.48697	2.07E-09	1.90E-07	uncharacterized LOC101891504
LOC101891510	362.2914	260.9111	0.47359	0.000541	0.010722	serine proteinase stubble
LOC101891528	2449.206	2101.068	0.22119	0.000467	0.009479	Niemann-Pick C1 protein, transcript variant X6
LOC101891530	1907.502	2262.678	-0.24635	0.000178	0.004423	WD repeat-containing protein 19
LOC101891576	12442.66	13923.15	-0.16219	0.000407	0.008533	NADP-dependent malic enzyme, transcript variant X2
LOC101891594	295.6336	203.7709	0.53686	0.000456	0.00934	low-density lipoprotein receptor-related protein 4
LOC101891600	2101.194	1785.976	0.2345	0.001394	0.023248	uncharacterized LOC101891600
LOC101891626	306.2111	209.7513	0.54585	0.000276	0.006343	uncharacterized LOC101891626, transcript variant X2
LOC101891628	7030.127	8235.09	-0.22823	0.000386	0.008212	protein purity of essence, transcript variant X2
LOC101891646	3463.415	4009.175	-0.21111	0.000284	0.006474	transcription factor Sp9

LOC101891663	104.1872	157.9254	-0.60007	0.002682	0.03886	enhancer of split m3 protein
LOC101891665	3538.764	3062.059	0.20874	0.000253	0.005923	transcription factor HNF-4 homolog
LOC101891667	8117.255	7229.73	0.16705	0.000544	0.010748	titin
LOC101891673	961.7341	1146.281	-0.25325	0.001838	0.02913	zinc finger protein 2
LOC101891674	6.702666	26.11079	-1.9618	0.001238	0.021049	abscisic acid and environmental stress-inducible protein
LOC101891684	2224.423	2612.142	-0.2318	0.000334	0.007292	proton-coupled amino acid transporter 2
LOC101891700	20738.9	22988.35	-0.14856	0.00133	0.022327	60S ribosomal protein L23a
LOC101891711	507.3107	335.6769	0.5958	4.42E-07	2.43E-05	WD repeat and HMG-box DNA-binding protein 1
LOC101891733	1214.532	915.5302	0.40772	2.78E-07	1.63E-05	facilitated trehalose transporter Tret1, transcript variant X1
LOC101891759	6269.046	8658.766	-0.46592	2.83E-20	1.10E-17	probable cytochrome P450 4d14, transcript variant X1
LOC101891770	640.7715	816.5382	-0.34971	0.000207	0.005025	dnaJ homolog subfamily C member 8, transcript variant X1
LOC101891783	260.7305	349.5625	-0.42299	0.002112	0.032346	26S proteasome non-ATPase regulatory subunit 4
LOC101891785	424.5008	165.2051	1.3615	1.02E-09	9.99E-08	uncharacterized LOC101891785
LOC101891787	5.165966	28.24123	-2.4507	0.000125	0.003307	uncharacterized LOC101891787
LOC101891808	5444.263	1993.008	1.4498	2.80E-54	4.92E-51	uncharacterized LOC101891808
LOC101891811	178.2853	117.9271	0.59629	0.002128	0.032493	dynein assembly factor 1, axonemal homolog
LOC101891825	2533.979	2192.475	0.20884	0.001148	0.01971	glycine-rich selenoprotein
LOC101891842	2514.496	2966.07	-0.23828	0.000143	0.003688	cyclin-dependent kinase-like 1
LOC101891870	8050.884	10164.14	-0.33627	8.12E-07	4.19E-05	serine/arginine repetitive matrix protein 2, transcript variant X5
LOC101891874	198.7066	313.5672	-0.65814	5.41E-06	0.00022	general odorant-binding protein 28a
LOC101891885	735.3157	593.3519	0.30948	0.001821	0.028937	RNA polymerase II-associated factor 1 homolog
LOC101891909	3271.6	4015.672	-0.29565	4.27E-07	2.36E-05	splicing factor 1
LOC101891931	28.17163	101.0957	-1.8434	8.78E-10	8.65E-08	probable cytochrome P450 4d14, transcript variant X3
LOC101891946	1436.834	1179.735	0.28443	0.00032	0.007053	clathrin interactor 1, transcript variant X2
LOC101891949	773.6216	1175.763	-0.6039	1.44E-11	2.09E-09	uncharacterized LOC101891949
LOC101891951	1720.69	2223.686	-0.36997	2.90E-08	2.11E-06	uncharacterized LOC101891951, transcript variant X2
LOC101891954	1507.892	1781.603	-0.24064	0.000571	0.011164	splicing factor 3B subunit 4
LOC101891960	962.491	1421.43	-0.5625	7.46E-13	1.25E-10	uncharacterized LOC101891960

LOC101891991	7109.679	6229.579	0.19065	0.000177	0.004415	uncharacterized LOC101891991
LOC101892029	28.73117	63.27755	-1.1391	0.001135	0.019527	adhesion G-protein coupled receptor G4
LOC101892030	2184.895	2491.861	-0.18966	0.001992	0.030872	T-cell immunomodulatory protein
LOC101892031	20121.73	17707.8	0.18437	4.78E-05	0.001483	protein D2-like
LOC101892037	400.5419	312.8412	0.35652	0.003524	0.047718	TBP-related factor
LOC101892039	145.2738	214.3876	-0.56145	0.001513	0.024922	pseudouridylate synthase 7 homolog
LOC101892046	681.2322	973.042	-0.51436	1.12E-07	7.08E-06	SRSF protein kinase 1, transcript variant X4
LOC101892069	1716.003	2165.802	-0.33585	5.04E-07	2.75E-05	protein DDI1 homolog 2, transcript variant X1
LOC101892072	33908.15	39532.26	-0.2214	1.33E-06	6.51E-05	cytochrome P450 6A1-like
LOC101892087	873.5221	721.4919	0.27586	0.001928	0.030104	fasciclin-2, transcript variant X1
LOC101892091	9422.73	12114.74	-0.36255	3.31E-08	2.33E-06	gamma-glutamyltranspeptidase 1
LOC101892111	3340.307	2784.78	0.26242	0.000833	0.015274	protein preli-like, transcript variant X1
LOC101892133	4974.007	6031.821	-0.27819	1.53E-07	9.40E-06	golgin subfamily A member 6-like protein 22
LOC101892139	2021.474	2419.908	-0.25954	6.36E-05	0.001877	proteasome subunit alpha type-5, transcript variant X2
LOC101892141	1.324791	15.68622	-3.5657	0.000391	0.008298	alcohol dehydrogenase
LOC101892143	4633.334	4027.638	0.20212	0.000742	0.013825	C3 and PZP-like alpha-2-macroglobulin domain-containing protein 8
LOC101892144	1368.407	1033.681	0.40471	1.23E-05	0.000446	programmed cell death protein 4
LOC101892152	4715.307	4026.648	0.22777	3.61E-05	0.001159	protein DEK
LOC101892170	199.984	278.0166	-0.47529	0.002436	0.036036	general odorant-binding protein 56h-like
LOC101892180	22501.19	26796.36	-0.25204	3.09E-08	2.21E-06	aldehyde dehydrogenase, dimeric NADP-preferring, transcript variant X3
LOC101892195	132.2515	80.32498	0.71936	0.002343	0.034989	uncharacterized LOC101892195
LOC101892202	1772.086	1485.792	0.25422	0.00021	0.005072	probable Ufm1-specific protease 2
LOC101892211	4011.464	4835.725	-0.2696	1.06E-06	5.32E-05	uncharacterized LOC101892211
LOC101892221	2441.79	1760.838	0.47168	4.95E-11	6.39E-09	dynein light chain Tctex-type
LOC101892230	1838.71	1327.157	0.47035	2.94E-11	4.03E-09	glutamine--fructose-6-phosphate aminotransferase [isomerizing] 2
LOC101892245	22.18114	5.156987	2.1047	0.001953	0.030353	carcinine transporter

LOC101892284	3761.643	4734.523	-0.33186	1.76E-09	1.64E-07	protein crumbs, transcript variant X2
LOC101892311	4025.507	4581.87	-0.18677	0.000663	0.012571	trehalase
LOC101892320	932.9626	717.5209	0.3788	2.36E-05	0.000797	isopentenyl-diphosphate Delta-isomerase 1
LOC101892332	425.7567	305.3945	0.47936	0.003725	0.04979	palmitoyltransferase ZDHHC23, transcript variant X3
LOC101892359	2397.458	2852.893	-0.25092	5.23E-05	0.001593	26S proteasome non-ATPase regulatory subunit 12
LOC101892370	336.5417	440.7173	-0.38907	0.002571	0.037557	dipeptidase 3
LOC101892377	638.3761	503.4642	0.34252	0.000926	0.016617	selenium-binding protein 1
LOC101892386	2120.511	1520.419	0.47994	1.33E-12	2.14E-10	scavenger receptor class B member 1
LOC101892399	5784.713	6754.229	-0.22355	1.66E-05	0.000581	facilitated trehalose transporter Tret1
LOC101892419	3098.171	2665.804	0.21685	0.002543	0.037272	aspartate aminotransferase, cytoplasmic
LOC101892451	167.8995	98.45988	0.76999	0.000115	0.003082	alpha-tocopherol transfer protein
LOC101892457	38.83655	14.03432	1.4685	0.001478	0.024467	extensin-3
LOC101892460	699.2018	508.3275	0.45995	3.87E-06	0.000164	laminin subunit alpha-2, transcript variant X1
LOC101892468	4756.202	5480.994	-0.20463	0.000151	0.00385	odorant receptor 1a-like
LOC101892510	3528.936	4312.199	-0.28919	2.01E-07	1.20E-05	GTP cyclohydrolase 1, transcript variant X2
LOC101892515	1026.48	798.0143	0.36322	0.002918	0.041334	retinol-binding protein pinta
LOC101892526	181.7907	108.419	0.74566	0.000125	0.003307	A-kinase anchor protein 1, mitochondrial
LOC101892538	346.3134	227.2324	0.60791	2.15E-05	0.000734	proton-coupled amino acid transporter-like protein CG1139
LOC101892565	416.2879	538.9294	-0.37251	0.001121	0.019316	centrosomin, transcript variant X1
LOC101892601	3179.09	2683.437	0.24453	2.54E-05	0.00085	putative fatty acyl-CoA reductase CG8306
LOC101892606	21229.87	25039.18	-0.23809	3.34E-07	1.91E-05	uncharacterized LOC101892606
LOC101892623	5953.018	7040.895	-0.24214	2.61E-06	0.000117	A-kinase anchor protein 17A, transcript variant X1
LOC101892632	384.3147	498.1056	-0.37416	0.002015	0.031059	uncharacterized protein DDB_G0271670-like
LOC101892633	573.2409	702.5406	-0.29344	0.002388	0.035576	uncharacterized LOC101892633
LOC101892636	278.8169	156.4798	0.83334	3.32E-07	1.90E-05	probable cytochrome P450 318a1
LOC101892644	1371.45	1135.398	0.2725	0.000782	0.014438	uncharacterized LOC101892644, transcript variant X2
LOC101892660	1606.848	1968.726	-0.29303	2.33E-05	0.000789	UDP-glucuronosyltransferase 2B31
LOC101892668	211.1747	344.7161	-0.70697	6.67E-07	3.56E-05	uncharacterized LOC101892668

LOC101892672	413.0522	275.077	0.58649	0.00036	0.00778	dolichol kinase
LOC101892708	12470.66	13955.18	-0.16226	0.000444	0.009118	zinc finger protein on ecdysone puffs
LOC101892720	38.78094	95.97436	-1.3073	5.14E-06	0.000211	putative leucine-rich repeat-containing protein DDB_G0290503
LOC101892729	3904.682	4605.848	-0.23826	1.92E-05	0.000659	phosphoserine phosphatase
LOC101892760	4606.209	3069.751	0.58546	2.85E-25	1.35E-22	protein FAM188B2
LOC101892765	204.2694	366.5064	-0.84337	2.47E-09	2.20E-07	UDP-glucuronosyltransferase-like
LOC101892767	123.8604	181.1653	-0.54859	0.00335	0.046279	uncharacterized LOC101892767
LOC101892779	600.5366	760.1169	-0.33997	0.000422	0.008746	platelet glycoprotein Ib alpha chain, transcript variant X1
LOC101892787	5.11929	32.26187	-2.6558	2.83E-05	0.000935	uncharacterized LOC101892787
LOC101892793	1662.704	1437.479	0.20999	0.003133	0.043846	probable serine/threonine-protein kinase kinX, transcript variant X2
LOC101892807	5160.462	4604.765	0.16437	0.002728	0.039353	bax inhibitor 1
LOC101892829	855.8792	661.5959	0.37146	4.62E-05	0.001441	peptide methionine sulfoxide reductase
LOC101892844	6288.809	7845.988	-0.31917	6.92E-10	6.98E-08	mycosubtilin synthase subunit C
LOC101892852	2450.701	2064.526	0.24738	0.003524	0.047718	uncharacterized LOC101892852
LOC101892858	113.8415	44.11547	1.3677	3.61E-07	2.03E-05	odorant receptor 33b-like
LOC101892928	5596.463	6294.192	-0.16951	0.001724	0.027723	E3 ubiquitin-protein ligase KCMF1, transcript variant X5
LOC101892938	1967.161	2607.593	-0.4066	4.56E-07	2.50E-05	UDP-glucuronosyltransferase
LOC101892956	1080.636	1299.022	-0.26555	0.000694	0.01302	uncharacterized LOC101892956
LOC101892992	735.7339	961.9124	-0.38672	0.000301	0.006737	hydroxymethylglutaryl-CoA synthase 1, transcript variant X2
LOC101892999	1693.007	1464.923	0.20876	0.003092	0.043405	farnesol dehydrogenase
LOC101893008	990.3572	1317.147	-0.4114	1.87E-07	1.13E-05	protein grindelwald
LOC101893013	429.8711	301.0521	0.51389	0.000125	0.003307	uncharacterized LOC101893013, transcript variant X2
LOC101893073	871.9371	1073.12	-0.29952	0.000321	0.007075	1-aminocyclopropane-1-carboxylate oxidase, transcript variant X1
LOC101893080	1133.229	921.039	0.29911	0.000276	0.006343	ubiquitin-conjugating enzyme E2-22 kDa
LOC101893090	25.11677	60.94895	-1.279	0.000365	0.007876	synaptic vesicular amine transporter
LOC101893093	2594.618	2210.37	0.23123	0.000197	0.004823	protein lifeguard 1, transcript variant X3
LOC101893109	2961.566	3397.783	-0.19823	0.001073	0.018666	uncharacterized LOC101893109

LOC101893113	591.0855	763.5197	-0.3693	0.000186	0.004607	lipase 3
LOC101893116	33145.7	37688.53	-0.1853	0.000445	0.009128	UDP-glucuronosyltransferase 2A3-like
LOC101893122	197.8919	293.0284	-0.56633	0.000168	0.004219	protein suppressor of variegation 3-7, transcript variant X1
LOC101893144	1148.406	908.1138	0.33869	2.96E-05	0.000968	homeobox protein homothorax, transcript variant X1
LOC101893159	841.792	626.2607	0.4267	8.13E-06	0.000314	insulin-like growth factor-binding protein complex acid labile subunit, transcript variant X2
LOC101893174	14.59465	46.11634	-1.6598	0.00021	0.005072	uncharacterized LOC101893174
LOC101893181	1054.73	1310.892	-0.31368	0.000762	0.014143	death domain-associated protein 6, transcript variant X1
LOC101893183	594.1846	360.4908	0.72095	2.35E-10	2.53E-08	circadian clock-controlled protein
LOC101893184	673.3868	962.1945	-0.51489	1.27E-08	1.01E-06	peptidoglycan-recognition protein SB1
LOC101893188	2734.58	2311.345	0.24259	0.000728	0.013606	putative mediator of RNA polymerase II transcription subunit 26, transcript variant X1
LOC101893192	15.92562	1.760055	3.1777	0.000437	0.008998	acidic mammalian chitinase
LOC101893236	17.65731	46.46869	-1.396	0.000538	0.010689	odorant receptor 67d-like
LOC101893252	227.2203	152.2975	0.5772	0.000481	0.0097	menin
LOC101893257	2227.346	2594.777	-0.22029	0.000645	0.012272	ribose-phosphate pyrophosphokinase 1, transcript variant X3
LOC101893268	596.4241	463.1411	0.36489	0.000546	0.010785	vacuolar protein sorting-associated protein 11 homolog
LOC101893274	145.403	227.9401	-0.6486	0.00015	0.003825	venom dipeptidyl peptidase 4, transcript variant X1
LOC101893282	1458.927	1826.709	-0.32434	3.49E-06	0.00015	integral membrane protein GPR155
LOC101893291	6054.582	6985.238	-0.20628	5.72E-05	0.001718	UDP-glucuronosyltransferase 2A3-like
LOC101893292	380.4726	292.8077	0.37784	0.00343	0.046847	leucine-rich PPR motif-containing protein, mitochondrial
LOC101893309	1626.378	1371.979	0.2454	0.000616	0.011819	SUN domain-containing ossification factor
LOC101893324	221.3333	337.5923	-0.60906	3.18E-05	0.001036	calcium-binding protein P
LOC101893329	3776.722	2281.409	0.72721	9.46E-35	9.22E-32	dynein beta chain, ciliary
LOC101893343	74101.63	80854.94	-0.12583	0.003106	0.043532	protein takeout, transcript variant X2
LOC101893350	30.27037	89.63921	-1.5662	2.86E-07	1.66E-05	sarcotoxin II-1-like
LOC101893354	390.5544	628.8046	-0.68709	4.55E-10	4.67E-08	pentatricopeptide repeat-containing protein 1, mitochondrial
LOC101893377	960.9331	1239.577	-0.36734	6.42E-06	0.000253	uncharacterized LOC101893377, transcript variant X1

LOC101893429	9614.958	8643.374	0.15369	0.002317	0.034687	eukaryotic translation initiation factor 5
LOC101893442	22.12941	92.01197	-2.0559	1.35E-10	1.54E-08	cuticle protein 1
LOC101893459	172.658	92.13083	0.90616	0.000554	0.010879	venom peptide Pc
LOC101893468	114.0001	185.7772	-0.70454	0.000162	0.004067	uncharacterized LOC101893468
LOC101893473	3542.282	2825.419	0.32621	5.10E-08	3.49E-06	stress-associated endoplasmic reticulum protein 2, transcript variant X1
LOC101893479	2.960125	76.41352	-4.6901	8.32E-20	3.11E-17	uncharacterized LOC101893479
LOC101893494	1984.225	1474.098	0.42874	1.14E-09	1.10E-07	alpha-tocopherol transfer protein-like
LOC101893499	110.4374	61.60994	0.84199	0.000558	0.010933	sialin-like
LOC101893502	2974.564	4033.923	-0.43951	1.20E-08	9.62E-07	failed axon connections, transcript variant X1
LOC101893522	7478.623	8613.988	-0.20391	6.62E-05	0.00193	probable cytochrome P450 12c1, mitochondrial
LOC101893532	3088.187	3974.034	-0.36384	1.05E-10	1.22E-08	sodium-independent sulfate anion transporter
LOC101893567	1183.092	985.3348	0.26388	0.001034	0.018167	D-xylose-proton symporter
LOC101893573	2873.005	3451.672	-0.26473	7.70E-06	0.000298	uncharacterized LOC101893573, transcript variant X1
LOC101893582	1221.2	944.7394	0.37031	3.03E-06	0.000133	far upstream element-binding protein 1, transcript variant X1
LOC101893610	322.4713	502.8079	-0.64084	9.60E-08	6.17E-06	uncharacterized LOC101893610
LOC101893611	31.19471	71.33546	-1.1933	0.002097	0.032133	facilitated trehalose transporter Tret1, transcript variant X1
LOC101893614	174.7755	252.6054	-0.53138	0.000979	0.017437	uncharacterized LOC101893614
LOC101893616	1732.882	1406.124	0.30145	0.001706	0.027521	GATA zinc finger domain-containing protein 16
LOC101893622	608.7827	744.3923	-0.29014	0.003483	0.047382	serine-rich adhesin for platelets
LOC101893641	1282.632	1007.533	0.34828	9.08E-06	0.000344	aminopeptidase N
LOC101893644	10.93107	60.11101	-2.4592	5.84E-08	3.91E-06	sarcocystatin-A
LOC101893655	730.1375	567.4017	0.3638	0.000642	0.012259	Major Facilitator Superfamily protein
LOC101893656	2028.255	1678.807	0.2728	7.00E-05	0.00203	endoribonuclease dcr-1, transcript variant X3
LOC101893684	2535.883	2032.357	0.31933	1.05E-05	0.000388	neuroplastin, transcript variant X2
LOC101893728	52.60804	93.07917	-0.82318	0.002253	0.033932	uncharacterized LOC101893728
LOC101893731	2712	2118.461	0.35634	1.47E-08	1.15E-06	venom carboxylesterase-6
LOC101893736	1616.841	1366.897	0.24227	0.000859	0.015579	uncharacterized LOC101893736

LOC101893737	7047.208	8478.784	-0.26681	0.001906	0.029976	RNA-binding protein Rsf1
LOC101893760	102.5443	182.2042	-0.82931	2.42E-05	0.000815	multifunctional protein ADE2
LOC101893822	1841.06	1585.173	0.2159	0.002215	0.033532	serine/threonine-protein phosphatase 2A activator
LOC101893834	647.8843	881.2201	-0.44377	2.47E-06	0.000111	microprocessor complex subunit DGCR8, transcript variant X1
LOC101893852	65.43831	280.4427	-2.0995	9.33E-29	5.85E-26	sarcotoxin-2A-like
LOC101893875	12534.93	14358.03	-0.1959	0.00014	0.003623	lipase 3-like
LOC101893876	523.8545	658.7426	-0.33055	0.000995	0.017644	uncharacterized LOC101893876
LOC101893895	16553.85	19003.76	-0.19912	2.93E-05	0.000961	thioredoxin reductase 1, mitochondrial-like
LOC101893898	1110.835	832.35	0.41638	7.15E-07	3.75E-05	E3 ubiquitin-protein ligase MARCH5
LOC101893904	651.5054	492.6744	0.40314	0.000126	0.003323	adenylate cyclase type 2
LOC101893919	1281.255	1666.573	-0.37933	3.38E-07	1.93E-05	uncharacterized LOC101893919, transcript variant X2
LOC101893922	2062.718	1624.914	0.34418	2.86E-07	1.66E-05	glutamine synthetase 1, mitochondrial
LOC101893928	3543.654	3989.341	-0.17091	0.003529	0.047728	putative uncharacterized protein DDB_G0271606, transcript variant X8
LOC101893931	703.3389	902.3974	-0.35954	9.11E-05	0.002545	SPRY domain-containing SOCS box protein 3
LOC101893954	3435.605	2979.227	0.20563	0.000459	0.009382	DNA topoisomerase 2, transcript variant X1
LOC101893959	1081.719	1430.696	-0.40339	1.94E-07	1.17E-05	2-hydroxyacylsphingosine 1-beta-galactosyltransferase
LOC101893986	1719.544	2110.447	-0.29552	1.97E-05	0.000674	proteasome subunit beta type-7
LOC101893992	391.5355	241.4593	0.69736	1.29E-07	7.99E-06	probable serine/threonine-protein kinase kinX
LOC101893997	201.6127	288.8972	-0.51897	0.000417	0.008669	putative sodium-dependent multivitamin transporter
LOC101894012	3165.912	2727.814	0.21488	0.00031	0.006888	adipocyte plasma membrane-associated protein, transcript variant X1
LOC101894014	5472.907	7125.811	-0.38075	2.77E-13	4.86E-11	serine protease 7
LOC101894016	1768.413	2620.54	-0.56741	1.68E-17	5.45E-15	chymotrypsin-elastase inhibitor ixodidin
LOC101894031	25.7129	6.172037	2.0587	0.002023	0.031145	fibroblast growth factor receptor homolog 1
LOC101894034	4914.583	3902.543	0.33265	2.94E-08	2.13E-06	uncharacterized LOC101894034
LOC101894050	6559.138	9552.684	-0.5424	2.42E-27	1.25E-24	lipase 3-like
LOC101894065	591.9548	436.1871	0.44054	0.00278	0.03996	uncharacterized protein CG45076

LOC101894072	86399.45	73108.92	0.24097	3.27E-08	2.31E-06	protein timeless
LOC101894088	398.9437	307.982	0.37334	0.002911	0.041267	esterase GA18864
LOC101894106	4514.352	5874.303	-0.3799	1.15E-12	1.87E-10	apoptotic chromatin condensation inducer in the nucleus
LOC101894109	24.75566	79.42615	-1.6819	3.89E-07	2.18E-05	uncharacterized LOC101894109, transcript variant X2
LOC101894143	3908.217	3484.355	0.16562	0.002567	0.037544	vacuolar protein sorting-associated protein 13C, transcript variant X3
LOC101894145	4631.247	6271.56	-0.43742	1.62E-16	4.50E-14	glutamic acid-rich protein
LOC101894147	1208.71	1586.701	-0.39256	1.41E-07	8.66E-06	transcription factor Adf-1
LOC101894157	1551.761	1878.891	-0.27598	9.15E-05	0.002549	run domain Beclin-1-interacting and cysteine-rich domain-containing protein
LOC101894167	979.0196	451.5067	1.1166	1.27E-08	1.01E-06	metallothionein-1-like, transcript variant X3
LOC101894179	1049.552	1601.837	-0.60995	1.49E-15	3.58E-13	probable asparagine synthetase [glutamine-hydrolyzing]
LOC101894196	74.6276	129.2333	-0.7922	0.000693	0.01302	antitrypsin
LOC101894206	3265.199	3663.859	-0.16619	0.00312	0.04369	V-type proton ATPase 116 kDa subunit a, transcript variant X10
LOC101894209	5473.654	4908.609	0.15719	0.002696	0.039036	ATP-binding cassette sub-family E member 1, transcript variant X1
LOC101894233	158.1324	100.0368	0.6606	0.001195	0.020411	protein FAM91A1
LOC101894252	1136.375	1436.143	-0.33776	1.18E-05	0.000429	jmjC domain-containing histone demethylation protein 1
LOC101894258	586.9298	461.3047	0.34747	0.001624	0.026409	dehydrogenase/reductase SDR family member 7
LOC101894270	630.6023	445.1155	0.50255	2.65E-06	0.000118	tropomodulin, transcript variant X10
LOC101894288	956.0253	784.6483	0.285	0.001057	0.018477	uncharacterized LOC101894288
LOC101894308	226.7867	117.8427	0.94447	6.45E-08	4.30E-06	peroxisomal membrane protein PEX14
LOC101894316	2451.008	2904.139	-0.24474	6.69E-05	0.001946	26S protease regulatory subunit 6B
LOC101894319	784.2691	978.6347	-0.31942	0.000386	0.008212	probable serine/threonine-protein kinase yakA
LOC101894321	10234.8	8770.714	0.22272	4.83E-06	0.0002	uncharacterized protein CG45076
LOC101894328	2081.26	1640.667	0.34317	7.85E-07	4.07E-05	DNA topoisomerase 1, transcript variant X2
LOC101894334	5088.927	4559.666	0.15843	0.002146	0.032703	troponin C-akin-1 protein
LOC101894344	5638.687	4381.557	0.36392	6.20E-11	7.72E-09	basic-leucine zipper transcription factor A-like

LOC101894352	263.3185	179.5742	0.55223	0.000401	0.008441	protein outspread
LOC101894367	405.9468	242.5165	0.74321	2.94E-08	2.13E-06	inositol oxygenase
LOC101894389	23.65262	5.231968	2.1766	0.000993	0.017618	metallothionein-1
LOC101894395	1191.978	932.8896	0.35358	8.37E-06	0.000321	protein real-time
LOC101894398	12383.47	14232.29	-0.20075	0.000127	0.003347	sialin
LOC101894427	11786.88	13012.43	-0.14271	0.00178	0.028424	14-3-3 protein epsilon
LOC101894437	367.8413	269.3677	0.44951	0.000795	0.014657	protein SMG5, transcript variant X3
LOC101894451	1077.036	807.3621	0.41578	6.02E-07	3.27E-05	transcription factor GAGA, transcript variant X1
LOC101894457	10292.31	9082.164	0.18046	0.000306	0.006818	ribonucleoprotein RB97D
LOC101894461	2673.93	2358.466	0.18111	0.002681	0.03886	uncharacterized LOC101894461, transcript variant X3
LOC101894473	2553.639	2160.009	0.24152	8.51E-05	0.002397	probable methylmalonate-semialdehyde dehydrogenase [acylating], mitochondrial
LOC101894477	1022.231	858.6356	0.2516	0.00288	0.040922	protein NASP homolog
LOC101894479	281.4285	204.1269	0.4633	0.002906	0.041223	carabin
LOC101894495	7954.841	10386.64	-0.38482	0.001544	0.025345	heterogeneous nuclear ribonucleoprotein K
LOC101894507	677.7291	861.0105	-0.34532	0.000481	0.009696	ankyrin-2, transcript variant X1
LOC101894508	153.2804	97.02482	0.65975	0.001352	0.022678	cGMP-specific 3',5'-cyclic phosphodiesterase
LOC101894515	1496.662	1259.15	0.2493	0.000638	0.012193	uncharacterized LOC101894515
LOC101894528	12207.7	14744.56	-0.27239	1.90E-05	0.000655	protein suppressor of white apricot
LOC101894546	4472.579	5026.519	-0.16845	0.002237	0.033785	uncharacterized LOC101894546
LOC101894557	626.4945	773.5725	-0.30423	0.003359	0.046311	DNA repair protein REV1
LOC101894565	946.9297	1155.774	-0.28753	0.000508	0.010175	general odorant-binding protein 28a
LOC101894597	877.2525	1245.724	-0.50592	8.81E-08	5.73E-06	alpha-ketoglutarate-dependent dioxygenase alkB homolog 6, transcript variant X1
LOC101894616	290.894	209.6537	0.47249	0.001976	0.030681	dynactin subunit 5
LOC101894621	1121.531	945.1345	0.24688	0.003508	0.047673	acidic mammalian chitinase
LOC101894657	892.9458	1095.919	-0.2955	0.000507	0.010175	uncharacterized LOC101894657, transcript variant X1
LOC101894663	2918.617	2496.321	0.22548	0.000342	0.007428	semaphorin-1A, transcript variant X2

LOC101894665	1915.36	1628.246	0.2343	0.000397	0.008403	peroxisomal acyl-coenzyme A oxidase 3, transcript variant X2
LOC101894698	313.2303	431.0923	-0.46077	0.000309	0.006862	FAST kinase domain-containing protein 5, mitochondrial
LOC101894707	938.0602	768.1568	0.28828	0.001392	0.023248	uncharacterized protein DDB_G0283357, transcript variant X1
LOC101894723	139.9538	395.4564	-1.4986	5.11E-24	2.36E-21	general odorant-binding protein 28a
LOC101894735	759.8844	1025.543	-0.43254	6.30E-07	3.41E-05	papilin, transcript variant X1
LOC101894736	2421.287	2075.23	0.2225	0.000723	0.013524	acidic leucine-rich nuclear phosphoprotein 32 family member A, transcript variant X1
LOC101894742	1729.674	1445.392	0.25904	0.000301	0.006734	CSC1-like protein 1, transcript variant X1
LOC101894751	2181.246	2507.198	-0.20092	0.001229	0.02093	neurexin-4, transcript variant X4
LOC101894772	805.8928	665.2754	0.27664	0.003265	0.045344	putative ATPase N2B
LOC101894797	7060.74	6165.9	0.19551	0.000222	0.005315	uncharacterized LOC101894797
LOC101894804	6342.753	7425.688	-0.22742	1.08E-05	0.000398	putative uncharacterized protein DDB_G0285119, transcript variant X2
LOC101894813	620.1062	769.8556	-0.31207	0.000935	0.016753	sarcocystatin-A
LOC101894839	806.24	1122.051	-0.47686	0.00073	0.013631	G-protein coupled receptor Mth2, transcript variant X3
LOC101894880	596.9674	743.1315	-0.31597	0.000773	0.014302	flocculation protein FLO11, transcript variant X1
LOC101894885	150.5262	324.1798	-1.1068	1.55E-09	1.47E-07	general odorant-binding protein 28a-like
LOC101894889	17.31701	85.60954	-2.3056	6.14E-11	7.69E-09	pro-resilin-like
LOC101894912	90.18869	47.70295	0.91887	0.00085	0.01547	pickpocket protein 28
LOC101894929	460.7996	580.3718	-0.33284	0.003352	0.046279	organic cation transporter protein, transcript variant X1
LOC101894960	2408.439	1568.96	0.61829	6.48E-11	8.01E-09	tryptophan--tRNA ligase, cytoplasmic
LOC101894983	4209.183	3609.683	0.22167	0.000839	0.015339	isocitrate dehydrogenase [NAD] subunit gamma, mitochondrial, transcript variant X1
LOC101895029	2043.983	1781.757	0.19808	0.002948	0.041684	putative mediator of RNA polymerase II transcription subunit 12
LOC101895032	467.8464	366.8627	0.35079	0.003396	0.046581	collagen alpha-1(III) chain, transcript variant X3
LOC101895046	1827.487	1449.11	0.33469	9.47E-07	4.80E-05	serine/threonine-protein kinase MARK2, transcript variant X3
LOC101895063	1010.984	1205.74	-0.25416	0.001837	0.02913	myosin-10
LOC101895074	1477.299	2026.182	-0.4558	6.60E-11	8.10E-09	peptidoglycan-recognition protein LB

LOC101895107	167.589	252.0862	-0.58899	0.000358	0.007739	two pore potassium channel protein sup-9
LOC101895128	23.37411	59.60504	-1.3505	0.000307	0.006843	pickpocket protein 28-like
LOC101895168	332.1627	578.4872	-0.8004	4.50E-12	6.76E-10	multidrug resistance protein homolog 49, transcript variant X1
LOC101895169	70.07956	133.8057	-0.93307	0.000208	0.005029	cell death abnormality protein 1
LOC101895175	1562.462	1942.267	-0.31392	3.37E-06	0.000146	TGF-beta receptor type-1, transcript variant X2
LOC101895193	1207.087	734.4522	0.71679	2.62E-17	8.35E-15	pro-resilin
LOC101895201	436.7625	722.6319	-0.72641	2.27E-12	3.62E-10	uncharacterized LOC101895201, transcript variant X1
LOC101895252	14329.85	18680.31	-0.3825	1.32E-16	3.75E-14	ATP-dependent RNA helicase dbp2
LOC101895253	134.7398	312.154	-1.2121	3.78E-14	7.46E-12	uncharacterized LOC101895253
LOC101895255	1584.329	1897.39	-0.26014	0.000411	0.008562	J domain-containing protein, transcript variant X1
LOC101895308	678.9455	482.8722	0.49165	2.05E-06	9.54E-05	peptidylglycine alpha-hydroxylating monooxygenase
LOC101895322	505.3614	382.5841	0.40154	0.000379	0.008116	protein cutoff
LOC101895334	1913.166	1409.259	0.44103	1.34E-08	1.05E-06	gustatory and odorant receptor 21a-like
LOC101895340	387.3407	277.6516	0.48033	0.000226	0.005376	Usher syndrome type-1G protein homolog
LOC101895356	6249.836	7394.073	-0.24255	2.40E-06	0.000109	UTP--glucose-1-phosphate uridylyltransferase, transcript variant X3
LOC101895357	76.04624	36.60648	1.0548	0.000521	0.010394	protein PIH1D3
LOC101895361	7.345952	58.45683	-2.9924	9.06E-10	8.89E-08	uncharacterized LOC101895361
LOC101895374	1525.518	1203.477	0.34209	1.25E-05	0.000452	sialin
LOC101895385	4361.7	5406.59	-0.30983	1.26E-08	1.01E-06	organic cation transporter protein, transcript variant X1
LOC101895413	1416.808	1668.221	-0.23567	0.00146	0.024213	protein doublesex, transcript variant X1
LOC101895448	3195.87	2273.132	0.49153	6.55E-16	1.64E-13	ABC transporter G family member 20
LOC101895455	3109.183	3890.658	-0.32348	3.40E-08	2.38E-06	60S ribosomal protein L10
LOC101895463	1690.963	2080.635	-0.29918	1.62E-05	0.000569	proteasome assembly chaperone 2
LOC101895479	1187.379	1695.119	-0.51361	4.93E-06	0.000204	proteasome subunit beta type-3
LOC101895482	220.4005	155.9925	0.49865	0.003029	0.042685	synaptic vesicle glycoprotein 2A, transcript variant X1
LOC101895494	464.1524	579.0086	-0.31899	0.002272	0.034166	cytoplasmic phosphatidylinositol transfer protein 1
LOC101895498	15558.99	17230.55	-0.14722	0.002259	0.033996	60S ribosomal protein L9

LOC101895519	846.8373	1022.219	-0.27155	0.001886	0.029744	odorant receptor 45a-like
LOC101895548	3509.754	4224.002	-0.26724	1.99E-06	9.29E-05	arginine/serine-rich protein PNISR, transcript variant X2
LOC101895559	0.992048	47.12162	-5.5698	1.62E-13	2.94E-11	sulfur globule protein CV3-like
LOC101895561	10.27039	133.0935	-3.6959	9.76E-29	5.90E-26	maltase A2
LOC101895597	5330.073	6502.488	-0.28684	5.54E-08	3.74E-06	syntaxin-binding protein 5, transcript variant X9
LOC101895606	1937.446	2283.079	-0.23683	0.000259	0.006006	26S proteasome non-ATPase regulatory subunit 14
LOC101895609	1042.049	760.2918	0.4548	1.74E-07	1.06E-05	twinkle protein, mitochondrial
LOC101895629	3952.074	7049.538	-0.83492	2.62E-55	5.11E-52	heat shock protein 70
LOC101895630	919.6577	1133.88	-0.3021	0.000325	0.007121	nuclear factor NF-kappa-B p110 subunit
LOC101895641	5.968304	29.29809	-2.2954	0.000738	0.013757	estrogen sulfotransferase
LOC101895643	741.6807	524.7775	0.49909	7.41E-07	3.87E-05	cytochrome b5-related protein-like
LOC101895654	233.4559	137.4935	0.76379	5.92E-06	0.000237	uncharacterized LOC101895654
LOC101895657	919.9103	1205.093	-0.38958	1.83E-06	8.63E-05	clusterin-associated protein 1 homolog
LOC101895658	336.0133	486.4574	-0.5338	6.20E-06	0.000246	muscle M-line assembly protein unc-89, transcript variant X1
LOC101895661	496.7686	385.6705	0.3652	0.002854	0.040723	E3 ubiquitin-protein ligase RNF126-B
LOC101895712	1770.238	2032.157	-0.19907	0.002836	0.040508	coiled-coil domain-containing protein 47
LOC101895719	4633.347	4152.354	0.15813	0.002511	0.036927	band 3 anion transport protein, transcript variant X1
LOC101895728	804.6109	614.6253	0.38858	5.38E-05	0.00163	prolyl 4-hydroxylase subunit alpha-1
LOC101895735	612.6074	268.094	1.1922	9.34E-20	3.41E-17	cuticle protein CP14.6
LOC101895767	688.7732	495.1904	0.47605	0.001926	0.030101	UPF0687 protein C20orf27 homolog, transcript variant X1
LOC101895795	1102.029	1931.276	-0.80939	1.09E-28	6.36E-26	putative fatty acyl-CoA reductase CG5065, transcript variant X1
LOC101895798	1107.697	931.8902	0.24933	0.003255	0.045295	SPARC
LOC101895808	2.931744	25.75327	-3.1349	1.67E-05	0.000582	membrane-bound alkaline phosphatase
LOC101895818	79.47985	142.9961	-0.84731	9.12E-05	0.002545	uncharacterized LOC101895818
LOC101895819	3245.06	3874.492	-0.25576	9.87E-06	0.00037	peroxisomal targeting signal 1 receptor
LOC101895820	3087.018	2662.24	0.21357	0.00039	0.008275	inosine-5'-monophosphate dehydrogenase
LOC101895831	5082.87	6090.338	-0.26088	8.38E-07	4.31E-05	23 kDa integral membrane protein
LOC101895834	3.936155	44.01334	-3.4831	7.46E-10	7.43E-08	protein rhomboid

LOC101895846	3377.849	3947.473	-0.22482	9.36E-05	0.0026	serine/threonine-protein phosphatase 2A 56 kDa regulatory subunit epsilon isoform, transcript variant X4
LOC101895849	2292.856	2700.621	-0.23615	0.000191	0.004702	sodium-coupled monocarboxylate transporter 1
LOC101895852	318.6295	188.3044	0.75881	3.43E-07	1.94E-05	probable multidrug resistance-associated protein lethal(2)03659
LOC101895858	830.372	631.0573	0.39599	0.000319	0.007047	zinc finger protein 616
LOC101895876	30.16052	85.1901	-1.498	1.74E-06	8.29E-05	phospholipase B1, membrane-associated
LOC101895886	2892.215	3290.056	-0.18594	0.002301	0.034506	platelet-derived growth factor receptor alpha, transcript variant X3
LOC101895915	17659.41	20527.35	-0.21711	3.44E-06	0.000148	cytochrome P450 4e3-like
LOC101895954	639.2485	792.9827	-0.31091	0.00139	0.023248	uncharacterized LOC101895954
LOC101895985	991.5399	757.4872	0.38845	0.00024	0.005678	serine/arginine repetitive matrix protein 2
LOC101895987	277.8993	452.3064	-0.70274	0.00273	0.039353	protein quiver, transcript variant X1
LOC101895991	1717.909	2004.225	-0.22239	0.001046	0.018331	probable proteasome subunit beta type-2
LOC101896011	6616.063	8378.147	-0.34066	1.44E-11	2.09E-09	uncharacterized LOC101896011
LOC101896014	209.423	284.2376	-0.44068	0.003533	0.047728	serine/threonine-protein phosphatase Pgam5, mitochondrial, transcript variant X2
LOC101896015	1035.569	1332.32	-0.36352	0.002715	0.039278	sphingosine kinase 2
LOC101896022	7.501349	38.04621	-2.3425	7.91E-05	0.002253	uncharacterized protein YMR317W-like
LOC101896043	2566.908	3835.131	-0.57924	2.31E-23	1.01E-20	zinc-type alcohol dehydrogenase-like protein C1773.06c, transcript variant X3
LOC101896064	2097.175	2744.122	-0.3879	6.83E-06	0.000268	CLK4-associating serine/arginine rich protein
LOC101896069	330.0479	239.6369	0.46183	0.000572	0.011164	flocculation protein FLO11
LOC101896074	110.1555	63.13369	0.80306	0.00173	0.027746	uncharacterized protein KIAA1841 homolog
LOC101896084	1517.671	1143.197	0.40879	0.000488	0.009824	dual specificity protein phosphatase 13, transcript variant X1
LOC101896089	4.547405	19.92992	-2.1318	0.003264	0.045344	maltase A1
LOC101896092	476.14	346.3124	0.45931	0.00017	0.004263	uncharacterized LOC101896092
LOC101896097	211.7449	135.1514	0.64775	0.000299	0.006706	MICOS complex subunit MIC27-like
LOC101896109	22656.2	24919.85	-0.13739	0.002824	0.040412	antennal-specific protein OS-C, transcript variant X2
LOC101896112	3637.505	2584.093	0.49329	3.28E-16	8.60E-14	uncharacterized LOC101896112

LOC101896114	1266.893	1511.151	-0.25435	0.000651	0.012371	hexokinase-1
LOC101896155	2602.365	3292.935	-0.33955	1.34E-08	1.05E-06	protein scylla
LOC101896168	1438.153	1072.975	0.4226	2.83E-08	2.08E-06	diphosphomevalonate decarboxylase
LOC101896171	2317.955	2826.708	-0.28627	1.22E-05	0.000442	uncharacterized LOC101896171
LOC101896172	725.1749	916.4416	-0.33771	0.000162	0.004069	facilitated trehalose transporter Tret1, transcript variant X4
LOC101896174	1513.843	1124.271	0.42923	1.63E-08	1.26E-06	endoplasmic reticulum resident protein 44, transcript variant X1
LOC101896194	298.9555	189.0219	0.66138	8.79E-06	0.000335	MATH and LRR domain-containing protein PFE0570w
LOC101896204	1129.189	1481.35	-0.39163	4.02E-07	2.24E-05	probable cytochrome P450 6v1
LOC101896206	700.6303	482.7752	0.5373	1.66E-07	1.02E-05	aminopeptidase N, transcript variant X2
LOC101896212	1516.335	1765.823	-0.21975	0.002224	0.033644	transmembrane and coiled-coil domains protein 2
LOC101896218	1440.061	2322.877	-0.68978	1.13E-23	5.07E-21	tyrosine 3-monooxygenase
LOC101896233	7879.297	6410.038	0.29773	2.96E-09	2.63E-07	glucose-6-phosphate 1-dehydrogenase, transcript variant X1
LOC101896252	2196.488	1917.349	0.19609	0.002636	0.038321	signal recognition particle 54 kDa protein
LOC101896253	5545.604	6289.524	-0.18161	0.000334	0.007292	CCHC-type zinc finger protein CG3800
LOC101896256	343.473	440.8959	-0.36024	0.002147	0.032703	leucine-rich repeats and immunoglobulin-like domains protein 1
LOC101896258	3595.617	2930.384	0.29515	2.40E-07	1.43E-05	troponin C, skeletal muscle-like, transcript variant X2
LOC101896288	15111.17	17087.99	-0.17737	0.000215	0.005158	uncharacterized LOC101896288
LOC101896324	5435.988	6471.251	-0.2515	1.81E-06	8.57E-05	homeotic protein female sterile, transcript variant X2
LOC101896327	4048.698	4725.22	-0.22292	8.18E-05	0.002319	T-box protein H15
LOC101896328	4329.524	3775.52	0.19753	0.000472	0.009581	uncharacterized LOC101896328
LOC101896347	480.0002	650.3775	-0.43824	0.00041	0.00856	vesicular inhibitory amino acid transporter, transcript variant X1
LOC101896369	816.4294	1069.473	-0.3895	4.42E-06	0.000185	odorant receptor 67d-like
LOC101896371	1246.941	1559.466	-0.32266	6.53E-05	0.001912	L-2-hydroxyglutarate dehydrogenase, mitochondrial
LOC101896375	456.7538	901.3878	-0.98073	1.20E-17	4.05E-15	uncharacterized LOC101896375
LOC101896409	6.093941	585.6742	-6.5866	2.28E-181	4.00E-177	maltase A1-like
LOC101896414	4103.784	4923.721	-0.26279	1.62E-06	7.76E-05	cytoplasmic dynein 2 heavy chain 1
LOC101896437	29590.24	26307.5	0.16965	0.000279	0.006405	cytochrome b5-like
LOC101896449	16.95954	43.20772	-1.3492	0.001732	0.027746	fibroleukin

LOC101896458	6849.921	8062.05	-0.23506	3.14E-06	0.000137	liprin-alpha-1, transcript variant X2
LOC101896459	366.2132	487.819	-0.41366	0.000408	0.008545	kelch domain-containing protein 3
LOC101896475	2090.798	2432.131	-0.21817	0.000645	0.012272	proteasome subunit alpha type-2
LOC101896480	971.8023	808.5933	0.26525	0.001924	0.030101	kelch-like protein 17
LOC101896485	3755.708	4876.222	-0.37668	2.32E-09	2.08E-07	uncharacterized LOC101896485, transcript variant X2
LOC101896508	138.7661	204.6197	-0.56029	0.002727	0.039353	uncharacterized LOC101896508
LOC101896511	210.4968	132.2279	0.67077	0.000248	0.005824	probable G-protein coupled receptor 52
LOC101896515	22.64593	49.73944	-1.1351	0.003585	0.048256	DNA-directed RNA polymerase II subunit RPB1
LOC101896516	2134.315	1753.787	0.2833	0.000682	0.012836	repressor of RNA polymerase III transcription MAF1 homolog
LOC101896521	137.9747	78.72295	0.80955	0.000284	0.006474	fasciclin-3, transcript variant X2
LOC101896527	738.3814	901.7794	-0.28841	0.001653	0.026761	xyloside xylosyltransferase 1
LOC101896535	30.7203	81.72751	-1.4116	5.38E-06	0.00022	glycine, alanine and asparagine-rich protein-like
LOC101896549	3362.991	3804.514	-0.17797	0.00201	0.031059	epsin-2, transcript variant X5
LOC101896552	82.2942	137.6473	-0.74211	0.001118	0.019298	uncharacterized LOC101896552
LOC101896556	11.78512	39.67426	-1.7512	0.000254	0.005933	putative transporter svop-1
LOC101896578	107.6034	61.75831	0.80102	0.000986	0.017548	neurexin-1, transcript variant X2
LOC101896588	263.1442	403.2262	-0.61574	3.71E-06	0.000158	cytochrome P450 4e2-like
LOC101896594	1715.692	2739.925	-0.67535	3.33E-05	0.001077	eukaryotic translation initiation factor 4E-binding protein 3
LOC101896601	7567.701	6611.166	0.19495	0.000103	0.002818	uncharacterized LOC101896601
LOC101896618	68.26864	140.7945	-1.0443	5.71E-06	0.000231	spindle assembly abnormal protein 6 homolog, transcript variant X2
LOC101896620	2220.045	2810.293	-0.34013	2.50E-08	1.85E-06	neural/ectodermal development factor IMP-L2, transcript variant X1
LOC101896627	112.892	175.1935	-0.63401	0.001321	0.02223	uncharacterized LOC101896627, transcript variant X1
LOC101896663	549.8977	718.9974	-0.38682	0.000253	0.005923	patronin
LOC101896672	1640.889	1345.23	0.28662	8.11E-05	0.002304	general odorant-binding protein 67, transcript variant X2
LOC101896676	2727.232	2283.19	0.25639	0.000188	0.004629	glycine-rich cell wall structural protein
LOC101896677	1250.252	1031.979	0.27681	0.000674	0.012711	sodium-coupled monocarboxylate transporter 2, transcript variant

						X3
LOC101896679	2053.724	2479.069	-0.27156	1.34E-05	0.000484	ATP-dependent RNA helicase WM6
LOC101896697	655.6894	839.8444	-0.35711	0.002086	0.031993	Ecdysteroid kinase, transcript variant X2
LOC101896703	1213.703	969.5498	0.32403	5.10E-05	0.001563	integrin alpha-PS2, transcript variant X8
LOC101896715	4792.188	6278.915	-0.38983	1.56E-13	2.84E-11	adenylosuccinate synthetase
LOC101896716	3.980554	38.77189	-3.284	1.80E-08	1.38E-06	ctenidin-1
LOC101896718	81.14609	26.76559	1.6001	1.02E-06	5.12E-05	inner centromere protein, transcript variant X1
LOC101896719	8705.294	9826.819	-0.17483	0.000287	0.006496	serine-arginine protein 55, transcript variant X6
LOC101896725	143.7803	85.04409	0.75758	0.000466	0.009472	phosphatidate cytidylyltransferase, mitochondrial
LOC101896732	26.97752	6.505502	2.052	0.001081	0.018728	uncharacterized LOC101896732, transcript variant X2
LOC101896759	129.46	78.00134	0.73094	0.002544	0.037272	dynein heavy chain 3, axonemal-like
LOC101896761	155.2102	80.41807	0.94863	1.36E-05	0.000488	vacuolar protein sorting-associated protein 26B-like
LOC101896782	2176.897	2543.998	-0.22482	0.000219	0.005251	protein slit
LOC101896797	1608.271	1280.756	0.32852	6.13E-06	0.000244	poly [ADP-ribose] polymerase
LOC101896810	4291.187	3535.536	0.27945	2.92E-07	1.70E-05	60 kDa heat shock protein, mitochondrial
LOC101896814	593.675	382.1281	0.63562	4.11E-09	3.51E-07	frizzled-3
LOC101896824	1229.098	1017.064	0.27319	0.000667	0.01262	ubiquitin carboxyl-terminal hydrolase 64E, transcript variant X3
LOC101896828	1029.964	860.028	0.26014	0.003373	0.046443	ero1-like protein
LOC101896843	16046.88	20721.66	-0.36885	1.72E-15	3.92E-13	band 7 protein AGAP004871-like
LOC101896857	184.809	396.3781	-1.1008	8.89E-14	1.68E-11	serine protease inhibitor Kazal-type 2
LOC101896868	0	10.53497	#NAME?	0.000412	0.008574	homeotic protein ultrabithorax, transcript variant X2
LOC101896879	1337.933	1013.241	0.40103	3.11E-07	1.80E-05	sodium-independent sulfate anion transporter, transcript variant X1
LOC101896887	580.0463	772.4519	-0.41328	1.53E-05	0.000544	asparagine synthetase domain-containing protein CG17486
LOC101896895	5552.31	7023.036	-0.33901	3.97E-07	2.22E-05	heterogeneous nuclear ribonucleoprotein 87F
LOC101896897	80.47054	340.4352	-2.0808	1.53E-33	1.34E-30	diptericin-D-like
LOC101896907	4024.044	3509.964	0.19719	0.00037	0.007952	peptidyl-prolyl cis-trans isomerase FKBP8
LOC101896928	6510.604	7734.634	-0.24854	1.24E-06	6.16E-05	venom protease

LOC101896929	5381.396	6068.322	-0.17332	0.00101	0.017835	cofilin/actin-depolymerizing factor homolog
LOC101896933	13.355	42.70202	-1.6769	0.000185	0.004576	protein D3
LOC101897025	2036.156	2592.728	-0.34862	5.30E-08	3.59E-06	GTP-binding protein 1
LOC101897043	2335.981	2657.618	-0.18611	0.003658	0.049031	probable 26S proteasome non-ATPase regulatory subunit 3, transcript variant X2
LOC101897044	44.28607	100.2739	-1.179	1.55E-05	0.000549	lysosome membrane protein 2, transcript variant X2
LOC101897046	8196.399	9265.73	-0.17691	0.000323	0.007099	protein ref(2)P
LOC101897050	1325.908	707.5715	0.90603	4.28E-28	2.42E-25	cystinosin homolog
LOC101897056	494.9508	1629.161	-1.7188	1.08E-57	2.37E-54	alpha-amylase 4N, transcript variant X2
LOC101897068	486.4212	318.8355	0.60939	1.62E-05	0.000569	uncharacterized LOC101897068
LOC101897069	33.05658	8.181744	2.0145	0.00014	0.003623	neuropeptide-like 3
LOC101897075	818.7699	1170.388	-0.51546	3.81E-06	0.000162	CD109 antigen
LOC101897086	227.3711	140.5953	0.6935	4.87E-05	0.001504	major facilitator superfamily domain-containing protein 6, transcript variant X1
LOC101897121	2624.164	3081.31	-0.23169	8.97E-05	0.002511	serine/threonine-protein kinase Tao
LOC101897146	1058.359	875.9879	0.27285	0.00155	0.025397	chitinase domain-containing protein 1
LOC101897160	2171.073	2623.252	-0.27295	1.51E-05	0.000538	26S protease regulatory subunit 4
LOC101897191	454.7197	353.2675	0.36422	0.003666	0.049111	uncharacterized LOC101897191
LOC101897194	879.5021	1149.026	-0.38565	0.000106	0.002891	double-headed protease inhibitor, submandibular gland
LOC101897200	52.62293	122.6969	-1.2213	9.49E-07	4.80E-05	leucine-rich repeat extensin-like protein 1
LOC101897204	2121.428	2527.546	-0.2527	6.97E-05	0.002026	proteasomal ubiquitin receptor ADRM1 homolog, transcript variant X3
LOC101897220	2146.474	2472.281	-0.20387	0.001077	0.01871	ubiquitin carboxyl-terminal hydrolase 14
LOC101897221	63.38486	116.8594	-0.88256	0.000378	0.008102	ejaculatory bulb-specific protein 3-like
LOC101897244	466.2932	794.1224	-0.76812	2.53E-14	5.10E-12	proline-rich extensin-like protein EPR1
LOC101897245	2078.934	2369.158	-0.18853	0.002463	0.036353	cleft lip and palate transmembrane protein 1 homolog
LOC101897279	2018.749	3012.699	-0.57759	1.96E-20	7.80E-18	probable cytochrome P450 28d1-like
LOC101897289	410.0221	615.5711	-0.58622	1.02E-07	6.50E-06	cytosolic carboxypeptidase 6

LOC101897290	6181.961	5252.073	0.23518	0.000668	0.012637	titin, transcript variant X2
LOC101897314	795.0583	961.3823	-0.27405	0.001419	0.023631	heat shock protein 23
LOC101897324	515.4325	660.9568	-0.35877	0.000476	0.009625	60S ribosomal export protein NMD3, transcript variant X1
LOC101897332	2505.197	2942.731	-0.23223	0.00014	0.003621	proteasome subunit alpha type-7-1
LOC101897335	2439.92	2818.375	-0.20803	0.000747	0.013902	mucin-5AC, transcript variant X1
LOC101897343	1269.352	1651.413	-0.37961	0.002616	0.038155	intraflagellar transport protein 56
LOC101897352	2903.744	2472.977	0.23167	9.72E-05	0.00269	cystathionine beta-synthase
LOC101897374	12005.31	10890.92	0.14055	0.002421	0.035882	eukaryotic translation initiation factor 4 gamma 2
LOC101897394	63.33062	180.6315	-1.5121	4.91E-11	6.39E-09	uncharacterized LOC101897394
LOC101897398	3143.066	3685.696	-0.22976	9.75E-05	0.002695	slowpoke-binding protein, transcript variant X1
LOC101897419	276.3783	371.1361	-0.4253	0.001072	0.018659	uncharacterized LOC101897419
LOC101897428	481.3539	345.6311	0.47786	0.000106	0.002891	phosphatidylserine decarboxylase proenzyme, mitochondrial, transcript variant X2
LOC101897443	907.0559	665.7397	0.44623	1.35E-06	6.61E-05	protein HEXIM1
LOC101897444	2412.218	2059.71	0.22792	0.000381	0.008151	putative aminopeptidase W07G4.4
LOC101897456	453.9137	566.343	-0.31926	0.003592	0.048256	apolipoprotein D
LOC101897457	8075.586	9710.307	-0.26595	1.26E-07	7.83E-06	uncharacterized LOC101897457, transcript variant X2
LOC101897467	2220.822	2532.352	-0.18938	0.003383	0.046492	26S proteasome non-ATPase regulatory subunit 6
LOC101897485	237.1305	146.2594	0.69715	0.000294	0.006627	Krueppel homolog 1
LOC101897488	266.7913	384.6786	-0.52794	5.24E-05	0.001593	vasotab-like
LOC101897490	5853.903	4586.787	0.35191	1.04E-05	0.000386	putative SERF-like protein, transcript variant X2
LOC101897501	1608.093	1862.261	-0.2117	0.002411	0.035797	esterase B1
LOC101897516	2346.537	1813.196	0.372	9.32E-09	7.79E-07	lysozyme
LOC101897522	4425.928	5309.85	-0.26269	1.39E-06	6.75E-05	scavenger receptor class B member 1
LOC101897526	4460.995	5635.173	-0.33709	1.86E-09	1.73E-07	probable serine/threonine-protein kinase DDB_G0282963
LOC101897538	31.6286	10.41761	1.6022	0.00166	0.02684	uncharacterized LOC101897538
LOC101897552	1091.255	816.9492	0.41767	6.56E-07	3.52E-05	sentrin-specific protease 8, transcript variant X2
LOC101897560	420.4285	649.6396	-0.62778	0.000118	0.00316	protein boule, transcript variant X6

LOC101897561	41.30968	82.50642	-0.99803	0.000636	0.012168	DNA-directed RNA polymerase II subunit RPB1, transcript variant X1
LOC101897571	128160.2	143423.6	-0.16233	0.000285	0.006475	ejaculatory bulb-specific protein 3
LOC101897583	1187.756	980.4291	0.27675	0.000664	0.012577	ubiquitin-protein ligase E3B
LOC101897587	4578.244	5884.868	-0.36222	1.61E-11	2.28E-09	cytochrome P450 4e2-like
LOC101897638	1029.761	791.6902	0.3793	1.09E-05	0.0004	cadherin-87A, transcript variant X1
LOC101897655	274.664	426.3888	-0.6345	6.56E-07	3.52E-05	heat shock protein 27
LOC101897660	2011.042	3179.889	-0.66103	3.65E-11	4.81E-09	vasotab
LOC101897667	4573.659	5419.655	-0.24485	7.15E-06	0.000278	potassium voltage-gated channel subfamily H member 6, transcript variant X1
LOC101897669	1573.862	1196.778	0.39515	6.49E-08	4.32E-06	cytochrome P450 4d2
LOC101897678	1336.901	1689.18	-0.33743	2.73E-06	0.000121	solute carrier family 25 member 36, transcript variant X2
LOC101897680	2505.34	2866.891	-0.19448	0.002159	0.032829	E3 ubiquitin-protein ligase RBBP6, transcript variant X1
LOC101897728	616.261	476.8079	0.37013	0.000398	0.008404	integrator complex subunit 6
LOC101897731	563.0309	695.5628	-0.30497	0.002826	0.040412	phospholipase B1, membrane-associated-like
LOC101897741	395.7065	539.7674	-0.44791	0.000773	0.014302	tropomyosin-2, transcript variant X1
LOC101897748	5273.8	6081.649	-0.20562	7.82E-05	0.002239	eukaryotic translation initiation factor 1A, X-chromosomal
LOC101897761	636.3662	477.2772	0.41503	8.72E-05	0.002449	collagen alpha-1(IV) chain
LOC101897767	1975.701	1526.918	0.37174	3.33E-08	2.34E-06	protein disulfide-isomerase A6 homolog, transcript variant X2
LOC101897774	25061.68	32603.1	-0.37953	1.93E-06	9.08E-05	latrophilin Cirl
LOC101897781	617.477	461.7834	0.41917	0.000111	0.002994	glutathione S-transferase theta-1
LOC101897791	1731.336	2080.84	-0.26528	0.000185	0.004575	UV excision repair protein RAD23 homolog B
LOC101897795	17368.79	20096.09	-0.21042	7.00E-06	0.000274	NADH-cytochrome b5 reductase 3, transcript variant X3
LOC101897797	23926.55	27533.3	-0.20257	1.08E-05	0.000398	glutathione S-transferase D7, transcript variant X4
LOC101897799	16709.81	14310.71	0.2236	1.45E-06	7.02E-05	catalase
LOC101897803	4654.72	5429.542	-0.22214	3.86E-05	0.001233	ctenidin-1
LOC101897810	3698.048	4539.181	-0.29567	1.01E-07	6.43E-06	E3 ubiquitin-protein ligase Nedd-4, transcript variant X6
LOC101897888	1117.021	1431.797	-0.35817	4.51E-06	0.000188	ras-responsive element-binding protein 1, transcript variant X1

LOC101897889	256.1724	344.9548	-0.42929	0.001908	0.029978	RNA-binding protein Nova-1
LOC101897909	17.84197	73.08648	-2.0343	2.88E-08	2.11E-06	ejaculatory bulb-specific protein 3-like
LOC101897932	1589.089	1297.879	0.29204	3.58E-05	0.00115	lipase 3-like
LOC101897944	3378.193	2871.094	0.23465	6.20E-05	0.001837	vigilin
LOC101897967	17498.98	20520.22	-0.22978	3.02E-06	0.000132	glycine-rich cell wall structural protein
LOC101897970	866.4516	1128.551	-0.38128	3.99E-06	0.000168	probable ATP-dependent RNA helicase DDX46, transcript variant X1
LOC101897978	3678.895	4356.453	-0.24388	4.31E-05	0.001363	serine/threonine-protein kinase PITSLRE, transcript variant X3
LOC101897982	315.9657	223.9004	0.49691	0.000321	0.007076	titin, transcript variant X4
LOC101897984	960.4269	1360.523	-0.50241	1.75E-10	1.92E-08	zinc transporter 2, transcript variant X5
LOC101897987	452.9472	566.8484	-0.32362	0.002415	0.035817	uncharacterized protein CG1785
LOC101898011	4163.318	4972.563	-0.25626	2.94E-06	0.000129	probable cytochrome P450 28c1
LOC101898023	44816.25	39988.24	0.16445	0.000154	0.003911	uncharacterized LOC101898023
LOC101898054	24283.35	27340.26	-0.17106	0.000261	0.00604	ABC transporter G family member 20
LOC101898060	2430.653	1986.942	0.29079	2.67E-06	0.000119	mucin-5AC
LOC101898082	1159.32	1383.92	-0.25548	0.001025	0.01805	probable glutamine--tRNA ligase
LOC101898087	324.2566	240.3059	0.43226	0.001727	0.027746	uncharacterized LOC101898087
LOC101898092	1.92986	15.05368	-2.9635	0.00189	0.029767	uncharacterized LOC101898092
LOC101898107	844.0607	2132.351	-1.337	2.88E-72	1.01E-68	cell division cycle 7-related protein kinase
LOC101898114	176.838	116.121	0.6068	0.001885	0.029744	mitogen-activated protein kinase kinase kinase 7, transcript variant X1
LOC101898118	13.42411	40.72061	-1.6009	0.000499	0.010016	uncharacterized LOC101898118
LOC101898127	801.1916	667.8306	0.26267	0.003406	0.046655	23 kDa integral membrane protein, transcript variant X1
LOC101898132	669.52	844.5465	-0.33505	0.00041	0.00856	fatty acyl-CoA reductase wat
LOC101898146	141.0753	207.6377	-0.5576	0.001569	0.025656	bumetanide-sensitive sodium-(potassium)-chloride cotransporter
LOC101898153	3126.909	3757.648	-0.26509	5.12E-06	0.000211	carboxypeptidase B
LOC101898174	1607.567	2070.658	-0.36521	4.41E-08	3.04E-06	uncharacterized LOC101898174, transcript variant X1
LOC101898179	1059.145	727.3762	0.54213	4.81E-10	4.91E-08	cysteine-rich with EGF-like domain protein 2, transcript variant

						X1
LOC101898207	136.5808	227.5994	-0.73674	1.64E-05	0.000575	uncharacterized LOC101898207
LOC101898210	863.3261	1058.8	-0.29445	0.001942	0.030242	gastrula zinc finger protein XlCGF26.1, transcript variant X1
LOC101898213	1869.284	2476.715	-0.40594	8.60E-10	8.52E-08	proteasome-associated protein ECM29 homolog
LOC101898215	5623.903	4891.608	0.20126	0.000134	0.003496	periostin, transcript variant X2
LOC101898238	1553.92	1124.194	0.46702	1.82E-10	1.97E-08	nicotinate phosphoribosyltransferase, transcript variant X1
LOC101898280	1342.834	1714.701	-0.35268	1.44E-06	6.97E-05	major heat shock 70 kDa protein Ba-like
LOC101898297	2131.643	1825.599	0.2236	0.000948	0.016949	UMP-CMP kinase
LOC101898304	1082.653	898.1967	0.26947	0.00181	0.028815	chitinase-like protein Idgf3
LOC101898322	2906.894	2290.564	0.34378	1.18E-08	9.57E-07	lysyl oxidase homolog 3, transcript variant X1
LOC101898329	197.6612	289.3374	-0.54972	0.000226	0.005376	uncharacterized LOC101898329
LOC101898334	0.97603	48.08978	-5.6227	1.64E-15	3.78E-13	uncharacterized LOC101898334
LOC101898335	4431.993	3670.833	0.27185	0.003588	0.048256	hepatic leukemia factor, transcript variant X1
LOC101898337	194.669	307.7334	-0.66066	1.02E-05	0.000379	transmembrane emp24 domain-containing protein 5
LOC101898352	0	15.58982	#NAME?	1.61E-06	7.70E-05	stomoxyn
LOC101898365	8393.416	10824.78	-0.36701	9.55E-14	1.76E-11	ejaculatory bulb-specific protein 3-like
LOC101898381	1074.701	1383.887	-0.36479	2.74E-05	0.000909	uncharacterized LOC101898381
LOC101898399	726.9544	999.4399	-0.45925	2.95E-07	1.71E-05	probable serine/threonine-protein kinase tsuA
LOC101898410	4626.053	3970.123	0.2206	4.26E-05	0.001352	mucin-5AC, transcript variant X1
LOC101898444	428.7648	282.5262	0.6018	1.76E-06	8.35E-05	synaptojanin-1
LOC101898450	169.2206	278.4465	-0.71849	5.41E-06	0.00022	heat shock protein 70-like
LOC101898511	757.9294	618.9766	0.29218	0.002802	0.040144	coronin-6, transcript variant X1
LOC101898526	3067.782	3508.817	-0.19379	0.00115	0.01973	esterase B1, transcript variant X1
LOC101898539	687.1621	1033.119	-0.58828	4.62E-11	6.06E-09	uncharacterized LOC101898539
LOC101898564	291.1348	216.3681	0.4282	0.003438	0.046907	uncharacterized LOC101898564
LOC101898587	752.3862	963.385	-0.35664	5.57E-05	0.00168	uncharacterized LOC101898587, transcript variant X2
LOC101898592	994.3221	754.4113	0.39836	2.91E-06	0.000128	sodium/hydrogen exchanger 8
LOC101898624	9775.575	11448.47	-0.2279	0.000123	0.003271	4-coumarate--CoA ligase 1-like

LOC101898630	4567.933	5431.435	-0.24979	5.71E-06	0.000231	RNA-binding protein cabeza, transcript variant X1
LOC101898640	8882.44	5032.424	0.8197	8.50E-58	2.13E-54	ecdysteroid-regulated 16 kDa protein
LOC101898644	881.2518	717.2733	0.29703	0.001078	0.01871	purine nucleoside phosphorylase, transcript variant X1
LOC101898646	4390.886	5397.085	-0.29767	4.27E-08	2.95E-06	glutamate receptor 2
LOC101898659	224.8978	142.6773	0.65651	0.000143	0.003688	exonuclease 1
LOC101898661	501.1125	675.886	-0.43165	0.000187	0.004618	peptidoglycan-recognition protein LC, transcript variant X1
LOC101898672	3664.687	4251.493	-0.21428	0.000106	0.00289	ankyrin repeat domain-containing protein 39
LOC101898677	1006.523	1265.516	-0.33035	4.99E-05	0.001535	trafficking protein particle complex subunit 10
LOC101898687	367.2615	486.2582	-0.40491	0.000575	0.01119	heat shock protein 22
LOC101898701	566.9081	746.8696	-0.39774	6.23E-05	0.00184	uncharacterized LOC101898701, transcript variant X1
LOC101898704	1411.625	1663.605	-0.23696	0.000838	0.01533	integrator complex subunit 10, transcript variant X1
LOC101898706	74.90358	132.292	-0.82062	0.001221	0.020816	uncharacterized LOC101898706, transcript variant X1
LOC101898708	3282.983	3836.385	-0.22474	0.00016	0.004048	probable pseudouridine-5'-phosphatase, transcript variant X3
LOC101898710	191.8694	125.3672	0.61396	0.001032	0.018152	transcription factor mef2A
LOC101898779	1357.644	1042.135	0.38156	1.25E-06	6.18E-05	uncharacterized LOC101898779
LOC101898780	1614.293	1990.675	-0.30236	8.37E-06	0.000321	glycerol-3-phosphate acyltransferase 3, transcript variant X1
LOC101898784	499.1256	390.9427	0.35245	0.002732	0.039353	D-2-hydroxyglutarate dehydrogenase, mitochondrial
LOC101898798	700.5977	890.8067	-0.34653	0.000125	0.003307	maltase 2, transcript variant X2
LOC101898824	219.3565	103.4498	1.0843	5.14E-09	4.34E-07	glycine-rich cell wall structural protein
LOC101898830	2435.263	3205.663	-0.39655	2.15E-11	2.99E-09	uncharacterized LOC101898830
LOC101898847	129.4083	239.8622	-0.89028	0.000669	0.012637	flightin
LOC101898872	6.918479	94.36275	-3.7697	1.05E-21	4.39E-19	uncharacterized LOC101898872
LOC101898873	1617.56	1963.999	-0.27998	0.000283	0.006449	proteasome subunit beta type-6
LOC101898874	1014.685	847.5573	0.25965	0.002672	0.038778	brefeldin A-inhibited guanine nucleotide-exchange protein 1
LOC101898875	1955.854	2306.959	-0.23819	0.000204	0.00497	type-1 angiotensin II receptor-associated protein
LOC101898881	21489.92	23854.54	-0.1506	0.001233	0.020982	60S ribosomal protein L4
LOC101898892	20.36479	57.32687	-1.4931	8.22E-05	0.002325	lectin subunit alpha
LOC101898895	11123.09	12369.9	-0.15328	0.001438	0.023897	60S ribosomal protein L11

LOC101898927	890.8952	715.4455	0.31641	0.000617	0.011828	nibrin
LOC101898975	522.088	664.6269	-0.34825	0.000938	0.01679	probable multidrug resistance-associated protein lethal(2)03659
LOC101898982	2092.903	3165.622	-0.59698	1.08E-21	4.41E-19	uncharacterized LOC101898982
LOC101899007	2357.443	2721.226	-0.20703	0.000991	0.01761	26S protease regulatory subunit 6A-B, transcript variant X1
LOC101899041	533.3916	400.7846	0.41237	0.000226	0.005376	alpha-tocopherol transfer protein-like, transcript variant X1
LOC101899051	355.5535	249.5079	0.51098	0.000193	0.004732	FAST kinase domain-containing protein 1, mitochondrial, transcript variant X1
LOC101899057	656.9743	887.548	-0.43399	2.18E-06	0.0001	uncharacterized LOC101899057
LOC101899080	2465.621	2813.249	-0.19029	0.0023	0.034506	proteasome subunit beta type-5
LOC101899102	245.3079	112.3555	1.1265	3.05E-10	3.20E-08	parkin coregulated gene protein homolog
LOC101899107	1563.775	2005.352	-0.35882	2.57E-07	1.52E-05	transport and Golgi organization protein 6
LOC101899122	1672.602	1445.732	0.21029	0.002987	0.042209	E3 ubiquitin-protein ligase Topors, transcript variant X1
LOC101899132	3636.402	4740.756	-0.38261	8.10E-08	5.30E-06	uncharacterized LOC101899132, transcript variant X2
LOC101899169	4767.393	4147.873	0.20083	0.000363	0.007835	endoplasmin homolog
LOC101899196	780.3681	950.1248	-0.28396	0.001101	0.019033	uncharacterized LOC101899196
LOC101899202	924.1016	1206.771	-0.38503	3.15E-05	0.001031	UDP-glucuronosyltransferase-like
LOC101899220	7.196986	33.3269	-2.2112	5.87E-05	0.001756	V-type proton ATPase 116 kDa subunit a
LOC101899235	275.6142	160.7514	0.77782	8.58E-07	4.40E-05	prisilkin-39
LOC101899297	778.1205	547.1756	0.50799	3.49E-07	1.97E-05	sarcocystatin-A
LOC101899304	4368.362	3546.612	0.30065	0.000109	0.002969	60S ribosomal protein L37a
LOC101899319	7.757656	156.8511	-4.3376	4.09E-33	3.26E-30	uncharacterized LOC101899319
LOC101899329	360.8547	256.5062	0.49242	0.000309	0.006862	tissue factor pathway inhibitor, transcript variant X1
LOC101899333	83.84966	149.622	-0.83545	0.000103	0.002833	uncharacterized LOC101899333
LOC101899347	515.6428	735.391	-0.51214	2.76E-07	1.63E-05	myb-like protein X, transcript variant X2
LOC101899371	3876.821	4445.359	-0.19743	0.000548	0.010807	protein mini spindles, transcript variant X3
LOC101899382	423.8236	327.8943	0.37023	0.002721	0.039333	activating transcription factor 7-interacting protein 1
LOC101899383	868.5285	1041.8	-0.26243	0.003713	0.049704	histone-lysine N-methyltransferase SETD1
LOC101899394	190.401	126.2497	0.59276	0.001408	0.023469	multiple epidermal growth factor-like domains protein 8,

						transcript variant X1
LOC101899430	1.286573	46.91838	-5.1885	2.00E-14	4.09E-12	adult cuticle protein 1-like
LOC101899440	315.5879	221.5704	0.51028	0.000425	0.008797	anosmin-1
LOC101899442	894.5751	1088.61	-0.28321	0.000711	0.013325	uncharacterized LOC101899442, transcript variant X3
LOC101899450	4.65335	21.7363	-2.2238	0.00108	0.018728	membrane-bound alkaline phosphatase-like
LOC101899460	2017.741	2348.008	-0.2187	0.000604	0.011654	uncharacterized LOC101899460
LOC101899471	344.3865	492.1094	-0.51495	1.28E-05	0.000462	chorion peroxidase, transcript variant X2
LOC101899495	15.94049	162.6181	-3.3507	1.59E-31	1.16E-28	uncharacterized LOC101899495
LOC101899506	93.64541	45.67219	1.0359	0.000285	0.006475	uncharacterized LOC101899506, transcript variant X3
LOC101899529	129.7672	38.72161	1.7447	3.53E-11	4.69E-09	pancreatic lipase-related protein 2-like
LOC101899539	524.4031	388.8857	0.43133	0.000233	0.00552	splicing factor U2AF 50 kDa subunit
LOC101899571	14151.62	22396.42	-0.6623	2.34E-44	2.93E-41	beta-galactosidase, transcript variant X1
LOC101899596	1058.188	1459.616	-0.46399	2.10E-09	1.92E-07	cyclin-dependent kinase F-4, transcript variant X2
LOC101899597	530.1773	667.8335	-0.33301	0.000911	0.016391	WD repeat-containing protein 47
LOC101899599	1520.008	1833.6	-0.2706	0.00012	0.003205	dual specificity tyrosine-phosphorylation-regulated kinase 2
LOC101899617	3332.131	4234.826	-0.34586	4.09E-10	4.24E-08	choline/ethanolamine kinase, transcript variant X1
LOC101899622	1509.069	1085.729	0.47499	3.40E-10	3.56E-08	supervillin, transcript variant X5
LOC101899629	2096.664	2739.636	-0.38589	7.42E-10	7.43E-08	focadhesin, transcript variant X5
LOC101899648	418.4214	544.6775	-0.38045	0.000763	0.014149	uncharacterized LOC101899648, transcript variant X2
LOC101899663	862.2204	1180.519	-0.45329	4.85E-08	3.32E-06	keratin-associated protein 19-2-like
LOC101899666	50.656	170.6395	-1.7521	3.76E-14	7.46E-12	maltase A3-like
LOC101899673	1443.836	1682.355	-0.22058	0.001731	0.027746	FAS-associated factor 1, transcript variant X1
LOC101899684	362.6249	215.2715	0.75232	2.71E-07	1.60E-05	vitellogenin-1-like
LOC101899689	165.88	108.3024	0.61508	0.001882	0.029744	uncharacterized LOC101899689
LOC101899738	400.3259	578.8453	-0.532	0.0004	0.008439	mitogen-activated protein kinase kinase kinase 13, transcript variant X2
LOC101899757	31919.48	35375.02	-0.14829	0.001065	0.01858	tubulin beta-1 chain
LOC101899781	8380.269	9557.762	-0.18968	0.000193	0.004741	V-type proton ATPase subunit S1

LOC101899788	3955.477	4916.498	-0.31378	1.88E-08	1.42E-06	uncharacterized LOC101899788, transcript variant X4
LOC101899801	2641.211	2062.419	0.35686	2.49E-08	1.85E-06	peptide transporter family 1, transcript variant X2
LOC101899813	3159.355	2459.983	0.36098	1.45E-09	1.39E-07	probable serine/threonine-protein kinase DDB_G0282963, transcript variant X3
LOC101899816	513.0039	326.6183	0.65136	1.98E-08	1.48E-06	synaptic vesicular amine transporter, transcript variant X8
LOC101899837	4.94422	73.95128	-3.9028	8.75E-18	3.01E-15	maltase A3-like
LOC101899850	310.3221	423.5823	-0.44888	0.000409	0.008555	zinc finger protein 470
LOC101899855	0.97603	13.14057	-3.751	0.000887	0.016026	leucine-rich repeat-containing G-protein coupled receptor 5
LOC101899861	665.255	340.9981	0.96414	3.22E-18	1.13E-15	solute carrier family 22 member 3, transcript variant X3
LOC101899904	3196.002	3906.331	-0.28955	0.000613	0.011799	T-box protein H15-like, transcript variant X1
LOC101899934	424.3177	598.2668	-0.49564	4.71E-06	0.000196	putative uncharacterized protein DDB_G0277255
LOC101899942	9.549516	29.11967	-1.6085	0.003582	0.048256	uncharacterized LOC101899942
LOC101899949	481.8146	363.0365	0.40836	0.00044	0.009051	39S ribosomal protein L18, mitochondrial
LOC101899972	0	6.97924	#NAME?	0.003398	0.046581	uncharacterized LOC101899972, transcript variant X3
LOC101899981	1.597117	13.0763	-3.0334	0.002837	0.040508	extensin-1
LOC101899990	1956.677	2436.067	-0.31615	1.57E-06	7.56E-05	proteasome subunit alpha type-1
LOC101900020	1617.401	1265.758	0.35368	0.000149	0.003823	probable serine/threonine-protein kinase dyrk2, transcript variant X2
LOC101900039	5801.869	6687.073	-0.20486	0.000114	0.003079	paired box protein Pax-3-A
LOC101900065	1781.213	1200.598	0.56911	2.87E-15	6.36E-13	probable cytochrome P450 6a14
LOC101900067	1319.808	1627.48	-0.30231	3.47E-05	0.001118	locomotion-related protein Hikaru genki, transcript variant X1
LOC101900106	13.34767	0.614216	4.4417	0.000282	0.006449	adult cuticle protein 1
LOC101900118	1015.982	612.5892	0.72988	1.47E-15	3.58E-13	uncharacterized LOC101900118
LOC101900121	360.3225	246.9405	0.54513	4.64E-05	0.001443	uncharacterized LOC101900121, transcript variant X2
LOC101900125	113.271	56.93271	0.99245	0.000225	0.005363	uncharacterized LOC101900125
LOC101900131	12.87947	36.18216	-1.4902	0.002014	0.031059	odorant receptor 63a
LOC101900136	1029.162	1519.146	-0.56179	4.29E-13	7.38E-11	aromatic-L-amino-acid decarboxylase, transcript variant X1
LOC101900157	5.682486	21.1233	-1.8942	0.003565	0.048127	protein amnionless

LOC101900158	4580.177	3856.887	0.24797	9.88E-06	0.00037	unconventional myosin-XVIIIa, transcript variant X2
LOC101900159	2280.62	3022.867	-0.40649	3.06E-11	4.13E-09	short-chain dehydrogenase/reductase family 16C member 6
LOC101900173	3539.486	4194.81	-0.24507	0.001817	0.028902	transport and Golgi organization protein 1, transcript variant X1
LOC101900188	1346.469	1662.858	-0.30448	2.88E-05	0.000949	uncharacterized protein DDB_G0284459
LOC101900194	301.8417	420.9698	-0.47992	0.000194	0.004745	ras guanine nucleotide exchange factor Y
LOC101900224	3149.867	3911.49	-0.31243	7.20E-08	4.75E-06	uncharacterized LOC101900224, transcript variant X5
LOC101900234	259.4048	359.5452	-0.47097	0.000398	0.008404	kinesin-like protein Nod
LOC101900253	576.8809	417.2949	0.46721	0.000131	0.003415	UNC93-like protein, transcript variant X2
LOC101900255	16111.53	18933.2	-0.23282	5.95E-07	3.24E-05	alkaline phosphatase 4
LOC101900267	106.9043	51.53791	1.0526	0.000106	0.002891	coiled-coil domain-containing protein 170
LOC101900275	3280.975	3765.777	-0.19882	0.000616	0.011819	catenin delta-2, transcript variant X2
LOC101900276	2074.327	2470.78	-0.25232	7.92E-05	0.002253	uncharacterized LOC101900276, transcript variant X2
LOC101900280	33.25383	142.2514	-2.0969	1.37E-15	3.39E-13	adult cuticle protein 1
LOC101900303	2797.375	3878.705	-0.4715	3.01E-16	8.02E-14	protein hairy
LOC101900306	1144.411	1552.536	-0.44002	6.30E-09	5.29E-07	potassium voltage-gated channel protein Shab, transcript variant X5
LOC101900326	611.1755	819.4658	-0.4231	7.01E-06	0.000274	sperm-specific antigen 2
LOC101900357	2270.059	1940.945	0.22597	0.000541	0.010721	acetyl-CoA acetyltransferase, cytosolic
LOC101900396	3829.17	3321.715	0.2051	0.000476	0.009624	cell cycle control protein 50A
LOC101900422	110.9448	170.7764	-0.62227	0.001917	0.030041	serine/threonine-protein phosphatase 2B catalytic subunit 1, transcript variant X3
LOC101900434	652.1471	838.6451	-0.36286	0.001393	0.023248	protein bride of sevenless, transcript variant X4
LOC101900435	25.42343	72.83927	-1.5186	1.01E-05	0.000376	uncharacterized LOC101900435
LOC101900445	2583.547	3352.981	-0.37609	3.67E-06	0.000157	lipopolysaccharide-induced tumor necrosis factor-alpha factor homolog, transcript variant X1
LOC101900451	2423.486	1663.41	0.54294	1.85E-16	5.06E-14	uncharacterized LOC101900451
LOC101900470	2646.333	3021.036	-0.19105	0.001637	0.026575	cold shock domain-containing protein E1
LOC101900481	654.9285	489.1082	0.42118	5.51E-05	0.001667	basement membrane-specific heparan sulfate proteoglycan core

						protein
LOC101900499	1325.513	1570.783	-0.24493	0.000677	0.012744	adapter molecule Crk
LOC101900502	2031.893	1528.26	0.41093	2.19E-09	1.99E-07	uncharacterized LOC101900502
LOC101900516	874.1559	670.517	0.38262	5.86E-05	0.001756	DNA N6-methyl adenine demethylase, transcript variant X2
LOC101900526	15.57458	71.31995	-2.1951	2.25E-09	2.03E-07	adult cuticle protein 1
LOC101900553	8297.192	9531.052	-0.20001	0.000207	0.005025	anoctamin-5
LOC101900559	775.3427	606.5639	0.35417	0.000199	0.004855	reticulon-4
LOC101900561	145.7628	305.211	-1.0662	7.14E-11	8.71E-09	alpha-amylase A
LOC101900572	655.8054	501.3491	0.38745	0.000432	0.00889	tubulin-specific chaperone E
LOC101900598	694.4782	492.0992	0.49698	1.32E-06	6.51E-05	phospholipase D3, transcript variant X2
LOC101900655	159.6432	103.8708	0.62006	0.001984	0.030782	UPF0047 protein YjbQ
LOC101900662	810.1132	996.1145	-0.29819	0.000888	0.016031	protein goliath, transcript variant X3
LOC101900675	511.0753	371.9691	0.45835	0.000106	0.00289	low-density lipoprotein receptor-related protein 1B
LOC101900709	6939.037	6142.901	0.17582	0.000676	0.012744	synaptobrevin, transcript variant X5
LOC101900721	119583.6	131651.9	-0.13871	0.001387	0.023218	uncharacterized LOC101900721, transcript variant X2
LOC101900764	727.716	1045.609	-0.5229	0.000132	0.003447	mucin-5AC, transcript variant X1
LOC101900767	254.0769	411.8582	-0.69688	1.08E-07	6.84E-06	uncharacterized LOC101900767
LOC101900769	368.9292	471.6177	-0.35427	0.003452	0.047026	protein stoned-B, transcript variant X2
LOC101900775	1808.93	2147.343	-0.24742	0.000214	0.005138	endothelin-converting enzyme 1, transcript variant X5
LOC101900822	8492.178	6419.377	0.4037	5.45E-16	1.39E-13	glycine-rich cell wall structural protein 1.8, transcript variant X2
LOC101900824	1127.497	1331.202	-0.23961	0.002014	0.031059	uncharacterized LOC101900824
LOC101900828	3396.606	4013.439	-0.24075	2.29E-05	0.000777	AMP deaminase 2, transcript variant X4
LOC101900841	1065.267	895.2045	0.25093	0.001925	0.030101	integrator complex subunit 1
LOC101900844	14.68825	82.6892	-2.493	3.95E-09	3.40E-07	collagen alpha-4(IV) chain
LOC101900870	300.1627	185.0955	0.69747	0.000402	0.008463	F-BAR and double SH3 domains protein 2
LOC101900871	0.332743	8.614898	-4.6944	0.003297	0.045698	uncharacterized LOC101900871
LOC101900875	616.1615	854.797	-0.47227	7.67E-07	4.00E-05	odorant receptor 42a-like
LOC101900899	2166.23	2552.383	-0.23666	0.000204	0.004963	protein rolling stone, transcript variant X2

LOC101900922	1.318609	18.41247	-3.8036	0.002248	0.033888	perlucin-like protein
LOC101900926	717.9644	542.5244	0.40422	3.19E-05	0.001037	O-acyltransferase like protein
LOC101900927	15.884	57.56093	-1.8575	7.85E-05	0.002243	glutamate-gated chloride channel subunit beta
LOC101900934	21250.26	13208.4	0.68603	1.51E-49	2.21E-46	alcohol dehydrogenase
LOC101900954	17299.35	14797.08	0.22541	1.56E-06	7.51E-05	polyadenylate-binding protein
LOC101900959	11.17501	34.26331	-1.6164	0.001609	0.026216	neuropeptide-like 1, transcript variant X1
LOC101900960	28.08627	266.3384	-3.2453	5.09E-17	1.57E-14	mediator of RNA polymerase II transcription subunit 15
LOC101900973	3881.533	2876.526	0.4323	4.10E-14	7.99E-12	NACHT and WD repeat domain-containing protein 2, transcript variant X2
LOC101900976	5260.639	5999.92	-0.18971	0.002783	0.039966	serine/threonine-protein phosphatase PP2A
LOC101901018	146.5654	408.6032	-1.4792	2.55E-05	0.000853	actin-5, muscle-specific, transcript variant X1
LOC101901022	337.488	252.9599	0.41593	0.003175	0.044329	transcription elongation factor SPT4
LOC101901044	2937.262	3728.087	-0.34396	3.91E-09	3.38E-07	microsomal glutathione S-transferase 1
LOC101901048	407.573	240.7861	0.75931	9.73E-09	8.10E-07	uncharacterized LOC101901048
LOC101901057	51.68033	23.0632	1.164	0.00266	0.038637	tyrosine-protein phosphatase Lar
LOC101901063	296.3236	215.7323	0.45793	0.001293	0.021862	BTB/POZ domain-containing protein kctd15-like, transcript variant X3
LOC101901072	371.8645	275.957	0.43033	0.001071	0.018659	integrator complex subunit 7
LOC101901107	1038.253	1248.617	-0.26617	0.000644	0.012272	REST corepressor, transcript variant X1
LOC101901110	261.1362	407.3038	-0.6413	1.42E-05	0.000506	H/ACA ribonucleoprotein complex subunit 4
LOC101901112	538.1219	418.205	0.36372	0.001306	0.022041	lipase member H-B
LOC101901113	759.3846	1100.599	-0.53539	6.75E-10	6.85E-08	inositol-trisphosphate 3-kinase homolog
LOC101901138	5.789579	67.42644	-3.5418	8.23E-15	1.76E-12	hypothetical protein
LOC101901144	1169.267	1807.804	-0.62863	1.31E-17	4.35E-15	protein pigeon, transcript variant X1
LOC101901158	3846.391	5043.17	-0.39083	1.12E-09	1.09E-07	eIF-2-alpha kinase activator GCN1
LOC101901167	397.4669	502.644	-0.3387	0.003342	0.046217	neurocalcin homolog
LOC101901173	99.662	325.0318	-1.7055	2.13E-16	5.76E-14	uncharacterized LOC101901173, transcript variant X2
LOC101901176	571.9856	769.3507	-0.42766	7.66E-05	0.0022	protein turtle

LOC101901187	3409.312	4018.073	-0.23702	3.39E-05	0.001094	chromodomain-helicase-DNA-binding protein Mi-2 homolog, transcript variant X2
LOC101901242	3379.126	2989.134	0.17692	0.00338	0.046482	E3 ubiquitin-protein ligase MARCH6
LOC101901250	667.1803	537.7559	0.31113	0.001723	0.027723	S-formylglutathione hydrolase
LOC101901263	1395.115	1733.511	-0.31331	1.37E-05	0.000491	putative epidermal cell surface receptor, transcript variant X1
LOC101901293	2137.331	1879.317	0.1856	0.003524	0.047718	unc-112-related protein, transcript variant X4
LOC101901302	1997.373	2462.376	-0.30195	3.28E-06	0.000142	arginine/serine-rich coiled-coil protein 2, transcript variant X1
LOC101901316	2998.551	2634.469	0.18675	0.002518	0.037009	ATP-binding cassette sub-family D member 3
LOC101901338	1076.654	892.7505	0.27023	0.000998	0.017671	ATP-binding cassette sub-family F member 3
LOC101901341	3489.981	4274.081	-0.2924	1.14E-07	7.14E-06	uncharacterized LOC101901341
LOC101901342	813.3058	578.5069	0.49146	4.20E-07	2.33E-05	ras-related protein Rab-21
LOC101901360	32.17803	90.34363	-1.4893	6.51E-07	3.51E-05	PAX-interacting protein 1
LOC101901400	1020.549	854.6755	0.2559	0.003082	0.043331	tudor domain-containing protein 3
LOC101901403	1430.114	1655.751	-0.21136	0.002633	0.038315	uncharacterized LOC101901403
LOC101901418	3859.336	3353.446	0.20271	0.000386	0.008212	low density lipoprotein receptor adapter protein 1-A
LOC101901420	10792.24	12029.75	-0.15661	0.00124	0.021056	Invariant surface glycoprotein
LOC101901439	761.3352	1001.183	-0.3951	5.28E-06	0.000216	uncharacterized LOC101901439, transcript variant X1
LOC101901453	2373.077	2019.965	0.23243	0.00033	0.007239	kinesin-like protein KIF19
LOC101901457	1093.486	914.2897	0.25821	0.001939	0.030241	uncharacterized LOC101901457
LOC101901462	317.3047	476.0152	-0.58514	2.26E-06	0.000103	serine-rich adhesin for platelets-like
LOC101901470	542.4057	366.127	0.56703	6.76E-07	3.59E-05	G protein-activated inward rectifier potassium channel 3, transcript variant X2
LOC101901505	2140.072	1749.113	0.29104	0.000323	0.007097	CAD protein
LOC101901517	821.0115	1074.741	-0.38852	8.12E-06	0.000314	endothelin-converting enzyme 1
LOC101901523	1670.622	1433.144	0.2212	0.002351	0.035049	threonine aspartase 1
LOC101901539	132.2268	234.2075	-0.82477	1.98E-06	9.29E-05	glutamate--cysteine ligase
LOC101901549	1408.858	1126.458	0.32273	0.00015	0.003827	serine/threonine-protein kinase MARK1
LOC101901562	2345.935	2726.004	-0.21663	0.000513	0.010264	proteasome subunit alpha type-6

LOC101901569	570.3308	895.8534	-0.65146	4.35E-12	6.58E-10	uncharacterized LOC101901569
LOC101901575	759.7834	1030.615	-0.43985	1.01E-06	5.10E-05	serine/threonine-protein kinase ATM
LOC101901585	111.2681	175.7605	-0.65957	0.000458	0.009371	uncharacterized LOC101901585
LOC101901589	623.9609	469.2926	0.41097	0.000114	0.003081	uncharacterized LOC101901589
LOC101901606	977.3035	1222.282	-0.3227	6.40E-05	0.001882	histone deacetylase Rpd3
LOC101901608	9832.63	8411.731	0.22517	2.48E-06	0.000112	pantothenate kinase 3, transcript variant X2
LOC101901624	43.17685	17.42486	1.3091	0.002397	0.03564	G2/mitotic-specific cyclin-B3, transcript variant X3
LOC101901627	4736.746	5354.64	-0.17689	0.002153	0.032761	serine/arginine repetitive matrix protein 2, transcript variant X2
LOC101901632	2526.447	2903.35	-0.20061	0.000656	0.012464	RING-variant domain protein, transcript variant X2
LOC101901643	1176.596	812.8672	0.53353	1.46E-10	1.65E-08	probable cytochrome P450 4ac1, transcript variant X1
LOC101901661	748.7793	590.0855	0.34362	0.000404	0.008489	activator of 90 kDa heat shock protein ATPase homolog 1
LOC101901667	1659.809	1226.373	0.43662	2.32E-09	2.08E-07	glucose dehydrogenase [FAD, quinone]
LOC101901668	1369.773	1174.578	0.22179	0.003513	0.047673	hypothetical protein, transcript variant X1
LOC101901673	999.9343	701.6022	0.51118	5.11E-09	4.33E-07	cartilage oligomeric matrix protein, transcript variant X2
LOC101901686	1362.348	1158.424	0.23393	0.001915	0.030023	band 4.1-like protein 4B, transcript variant X3
LOC101901691	501.3763	671.6756	-0.42187	5.90E-05	0.001759	chromatin assembly factor 1 subunit A-B
LOC101901709	1611.532	2181.78	-0.43707	0.000295	0.006631	methionine-R-sulfoxide reductase B1, transcript variant X4
LOC101901734	14737.75	17223.49	-0.22486	2.37E-06	0.000108	potassium voltage-gated channel subfamily KQT member 1, transcript variant X1
LOC101901744	1815.838	2094.284	-0.20582	0.00248	0.036577	sodium-dependent nutrient amino acid transporter 1
LOC101901772	364.5035	272.6088	0.4191	0.001711	0.027562	protein takeout
LOC101901795	1105.98	706.1175	0.64735	4.26E-14	8.22E-12	ammonium transporter Rh type A
LOC101901809	804.8741	583.8403	0.46319	0.000987	0.017548	transmembrane protein 205, transcript variant X2
LOC101901821	8.643741	44.75769	-2.3724	6.60E-07	3.53E-05	adult cuticle protein 1
LOC101901826	2838.516	3518.012	-0.30962	5.92E-05	0.00176	uncharacterized LOC101901826
LOC105261418	62.00103	29.7601	1.0589	0.001829	0.029044	tRNA pseudouridine synthase-like 1
LOC105261428	164.3621	239.7853	-0.54487	0.000835	0.015292	WD repeat domain-containing protein 83
LOC105261445	427.9183	329.8086	0.37571	0.001595	0.026017	cyclic AMP-responsive element-binding protein 5

LOC105261482	55.25259	99.46366	-0.84813	0.001884	0.029744	agrin-like
LOC105261536	295.2747	398.2965	-0.43178	0.003431	0.046847	CD109 antigen
LOC105261546	25.85593	75.7853	-1.5514	2.61E-06	0.000117	uncharacterized LOC105261546
LOC105261547	80.88815	136.8943	-0.75906	0.000602	0.011627	uncharacterized LOC105261547
LOC105261579	45.25202	86.68801	-0.93785	0.001548	0.025388	uncharacterized LOC105261579
LOC105261580	603.9368	477.892	0.33771	0.001247	0.021158	pickpocket protein 28-like
LOC105261589	591.0594	457.4965	0.36954	0.000465	0.009467	uncharacterized LOC105261589
LOC105261619	82.83518	179.9227	-1.1191	7.71E-08	5.07E-06	E3 ubiquitin-protein ligase XBAT32
LOC105261620	4.503006	113.9514	-4.6614	3.73E-13	6.48E-11	defensin-2-like
LOC105261724	65.19551	125.319	-0.94276	5.87E-05	0.001756	uncharacterized LOC105261724
LOC105261744	2958.209	2492.44	0.24717	0.000341	0.007421	hexokinase type 2
LOC105261789	1526.617	1305.316	0.22594	0.003175	0.044329	probable cytochrome P450 12c1, mitochondrial
LOC105261794	81.23326	136.2851	-0.74649	0.001	0.017693	uncharacterized LOC105261794
LOC105261803	76924.15	87280.56	-0.18222	2.51E-05	0.000844	uncharacterized LOC105261803
LOC105261829	7959.936	7082.807	0.16844	0.002628	0.038274	cytochrome P450 6A1-like
LOC105261850	203.2178	137.6731	0.56178	0.002349	0.035049	uncharacterized LOC105261850
LOC105261894	109.8447	60.70426	0.8556	0.000592	0.011466	uncharacterized LOC105261894
LOC105261919	272.1986	190.911	0.51176	0.003047	0.042875	uncharacterized LOC105261919
LOC105261927	287.3002	159.4829	0.84916	5.60E-08	3.76E-06	farnesyl pyrophosphate synthase
LOC105261934	337.2772	522.8967	-0.63259	1.16E-07	7.26E-06	transposable element Hobo transposase, transcript variant X1
LOC105261990	368.0309	472.0307	-0.35905	0.002059	0.031632	protein diaphanous
LOC105262125	2415.372	2915.779	-0.27164	1.03E-05	0.000381	uncharacterized LOC105262125, transcript variant X2
LOC105262193	83.47276	38.4543	1.1182	0.000155	0.003933	uncharacterized LOC105262193
LOC105262206	105.1301	173.8017	-0.72527	0.00016	0.004048	uncharacterized LOC105262206
LOC105262250	54.0871	115.1627	-1.0903	4.95E-05	0.001527	centromere/kinetochore protein zw10
LOC105262287	1.885461	24.19194	-3.6815	3.46E-06	0.000149	serine protease snake
LOC105262305	1968.982	2742.001	-0.47778	1.71E-07	1.04E-05	uncharacterized LOC105262305, transcript variant X2
LOC105262322	12.4998	71.31902	-2.5124	1.80E-10	1.96E-08	uncharacterized LOC105262322, transcript variant X2

LOC105262361	12457.51	11032.65	0.17524	0.000881	0.01594	nuclear protein 1
LOC105262366	405.6756	282.2546	0.52333	5.13E-05	0.001569	uncharacterized LOC105262366, transcript variant X1
LOC105262372	1483.627	1869.501	-0.33352	2.23E-06	0.000102	uncharacterized LOC105262372
LOC105262393	73.20077	33.13025	1.1437	0.000299	0.006706	uncharacterized LOC105262393
LOC105262402	2689.042	3163.343	-0.23436	0.000115	0.003082	uncharacterized LOC105262402
LOC105262418	2.262603	15.3832	-2.7653	0.001773	0.028338	ficolin-2
LOC105262471	72.22702	141.1448	-0.96657	1.73E-05	0.000599	heat shock protein 70-like
LOC105262478	1549.45	2410.065	-0.63732	1.55E-10	1.72E-08	nucleic-acid-binding protein from mobile element jockey
LOC105262486	876.6942	715.4496	0.29322	0.00151	0.024899	uncharacterized LOC105262486, transcript variant X1
LOC105262529	103.667	220.3678	-1.088	3.57E-09	3.13E-07	spidroin-2
LOC105262572	795.547	984.1137	-0.30688	0.000395	0.008377	one cut domain family member 2
LOC109611600	849.3051	1023.163	-0.26868	0.002308	0.034586	toll-interacting protein-like, transcript variant X2
LOC109611607	0.332743	9.032829	-4.7627	0.002861	0.040746	adult cuticle protein 1-like
LOC109611626	112	185.2656	-0.7261	0.000532	0.010605	glycine-rich protein 5-like
LOC109611666	40.05034	76.62547	-0.93601	0.001953	0.030353	uncharacterized LOC109611666
LOC109611756	262.4402	362.2013	-0.4648	0.000554	0.010879	uncharacterized LOC109611756
LOC109611794	1752.73	2187.641	-0.31977	1.36E-06	6.63E-05	uncharacterized LOC109611794
LOC109611815	393147.9	448675.4	-0.1906	1.48E-05	0.000526	uncharacterized LOC109611815
LOC109611821	362.6407	518.1503	-0.51483	6.75E-06	0.000265	glutamate receptor 1-like
LOC109611832	198.9567	275.1476	-0.46775	0.001942	0.030242	uncharacterized LOC109611832
LOC109611842	150.1109	79.05519	0.9251	1.97E-05	0.000675	melanization protease 1-like
LOC109611918	19.29492	1.547894	3.6398	4.99E-05	0.001535	membrane alanyl aminopeptidase-like
LOC109611919	94.84365	40.47894	1.2284	0.000222	0.005315	membrane alanyl aminopeptidase-like
LOC109611981	559.9794	710.9173	-0.34431	0.00058	0.011286	uncharacterized LOC109611981, transcript variant X2
LOC109612051	977.2427	1221.244	-0.32156	5.89E-05	0.001757	mucin-5AC-like
LOC109612094	67.64782	21.7363	1.6379	6.39E-06	0.000252	uncharacterized LOC109612094
LOC109612338	105.6793	57.50944	0.87782	0.000351	0.007597	acid-sensing ion channel 4
LOC109612354	9.93284	0.394443	4.6543	0.001194	0.020411	uncharacterized LOC109612354

LOC109612423	581.959	765.1413	-0.39481	4.56E-05	0.001426	uncharacterized LOC109612423
LOC109612700	5821.091	6958.739	-0.25754	9.90E-05	0.002726	cytochrome P450 6d1
LOC109612832	26.87018	989.7458	-5.203	1.90E-169	1.66E-165	uncharacterized LOC109612832
LOC109612900	1569.495	871.7907	0.84825	2.57E-27	1.29E-24	tryptophan--tRNA ligase, cytoplasmic-like
LOC109612926	52.58105	21.12603	1.3155	0.000617	0.011827	uncharacterized LOC109612926
LOC109612950	123.5496	308.7409	-1.3213	5.10E-16	1.32E-13	uncharacterized LOC109612950, transcript variant X2
LOC109612951	86.68302	138.4408	-0.67545	0.002135	0.032577	uncharacterized LOC109612951
LOC109613154	372.8529	503.1521	-0.43239	0.000204	0.004967	uncharacterized LOC109613154
LOC109613281	0.95383	35.47464	-5.2169	3.03E-11	4.12E-09	lectin subunit alpha-like
LOC109613374	24550.61	29172.2	-0.24884	5.13E-08	3.49E-06	general odorant-binding protein lush
LOC109613495	17.67699	2.374271	2.8963	0.000589	0.011416	uncharacterized LOC109613495
LOC109613504	35.38325	67.84804	-0.93924	0.002871	0.040864	uncharacterized LOC109613504
LOC109613556	8785.074	10898.15	-0.31096	4.44E-10	4.58E-08	uncharacterized LOC109613556
LOC109613800	868.0978	1334.834	-0.62073	1.01E-14	2.12E-12	uncharacterized LOC109613800, transcript variant X1
LOC109613803	567.6006	717.218	-0.33754	0.000847	0.015456	uncharacterized LOC109613803
LOC109613847	2859.599	2246.841	0.34792	1.85E-08	1.41E-06	uncharacterized LOC109613847
LOC109613902	371.054	481.0737	-0.37463	0.002545	0.037272	uncharacterized LOC109613902
LOC109613965	8.748557	28.84389	-1.7211	0.002009	0.031059	uncharacterized LOC109613965
LOC109614058	29.3138	242.9246	-3.0509	6.89E-40	7.56E-37	histidine-rich glycoprotein-like
LOC109614140	140.8531	218.1762	-0.6313	0.001368	0.022927	uncharacterized LOC109614140
LOC109614150	79.08644	125.7936	-0.66956	0.003375	0.046443	uncharacterized LOC109614150
LOC109614207	1742.257	2138.56	-0.29568	1.37E-05	0.000491	uncharacterized LOC109614207
LOC109614215	777.3083	1162.901	-0.58117	1.34E-11	1.97E-09	uncharacterized LOC109614215
LOC109614224	16.06247	56.10934	-1.8045	5.90E-06	0.000236	uncharacterized LOC109614224
LOC109614484	2006.882	2522.653	-0.32999	6.23E-05	0.00184	uncharacterized LOC109614484
Novel00012	248.7905	364.4047	-0.55061	3.94E-05	0.001254	-
Novel00030	21.152	2.487963	3.0878	0.000106	0.002891	-
Novel00034	519.4524	185.4044	1.4863	6.08E-29	3.95E-26	-

Novel00036	20.86138	52.66731	-1.3361	0.000526	0.010481	-
Novel00039	21.39229	1.948728	3.4565	8.96E-05	0.002511	-
Novel00076	40.34854	2.662633	3.9216	2.69E-10	2.85E-08	-
Novel00138	242.7111	154.2104	0.65434	0.00012	0.003205	-
Novel00168	66.87978	1.942337	5.1057	5.89E-20	2.25E-17	-
Novel00192	323.5015	445.3614	-0.4612	0.000264	0.006089	-
Novel00213	133.4557	83.5275	0.67604	0.003023	0.042641	-
Novel00265	13.13211	1.653974	2.9891	0.003309	0.045828	-
Novel00304	42.98942	78.36139	-0.86616	0.002801	0.040144	-
Novel00311	262.5777	365.0335	-0.47528	0.000474	0.009604	-
Novel00313	559.7983	751.0593	-0.42402	1.69E-05	0.000587	-
Novel00317	234.7658	347.8578	-0.56728	5.98E-05	0.001776	-
Novel00322	38.26378	3.602702	3.4088	0.002396	0.03564	-
Novel00340	36.19064	13.4512	1.4279	0.00234	0.034969	-
Novel00355	2.332857	14.97992	-2.6829	0.002786	0.039976	-
Novel00374	445.3859	312.7809	0.5099	4.19E-05	0.001332	-
Novel00376	27.61348	64.01824	-1.2131	0.000465	0.009467	-
Novel00483	134.0965	81.86017	0.71204	0.001187	0.020324	-
Novel00495	46.44248	13.7484	1.7562	8.46E-05	0.002387	-
Novel00516	132.9038	210.4561	-0.66314	0.000293	0.006614	-
Novel00526	47.55535	19.24194	1.3054	0.001639	0.026575	-
Novel00593	784.4836	1206.116	-0.62055	2.17E-11	3.00E-09	-
Novel00647	0	7.629727	#NAME?	0.002232	0.033728	-
Novel00728	4860.026	6153.067	-0.34034	1.55E-10	1.72E-08	-
Novel00734	0.348761	8.553919	-4.6163	0.002904	0.041223	-
Novel00783	251.6885	368.844	-0.55137	5.67E-05	0.001705	-
Novel00802	76.40256	41.18365	0.89155	0.003581	0.048256	-
Novel00831	755.9766	520.0472	0.5397	0.001108	0.019128	-

Novel00843	51.77366	21.82198	1.2464	0.001302	0.021992	-
Novel00844	79.00359	27.5493	1.5199	3.05E-06	0.000133	-
Novel00874	26.61022	7.012417	1.924	0.001651	0.026743	-
Novel00904	223.362	327.1278	-0.55047	7.62E-05	0.002193	-
Novel01019	76.48633	28.12933	1.4431	1.89E-05	0.000651	-
Novel01043	61.86808	127.6765	-1.0452	8.58E-06	0.000329	-
Novel01096	144.6822	211.9273	-0.55068	0.001887	0.029744	-
Novel01230	41.93808	16.42691	1.3522	0.00163	0.02648	-
Novel01289	264.7388	184.1303	0.52384	0.00103	0.018133	-
Novel01453	7.53704	25.63253	-1.7659	0.003091	0.043405	-
Novel01498	0.621087	17.04751	-4.7786	1.60E-05	0.000564	-
Novel01499	0	10.41366	#NAME?	0.000155	0.003923	-
Novel01500	1.945878	19.45532	-3.3217	7.92E-05	0.002253	-
Novel01539	2.639745	16.15133	-2.6132	0.002448	0.036162	-
Novel01561	214.7752	309.769	-0.52837	0.00028	0.006418	-
Novel01563	237.8335	158.994	0.58098	0.000534	0.010615	-
Novel01571	74.03237	20.46793	1.8548	9.58E-08	6.17E-06	-
Novel01635	97.80996	180.6938	-0.88549	5.79E-06	0.000233	-
Novel01661	552.7939	753.3256	-0.44653	6.00E-06	0.000239	-
Novel01681	271.0702	434.3221	-0.6801	1.33E-07	8.21E-06	-
Novel01696	11.62494	39.38919	-1.7606	0.000239	0.005661	-
Novel01705	21.85936	5.543819	1.9793	0.002174	0.033007	-
Novel01737	16.93621	51.74332	-1.6113	7.02E-05	0.002032	-
Novel01754	45.25592	95.91468	-1.0836	0.000148	0.003797	-
Novel01785	146.3361	72.67343	1.0098	7.14E-06	0.000278	-
Novel01788	356.6497	527.7776	-0.56542	2.15E-06	9.94E-05	-
Novel01790	369.044	270.6734	0.44724	0.002622	0.038218	-
Novel01802	1112.667	1342.242	-0.27062	0.001615	0.026281	-

Novel01806	93.45178	159.4748	-0.77103	0.000255	0.005933	-
Novel01820	161.4877	227.9857	-0.49752	0.00336	0.046311	-
Novel01828	2296.077	1239.199	0.88976	4.46E-37	4.61E-34	-
Novel01934	152.4128	99.94867	0.60873	0.002765	0.039768	-
Novel02036	193.6367	96.71683	1.0015	8.10E-07	4.19E-05	-
Novel02053	53.9152	7.667217	2.8139	2.16E-05	0.000737	-
Novel02100	204.9628	1.008659	7.6668	1.59E-71	4.65E-68	-
Novel02124	233.594	309.8167	-0.40741	0.003637	0.048834	-
Novel02131	31.25739	68.4382	-1.1306	0.000608	0.011708	-
Novel02136	431.1414	298.3205	0.5313	3.26E-05	0.00106	-
Novel02139	133.6182	62.74572	1.0905	1.70E-06	8.12E-05	-
Novel02148	7.841402	0	Inf	0.001578	0.025758	-
Novel02161	117.9042	68.04066	0.79315	0.001308	0.022055	-
Novel02181	38.07723	9.957022	1.9351	0.000129	0.003382	-
Novel02218	48.31193	13.48869	1.8406	0.000138	0.003596	-
Novel02228	544.9782	311.1795	0.80845	1.53E-11	2.20E-09	-
Novel02295	1290.305	1641.224	-0.34706	1.24E-06	6.17E-05	-
Novel02309	13.65456	0.940069	3.8605	0.000565	0.011068	-
Novel02333	67.25604	12.68824	2.4062	1.95E-09	1.80E-07	-
Novel02334	17.88889	2.411761	2.8909	0.000489	0.009831	-
Novel02335	85.21242	9.857332	3.1118	1.56E-15	3.71E-13	-
Novel02336	67.29654	11.30741	2.5733	1.16E-10	1.33E-08	-
Novel02353	5.565307	22.57977	-2.0205	0.0024	0.035667	-
Novel02355	96.5673	177.4724	-0.87799	1.19E-05	0.000431	-
Novel02384	124.8488	54.7693	1.1887	2.25E-06	0.000103	-
Novel02388	23.7684	58.71165	-1.3046	0.000384	0.008192	-
Novel02415	118.8622	34.27092	1.7942	8.11E-11	9.58E-09	-
Novel02421	1933.689	1410.745	0.4549	8.02E-11	9.58E-09	-

Novel02427	10.6006	0.288363	5.2001	0.00316	0.044183	-
Novel02438	195.2382	372.9843	-0.93388	5.55E-11	7.06E-09	-
Novel02459	67.59219	30.25582	1.1596	0.000571	0.011164	-
Novel02527	56.07234	7.323048	2.9368	2.57E-10	2.73E-08	-
Novel02528	40.90806	14.01637	1.5453	0.000807	0.014843	-
Novel02615	3461.947	2832.988	0.28926	1.64E-05	0.000575	-
Novel02616	473.5661	348.3459	0.44305	0.000244	0.005758	-
Novel02626	36.00459	5.732492	2.6509	2.11E-06	9.81E-05	-
Novel02631	511.1878	771.0824	-0.59303	3.02E-09	2.66E-07	-
Novel02633	26.42303	4.581482	2.5279	0.000181	0.004483	-
Novel02656	1.046283	11.43942	-3.4507	0.003297	0.045698	-
Novel02664	48587.9	22269.89	1.1255	3.89E-53	6.21E-50	-
Novel02671	17.75204	54.18232	-1.6098	0.000369	0.007952	-
Novel02687	6917.272	7884.774	-0.18887	0.000118	0.00316	-
Novel02700	59.10272	24.0121	1.2995	0.000587	0.011402	-
Novel02710	147.5341	14.74183	3.3231	9.14E-28	5.01E-25	-
Novel02741	33.69872	11.89814	1.502	0.002753	0.039626	-
Novel02743	124.9477	65.59789	0.9296	0.000373	0.008005	-
Novel02793	39.69037	1.008659	5.2983	1.03E-12	1.69E-10	-
Novel02822	155.2996	238.2891	-0.61766	0.000211	0.005086	-
Novel02847	29.63329	8.545088	1.7941	0.000964	0.017216	-
Novel02857	24.43779	4.161111	2.5541	0.000584	0.011351	-
Novel02879	66.98006	33.54753	0.99753	0.003187	0.044456	-
Novel02898	274.5457	190.3293	0.52855	0.001272	0.021548	-
Novel02942	274.3077	185.111	0.5674	0.000261	0.00604	-
Novel02945	59.62173	14.53728	2.0361	0.000552	0.010868	-
Novel02958	25.71315	2.1996	3.5472	3.20E-06	0.000139	-
Novel02976	245.2143	175.8404	0.47978	0.003448	0.047009	-

Novel03041	158.3093	245.272	-0.63164	0.000145	0.003738	-
Novel03063	584.1543	442.649	0.40019	0.000295	0.006631	-
Novel03066	378.0732	481.4297	-0.34866	0.003098	0.043456	-
Novel03073	101.9308	47.41459	1.1042	3.64E-05	0.001166	-
SPN13	19.76314	53.71	-1.4424	0.002246	0.033888	serine protease inhibitor 3/4-like
TCTP	10520.14	9304.351	0.17718	0.002119	0.032387	translationally-controlled tumor protein homolog
THY	1242.084	1592.304	-0.35835	6.63E-06	0.000261	thymosin, transcript variant X1
attacin	155.0775	247.6301	-0.6752	2.54E-05	0.00085	attacin-A-like, transcript variant X2
attacin_1	7.527203	59.29872	-2.9778	8.04E-11	9.58E-09	sarcotoxin II-1-like
defensin-1	2074.456	5211.894	-1.3291	1.89E-115	1.10E-111	phormicin-like
diptericin	95.45808	167.3949	-0.81032	5.10E-05	0.001563	diptericin-D-like
yp3	2695.437	1551.561	0.7968	3.98E-34	3.68E-31	vitellogenin-1-like
