# Supplementary material for: Short-term particulate matter contamination severely compromises insect antennal olfactory perception
Source: Nat Commun. 2023 Jul 11;14:4112. doi: 10.1038/s41467-023-39469-3 (PMC10336072; doi:10.1038/s41467-023-39469-3)
Supplement: Supplementary file 8 — Supplementary Data 5 [file 41467_2023_39469_MOESM8_ESM.pdf]

Supplementary Data 5. Details of differential expressed genes (DEGs) between the bodies of uncontaminated and contaminated female houseflies in spring. Readcount is the average read count of each gen, log2FoldChange is calculated by formular log2(readcount_CFB/readcount_UFB), pval is the *p* value, padj is the fdr corrected *p* value using Benjamini/Hochberg method. All *p*-values are based on two-sided tests. CFB: contaminated female bodies, UFB: uncontaminated female bodies.

**Gene_id**	**readcount_CFB**	**readcount_UFB**	**log2FoldChange**	**pval**	**padj**	**gene_name**	**description**
Novel00281	94.5838	201.9008	-1.094	6.58E-06	0.002097	-	-
Novel01165	161.0896	68.27566	1.2384	1.09E-05	0.003105	-	-
Novel01285	17.52002	1.308442	3.7431	0.000119	0.019132	-	-
Novel01368	12.38986	50.41142	-2.0246	1.27E-05	0.003509	-	-
Novel01713	9.783462	36.08158	-1.8828	0.000388	0.045356	-	-
Novel01738	3.057852	38.92454	-3.6701	9.50E-06	0.002805	-	-
gene-CYP6G4	888.0758	1506.431	-0.76238	7.23E-07	0.000419	CYP6G4	cytochrome P450 6g1-like
gene-LOC101887397	116.4396	232.6396	-0.99852	7.91E-06	0.002471	LOC101887397	H/ACA ribonucleoprotein complex subunit 1
gene-LOC101887422	46.36106	109.9672	-1.2461	3.78E-05	0.008407	LOC101887422	putative glycine-rich cell wall structural protein 1
gene-LOC101887577	5070.624	3300.481	0.61949	1.15E-05	0.003211	LOC101887577	D-3-phosphoglycerate dehydrogenase
gene-LOC101887634	414.8381	257.1498	0.68994	0.000173	0.024411	LOC101887634	saccharopine dehydrogenase-like oxidoreductase%2C transcript variant X2||saccharopine dehydrogenase-like oxidoreductase isoform X2
gene-LOC101887655	66.55833	138.4968	-1.0572	6.63E-05	0.012241	LOC101887655	probable cytochrome P450 49a1%2C transcript variant X1||probable cytochrome P450 49a1 isoform X1
gene-LOC101887683	8735.483	5469.996	0.67535	1.14E-06	0.000632	LOC101887683	5-aminolevulinate synthase%2C erythroid-specific%2C mitochondrial%2C transcript variant X2
gene-LOC101887687	3943.463	1818.359	1.1168	2.19E-06	0.001013	LOC101887687	amidophosphoribosyltransferase
gene-LOC101887760	4507.266	6846.809	-0.60318	8.18E-05	0.014299	LOC101887760	dihydropyrimidine dehydrogenase [NADP(+)]
gene-LOC101887984	536.3401	312.2747	0.78033	5.85E-05	0.011061	LOC101887984	tissue-type plasminogen activator
gene-LOC101888070	848.2084	1362.927	-0.68422	8.87E-06	0.002669	LOC101888070	putative RNA-binding protein 15%2C transcript variant X2
gene-LOC101888372	56.87907	19.69665	1.5299	0.000183	0.025468	LOC101888372	retinoid-inducible serine carboxypeptidase-like
gene-LOC101888515	16.52763	55.13709	-1.7381	0.000301	0.037621	LOC101888515	uncharacterized LOC101888515||uncharacterized

							protein LOC101888515
gene-LOC101888614	31292.73	17049.64	0.87609	1.31E-10	2.36E-07	LOC101888614	fatty acid synthase
gene-LOC101888695	1639.202	3527.107	-1.1055	1.79E-12	7.28E-09	LOC101888695	ATP-binding cassette sub-family G member 1
gene-LOC101888731	271.8097	438.2474	-0.68915	0.000128	0.019976	LOC101888731	peroxisomal carnitine O-octanoyltransferase||peroxisomal carnitine O-octanoyltransferase%2C transcript variant X2
gene-LOC101888820	719.7009	413.6009	0.79916	1.68E-06	0.000801	LOC101888820	peroxisomal (S)-2-hydroxy-acid oxidase GLO5
gene-LOC101888890	335.1305	200.0827	0.74413	0.00014	0.021093	LOC101888890	putative phosphatidate phosphatase
gene-LOC101889163	1223.193	1775.548	-0.53761	0.000237	0.031357	LOC101889163	uncharacterized protein LOC101889163||uncharacterized LOC101889163
gene-LOC101889496	32.8199	85.53687	-1.382	0.000166	0.023675	LOC101889496	UDP-glucuronosyltransferase 2C1-like
gene-LOC101889504	1073.498	1596.789	-0.57285	0.000279	0.035093	LOC101889504	dnaJ homolog subfamily C member 3
gene-LOC101889618	1409.758	2955.051	-1.0677	1.66E-06	0.000801	LOC101889618	uncharacterized LOC101889618||uncharacterized protein LOC101889618
gene-LOC101889924	95.83859	15.26263	2.6506	4.31E-07	0.00028	LOC101889924	probable cytochrome P450 304a1
gene-LOC101890105	300.5709	106.7398	1.4936	3.21E-06	0.00124	LOC101890105	cubilin homolog
gene-LOC101890109	2259.582	3271.098	-0.53372	0.000161	0.023412	LOC101890109	uncharacterized protein LOC101890109||hypothetical protein
gene-LOC101890125	165.9182	342.0939	-1.0439	0.00011	0.018071	LOC101890125	elongation of very long chain fatty acids protein 7
gene-LOC101890180	509.3907	287.2095	0.82667	3.64E-06	0.001371	LOC101890180	period circadian protein%2C transcript variant X1
gene-LOC101890413	472.8383	751.2005	-0.66785	4.77E-05	0.009988	LOC101890413	KH domain-containing%2C RNA-binding%2C signal transduction-associated protein 2 isoform X2||KH domain-containing%2C RNA-binding%2C signal transduction-associated protein 2%2C transcript variant X2
gene-LOC101890446	3654.239	1994.154	0.87379	2.27E-08	2.46E-05	LOC101890446	probable galactose-1-phosphate uridylyltransferase
gene-LOC101890653	7027.387	4409.124	0.6725	0.000218	0.029281	LOC101890653	cytosolic 10-formyltetrahydrofolate dehydrogenase
gene-LOC101890676	502.9248	304.1303	0.72565	4.92E-05	0.009988	LOC101890676	uncharacterized protein LOC101890676||3-oxoacyl-

							[acyl-carrier-protein] reductase FabG
gene-LOC101890698	209.8356	553.4398	-1.3992	2.01E-07	0.000156	LOC101890698	mitochondrial basic amino acids transporter%2C transcript variant X2||mitochondrial basic amino acids transporter isoform X2
gene-LOC101891486	9183.434	19479.48	-1.0848	6.09E-06	0.002019	LOC101891486	uncharacterized protein LOC101891486||uncharacterized LOC101891486
gene-LOC101891576	8449.505	5201.678	0.69989	5.85E-07	0.000352	LOC101891576	NADP-dependent malic enzyme isoform X2||NADP-dependent malic enzyme%2C transcript variant X2
gene-LOC101891733	6910.604	4423.095	0.64376	4.13E-06	0.001492	LOC101891733	facilitated trehalose transporter Tret1%2C transcript variant X3||facilitated trehalose transporter Tret1 isoform X2
gene-LOC101891747	164.7312	307.2664	-0.89938	6.37E-06	0.00207	LOC101891747	protein yellow%2C transcript variant X3||protein yellow
gene-LOC101891843	67.26228	27.54556	1.288	0.000414	0.046389	LOC101891843	BTB/POZ domain-containing protein At1g55760-like isoform X3||protein roadkill-like%2C transcript variant X3
gene-LOC101891927	31596.63	45091.68	-0.51309	0.0004	0.045444	LOC101891927	sarcoplasmic calcium-binding protein%2C beta chain
gene-LOC101891995	72.92126	165.8164	-1.1852	2.49E-06	0.001039	LOC101891995	uncharacterized protein LOC101891995||uncharacterized LOC101891995
gene-LOC101892043	161.9063	390.0008	-1.2683	6.72E-05	0.012271	LOC101892043	putative transporter svop-1
gene-LOC101892053	103.1772	198.2883	-0.94247	4.67E-05	0.009982	LOC101892053	uncharacterized LOC101892053||uncharacterized protein LOC101892053
gene-LOC101892229	230.5858	374.6155	-0.70011	0.000279	0.035093	LOC101892229	protein tramtrack%2C beta isoform isoform X2||protein tramtrack%2C beta isoform%2C transcript variant X4
gene-LOC101892245	164.1756	321.6453	-0.97023	3.33E-05	0.007817	LOC101892245	carcinine transporter
gene-LOC101892461	367.7036	815.1523	-1.1485	4.56E-06	0.001611	LOC101892461	probable 4-coumarate--CoA ligase 1
gene-LOC101892515	579.6805	888.7701	-0.61655	9.52E-05	0.016449	LOC101892515	retinol-binding protein pinta
gene-LOC101892627	345.5922	166.8666	1.0504	2.77E-06	0.001127	LOC101892627	uncharacterized protein

								LOC101892627||uncharacterized LOC101892627
gene-LOC101892670	24.66347	65.20878	-1.4027	0.000258	0.033289	LOC101892670	male accessory gland serine protease inhibitor
gene-LOC101892892	569.3049	279.4976	1.0264	7.16E-05	0.01278	LOC101892892	growth/differentiation factor 8
gene-LOC101892985	36.84748	8.785663	2.0683	0.000148	0.022108	LOC101892985	PERQ amino acid-rich with GYF domain-containing protein 2
gene-LOC101893034	107.9543	230.1302	-1.092	3.37E-05	0.007817	LOC101893034	uncharacterized LOC101893034
gene-LOC101893206	175.8406	67.4914	1.3815	1.91E-07	0.000155	LOC101893206	chymotrypsin-1
gene-LOC101893377	249.6138	423.7737	-0.7636	2.89E-05	0.00733	LOC101893377	uncharacterized LOC101893377%2C transcript variant X1
gene-LOC101893548	196.1166	384.8346	-0.97253	2.89E-07	0.000213	LOC101893548	membrane metallo-endopeptidase-like 1
gene-LOC101893820	267.1955	427.7314	-0.67881	0.000201	0.027422	LOC101893820	monocarboxylate transporter 9
gene-LOC101893852	44.72992	8.368217	2.4182	3.71E-06	0.001371	LOC101893852	sarcotoxin-2A-like
gene-LOC101893922	16868.47	11100.28	0.60373	1.04E-05	0.003021	LOC101893922	glutamine synthetase 1%2C mitochondrial
gene-LOC101893990	1091.077	666.1994	0.71173	5.44E-06	0.001843	LOC101893990	organic cation transporter protein
gene-LOC101894268	247.091	114.8806	1.1049	5.02E-07	0.000314	LOC101894268	uncharacterized LOC101894268||uncharacterized protein LOC101894268
gene-LOC101894344	144.506	68.93599	1.0678	4.63E-05	0.009982	LOC101894344	basic-leucine zipper transcription factor A-like
gene-LOC101894431	1491.105	2557.493	-0.77835	8.28E-08	8.20E-05	LOC101894431	clavesin-2%2C transcript variant X1||clavesin-2
gene-LOC101894491	44.34013	11.27691	1.9752	5.03E-05	0.010093	LOC101894491	uncharacterized LOC101894491||uncharacterized protein LOC101894491
gene-LOC101894606	1756.336	2959.766	-0.75291	0.000325	0.040347	LOC101894606	uncharacterized protein LOC101894606||uncharacterized LOC101894606
gene-LOC101894757	684.1927	1053.433	-0.62262	0.000174	0.024411	LOC101894757	uncharacterized LOC101894757||uncharacterized protein LOC101894757
gene-LOC101894765	321.0763	119.6877	1.4236	3.59E-07	0.000245	LOC101894765	uncharacterized protein LOC101894765||uncharacterized LOC101894765
gene-LOC101894880	357.8716	217.7146	0.717	0.000189	0.026026	LOC101894880	flocculation protein FLO11%2C transcript variant X5||flocculation protein FLO11 isoform X3

gene-LOC101895034	1715.849	838.418	1.0332	9.42E-12	2.19E-08	LOC101895034	uncharacterized LOC101895034||uncharacterized protein LOC101895034
gene-LOC101895236	121.7748	61.4652	0.98637	0.000463	0.049827	LOC101895236	centrosomal protein of 135 kDa%2C transcript variant X1||centrosomal protein of 135 kDa isoform X1
gene-LOC101895241	591.334	278.6307	1.0856	0.000161	0.023412	LOC101895241	metallothionein-2-like
gene-LOC101895246	518.7905	336.4551	0.62474	0.000396	0.045356	LOC101895246	carboxypeptidase B
gene-LOC101895341	405.4681	126.4909	1.6806	1.15E-14	1.86E-10	LOC101895341	uncharacterized protein LOC101895341||uncharacterized LOC101895341
gene-LOC101895520	132.4054	517.8402	-1.9675	1.17E-06	0.000632	LOC101895520	putative inorganic phosphate cotransporter
gene-LOC101895559	250.9537	461.0409	-0.87747	2.31E-06	0.001013	LOC101895559	uncharacterized protein LOC101895559||sulfur globule protein CV3-like
gene-LOC101895609	360.4567	212.5211	0.76222	6.89E-05	0.012442	LOC101895609	twinkle protein%2C mitochondrial
gene-LOC101895644	173.2924	578.1675	-1.7383	0.0001	0.016989	LOC101895644	putative inorganic phosphate cotransporter
gene-LOC101895696	100.8729	187.8813	-0.89728	0.000125	0.019724	LOC101895696	putative inorganic phosphate cotransporter
gene-LOC101895730	165.0276	59.67133	1.4676	1.65E-06	0.000801	LOC101895730	uncharacterized protein LOC101895730||uncharacterized LOC101895730
gene-LOC101895795	285.8168	759.1159	-1.4092	0.000342	0.041736	LOC101895795	putative fatty acyl-CoA reductase CG5065%2C transcript variant X4||putative fatty acyl-CoA reductase CG5065
gene-LOC101895906	265.3949	90.34753	1.5546	5.44E-12	1.77E-08	LOC101895906	uncharacterized protein LOC101895906||uncharacterized LOC101895906
gene-LOC101896011	480.8	740.3245	-0.62272	0.00015	0.022108	LOC101896011	uncharacterized LOC101896011||uncharacterized protein LOC101896011
gene-LOC101896308	12199.56	8185.446	0.5757	3.56E-05	0.008032	LOC101896308	uncharacterized protein LOC101896308||uncharacterized LOC101896308
gene-LOC101896380	898.8989	423.0501	1.0873	0.000389	0.045356	LOC101896380	phosphatidylserine synthase 1%2C transcript variant X3||phosphatidylserine synthase 1 isoform X3
gene-LOC101896469	3241.482	1577.025	1.0394	6.07E-09	7.04E-06	LOC101896469	probable cytochrome P450 313a4%2C transcript

								variant X2||probable cytochrome P450 313a4 isoform X2
gene-LOC101896484	12939.39	8250.152	0.64928	4.81E-06	0.001661	LOC101896484	uncharacterized protein LOC101896484||uncharacterized LOC101896484
gene-LOC101896601	2278.575	3471.289	-0.60734	2.03E-05	0.005323	LOC101896601	uncharacterized LOC101896601||uncharacterized protein LOC101896601
gene-LOC101896716	1.944202	28.00262	-3.8483	9.65E-05	0.016508	LOC101896716	ctenidin-1
gene-LOC101896891	3.097644	22.45925	-2.8581	0.000242	0.031478	LOC101896891	glycine-rich cell wall structural protein 1-like
gene-LOC101896932	741.9995	478.9135	0.63165	0.000103	0.017088	LOC101896932	putative metabolite transport protein YwtG||uncharacterized protein LOC101896932
gene-LOC101897154	1411.647	2974.991	-1.0755	1.87E-13	1.01E-09	LOC101897154	esterase B1
gene-LOC101897248	98.02331	41.91941	1.2255	0.000423	0.046443	LOC101897248	uncharacterized protein LOC101897248||uncharacterized LOC101897248
gene-LOC101897347	1247.043	2046.44	-0.71461	1.30E-06	0.000683	LOC101897347	dnaJ protein homolog 1%2C transcript variant X2||dnaJ protein homolog 1
gene-LOC101897428	2085.533	529.2644	1.9784	2.41E-06	0.001031	LOC101897428	phosphatidylserine decarboxylase proenzyme%2C mitochondrial%2C transcript variant X2
gene-LOC101897431	127.4745	51.19849	1.316	0.000423	0.046443	LOC101897431	aminoacylase-1B-like
gene-LOC101897683	888.9321	454.0062	0.96936	1.53E-07	0.000131	LOC101897683	sphingomyelin phosphodiesterase
gene-LOC101897689	442.9831	268.1593	0.72416	5.52E-05	0.010694	LOC101897689	open rectifier potassium channel protein 1
gene-LOC101897812	1939.303	1285.6	0.5931	6.28E-05	0.011719	LOC101897812	ribonucleoside-diphosphate reductase subunit M2
gene-LOC101897868	1889.344	2748.768	-0.5409	0.000203	0.027422	LOC101897868	uncharacterized LOC101897868%2C transcript variant X1||uncharacterized protein LOC101897868 isoform X1
gene-LOC101897942	3317.38	2078.684	0.67437	0.000395	0.045356	LOC101897942	serine proteases 1/2
gene-LOC101898023	303.8522	661.2807	-1.1219	1.18E-10	2.36E-07	LOC101898023	uncharacterized LOC101898023||uncharacterized protein LOC101898023
gene-LOC101898109	519.4219	218.9473	1.2463	0.000429	0.046768	LOC101898109	serine proteases 1/2-like

gene-LOC101898132	551.0826	1064.181	-0.9494	2.10E-09	2.85E-06	LOC101898132	fatty acyl-CoA reductase wat
gene-LOC101898204	724.9088	235.5296	1.6219	8.65E-06	0.002653	LOC101898204	cubilin homolog
gene-LOC101898346	598.0385	197.3647	1.5994	1.71E-09	2.52E-06	LOC101898346	organic cation transporter protein
gene-LOC101898458	1159.738	1675.283	-0.53061	0.000404	0.04553	LOC101898458	ethanolamine kinase
gene-LOC101898634	1796.318	1209.517	0.57061	0.000114	0.018519	LOC101898634	proton-coupled folate transporter
gene-LOC101898663	1277.818	2722.703	-1.0914	1.06E-13	8.64E-10	LOC101898663	carnitine O-palmitoyltransferase 1%2C liver isoform%2C transcript variant X2
gene-LOC101898751	1.02202	17.86498	-4.1276	3.24E-05	0.007817	LOC101898751	protein ALP1-like
gene-LOC101898778	152.9277	74.27331	1.0419	4.61E-05	0.009982	LOC101898778	uncharacterized LOC101898778||uncharacterized protein LOC101898778
gene-LOC101898892	192.1603	100.2868	0.93818	5.53E-05	0.010694	LOC101898892	lectin subunit alpha
gene-LOC101898919	67.31686	145.3857	-1.1108	2.66E-05	0.006858	LOC101898919	potassium channel subfamily K member 1
gene-LOC101898975	369.1322	757.0543	-1.0363	6.37E-10	1.04E-06	LOC101898975	probable multidrug resistance-associated protein lethal(2)03659
gene-LOC101899222	194.0314	80.00744	1.2781	0.000102	0.017088	LOC101899222	transmembrane protein 209
gene-LOC101899240	443.7254	804.9587	-0.85925	3.11E-06	0.001231	LOC101899240	carbohydrate sulfotransferase 13 isoform X1||carbohydrate sulfotransferase 13%2C transcript variant X1
gene-LOC101899489	2754.39	1917.439	0.52255	0.000241	0.031478	LOC101899489	integumentary mucin A.1-like%2C transcript variant X1||integumentary mucin A.1-like isoform X1
gene-LOC101899571	4543.084	2899.805	0.64772	1.83E-05	0.004861	LOC101899571	beta-galactosidase%2C transcript variant X1||beta-galactosidase isoform X1
gene-LOC101899651	73.1864	139.1999	-0.92751	0.000384	0.045356	LOC101899651	uncharacterized LOC101899651||uncharacterized protein LOC101899651
gene-LOC101899663	7.905819	68.85584	-3.1226	7.31E-12	1.98E-08	LOC101899663	keratin-associated protein 19-2-like
gene-LOC101899678	462.1176	296.2976	0.64121	0.000332	0.040828	LOC101899678	sodium-dependent nutrient amino acid transporter 1
gene-LOC101899803	27.46909	217.3163	-2.9839	1.29E-07	0.000116	LOC101899803	arylphorin subunit C223
gene-LOC101899837	1008.304	690.7176	0.54576	0.000385	0.045356	LOC101899837	maltase A3-like

gene-LOC101899915	743.0483	372.2908	0.99703	3.11E-09	3.89E-06	LOC101899915	glycine receptor subunit alpha-4
gene-LOC101900034	37.03726	92.88279	-1.3264	4.89E-05	0.009988	LOC101900034	prolyl 4-hydroxylase subunit alpha-1
gene-LOC101900049	578.7495	851.2709	-0.55668	0.000442	0.04793	LOC101900049	KH domain-containing%2C RNA-binding%2C signal transduction-associated protein 2 isoform X2||KH domain-containing%2C RNA-binding%2C signal transduction-associated protein 2%2C transcript variant X2
gene-LOC101900255	2696.546	1688.045	0.67576	3.46E-05	0.007927	LOC101900255	alkaline phosphatase 4
gene-LOC101900465	469.5578	224.0943	1.0672	8.58E-08	8.20E-05	LOC101900465	sialin-like
gene-LOC101900478	234.1248	132.8514	0.81746	0.00014	0.021093	LOC101900478	protein anon-37Cs
gene-LOC101900480	78.41038	197.0369	-1.3293	2.25E-06	0.001013	LOC101900480	endothelin-converting enzyme 2
gene-LOC101900680	151.706	74.81628	1.0199	7.39E-05	0.013051	LOC101900680	alcohol dehydrogenase-related 31 kDa protein
gene-LOC101900775	515.6328	812.9064	-0.65675	5.78E-05	0.011057	LOC101900775	endothelin-converting enzyme 1%2C transcript variant X5
gene-LOC101900974	86.60792	173.3921	-1.0015	3.35E-05	0.007817	LOC101900974	lipopolysaccharide-induced tumor necrosis factor-alpha factor homolog
gene-LOC101901193	546.9905	348.4616	0.65052	0.00037	0.04483	LOC101901193	uncharacterized protein LOC101901193||uncharacterized LOC101901193
gene-LOC101901284	19.53775	66.25375	-1.7617	0.000135	0.020661	LOC101901284	shematrin-like protein 2
gene-LOC101901292	561.1026	984.4459	-0.81105	1.58E-05	0.00428	LOC101901292	gonadotropin-releasing hormone receptor isoform X1||gonadotropin-releasing hormone receptor%2C transcript variant X3
gene-LOC101901423	832.019	561.8821	0.56635	0.000394	0.045356	LOC101901423	uncharacterized LOC101901423||uncharacterized protein LOC101901423
gene-LOC101901572	1200.199	770.1845	0.64	3.14E-05	0.007817	LOC101901572	uncharacterized threonine-rich GPI-anchored glycoprotein PJ4664.02
gene-LOC101901690	709.0702	1260.584	-0.83009	0.000418	0.046443	LOC101901690	retinol-binding protein pinta
gene-LOC101901750	401.7292	247.1371	0.70091	0.00013	0.020148	LOC101901750	solute carrier family 2%2C facilitated glucose

							transporter member 3 isoform X2||solute carrier family 2%2C facilitated glucose transporter member 3%2C transcript variant X2
gene-LOC105261535	309.4055	496.3112	-0.68175	0.000121	0.019301	LOC105261535	uncharacterized LOC105261535
gene-LOC105261748	98.84941	178.4914	-0.85255	0.000378	0.045356	LOC105261748	uncharacterized LOC105261748||uncharacterized protein LOC105261748
gene-LOC105261780	95.79755	222.0951	-1.2131	4.84E-05	0.009988	LOC105261780	uncharacterized LOC105261780
gene-LOC105261881	282.272	155.7988	0.8574	3.20E-05	0.007817	LOC105261881	uncharacterized LOC105261881
gene-LOC109611647	199.6608	76.29138	1.388	3.62E-07	0.000245	LOC109611647	polynucleotide 5'-hydroxyl-kinase nol9-like
gene-LOC109614089	292.9336	104.0277	1.4936	5.44E-05	0.010694	LOC109614089	uncharacterized protein LOC109614089||uncharacterized LOC109614089
gene-LOC109614127	136.3816	67.90592	1.006	0.000166	0.023675	LOC109614127	tetratricopeptide repeat protein 19 homolog%2C mitochondrial-like
gene-LOC109614258	45.89443	101.1644	-1.1403	0.000223	0.029638	LOC109614258	uncharacterized LOC109614258
gene-LOC109614336	184.5613	101.9941	0.85561	0.000276	0.035093	LOC109614336	uncharacterized LOC109614336
