# Supplementary material for: Short-term particulate matter contamination severely compromises insect antennal olfactory perception
Source: Nat Commun. 2023 Jul 11;14:4112. doi: 10.1038/s41467-023-39469-3 (PMC10336072; doi:10.1038/s41467-023-39469-3)
Supplement: Supplementary file 9 — Supplementary Data 6 [file 41467_2023_39469_MOESM9_ESM.pdf]

Supplementary Data 6. Details of differential expressed genes (DEGs) between the bodies of uncontaminated and contaminated male houseflies in spring. Readcount is the average read count of each gen, log2FoldChange is calculated by formular log2(readcount_CMB/readcount_UMB), pval is the *p* value, padj is the fdr corrected *p* value using Benjamini/Hochberg method. All *p*-values are based on two-sided tests. CMB: contaminated male body, UMB: uncontaminated male body.

**Gene_id**	**readcount_CMB**	**readcount_UMB**	**log2FoldChange**	**pval**	**padj**	**gene_name**	**description**
Novel00040	824.7825	507.8544	0.6996	2.45E-06	0.000618	-	-
Novel00395	0	9.994257	∞	0.000175	0.017044	-	-
Novel00403	169.8662	88.06408	0.94777	6.31E-05	0.008227	-	-
Novel00468	30.31882	69.5348	-1.1975	0.000489	0.036968	-	-
Novel00966	395.888	630.2736	-0.67089	0.000343	0.02836	-	-
Novel01061	125.4426	221.9263	-0.82305	0.000114	0.012835	-	-
Novel01368	9.289566	49.11089	-2.4024	8.72E-06	0.001559	-	-
Novel01724	6.278327	37.88312	-2.5931	0.000358	0.029205	-	-
Novel01932	51.5943	125.5335	-1.2828	0.000296	0.025692	-	-
gene-CYP6G4	1257.351	2135.652	-0.76429	4.92E-06	0.001037	CYP6G4	cytochrome P450 6g1-like
gene-LOC101887208	595.4578	845.043	-0.50503	0.000701	0.04762	LOC101887208	uncharacterized LOC101887208||uncharacterized protein LOC101887208
gene-LOC101887281	1359.298	951.354	0.51481	0.000174	0.017042	LOC101887281	uncharacterized LOC101887281||uncharacterized protein LOC101887281
gene-LOC101887458	3596.637	5574.826	-0.63228	1.12E-06	0.000291	LOC101887458	heat shock 70 kDa protein cognate 3%2C transcript variant X2
gene-LOC101887496	492.9988	702.9506	-0.51184	0.000495	0.03708	LOC101887496	thioredoxin-like protein 1
gene-LOC101887499	349.9047	3866.084	-3.4658	2.10E-09	1.16E-06	LOC101887499	venom carboxylesterase-6
gene-LOC101887632	1603.52	1126.546	0.50934	0.000269	0.024078	LOC101887632	uncharacterized protein LOC101887632 isoform X3||uncharacterized LOC101887632%2C transcript variant X3
gene-LOC101887634	565.2995	329.059	0.78067	2.09E-05	0.003276	LOC101887634	saccharopine dehydrogenase-like oxidoreductase%2C transcript variant X2||saccharopine dehydrogenase-like oxidoreductase isoform X2

gene-LOC101887683	8414.582	5688.467	0.56485	5.54E-06	0.001116	LOC101887683	5-aminolevulinate synthase%2C erythroid-specific%2C mitochondrial%2C transcript variant X2
gene-LOC101887696	1424.123	3870.708	-1.4425	1.20E-27	9.96E-24	LOC101887696	succinate dehydrogenase assembly factor 4%2C mitochondrial
gene-LOC101887814	372.9082	226.7388	0.71779	0.000225	0.020927	LOC101887814	tyrosine-protein phosphatase 69D
gene-LOC101887839	276.7906	785.8875	-1.5055	9.48E-13	1.21E-09	LOC101887839	venom carboxylesterase-6-like isoform X3||venom carboxylesterase-6-like%2C transcript variant X3
gene-LOC101887863	11.9759	38.30261	-1.6773	0.00043	0.033723	LOC101887863	putative pyrimidine-specific ribonucleoside hydrolase RihB||uncharacterized protein LOC101887863
gene-LOC101887872	13.1407	78.42162	-2.5772	3.42E-07	0.000106	LOC101887872	phormicin
gene-LOC101888064	694.2859	1089.555	-0.65014	0.000148	0.01499	LOC101888064	tyrosine aminotransferase isoform X1||tyrosine aminotransferase%2C transcript variant X1
gene-LOC101888070	474.4757	694.6191	-0.54989	0.000347	0.028416	LOC101888070	putative RNA-binding protein 15%2C transcript variant X2
gene-LOC101888071	562.1754	904.5263	-0.68614	4.10E-06	0.000909	LOC101888071	homogentisate 1%2C2-dioxygenase
gene-LOC101888140	3901.82	2711.245	0.52519	4.99E-05	0.006978	LOC101888140	probable fatty acid-binding protein
gene-LOC101888244	179.4198	85.34742	1.0719	1.01E-05	0.001756	LOC101888244	protein TsetseEP
gene-LOC101888307	46.21526	100.6936	-1.1235	0.000138	0.014482	LOC101888307	angiopoietin-related protein 2
gene-LOC101888363	2167.873	1339.985	0.69406	1.28E-07	4.45E-05	LOC101888363	succinyl-CoA:3-ketoacid coenzyme A transferase 1%2C mitochondrial||succinyl-CoA:3-ketoacid coenzyme A transferase 1%2C mitochondrial%2C transcript variant X2
gene-LOC101888429	236.2325	93.22224	1.3415	1.01E-06	0.000274	LOC101888429	uncharacterized LOC101888429%2C transcript variant X1||uncharacterized protein LOC101888429 isoform X1
gene-LOC101888467	14.17005	76.07867	-2.4246	0.000119	0.013069	LOC101888467	alpha-methyldopa hypersensitive protein
gene-LOC101888547	185.4583	106.9707	0.79388	0.000306	0.026111	LOC101888547	polyribonucleotide nucleotidyltransferase 1%2C mitochondrial
gene-LOC101888576	6564.592	4454.147	0.55956	3.28E-05	0.004874	LOC101888576	uncharacterized family 31 glucosidase KIAA1161
gene-LOC101888580	255.9669	119.2438	1.102	8.51E-08	3.22E-05	LOC101888580	Niemann-Pick type protein homolog 1B

gene-LOC101888600	2554.52	1836.781	0.47587	0.000289	0.025613	LOC101888600	facilitated trehalose transporter Tret1-like%2C transcript variant X1||facilitated trehalose transporter Tret1-like isoform X1
gene-LOC101888614	29290.95	15602.31	0.9087	2.74E-06	0.00066	LOC101888614	fatty acid synthase
gene-LOC101888657	1347.808	739.6602	0.86568	9.18E-10	6.36E-07	LOC101888657	sterol O-acyltransferase 1
gene-LOC101888662	7060.859	5254.794	0.42621	0.000601	0.043202	LOC101888662	uncharacterized protein LOC101888662||uncharacterized LOC101888662
gene-LOC101888695	2357.749	3721.001	-0.65828	0.000375	0.030172	LOC101888695	ATP-binding cassette sub-family G member 1
gene-LOC101888722	158.9783	258.3532	-0.70052	0.000602	0.043202	LOC101888722	gram-negative bacteria-binding protein 2 isoform X1||gram-negative bacteria-binding protein 2%2C transcript variant X1
gene-LOC101888736	3215.717	2237.457	0.52328	6.45E-05	0.008321	LOC101888736	bacchus
gene-LOC101888799	3244.658	6981.491	-1.1055	3.35E-07	0.000105	LOC101888799	phosphoenolpyruvate carboxykinase [GTP]
gene-LOC101888820	968.1698	587.4987	0.72067	0.000145	0.014892	LOC101888820	peroxisomal (S)-2-hydroxy-acid oxidase GLO5
gene-LOC101888844	5628.961	2689.641	1.0655	4.09E-17	9.73E-14	LOC101888844	uncharacterized protein LOC101888844||serine-rich adhesin for platelets
gene-LOC101888850	10695.35	7512.609	0.5096	9.03E-05	0.010891	LOC101888850	uncharacterized LOC101888850||uncharacterized protein LOC101888850
gene-LOC101888874	1511.136	2140.65	-0.50242	0.00044	0.034045	LOC101888874	general odorant-binding protein 99a
gene-LOC101888935	5402.889	9236.061	-0.77355	9.69E-06	0.001697	LOC101888935	mucin-5AC
gene-LOC101888952	320.4512	594.4187	-0.89138	0.000145	0.014892	LOC101888952	uncharacterized protein LOC101888952||uncharacterized LOC101888952
gene-LOC101888968	20565.7	13958.67	0.55908	5.11E-06	0.001062	LOC101888968	actin-5C%2C transcript variant X2
gene-LOC101888983	527.1391	344.9911	0.61162	0.00017	0.01685	LOC101888983	Myb/SANT-like DNA-binding protein%2C transcript variant X1
gene-LOC101889021	463.7106	667.4738	-0.52549	0.00049	0.036968	LOC101889021	dnaJ homolog shv
gene-LOC101889108	117.4378	218.5009	-0.89574	0.000641	0.045165	LOC101889108	uncharacterized LOC101889108||uncharacterized protein LOC101889108

gene-LOC101889139	4123.418	2086.437	0.9828	9.75E-11	9.01E-08	LOC101889139	UDP-glucose 4-epimerase
gene-LOC101889163	991.4891	1872.984	-0.91767	1.34E-11	1.39E-08	LOC101889163	uncharacterized protein LOC101889163||uncharacterized LOC101889163
gene-LOC101889200	1144.651	1587.351	-0.47171	0.000626	0.044284	LOC101889200	protein croquemort
gene-LOC101889354	426.8538	1170.256	-1.455	1.18E-09	7.01E-07	LOC101889354	lipase 3
gene-LOC101889471	1114.769	1741.678	-0.64373	3.98E-06	0.000894	LOC101889471	facilitated trehalose transporter Tret1-2 homolog
gene-LOC101889524	1631.448	999.041	0.70754	3.03E-07	9.88E-05	LOC101889524	cytochrome P450 CYP12A2-like
gene-LOC101889527	36.89881	91.37647	-1.3082	3.49E-05	0.005107	LOC101889527	lipase 3-like
gene-LOC101889548	301.8378	520.9291	-0.78731	1.97E-05	0.003126	LOC101889548	angiopoietin-related protein 2
gene-LOC101889641	423.0922	840.716	-0.99065	3.40E-09	1.72E-06	LOC101889641	gamma-glutamylcyclotransferase-like
gene-LOC101889786	2702.74	1790.866	0.59377	5.42E-06	0.001114	LOC101889786	protein clueless
gene-LOC101889812	1716.057	1175.196	0.5462	9.37E-05	0.011209	LOC101889812	uncharacterized LOC101889812||uncharacterized protein LOC101889812
gene-LOC101889853	4595.678	7675.962	-0.74007	4.91E-09	2.40E-06	LOC101889853	alpha-2-macroglobulin%2C transcript variant X14||alpha-2-macroglobulin isoform X14
gene-LOC101889893	127.8078	317.101	-1.311	6.75E-06	0.001276	LOC101889893	uncharacterized protein LOC101889893||Lectin C-type protein
gene-LOC101889924	41.74142	6.537805	2.6746	4.58E-07	0.000134	LOC101889924	probable cytochrome P450 304a1
gene-LOC101890018	62.13564	25.32333	1.295	0.000412	0.032647	LOC101890018	esterase E4
gene-LOC101890054	178.3368	65.35071	1.4483	5.88E-05	0.007949	LOC101890054	probable 4-coumarate--CoA ligase 1
gene-LOC101890125	245.8232	424.6128	-0.78853	0.000744	0.049891	LOC101890125	elongation of very long chain fatty acids protein 7
gene-LOC101890274	455.476	218.2062	1.0617	1.11E-09	7.01E-07	LOC101890274	saccharopine dehydrogenase-like oxidoreductase
gene-LOC101890301	183.7569	322.1153	-0.80978	1.55E-05	0.002542	LOC101890301	putative transcriptional regulator cudA
gene-LOC101890310	997.2308	1523.089	-0.611	3.13E-05	0.004695	LOC101890310	serine--pyruvate aminotransferase%2C mitochondrial
gene-LOC101890358	2942.274	3982.98	-0.43692	0.00073	0.049171	LOC101890358	proclotting enzyme%2C transcript variant X1
gene-LOC101890413	275.621	412.0268	-0.58005	0.000649	0.04534	LOC101890413	KH domain-containing%2C RNA-binding%2C signal transduction-associated protein 2 isoform X2||KH domain-containing%2C RNA-binding%2C signal transduction-

							associated protein 2%2C transcript variant X2
gene-LOC101890446	4653.205	2775.204	0.74563	7.89E-09	3.55E-06	LOC101890446	probable galactose-1-phosphate uridylyltransferase
gene-LOC101890698	204.9298	574.5677	-1.4873	1.99E-18	6.63E-15	LOC101890698	mitochondrial basic amino acids transporter%2C transcript variant X2||mitochondrial basic amino acids transporter isoform X2
gene-LOC101890724	2707.579	4503.914	-0.73418	2.55E-07	8.50E-05	LOC101890724	chitinase-like protein Idgf4
gene-LOC101890852	2037.656	2932.836	-0.52539	0.000607	0.04327	LOC101890852	general odorant-binding protein 56a
gene-LOC101890861	195.9333	106.9899	0.87289	6.33E-05	0.008227	LOC101890861	uncharacterized protein LOC101890861||uncharacterized LOC101890861
gene-LOC101890875	22.54469	1.892654	3.5743	5.33E-05	0.007383	LOC101890875	juvenile hormone acid O-methyltransferase-like
gene-LOC101890930	1687.134	1220.802	0.46675	0.000491	0.036968	LOC101890930	aminopeptidase N isoform X1||aminopeptidase N%2C transcript variant X1
gene-LOC101890965	396.4546	257.2764	0.62384	0.000332	0.027616	LOC101890965	myosin-VIIa
gene-LOC101891134	499.8169	305.3004	0.71117	1.59E-05	0.002565	LOC101891134	3-oxoacyl-[acyl-carrier-protein] reductase FabG%2C transcript variant X2||uncharacterized protein LOC101891134
gene-LOC101891486	3950.804	6047.194	-0.61412	0.000294	0.025692	LOC101891486	uncharacterized protein LOC101891486||uncharacterized LOC101891486
gene-LOC101891572	601.4109	955.5556	-0.66799	0.000433	0.033846	LOC101891572	membrane alanyl aminopeptidase
gene-LOC101891576	12949.09	7324.163	0.82211	2.20E-10	1.66E-07	LOC101891576	NADP-dependent malic enzyme isoform X2||NADP-dependent malic enzyme%2C transcript variant X2
gene-LOC101891596	654.2824	1191.662	-0.86499	1.51E-09	8.67E-07	LOC101891596	sodium- and chloride-dependent neutral and basic amino acid transporter B(0+)
gene-LOC101891733	9257.785	6485.726	0.5134	4.80E-05	0.006886	LOC101891733	facilitated trehalose transporter Tret1%2C transcript variant X3||facilitated trehalose transporter Tret1 isoform X2
gene-LOC101891769	239.575	387.6727	-0.69436	0.000116	0.012835	LOC101891769	uncharacterized LOC101891769
gene-LOC101891839	343.7481	544.7723	-0.6643	4.92E-05	0.006937	LOC101891839	uncharacterized LOC101891839||uncharacterized protein

							LOC101891839
gene-LOC101891858	10336.37	7644.316	0.43527	0.000533	0.039402	LOC101891858	ATP-citrate synthase%2C transcript variant X4||ATP-citrate synthase isoform X1
gene-LOC101891911	507.0284	320.127	0.66342	0.000112	0.012732	LOC101891911	multidrug resistance-associated protein 4%2C transcript variant X2
gene-LOC101891930	110.5006	54.07615	1.031	0.00017	0.01685	LOC101891930	uncharacterized LOC101891930||uncharacterized protein LOC101891930
gene-LOC101891960	363.0343	537.7219	-0.56675	0.000344	0.02836	LOC101891960	uncharacterized protein LOC101891960||uncharacterized LOC101891960
gene-LOC101891995	233.795	518.3381	-1.1486	1.31E-11	1.39E-08	LOC101891995	uncharacterized protein LOC101891995||uncharacterized LOC101891995
gene-LOC101891999	868.3708	565.7174	0.61823	3.07E-05	0.004637	LOC101891999	zinc finger protein hangover
gene-LOC101892027	1052.373	736.0965	0.51568	0.00025	0.022855	LOC101892027	probable multidrug resistance-associated protein lethal(2)03659
gene-LOC101892147	1355.447	643.4666	1.0748	2.49E-07	8.44E-05	LOC101892147	phospholipase A1
gene-LOC101892163	488.3417	739.7564	-0.59916	6.98E-05	0.008803	LOC101892163	mitochondrial coenzyme A transporter SLC25A42
gene-LOC101892235	255.8058	163.8926	0.6423	0.000701	0.04762	LOC101892235	solute carrier family 25 member 45
gene-LOC101892243	477.3667	722.2909	-0.59748	0.000103	0.011995	LOC101892243	bifunctional heparan sulfate N-deacetylase/N-sulfotransferase%2C transcript variant X3||bifunctional heparan sulfate N-deacetylase/N-sulfotransferase
gene-LOC101892265	74.62201	160.5985	-1.1058	3.73E-06	0.000875	LOC101892265	transcription factor Adf-1
gene-LOC101892322	1770.227	2904.286	-0.71425	0.000514	0.03818	LOC101892322	chitinase-like protein CG5210
gene-LOC101892399	1098.617	724.1713	0.60129	9.71E-05	0.011451	LOC101892399	facilitated trehalose transporter Tret1
gene-LOC101892461	554.4698	1117.974	-1.0117	4.48E-07	0.000133	LOC101892461	probable 4-coumarate--CoA ligase 1
gene-LOC101892611	298.109	464.707	-0.64048	0.000116	0.012835	LOC101892611	GTP:AMP phosphotransferase AK3%2C mitochondrial
gene-LOC101892627	166.7591	87.08843	0.93721	5.54E-05	0.007556	LOC101892627	uncharacterized protein LOC101892627||uncharacterized LOC101892627
gene-LOC101892660	595.9963	847.3244	-0.50761	0.000674	0.046551	LOC101892660	UDP-glucuronosyltransferase 2B31

gene-LOC101892730	108.2862	345.4979	-1.6738	2.58E-06	0.000633	LOC101892730	L-threonine 3-dehydrogenase%2C mitochondrial
gene-LOC101892747	483.9582	866.8886	-0.84096	3.04E-05	0.004637	LOC101892747	uncharacterized protein LOC101892747||uncharacterized LOC101892747
gene-LOC101892829	810.1183	555.3296	0.54479	0.000288	0.025613	LOC101892829	peptide methionine sulfoxide reductase||LOW QUALITY PROTEIN: peptide methionine sulfoxide reductase
gene-LOC101892924	227.6377	86.60012	1.3943	0.000476	0.036451	LOC101892924	uncharacterized protein LOC101892924||uncharacterized LOC101892924
gene-LOC101893067	2206.421	3765.902	-0.77129	1.20E-05	0.002043	LOC101893067	CD109 antigen
gene-LOC101893128	4.36828	28.06316	-2.6835	3.50E-05	0.005107	LOC101893128	lectin subunit alpha
gene-LOC101893200	179.8852	293.3582	-0.70559	0.000222	0.020735	LOC101893200	male accessory gland serine protease inhibitor-like
gene-LOC101893291	2437.207	1473.987	0.72551	3.58E-08	1.49E-05	LOC101893291	UDP-glucuronosyltransferase 2A3-like
gene-LOC101893323	1990.721	1317.431	0.59556	5.57E-06	0.001116	LOC101893323	monocarboxylate transporter 10%2C transcript variant X2
gene-LOC101893362	26.1612	61.47237	-1.2325	0.000581	0.042377	LOC101893362	tissue factor pathway inhibitor
gene-LOC101893410	89.7462	183.7938	-1.0342	6.05E-06	0.001185	LOC101893410	juvenile hormone acid O-methyltransferase-like
gene-LOC101893430	248.2739	383.8862	-0.62875	0.000308	0.026181	LOC101893430	monocarboxylate transporter 12
gene-LOC101893472	159.8593	317.4077	-0.98953	1.93E-05	0.003085	LOC101893472	cytochrome P450 4p1||LOW QUALITY PROTEIN: cytochrome P450 4p1
gene-LOC101893479	4162.765	2941.71	0.50089	0.000152	0.015343	LOC101893479	uncharacterized protein LOC101893479||uncharacterized LOC101893479
gene-LOC101893529	279.2253	522.8492	-0.90497	1.06E-06	0.000285	LOC101893529	kunitz-type serine protease inhibitor Hg1 isoform X1||kunitz-type serine protease inhibitor Hg1%2C transcript variant X1
gene-LOC101893605	669.9489	409.2858	0.71094	3.89E-06	0.000886	LOC101893605	uncharacterized protein LOC101893605||Ecdysteroid kinase
gene-LOC101893804	62.61793	163.483	-1.3845	0.000133	0.014225	LOC101893804	uncharacterized LOC101893804||uncharacterized protein LOC101893804
gene-LOC101893838	24.47447	146.2278	-2.5789	9.72E-07	0.000269	LOC101893838	trypsin beta-like
gene-LOC101893922	11535.3	6050.076	0.93103	7.98E-08	3.09E-05	LOC101893922	glutamine synthetase 1%2C mitochondrial

gene-LOC101894147	105.0269	362.2425	-1.7862	6.10E-20	2.54E-16	LOC101894147	transcription factor Adf-1
gene-LOC101894179	724.5336	370.9628	0.96578	7.52E-10	5.44E-07	LOC101894179	probable asparagine synthetase [glutamine-hydrolyzing]
gene-LOC101894268	96.51468	36.22211	1.4139	4.04E-05	0.005844	LOC101894268	uncharacterized LOC101894268||uncharacterized protein LOC101894268
gene-LOC101894318	241.4031	35.73782	2.7559	3.15E-09	1.64E-06	LOC101894318	uncharacterized protein LOC101894318||uncharacterized LOC101894318
gene-LOC101894451	541.8841	295.6488	0.8741	9.72E-08	3.51E-05	LOC101894451	transcription factor GAGA%2C transcript variant X2
gene-LOC101894589	78.68396	150.6333	-0.9369	0.000133	0.014225	LOC101894589	uncharacterized protein LOC101894589||uncharacterized LOC101894589
gene-LOC101894736	920.815	601.2242	0.61501	0.0004	0.031828	LOC101894736	acidic leucine-rich nuclear phosphoprotein 32 family member A%2C transcript variant X1
gene-LOC101894750	390.5155	193.7648	1.0111	1.20E-07	4.24E-05	LOC101894750	gamma-butyrobetaine dioxygenase
gene-LOC101894813	1334.649	922.9594	0.53212	0.000107	0.012242	LOC101894813	sarcocystatin-A
gene-LOC101894824	311.486	154.9045	1.0078	3.83E-06	0.000884	LOC101894824	Krueppel-like factor 10
gene-LOC101894841	449.0937	274.2322	0.71162	1.52E-05	0.00253	LOC101894841	diacylglycerol O-acyltransferase 1 isoform X1||diacylglycerol O-acyltransferase 1%2C transcript variant X1
gene-LOC101894909	859.8642	557.9981	0.62385	2.51E-05	0.003868	LOC101894909	ATP-binding cassette sub-family G member 4%2C transcript variant X9||ATP-binding cassette sub-family G member 4 isoform X2
gene-LOC101895034	2220.758	857.2243	1.3733	3.38E-06	0.000803	LOC101895034	uncharacterized LOC101895034||uncharacterized protein LOC101895034
gene-LOC101895087	675.0659	441.2726	0.61336	6.26E-05	0.008227	LOC101895087	nucleolar protein 58
gene-LOC101895125	419.7797	752.1672	-0.84142	6.84E-06	0.001278	LOC101895125	uncharacterized LOC101895125||uncharacterized protein LOC101895125
gene-LOC101895238	38116.78	28349.43	0.42711	0.000609	0.04327	LOC101895238	polyubiquitin isoform X1||polyubiquitin-C%2C transcript variant X1
gene-LOC101895241	653.9373	371.9985	0.81386	0.000123	0.013371	LOC101895241	metallothionein-2-like

gene-LOC101895253	598.8447	405.1461	0.56374	0.000323	0.026983	LOC101895253	uncharacterized protein LOC101895253||uncharacterized LOC101895253
gene-LOC101895341	513.2259	144.3304	1.8302	1.07E-24	5.92E-21	LOC101895341	uncharacterized protein LOC101895341||uncharacterized LOC101895341
gene-LOC101895374	1490.508	2992.862	-1.0057	4.91E-06	0.001037	LOC101895374	sialin
gene-LOC101895383	4022.551	1957.305	1.0392	1.72E-12	2.05E-09	LOC101895383	maltase A3
gene-LOC101895551	345.8922	170.3267	1.022	5.59E-08	2.21E-05	LOC101895551	uncharacterized protein LOC101895551||uncharacterized LOC101895551
gene-LOC101895635	678.4657	2095.354	-1.6268	2.20E-09	1.18E-06	LOC101895635	mast cell tryptase
gene-LOC101895696	208.5808	384.3496	-0.88181	4.33E-07	0.000131	LOC101895696	putative inorganic phosphate cotransporter
gene-LOC101895730	213.3268	57.24412	1.8979	3.59E-14	4.97E-11	LOC101895730	uncharacterized protein LOC101895730||uncharacterized LOC101895730
gene-LOC101895906	171.3013	41.69788	2.0385	4.47E-15	7.44E-12	LOC101895906	uncharacterized protein LOC101895906||uncharacterized LOC101895906
gene-LOC101895925	347.8012	536.3014	-0.62478	0.000114	0.012835	LOC101895925	uncharacterized protein DDB_G0271670 isoform X3||uncharacterized protein DDB_G0271670%2C transcript variant X3
gene-LOC101895933	27.40744	3.714087	2.8835	9.70E-05	0.011451	LOC101895933	probable cytochrome P450 304a1
gene-LOC101895966	111.6068	197.7591	-0.82532	0.000105	0.012103	LOC101895966	uncharacterized protein LOC101895966||uncharacterized LOC101895966
gene-LOC101896019	281.6961	113.4121	1.3126	1.30E-10	1.14E-07	LOC101896019	uncharacterized LOC101896019||uncharacterized protein LOC101896019
gene-LOC101896060	2384.056	5277.542	-1.1464	0.00025	0.022855	LOC101896060	glutamate receptor ionotropic%2C kainate 2 isoform X1||glutamate receptor ionotropic%2C kainate 2%2C transcript variant X1
gene-LOC101896218	3051.075	1795.043	0.7653	2.59E-06	0.000633	LOC101896218	tyrosine 3-monooxygenase
gene-LOC101896272	1510.157	1082.771	0.47997	0.000369	0.029806	LOC101896272	protein fem-1 homolog CG6966
gene-LOC101896308	8634.269	6345.043	0.44444	0.000299	0.025801	LOC101896308	uncharacterized protein LOC101896308||uncharacterized

							LOC101896308
gene-LOC101896354	262.9835	158.2141	0.73309	0.000137	0.014445	LOC101896354	solute carrier family 25 member 45
gene-LOC101896380	393.1527	142.4524	1.4646	5.92E-15	8.96E-12	LOC101896380	phosphatidylserine synthase 1%2C transcript variant X3||phosphatidylserine synthase 1 isoform X3
gene-LOC101896491	325.6503	540.8305	-0.73185	2.21E-05	0.003435	LOC101896491	putative uncharacterized protein DDB_G0281733
gene-LOC101896556	581.5481	855.1412	-0.55626	0.000188	0.018032	LOC101896556	putative transporter svop-1
gene-LOC101896601	2815.85	5607.015	-0.99366	1.18E-09	7.01E-07	LOC101896601	uncharacterized LOC101896601||uncharacterized protein LOC101896601
gene-LOC101896621	1236.807	1748.919	-0.49984	0.000244	0.022554	LOC101896621	receptor-type guanylate cyclase Gyc76C%2C transcript variant X4
gene-LOC101896648	265.5601	413.5625	-0.63907	0.000195	0.018504	LOC101896648	protein daughter of sevenless
gene-LOC101896821	211.4636	543.7656	-1.3626	4.19E-06	0.000918	LOC101896821	branched-chain-amino-acid aminotransferase%2C cytosolic
gene-LOC101896892	349.7456	224.9503	0.6367	0.000478	0.036451	LOC101896892	peptidoglycan-recognition protein SC2-like
gene-LOC101896932	776.3701	470.1364	0.72367	0.000174	0.017042	LOC101896932	putative metabolite transport protein YwtG||uncharacterized protein LOC101896932
gene-LOC101896933	3108.208	2243.529	0.47031	0.00031	0.026214	LOC101896933	protein D3
gene-LOC101896986	786.5711	506.5437	0.63489	0.000506	0.03773	LOC101896986	RNA-binding protein lark
gene-LOC101897017	2113.385	3022.521	-0.5162	8.95E-05	0.010886	LOC101897017	homocysteine-responsive endoplasmic reticulum-resident ubiquitin-like domain member 2 protein%2C transcript variant X3||homocysteine-responsive endoplasmic reticulum-resident ubiquitin-like domain member 2 protein isoform X3
gene-LOC101897053	156.1167	249.2387	-0.6749	0.000654	0.045496	LOC101897053	aminopeptidase N isoform X1||aminopeptidase N%2C transcript variant X1
gene-LOC101897068	55.26177	117.4574	-1.0878	0.000119	0.013067	LOC101897068	uncharacterized LOC101897068||uncharacterized protein LOC101897068
gene-LOC101897428	1089.431	460.3636	1.2427	2.65E-17	7.34E-14	LOC101897428	phosphatidylserine decarboxylase proenzyme%2C

							mitochondrial%2C transcript variant X2
gene-LOC101897506	4615.457	2599.189	0.82841	1.61E-10	1.34E-07	LOC101897506	protein pinocchio isoform X2||RecR protein%2C transcript variant X2
gene-LOC101897602	204.3313	319.6562	-0.64561	0.000368	0.029806	LOC101897602	collagen alpha-1(IV) chain
gene-LOC101897703	212.3678	343.631	-0.6943	0.000599	0.043202	LOC101897703	myoneurin
gene-LOC101897734	311.4534	132.1862	1.2364	1.76E-10	1.39E-07	LOC101897734	G-box-binding factor%2C transcript variant X4
gene-LOC101897743	1030.746	1473.96	-0.51601	0.000127	0.013759	LOC101897743	neuropathy target esterase sws%2C transcript variant X2||neuropathy target esterase sws isoform X1
gene-LOC101897758	451.8332	296.2418	0.60901	0.000417	0.032853	LOC101897758	fibronectin type-III domain-containing protein 3a
gene-LOC101897807	1182.994	1812.045	-0.61518	6.81E-05	0.008644	LOC101897807	phosphofurin acidic cluster sorting protein 2
gene-LOC101897924	333.6344	217.1729	0.61942	0.000558	0.040919	LOC101897924	Hemolymph juvenile hormone binding protein (JHBP)%2C transcript variant X2||uncharacterized protein LOC101897924 isoform X2
gene-LOC101897981	1611.478	992.7008	0.69895	3.10E-07	9.90E-05	LOC101897981	serine proteases 1/2
gene-LOC101898046	74.05115	147.1152	-0.99035	4.86E-05	0.006912	LOC101898046	atrial natriuretic peptide receptor 2
gene-LOC101898132	508.2539	815.1629	-0.68154	6.15E-06	0.001189	LOC101898132	fatty acyl-CoA reductase wat
gene-LOC101898159	653.1476	947.1316	-0.53616	0.000257	0.02328	LOC101898159	50 kDa gamma-zein
gene-LOC101898182	676.1783	362.3187	0.90014	6.19E-09	2.86E-06	LOC101898182	facilitated trehalose transporter Tret1-2 homolog
gene-LOC101898279	485.0989	294.3281	0.72085	8.81E-06	0.001559	LOC101898279	putative inorganic phosphate cotransporter
gene-LOC101898346	750.4374	349.6638	1.1018	6.62E-07	0.00019	LOC101898346	organic cation transporter protein
gene-LOC101898347	528.4349	776.3284	-0.55494	0.000187	0.018032	LOC101898347	esterase B1
gene-LOC101898362	127.0945	68.87664	0.88382	0.000699	0.04762	LOC101898362	dual specificity tyrosine-phosphorylation-regulated kinase 1A%2C transcript variant X5||dual specificity tyrosine-phosphorylation-regulated kinase 1A isoform X2
gene-LOC101898389	1319.3	923.4261	0.5147	0.000265	0.023835	LOC101898389	L-galactose dehydrogenase
gene-LOC101898421	757.4678	502.8709	0.591	0.000102	0.011995	LOC101898421	uncharacterized protein LOC101898421||uncharacterized LOC101898421
gene-LOC101898651	6935.771	4282.836	0.69549	3.97E-08	1.61E-05	LOC101898651	protein TsetseEP isoform X2||protein TsetseEP%2C

							transcript variant X2
gene-LOC101898663	1673.93	3490.691	-1.0603	2.00E-16	3.70E-13	LOC101898663	carnitine O-palmitoyltransferase 1%2C liver isoform%2C transcript variant X2
gene-LOC101898763	53.80367	136.2896	-1.3409	8.38E-05	0.01033	LOC101898763	uncharacterized protein LOC101898763||Apolipophorin-3 precursor
gene-LOC101898790	397.2288	265.646	0.58047	0.000684	0.046989	LOC101898790	serine/arginine repetitive matrix protein 2
gene-LOC101898969	2153.391	1203.605	0.83925	5.38E-05	0.007397	LOC101898969	uncharacterized LOC101898969||uncharacterized protein LOC101898969
gene-LOC101899115	271.1103	416.0683	-0.61794	0.00044	0.034045	LOC101899115	uncharacterized protein LOC101899115||uncharacterized LOC101899115
gene-LOC101899169	1154.483	1634.174	-0.50131	0.000203	0.01919	LOC101899169	endoplasmin homolog
gene-LOC101899240	436.3971	900.2189	-1.0446	3.04E-11	2.97E-08	LOC101899240	carbohydrate sulfotransferase 13 isoform X1||carbohydrate sulfotransferase 13%2C transcript variant X1
gene-LOC101899359	332.854	1362.097	-2.0329	0.000305	0.026111	LOC101899359	pancreatic triacylglycerol lipase
gene-LOC101899411	901.6198	1945.363	-1.1094	5.65E-06	0.001118	LOC101899411	protein SERAC1
gene-LOC101899548	1196.858	1704.213	-0.50985	0.000219	0.020552	LOC101899548	lysozyme c-1%2C transcript variant X1
gene-LOC101899651	80.85141	166.8484	-1.0452	7.48E-06	0.001368	LOC101899651	uncharacterized LOC101899651||uncharacterized protein LOC101899651
gene-LOC101899695	1184.63	1838.061	-0.63375	2.43E-06	0.000618	LOC101899695	electron transfer flavoprotein regulatory factor 1
gene-LOC101899755	124.4379	55.91132	1.1542	7.81E-05	0.009763	LOC101899755	prion-like-(Q/N-rich) domain-bearing protein 25
gene-LOC101899837	2556.834	1554.591	0.71782	0.000648	0.04534	LOC101899837	maltase A3-like
gene-LOC101899863	10.4718	40.52717	-1.9524	6.29E-05	0.008227	LOC101899863	peritrophin-48
gene-LOC101899872	193.3666	451.4282	-1.2232	4.59E-06	0.000991	LOC101899872	serine protease snake
gene-LOC101900043	337.5133	525.3727	-0.6384	7.99E-05	0.009923	LOC101900043	uncharacterized LOC101900043
gene-LOC101900137	629.59	938.7828	-0.57638	6.67E-05	0.008532	LOC101900137	membrane metallo-endopeptidase-like 1
gene-LOC101900145	702.3555	988.9069	-0.49363	0.000662	0.0459	LOC101900145	juvenile hormone epoxide hydrolase 1
gene-LOC101900255	2681.447	1579.392	0.76364	5.07E-09	2.41E-06	LOC101900255	alkaline phosphatase 4
gene-LOC101900355	13.12371	55.13634	-2.0708	0.000144	0.014892	LOC101900355	adult cuticle protein 1-like

gene-LOC101900379	731.065	1335.86	-0.8697	6.30E-06	0.001204	LOC101900379	serine protease inhibitor 77Ba
gene-LOC101900480	32.54014	117.1524	-1.8481	9.90E-10	6.59E-07	LOC101900480	endothelin-converting enzyme 2
gene-LOC101900522	589.1553	1018.933	-0.79034	1.11E-06	0.000291	LOC101900522	uncharacterized protein LOC101900522||uncharacterized LOC101900522
gene-LOC101900528	262.4318	444.2018	-0.75927	7.26E-06	0.001342	LOC101900528	5'-nucleotidase domain-containing protein 1
gene-LOC101900551	501.0826	873.5526	-0.80185	8.96E-08	3.31E-05	LOC101900551	endothelin-converting enzyme 2
gene-LOC101900701	151.9761	86.09661	0.81982	0.000549	0.04038	LOC101900701	uncharacterized protein LOC101900701 isoform X1||uncharacterized LOC101900701%2C transcript variant X1
gene-LOC101900728	70.92986	157.1883	-1.148	0.000135	0.014337	LOC101900728	cytochrome P450 18a1
gene-LOC101900869	327.1122	170.364	0.94117	0.000602	0.043202	LOC101900869	probable multidrug resistance-associated protein lethal(2)03659
gene-LOC101901021	492.4569	750.2876	-0.60745	0.000297	0.025692	LOC101901021	myotubularin-related protein 14
gene-LOC101901050	35.62195	98.35311	-1.4652	0.000392	0.031323	LOC101901050	uncharacterized LOC101901050||uncharacterized protein LOC101901050
gene-LOC101901284	18.91683	78.0067	-2.0439	1.73E-08	7.37E-06	LOC101901284	shematrin-like protein 2
gene-LOC101901326	1303.043	1792.805	-0.46033	0.000725	0.048996	LOC101901326	lamin-C isoform X1||lamin-C%2C transcript variant X1
gene-LOC101901413	27.86797	73.08823	-1.391	8.96E-05	0.010886	LOC101901413	cuticle protein 16.5-like
gene-LOC101901419	408.6311	82.60319	2.3065	1.51E-16	3.15E-13	LOC101901419	4-coumarate--CoA ligase-like 7
gene-LOC101901604	311.7881	519.3686	-0.73619	8.37E-06	0.001514	LOC101901604	BTB/POZ domain-containing protein 9
gene-LOC101901626	81.90616	146.0658	-0.83458	0.000453	0.0349	LOC101901626	serine protease persephone%2C transcript variant X1||serine protease persephone isoform X1
gene-LOC101901660	263.7664	466.638	-0.82304	7.14E-07	0.000201	LOC101901660	endothelin-converting enzyme-like 1
gene-LOC101901690	962.1331	1371.916	-0.51188	0.000191	0.018246	LOC101901690	retinol-binding protein pinta
gene-LOC101901752	57.2617	123.7968	-1.1123	1.39E-05	0.002338	LOC101901752	transcription initiation factor TFIID subunit 11
gene-LOC105261419	974.5639	600.066	0.69964	1.14E-05	0.001962	LOC105261419	high mobility group protein D%2C transcript variant X1
gene-LOC105261546	65.59855	527.7535	-3.0081	3.20E-42	5.32E-38	LOC105261546	uncharacterized protein LOC105261546||uncharacterized LOC105261546

gene-LOC105261589	582.8617	390.6117	0.57742	0.000294	0.025692	LOC105261589	uncharacterized protein LOC105261589||uncharacterized LOC105261589
gene-LOC105261940	136.5309	245.2068	-0.84477	0.000252	0.022909	LOC105261940	uncharacterized protein LOC105261940||uncharacterized LOC105261940
gene-LOC105261977	7.900013	31.362	-1.9891	0.000321	0.026934	LOC105261977	uncharacterized protein LOC105261977||uncharacterized LOC105261977
gene-LOC105262484	23.90547	73.23807	-1.6153	0.000153	0.015343	LOC105262484	mitochondrial uncoupling protein 4-like
gene-LOC109611602	236.5969	103.1957	1.197	1.62E-08	7.08E-06	LOC109611602	4-coumarate--CoA ligase 1-like
gene-LOC109612482	101.0242	217.1423	-1.1039	0.000146	0.014892	LOC109612482	uncharacterized LOC109612482
gene-LOC109612838	235.5254	100.178	1.2333	1.56E-05	0.002542	LOC109612838	uncharacterized protein LOC109612838||uncharacterized LOC109612838
gene-LOC109614215	2714.967	3898.462	-0.52197	5.97E-05	0.008008	LOC109614215	uncharacterized LOC109614215||uncharacterized protein LOC109614215
