# Supplementary material for: Short-term particulate matter contamination severely compromises insect antennal olfactory perception
Source: Nat Commun. 2023 Jul 11;14:4112. doi: 10.1038/s41467-023-39469-3 (PMC10336072; doi:10.1038/s41467-023-39469-3)
Supplement: Supplementary file 10 — Reporting Summary [file 41467_2023_39469_MOESM10_ESM.pdf]

nature portfolio

Corresponding author(s): Dong Zhang

Last updated by author(s): Jun 15, 2023

Reporting Summary

Nature Portfolio wishes to improve the reproducibility of the work that we publish. This form provides structure for consistency and transparency in reporting. For further information on Nature Portfolio policies, see our Editorial Policies and the Editorial Policy Checklist.

Statistics

For all statistical analyses, confirm that the following items are present in the figure legend, table legend, main text, or Methods section.

n/a	Confirmed	
☐	☒	The exact sample size (*n*) for each experimental group/condition, given as a discrete number and unit of measurement
☐	☒	A statement on whether measurements were taken from distinct samples or whether the same sample was measured repeatedly
☐	☒	The statistical test(s) used AND whether they are one- or two-sided<br>*Only common tests should be described solely by name; describe more complex techniques in the Methods section.*
☐	☒	A description of all covariates tested
☐	☒	A description of any assumptions or corrections, such as tests of normality and adjustment for multiple comparisons
☐	☒	A full description of the statistical parameters including central tendency (e.g. means) or other basic estimates (e.g. regression coefficient) AND variation (e.g. standard deviation) or associated estimates of uncertainty (e.g. confidence intervals)
☐	☒	For null hypothesis testing, the test statistic (e.g. *F*, *t*, *r*) with confidence intervals, effect sizes, degrees of freedom and *P* value noted<br>*Give P values as exact values whenever suitable.*
☒	☐	For Bayesian analysis, information on the choice of priors and Markov chain Monte Carlo settings
☐	☒	For hierarchical and complex designs, identification of the appropriate level for tests and full reporting of outcomes
☒	☐	Estimates of effect sizes (e.g. Cohen's *d*, Pearson's *r*), indicating how they were calculated

Our web collection on statistics for biologists contains articles on many of the points above.

Software and code

Policy information about availability of computer code

Data collection	Electroantennogram (EAG) data was collected using Autospike 3.4 and the highest action potential change of each trial was determined by software EagPro (Syntech, Buchenbach, Germany).
Data analysis	Most statistical anlysis was conducted using JMP Pro 14.2.0 unless otherwise specified.

For manuscripts utilizing custom algorithms or software that are central to the research but not yet described in published literature, software must be made available to editors and reviewers. We strongly encourage code deposition in a community repository (e.g. GitHub). See the Nature Portfolio guidelines for submitting code & software for further information.

Data

Policy information about availability of data

All manuscripts must include a data availability statement. This statement should provide the following information, where applicable:

- Accession codes, unique identifiers, or web links for publicly available datasets
- A description of any restrictions on data availability
- For clinical datasets or third party data, please ensure that the statement adheres to our policy

The SEM, behaviour, EAG, and RT-QPCR data generated in this study have been deposited in the Open Science Framework database under accession code https://osf.io/v92xe/?view_only=0744a389866a4cdcb602ae967fe61960. The transcriptome data generated in this study have been deposited in the NCBI database under accession code PRJNA909937 https://www.ncbi.nlm.nih.gov/bioproject/PRJNA909937. The SEM, EDX, behaviour, and transcriptome data generated in this study

are provided in the Supplementary Information/Source Data file. The world PM pollution level datasets are accessible via link https://sites.wustl.edu/acag/datasets/surface-pm2-5/#V4.GL.03, and the KEGG pathway database is accessible via link https://www.genome.jp/kegg-bin/show_organism?org=mde.

Human research participants

Policy information about studies involving human research participants and Sex and Gender in Research.

Reporting on sex and gender	No human subjects was involved.
Population characteristics	*Describe the covariate-relevant population characteristics of the human research participants (e.g. age, genotypic information, past and current diagnosis and treatment categories). If you filled out the behavioural & social sciences study design questions and have nothing to add here, write "See above."*
Recruitment	*Describe how participants were recruited. Outline any potential self-selection bias or other biases that may be present and how these are likely to impact results.*
Ethics oversight	*Identify the organization(s) that approved the study protocol.*

Note that full information on the approval of the study protocol must also be provided in the manuscript.

Field-specific reporting

Please select the one below that is the best fit for your research. If you are not sure, read the appropriate sections before making your selection.

☐ Life sciences ☐ Behavioural & social sciences ☒ Ecological, evolutionary & environmental sciences

For a reference copy of the document with all sections, see nature.com/documents/nr-reporting-summary-flat.pdf

Ecological, evolutionary & environmental sciences study design

All studies must disclose on these points even when the disclosure is negative.

Study description	This study compares the sensory functions of houseflies (Musca domestica L.) exposed briefly to urban particulate matter pollution. The laboratory houseflies were randomly allocated into two groups either exposed to urban particulate matter pollution or not, and the each experiment was conducted in three different batches to increase the representativeness.
Research sample	Research samples include wild houseflies (Musca domestica L.) of both sexes captured between June and July 2020, and laboratory population of wild type houseflies of both sexes kept in the Chinese Centre for Disease Control and Prevention.
Sampling strategy	At all times we ensured we had large sample sizes, since we could not fully control for the quantity of PM on the antennae of flies from the 'contamination' group. Accordingly, we obtained data from 50 flies for each treatment in the y-maze choice assays, as this would minimise the likelihood of false negatives arising from the requirement of very high effect sizes in binary choice with sample sizes <20. This issue is not so significant for the EAG experiments, with a continuous response variable, and so our sample sizes are smaller (15 trials for each treatment). We collected the three biological replicas in our transcriptomic analysis and included both males and females to increase generality.
Data collection	G.T.L., J.B.W., W.Y.P., and W.T.X. obtained SEM images and performed EDX analysis; G.T.L. performed behavioural and EAG experiments; the procedures are described in detail in Methods section.
Timing and spatial scale	All our tests including field collections were conducted between March and November when houseflies naturally occurs in Beijing.
Data exclusions	In behavioural assay, we discarded trials in which the individual did not enter either arms of the y-maze. In the EAG analysis, we excluded any trials with baseline lower than solvent response.
Reproducibility	All the results were compared with and validated by the pilot experiments of the similar sample size. The procedures of imaging, behavioural assays, EAG and transcriptomic analysis followed standard protocols published repeatedly in previous research. All the tests were conducted on at least three batches of flies.
Randomization	Treatments were allocated by collecting individuals from batches of over 1000 freshly hatched flies in a large fly cage.
Blinding	The behavioural assays were conducted with the observer blind to the treatment, and for EAG analysis, the data was recorded automatically using software.

Did the study involve field work? ☒ Yes ☐ No

Field work, collection and transport

Field conditions	Wild houseflies (Musca domestica L.) were captured between June and July 2020, when AQIs (Air Quality Index) conditions in Beijing were low (0-50), moderate (51-100), and heavy (101-150).
Location	Urban area of Beijing.
Access & import/export	No permits are needed.
Disturbance	We only collected some houseflies, the most common pest in the area.

Reporting for specific materials, systems and methods

We require information from authors about some types of materials, experimental systems and methods used in many studies. Here, indicate whether each material, system or method listed is relevant to your study. If you are not sure if a list item applies to your research, read the appropriate section before selecting a response.

Materials & experimental systems

n/a	Involved in the study
☒	☐ Antibodies
☒	☐ Eukaryotic cell lines
☒	☐ Palaeontology and archaeology
☐	☒ Animals and other organisms
☒	☐ Clinical data
☒	☐ Dual use research of concern

Methods

n/a	Involved in the study
☒	☐ ChIP-seq
☒	☐ Flow cytometry
☒	☐ MRI-based neuroimaging

Animals and other research organisms

Policy information about studies involving animals; ARRIVE guidelines recommended for reporting animal research, and Sex and Gender in Research

Laboratory animals	Wild type houseflies (Musca domestica L.) population of both sexes kept in the Chinese Centre for Disease Control and Prevention.
Wild animals	Wild houseflies (Musca domestica L.) of both sexes captured between June and July 2020. Samples were immobilized and pinned using standard procedures.
Reporting on sex	The findings in this study was based on both sexes of houseflies, which was determined using morphological traits of their genitalia. We compared the impacts of PM to both male and female houseflies, all the analysis were performed with sex as a factor.
Field-collected samples	Samples were immobilized and pinned using standard procedures up on collection.
Ethics oversight	We observe the relavent ethics guidelines of both Beijing Forestry University and University of Melbourne.

Note that full information on the approval of the study protocol must also be provided in the manuscript.
